# Genetic structure and evolution of the Vps25 family, a yeast ESCRT-II component

**DOI:** 10.1186/1471-2148-6-59

**Published:** 2006-08-04

**Authors:** Ruth Slater, Naomi E Bishop

**Affiliations:** 1 Faculty of Life Sciences, Michael Smith Building, University of Manchester, Oxford Road, Greater Manchester M13 9PT, UK

## Abstract

**Background:**

Vps25p is the product of yeast gene *VPS25 *and is found in an endosomal sorting complex required for transport (ESCRT)-II, along with Vps22p and Vps36p. This complex is essential for sorting of ubiquitinated biosynthetic and endosomal cargoes into endosomes.

**Results:**

We found that *VPS25 *is a highly conserved and widely expressed eukaryotic gene, with single orthologs in chromalveolate, excavate, amoebozoan, plant, fungal and metazoan species. Two paralogs were found in *Trichomonas vaginalis*. An ortholog was strikingly absent from the *Encephalitozoon cuniculi *genome. Intron positions were analyzed in *VPS25 *from 36 species. We found evidence for five ancestral *VPS25 *introns, intron loss, and single instances of intron gain (a *Paramecium *species) and intron slippage (*Theileria *species). Processed pseudogenes were identified in four mammalian genomes, with a notable absence in the mouse genome. Two retropseudogenes were found in the chimpanzee genome, one more recently inserted, and one evolving from a common primate ancestor. The amino acid sequences of 119 Vps25 orthologs are aligned, compared with the known secondary structure of yeast Vps25p, and used to carry out phylogenetic analysis. Residues in two amino-terminal PPXY motifs (motif I and II), involved in dimerization of Vps25p and interaction with Vps22p and Vps36p, were closely, but not absolutely conserved. Specifically, motif I was absent in Vps25 homologs of chromalveolates, euglenozoa, and diplomonads. A highly conserved carboxy-terminal lysine was identified, which suggests Vps25 is ubiquitinated. Arginine-83 of yeast Vps25p involved in Vps22p interaction was highly, but not absolutely, conserved. Human tissue expression analysis showed universal expression.

**Conclusion:**

We have identified 119 orthologs of yeast Vps25p. Expression of mammalian *VPS25 *in a wide range of tissues, and the presence in a broad range of eukaryotic species, indicates a basic role in eukaryotic cell function. Intron splice site positions were highly conserved across all major eukaryotic species, suggesting an ancestral origin. Amino acid sequence analysis showed the consensus for the amino-terminal proline-rich motifs is P- [WP]-X-[YF] for motif I (when present) and P-P-[FYL]-[FY] for motif II, and that Vps25 may be ubiquitinated.

## Background

The endosomal pathway of eukaryotic cells receives both biosynthetic cargo from the trans-Golgi network and endocytic cargo from the cell surface [1,2]. Many cargo proteins are further sorted into the internal vesicles that are a prominent morphological characteristic of the late endosome/multivesicular body (MVB) [3,4]. After fusion of MVBs with lysosomes, or the vacuole (which is the equivalent compartment in yeast), the internal vesicles and their contents are degraded. Sorting into MVB lumenal vesicles is a crucial process in determining whether a membrane-associated protein is degraded or recycled. In mammalian cells, MVB sorting is essential for the downregulation of activated cell surface receptors, such as the epidermal growth factor receptor, and defective sorting is strongly associated with carcinogenesis [5].

Many of the components of the cellular machinery required for endosomal sorting have now been identified. In the budding yeast *Saccharomyces cerevisiae *(hereafter referred to as 'yeast'), three complexes known as ESCRT-I (endosomal sorting complex required for transport-I), ESCRT-II and ESCRT-III, have been shown to be essential for sorting of ubiquitinated cargo [6-9] into lumenal vesicles of endosomes and/or their formation [6,10,11]. ESCRT-I initiates sorting into endosomal lumenal vesicles by binding ubiquitinated membrane proteins. ESCRT-II, composed of Vps22p (also known as Snf8p), Vps25p and Vps36p [9,12,13], functions downstream of ESCRT-I, and regulates the formation of ESCRT-III [9]. Mammalian equivalents of ESCRT-II components have also been found, known as Eap30 (Vps22p homolog), Eap20 (Vps25), and Eap45 (Vps36) [14,15]. The ESCRT complexes are conserved in mammalian cells [4], and ESCRT-I and -III have been found essential for the budding of a wide range of viruses, including HIV-1 [16-19].

The crystal structure of yeast ESCRT-II has recently been solved [12,13]. The complex has a trilobed 'Y'-shaped topology and consists of two copies of Vps25p, forming two protrusions, and one each of Vps22p and Vps36p, which form the third protrusion. While two copies of Vps25 are required to form the complex, the two Vps25p subunits make no direct contact with each other, with one Vps25p subunit binds Vps22p and the other Vps36p. The most extensive interactions within ESCRT-II are between Vps22p and Vps36p. However, as Vps25p binds both Vps22p and Vps36p, it may facilitate assembly of ESCRT-II. Although these data revealed that yeast Vps25p has a dual "winged helix" structure and two PPXY motifs [12,13,20], Vps25p has no obvious functional motifs [9].

We aimed to characterize the Vps25 family of proteins across the species, to find out more about its genetic structure and evolution, and to identify conserved residues and motifs that may have relevance to the function of this protein. *VPS25 *is a good choice for evolutionary studies for a number of reasons. First, the coding region is a compact size, with single expressed sequence tag (EST) sequence reads often covering the full coding region, meaning data can be garnered from a large range of species. Second, the genomic sequence of *VPS25 *in many major eukaryotic groups contain introns, which enables analyses of intron evolution. Thirdly, the range of proteins used for eukaryote-wide evolutionary studies have, to date, been quite restricted and included very few trafficking proteins [21-23]. Evolutionary analyses of more proteins involved in subcellular trafficking will ensure evolutionary data are more representative. Finally, we identify *VPS25 *genes in a wide variety of organisms many with known medical, agricultural, and economic importance, which will aid insights into its function in these organisms.

No studies have previously reported on the phylogenetic relationship and evolutionary history of the Vps25 family. To deduce evolutionary constraints on, and changes in, Vps25 primary structure, we use comparative genomics methods involving computer programs that line up multiple sequences and look for regions of similarity among them. A family tree of the Vps25 family was created and evolutionary history examined further by analysing the structure of mapped genes, revealing a highly conserved intron-exon organization. Finally, the expression profile of mammalian *Vps25 *genes was determined.

Our study is of further importance for a number of reasons. First, the mammalian MVB sorting machinery appears overall to be more complex than that of yeast, with the human genome expressing several homologs of several of the yeast ESCRT components [24]. In order to examine the function of mammalian ESCRT-II subunits, it is therefore necessary to identify all members of each subunit family, determine how similar they are in both sequence and expression to the yeast proteins, and trace their evolutionary history. For example, if paralogs exist, functionality may not be conserved for each protein. This is particularly relevant for ESCRT-II subunits because, by contrast to the endosomal function of yeast ESCRT-II [9], mammalian ESCRT-II was originally identified as a having a nuclear location [14,15]. This suggests that mammalian Vps25 homologs (and other ESCRT-II subunits) could have a different functionality. Secondly, there is a precedent for significant structural reorganization of the Vps36 ESCRT-II subunit through evolution. Vps36 proteins of higher species lacks the NZF ubiquitin-binding motif found in yeast Vps36p [25], and have an alternative ubiquitin-binding motif, GLUE [26]. Therefore it is necessary to look for alterations in other ESCRT-II subunits, such as Vps25. Finally, the greater relevance of the recent structural data is entirely dependent on the conservation of crucial residues of Vps25p in other Vps25 family members. Our study has determined which residues and motifs are conserved within the Vps25 family. Of great interest is an absolutely conserved lysine residue, which considering the role in ESCRT-II in the sorting of ubiquitinated proteins, suggests Vps25 may be ubiquitinated. Our data have further demonstrated that mammalian Vps25 proteins are true orthologs of yeast Vps25p and are therefore predicted to have conserved function. Expression profiling of human Vps25 showed expression in a wide variety of tissue types. Overall, these results are consistent with a conserved and fundamental role of Vps25 in organisms other than yeast.

## Results

### Identification of Vps25p orthologs

**Figure 1:**
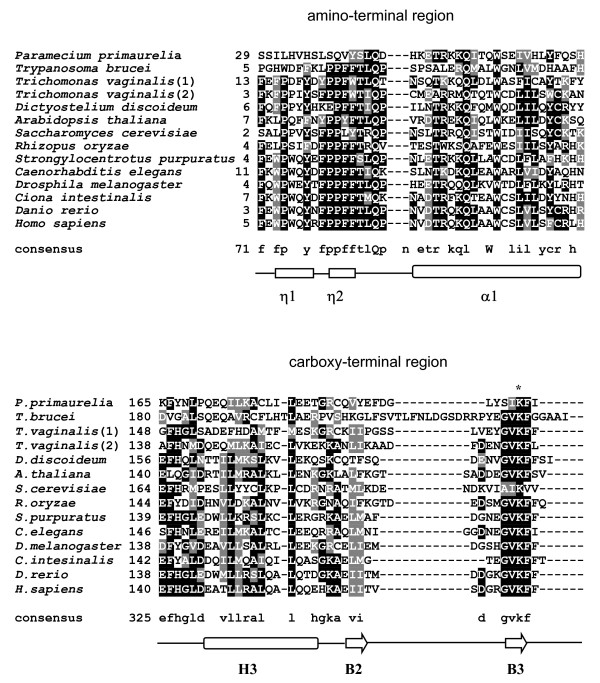
**Comparison of Vps25 amino acid sequences**. A multiple alignment of 119 Vps25 homologs was generated using ClustalX. Only the amino-terminal region (top) and carboxy-terminal region (bottom) of an abridged number of species is presented here. A full alignment of all known Vps25 homologs can be found in Additional File 18. Amino acid numbering is to the left of aligned sequences. Consensus amino acid sequences (obtained from the alignment of Vps25 from all species) are found below the alignment, where an uppercase letter represent the only completely conserved amino acid in the regions aligned [see Additional File 18]. Shading was done using Boxshade, where regions of greater than 50% conservation in the total alignment [Additional File 18] are shaded (identical amino acids are in black boxes and similar amino acids in gray boxes). Protein accession numbers are found in Additional File 1. Gaps that were required for optimal alignment of the full length Vps25 homologs are indicated by dashes. Standard single letter amino acid abbreviations are used. The secondary structure elements of yeast Vps25p are provided beneath the alignment. The two 3/10 helices (η1 and η2) prior to the first winged helix (WH domain) and the first alpha helix (α1) of WH-1 are in the aligned amino-terminal region, while helix (H2) and the final two beta strands (B2 and B3) of WH-2 are in the aligned carboxy-terminal region. An asterisk indicates the position of a highly conserved lysine residue close to the carboxy-terminus.

The *VPS25 *gene product of yeast has predicted orthologs in both the rat (Eap20) and human (HsVps25) genomes [14,15,17,18] and twenty eight correctly annotated orthologs are found listed on the Pfam database [27] under accession number PF05871. The PF05871 family consensus is referred to as the DUF852 domain on the Conserved Domain Database (CDD) nomenclature [28,29]. To characterize the Vps25 family, we used Basic Local Alignment Search Tool (BLAST) and keyword searches of a wide range of databases to identify sequences homologous to yeast and human Vps25. Reciprocal searches using each extracted protein confirmed that all members of the Vps25 family currently on the database were identified and that no false positives were included. In total 119 full length orthologs were identified in a wide range of eukaryotic species (Figure 1) [Additional Files 1, 2 and 18]. The species include representatives of all the major eukaryotic groupings, with the exclusion of the Rhizaria, for which only a very limited amount of sequence data is currently available. Vps25 orthologs were absent from the 'completed' genomes of *Plasmodium falciparum *and *Encephalitozoon cuniculi*, although a possible partial homolog may be present in *Plasmodium *species [Additional File 1]. A further 48 partial Vps25 orthologs were also identified [Additional Files 3 and 4]. The coding region of *Homo sapiens, Mus musculus*, *Rattus norvegicus*, and *Bos taurus VPS25 *begins with two ATGs separated by a triplet encoding a single amino acid. By comparison to other Vps25 proteins, the first ATG appears most likely to be used. The nucleotide sequence surrounding the first ATG is also closer to that of a Kozak consensus [30]. Pairwise BLASTP E-values indicate the similarity within the Vps25 family members and were used to compare members of the Vps25 family (Table 1). Significant pair-wise similarity for a given Vps25 protein was found always to be limited to a subset of the most closely related sequences, and no Vps25 ortholog showed significant E-values (of less than 10) when it was compared to all other Vps25 orthologs. These similarities are best represented in phylogenetic analysis (see below).

**Table 1:** Physical characteristics of Vps25 family and similarity to orthologs from key species.

Species^a^	Length (amino acids)	pI^b^	Molecular mass^c ^(kDa)	BLASTP^d ^similarity to^e^:					
				
				*S. cerevisiae*	*H. sapiens*	*P. primaurelia*	*T. brucei*	*D. discoideum*	*A. thaliana*
**CHROMALVEOLATES**									
**Alveolates**									
Apicomplexans									
*Theileria annulata*	249	8.3	29.0	0.53	0.7	>10^f^	>10	>10	1.2
*Theileria parva*	249	8.1	29.0	4.5	3.5	>10	>10	>10	3.5
Ciliates									
*Paramecium primaurelia*	201	6.3	23.5	0.006	5e-09	-^g^	>10	1e-09	4e-04
**EXCAVATES**									
**Euglenozoa**									
Kinetoplasts									
*Leishmania brazilia*	230	6.5	25.2	>10	>10	>10	1e-63	1.0	0.055
*Leishmania infantum*	230	6.7	25.4	>10	2.3	>10	7e-64	3.9	6e-04
*Leishmania major*	230	7.0	25.5	>10	3.0	>10	5e-62	2.3	0.005
*Trypanosoma brucei*	234	6.6	26.1	>10	8e-04	>10	-	2e-04	0.015
*Trypanosoma brucei gambiae*	234	6.6	26.1	>10	8e-04	>10	4e-73	2e-04	0.015
*Trypanosoma congolese*	301	7.1	26.1	>10	0.067	>10	8e-77	0.005	7e-04
*Trypanosoma cruzi*	240	7.5	26.2	>10	>10	>10	3e-73	>10	>10
*Trypanosoma vivax*	232	6.5	25.6	>10	0.014	>10	3e-73	0.27	0.019
**Diplomonads**									
*Giardia lamblia*	185	5.8	21.4	>10	3e-08	>10	>10	7e-05	1e-05
**Parabasalia**									
*Trichomonas vaginalis(1)*	186	6.6	21.7	2e-09	7e-22	8e-07	>10	2e-18	2e-15
*Trichomona vaginalis(2)*	176	7.7	20.1	3e-16	1e-34	3e-11	0.038	2e-27	1e-25
**AMOEBOZOA**									
**Entamoebidae**									
*Entamoeba histolytica*	171	6.3	20.2	7e-08	6e-16	3e-05	1.5	6e-13	8e-13
**Eumycetozoa**									
*Dictyostelium discoideum*	194	7.8	23.3	3e-19	3e-32	5e-08	2e-04	-	3e-28
**PLANTAE**									
**Green algae**									
*Chlamydomonas reinhardtii*	175	6.8	19.9	9e-12	8e-29	0.013	4e-04	8e-32	3e-48
**Rhodophyceae**									
*Cyanidioschyzon merolae*	209	8.3	24.2	1e-04	2e-18	>10	0.015	2e-20	3e-17
**Land plants**e									
**Bryophyta**									
*Physcomitrella patens*	175	5.5	20.6	1e-09	5e-23	1e-04	6.0	3e-24	4e-58
**Gnetophyta**									
*Welwitschia mirabilis*	181	7.6	21.0	2e-13	6e-30	0.002	9e-05	2e-31	7e-71
**Pinophyta**									
*Pinus taeda*	176	7.4	20.4	3e-12	3e-30	4e-05	2e-04	6e-29	3e-75
**Magnoliophyta**									
Liliopsida									
Asparagales									
*Asparagus officinalis*	179	6.9	20.8	1e-11	4e-27	1e-05	0.002	7e-28	6e-80
Cyperales									
*Hordeum vulgare*	177	6.3	20.7	2e-12	3e-28	4e-05	0.006	2e-25	4e-74
*Oryza sativa*	179	6.6	20.8	9e-15	9e-29	4e-05	0.005	3e-30	2e-80
*Sorghum bicolor*	177	6.8	20.6	1e-13	3e-28	0.001	0.003	8e-29	6e-77
*Saccharum officinarum*	177	6.8	20.6	3e-14	3e-28	0.001	0.003	8e-29	2e-76
*Triticum aestivum*	177	6.9	20.7	9e-12	2e-27	3e-05	0.013	1e-25	3e-74
Eudicotyledons									
Asteridae									
*Antirrhinum majus*	179	6.2	20.8	5e-16	2e-26	1e-04	0.004	5e-30	5e-84
*Coffea canephora*	179	6.9	20.8	7e-12	1e-26	5e-06	0.04	3e-29	2e-82
*Lycopersicon esculentum*	179	6.7	20.7	5e-14	3e-28	4e-05	0.008	8e-30	4e-85
Rosidae									
*Arabidopsis thaliana*	179	5.7	20.7	2e-15	8e-27	3e-04	0.008	3e-30	-
*Brassica napus*	179	9.4	20.7	9e-13	7e-26	4e-05	0.004	1e-28	2e-92
*Citrus clementina*	179	6.2	20.8	3e-12	2e-24	0.069	0.053	5e-27	3e-79
*Fragaria vesca*	179	6.9	21.0	2e-14	5e-29	3e-04	0.018	8e-32	2e-84
*Glycine max*	179	6.1	20.7	4e-16	2e-27	3e-04	0.014	1e-29	3e-84
*Medicago truncatula*	179	5.9	20.9	7e-15	1e-27	2e-05	0.005	6e-28	2e-83
*Populus trichocarpa *× *Populus nigra*	179	6.5	21.0	3e-14	1e-27	0.001	0.011	5e-30	8e-88
*Populus tremula*	179	6.5	21.0	3e-13	3e-27	0.005	0.053	7e-29	2e-86
*Vitis vinifera*	179	7.0	20.9	3e-14	3e-28	2e-05	0.006	8e-30	1e-85
**OPISTHOKONTS**									
**Fungi**									
**Ascomycetes**									
Saccharomycotina									
*Candida albicans*	196	6.1	22.8	3e-20	7e-20	>10	0.05	1e-20	4e-15
*Candida glabrata*	194	5.2	22.0	5e-55	3e-16	>10	>10	1e-12	2e-11
*Clavispora lusitaniae *(*Candida lusitaniae*)	187	6.2	21.4	1e-13	4e-16	>10	0.65	1e-13	5e-14
*Debaryomyces hansenii*	196	6.5	22.5	3e-16	7e-18	>10	>10	9e-15	3e-11
*Eremothecium gossypii *(*Ashbya gossypii*)	180	6.7	20.2	1e-39	8e-19	>10	>10	2e-11	9e-10
*Kluyveromyces lactis*	213	5.4	24.6	1e-35	8e-13	>10	>10	2e-12	7e-08
*Kluyveromyce waltii*	179	5.6	20.7	5e-50	3e-20	>10	0.2	4e-14	3e-15
*Saccharomyces cerevisiae*	202	5.5	23.6	-	2e-17	0.071	>10	2e-19	4e-14
*Saccharomyces bayanus*	203	5.4	23.7	1e-98	4e-20	1.3	>10	1e-20	3e-17
*Saccharomyces castellii*	179	5.7	20.9	2e-59	5e-18	>10	>10	5e-16	2e-15
*Saccharomyces kluyveri*	185	5.3	21.5	9e-51	3e-15	>10	>10	9e-13	5e-12
*Saccharomyces kudriavzevii*	202	5.5	23.4	4e-100	1e-18	>10	>10	3e-21	7e-15
*Saccharomyces mikatae*	202	5.5	23.4	1e-99	3e-19	>10	>10	6e-22	3e-14
*Saccharomyces paradoxus*	202	5.3	23.4	4e-108	2e-18	0.093	>10	2e-21	9e-16
*Yarrowia lipolytica*	169	5.9	19.5	4e-18	2e-24	>10	2e-05	2e-19	1e-17
Schizosaccharomycotina									
*Schizosaccharomyces pombe*	175	7.9	20.8	3e-11	4e-22	>10	7.8	5e-15	1e-15
Pezizomycotina									
*Aspergillus fumigatus*	195	8.1	22.3	5e-12	2e-28	2e-07	>10	6e-24	1e-14
*Aspergillus oryzae*	186	7.5	21.5	8e-10	5e-27	3e-07	>10	4e-24	5e-14
*Botryotinia fuckeliana*	213	8.0	24.6	2e-12	6e-29	2e-07	2e-07	8e-28	1e-19
*Coccidioides immitis*	196	8.0	22.3	>10	1e-27	1e-07	>10	3e-25	6e-16
*Neosartorya fischeri*	191	7.5	21.9	1e-09	1e-26	7e-06	>10	6e-24	2e-13
*Neurospora crassa*	225	8.7	25.0	1e-05	7e-17	>10	>10	9e-18	3e-09
*Phaeosphaeria nodorum*	214	8.4	24.1	1e-14	2e-33	1e-05	5e-05	3e-31	2e-22
*Sclerotinia sclerotiorum*	192	7.5	22.0	9e-17	7e-33	4e-09	6e-07	4e-32	1e-23
*Trichoderma reesei*	222	7.7	24.0	7e-12	6e-32	0.001	3e-05	5e-27	2e-17
*Uncinocarpus reesii*	202	7.6	22.7	2e-12	4e-27	5e-05	0.001	3e-25	5e-16
**Basidiomycetes**									
*Coprinus cinereus (Coprinopsis cinerea okayama)*	234	7.0	26.4	6e-09	4e-16	>10	0.056	1e-16	1e-09
*Phanerochaete chrysosporium*	200	6.9	22.8	3e-12	1e-18	>10	0.090	4e-18	3e-11
*Ustilago maydis*	232	8.1	25.2	2e-17	7e-26	2e-04	3e-04	2e-25	3e-20
**Zygomycete**									
*Rhizopus oryzae*	183	5.0	21.5	7e-16	6.7	0.16	0.002	8e-27	1e-23
**Chytridiomycete**									
*Blastocladiella emersonii*	186	7.4	21.2	1e-13	3e-20	0.044	0.49	6e-17	3e-16
**Metazoa**									
**Echinodermata**									
*Strongylocentrotus purpuratus*	175	6.5	20.6	7e-17	3e-56	1e-10	0.002	2e-39	7e-25
**Nematoda**									
*Caenorhabditis briggsae*	179	6.2	21.1	2e-17	2e-40	9e-10	2e-05	1e-33	2e-22
*Caenorhabditis elegans*	183	6.2	21.5	1e-16	5e-41	2e-10	2e-05	2e-33	3e-22
*Heterodera glycines*	175	6.2	20.7	7e-14	6e-43	5e-10	4e-05	3e-33	2e-24
**Platyhelminthes**									
Trematodes									
*Paragonimus westermani*	178	6.4	20.9	5e-21	4e-45	7e-12	0.001	6e-33	9e-28
*Schistosoma japonicum*	179	7.8	20.7	2e-19	7e-45	6e-09	4e-04	1e-24	9e-26
*Schistosoma mansoni*	180	6.5	20.8	2e-22	1e-46	6e-12	3e-04	2e-26	4e-28
*Schmidtea mediterranea*	176	5.0	20.3	2e-18	3e-51	1e-13	0.017	9e-31	2e-27
**Arthropoda**									
Chelicerata									
*Amblyomma variegatum*	175	6.6	20.8	2e-18	7e-46	1e-09	1e-04	1e-31	3e-25
*Boophilus microplus*	176	5.3	20.3	1e-17	5e-47	6e-11	1e-04	3e-33	7e-25
Hexapoda									
*Aedes aegypti*	173	5.5	19.9	8e-15	5e-34	1e-05	0.061	6e-27	7e-28
*Anopheles gambiae*	173	5.2	19.9	9e-14	2e-37	3e-05	0.89	6e-26	9e-27
*Apis mellifera*	175	5.0	20.5	2e-18	2e-48	3e-10	0.017	2e-28	2e-29
*Acyrthosiphon pisum*	171	5.4	19.6	2e-17	2e-39	6e-05	0.007	2e-26	6e-28
Bombyx mori	175	4.9	20.4	5e-21	6e-45	3e-12	0.008	4e-34	1e-28
*Drosophila melanogaster*	174	5.1	20.6	8e-16	1e-39	4e-11	4e-05	4e-27	1e-24
*Drosophila pseudoobscura*	174	5.2	20.7	6e-19	3e-41	2e-11	0.003	2e-26	2e-25
*Lutzomyia longipalpis*	171	5.2	20.0	2e-16	5e-36	1e-06	3e-04	1e-28	4e-25
**Chordata**									
Urochordata									
*Ciona intestinalis*	176	7.5	20.7	2e-09	6e-38	4e-09	1e-04	1e-21	1e-21
*Molgula tectiformis*	173	5.7	20.6	7e-14	7e-41	2e-05	1e-04	4e-24	5e-22
Vertebrata									
Chondrichthyes									
*Leucoraja erinacea*	174	7.4	20.8	2e-19	7e-75	3e-08	2e-04	1e-38	1e-30
Neopterygii									
*Danio rerio*	174	6.6	20.7	2e-20	5e-77	2e-10	1e-04	2e-42	2e-32
*Fugu rubripes*	174	6.0	20.7	7e-20	2e-77	7e-11	0.001	2e-41	3e-32
*Gasterosteus aculeatus*	174	6.2	20.6	7e-19	2e-76	6e-10	0.006	2e-40	2e-31
*Ictalurus punctatus*	174	6.1	20.7	3e-21	4e-76	1e-10	2e-04	5e-41	2e-32
*Oryzias latipes*	174	6.0	20.8	8e-21	3e-75	1e-11	0.006	1e-41	7e-33
*Oncorhynchus mykiss*	174	5.9	20.7	1e-18	6e-74	7e-11	0.01	2e-41	7e-30
*Platichthys flesus*	173	5.6	20.6	2e-18	3e-77	8e-10	0.003	2e-40	4e-32
*Pimephales promelas*	174	6.6	20.7	2e-20	7e-76	6e-10	5e-04	2e-42	2e-31
*Salmo salar*	174	5.9	20.7	1e-18	6e-74	7e-11	0.01	2e-41	7e-30
*Tetraodon nigroviridis*	174	6.0	20.7	2e-16	5e-70	1e-07	0.001	6e-37	8e-29
Tetrapoda									
Aves									
*Gallus gallus*	174	8.1	20.4	3e-16	1e-70	4e-07	5e-04	1e-29	2e-24
*Taeniopygia guttata*	174	6.2	20.4	2e-16	6e-71	5e-10	0.022	1e-28	4e-23
Amphibia									
*Xenopus laevis*	174	6.6	20.7	6e-22	1e-78	4e-12	2e-04	3e-40	4e-32
*Xenopus tropicalis*	174	6.8	20.7	7e-22	5e-78	7e-12	3e-05	6e-40	6e-32
Mammalia									
*Bos taurus*	176	6.3	20.7	2e-16	1e-90	1e-07	4e-04	3e-31	9e-23
*Canis familiaris*	176	6.3	20.8	2e-16	2e-91	2e-07	3e-04	3e-31	9e-23
*Equus caballus*	176	6.3	20.8	2e-16	2e-91	2e-07	3e-04	3e-31	9e-23
*Homo sapiens*	176	6.3	20.7	9e-17	-	1e-06	4e-04	4e-31	1e-22
*Macaca mulatta*	176	6.3	20.7	9e-17	7e-92	1e-06	4e-04	4e-31	1e-22
*Monodelphis domestica*	176	6.3	20.8	9e-17	2e-89	5e-07	2e-04	8e-32	7e-23
*Mus musculus*	176	6.3	20.7	2e-16	6e-91	3e-07	4e-04	1e-30	5e-23
*Oryctolagus cuniculus*	176	6.3	20.8	1e-16	8e-91	2e-07	4e-04	2e-31	5e-23
*Rattus norvegicus*	176	6.3	20.8	3e-11	4e-78	2e-05	4e-04	2e-23	2e-18
*Sus scrofa*	176	6.3	20.8	2e-16	2e-91	2e-07	3e-04	3e-31	9e-23
**RHIZARIA**									
None identified to date.									

^a^See Additional File 1 for comments on classification of species into major eukaryotic groupings and accession numbers.

^b^Isoelectric point calculated using the assumption that all residues have pKa values equivalent to that of isolated residues, so may not accurately represent the value for the folded protein.

^c^Isotopically averaged molecular weight prediction in kiloDaltons.

^d^The BLASTP E-value (Expect value) measures the statistical significance threshold for protein sequence matches. The smaller the number the better the match. Computer shorthand nomenclature is used to present E-values when values are small. For example, 5e-01 = 0.5 and 5e-04 = 0.0005.

^e^Proteins were compared to *Saccharomyces cerevisiae*, *Homo sapiens*, *Paramecium primaurelia*, *Trypanosoma brucei*, *Dictystelium discoideum*, or *Arabidopsis thaliana *Vps25p by BLASTP. The Vps25 proteins used for comparison were chosen as the representive of fungal, metazoan, chromalveolate, excavate, amoebozoan, or plant species because these sequence are documented on the non-redundant protein database and they are well-known eukaryotic species.

^f^When E-values were greater than 10, details are not provided.

^g^A dash is used when alignments have 100% identity.

The length, predicted pI and molecular masses of the Vps25 orthologs were calculated (Table 1). Vps25 family members are of similar size and vary in predicted mass from 19.5 kDa (*Yarrowia lipolytica*) to 26.4 kDa (*Coprinus cinereus*) with an average of 21.8 kDa. The vast majority of proteins (93 of the 119 full length orthologs) have an acidic pI (Table 1). The pI of all other orthologs is between 7.1 and 8.4, with the striking exceptions of and *Neurospora crassa *and *Brassica napus *with values of 8.7 and 9.4, respectively. It should be noted, however, that predicted pI values do not always correlate with the exact pI values of folded proteins. Acidic pIs can relate to protein function, and acidic amino acids within Vps25 may form electrostatic interactions with the positively-charged amino-terminus of binding partner Vps20, or mediate interaction with an unknown charged nuclear protein(s).

Overall, the putative orthologs of Vps25 identified have a similar length, amino acid sequence, pI, and domain structure, compared to the yeast protein. To obtain a consensus sequence for the protein family, conservation of amino acid sequences between members of the Vps25 family were analysed in detail by alignment using the ClustalX program (Figure 1). Conservation was approximately equal over the entire alignment [Additional File 18]. Only three amino acids were totally conserved in addition to the initiation methionine, although further amino acids are conserved in the amino- and carboxy-terminal regions of non-protist sequences. These analyses indicate these Vps25 proteins form a well-conserved eukaryotic protein family.

All three proteins contributing to the formation of ESCRT-II in yeast (Vps22p, Vps25p, Vps36p) have two repeats of a winged-helix (WH) domain, despite having no recognizable sequence similarity [12,13]. The WH domain has an H1/B1/H2/H3/B2/B3 (H = helix; B = beta) topology [Additional File 18] and is common in transcription factors [31]. We use Greek abbreviations in the first WH domain of Vps25p (α = helix; β = beta) to distinguish it from the second WH domain. The first WH domain of yeast Vps25p has additional beta strands between β1 and α1, known as β1' and β1" [13], and α2 is very short. Furthermore, a conserved arginine residue (Arg83), which forms a salt link with a residue in Vps22p [12,13], is found in a beta hairpin in the unliganded Vps25p structure [20]. Comparison of the amino acid alignment of the Vps25 family with the known secondary structure of yeast Vps25p [Additional File 18] indicates that the dual WH domain structure of Vps25 is likely to be conserved throughout the eukaryota. Regions of sequence predicted to be disordered in the yeast Vps25 structure [12,13,20] are regions in which the largest indels are found [Additional File 18]. Arg83 is highly, but not absolutely conserved, and is a lysine in a number of species. Strikingly, the non-conventional β1' and β1" strands of the first WH domain of Vps25p, are only predicted to be conserved in a subset of fungi, predominantly the Saccharomycotina [Additional File 18]. As their presence is atypical of WH domains, our analysis predicts that the WH domains of most Vps25 proteins will have a more typical WH topology than that of yeast Vps25p.

The amino-terminal region of Vps25p, before the start of the first WH, contains two proline-rich motifs (PRM), PRM-I and PRM-II, which have the consensus PPXY in yeast [12,13]. In one of the molecules of Vps25p in ESCRT-II these mediate interactions with Vps22p, and in the other molecule they interact with Vps36p. A phenylalanine (Phe10), immediately prior to PRM-II, lines a hydrophobic pocket of either Vps22p or Vps36p [12,13] and is also important for dimerization of unliganded Vps25p [20]. The second proline of PRM-1, and both prolines of PRM-II, make hydrophobic interactions with Vps22p and Vps36p, and the tyrosine residues are important for this interaction [12,13]. Our analysis of the PRM region of the Vps25 family shows the most striking difference is the lack of conservation of PRM-I in chromalveolate, diplomonad and kinetoplast sequences (corresponding to the first 3/10 helix in Figure 1), while *Paramecium primaurelia *lacks both PRMs. While it is the second proline in PRM-1 that is known to interact with other ESCRT-II components, we unexpectedly find the first proline in PRM-1 is more highly conserved than the second proline. In metazoan species the amino acid corresponding to the second proline of yeast Vps25p PRM-1 is invariably substituted with a tryptophan. The second proline of PRM-1 is not even well conserved among the fungi, and is present in only 12 of the 33 fungal regions examined. The residue corresponding to Phe10, located between PRM-I and PRM-II of yeast Vps25p, is highly conserved, and is retained in apicomplexan, diplomonad, and parabasalid sequences. It is conservatively substituted with tyrosine in all Plantae, and with another hydrophobic residue in kinetoplast species. It is unexpectedly absent from two basidiomycete species, *Coprinus cinereus *and *Phanerochete chrysosporium*, and *Dictyostelium discoideum*, which also lack the conserved tyrosine of the first PRM. A few other fungal species also lack the tyrosine of the first PRM.

In contrast to PRM-I, PRM-II is highly conserved across the eukaryota. The most striking difference is that rather that the PPXY consensus identified in yeast, we show the consensus for PRM-II is PPXF. This is because the final tyrosine (Tyr14) of yeast Vps25p is only conserved in 17 of the 119 family members aligned. In 97 of the 119 Vps25 proteins, this final PRM-II residue is a phenylalanine. In addition, rather than amino acid 13 being an arbitrary amino acid, we show this position is also conserved across the Vps25 family, as is typically a phenylalanine, but a tyrosine in plants. PRM-2 can therefore be more accurately defined as P-P-[FY]-[FY]. Finally, the four amino acids after PRM-II are also highly conserved. These are: an almost absolutely conserved threonine residue and highly conserved leucine residue (Thr15, Leu16 using yeast numbering), followed by the first absolutely conserved amino acid of the Vps25p family, a glutamine (Gln17), and finally there is a further highly conserved proline residue (Figure 1). Overall a consensus for the expanded PRM-I and PRM-II region, conserved in most Vps25 family members is: P-[PW]-X-[YF]-X-[FYL]-P-P-[FYL]-[FY]-T-L-Q-P.

In addition to the absolutely conserved glutamine residue in the proline-rich amino-terminal domain, mentioned above, there are two further totally conserved residues across the Vps25 family. These are a tryptophan in the first alpha-helix of WH-1, and a threonine at the end of the first beta-strand in WH-2 [Additional File 18]. The significance of these residues remains to be determined. An additional, potentially exciting, finding from our amino acid alignment of Vps25 family members is the presence of a conserved lysine residue near the carboxy-terminus (Figure 1). In the yeast Vps25 structure [20], this lies at the end of a beta-strand (Figure 1). Given the role of ESCRT complexes in sorting ubiquitinated cargo, and the proposed role of ubiquitination in regulating function of other ESCRT proteins [6-9], this suggests that Vps25 proteins may have the ability to be ubiquitinated. Ciliate species were the only species lacking a lysine residue in the carboxy-terminal region [Additional File 18]. *Entamoeba histolytica *and the fungus *Coprinus cinereus *lack a lysine in the conserved consensus, but rather have a lysine as the final amino acid. The highly conserved lysine is typically found in a G-V-K-F motif, with 98% of lysine residues found in a [GA]-[VIL]-K-[FVI] consensus motif. Although a consensus for ubiquitination has not been determined [32], it is of interest that this motif is found in a number of proteins involved in ubiquitin cascades. For example: human RING-finger protein-31 (Q96EP0), two fungal ubiquitin-conjugating E2 enzymes (Q4WLA7 and Q96UP5) and human deubiquitinating enzymes -33 (Q8TEY7) and -7 (Q93009).

To assess the evolutionary relatedness of the Vps25 proteins, a phylogenetic tree, based on the alignment of the amino acid sequences of the Vps25 orthologs [Additional File 5] was generated [Additional File 19]. A distance-based tree was constructed under minimum evolution criteria [34]. Bootstrap support for branching of many of the more disparate sequences was low. This was attributed to the lack of conservation in key areas, such as PRM-1 and Phe-10 [Additional File 18]. At present, we cannot rule out that at least some of these differences are due to uncharacterized sequence and gene annotation errors. Indeed, we have corrected errors in both protein and nucleotide sequences when we have found them, including those in five of the Vps25 orthologs on the Pfam database [see Additional Files 1, 3 and 12]. This makes an error rate, even on the highly-curated protein databases, of around one in every six sequences. Additional Figure 15 [Additional File 18] illustrates our current knowledge of the evolutionary relationship between Vps25 proteins, and shows homologs from protists, fungi, plants, and metazoans clustering with each other.

### Intron positions of VPS25 genes

To examine the evolutionary conservation of the structure of *VPS25 *genes, exon-intron mapping was carried out when genomic sequence was available via the genomic databases. In many microbial eukaryotes the *VPS25 *gene was encoded by a single exon: *Giardia lamblia, Trichomonas vaginalis*, *Leishmania *sp., *Trypanosoma *sp., all the Saccharomycotina, some (*Magnaporthe grisea*, *Neruospora crassa*, and *Phaeosphaeria nodorum*) but not all Pezizomycotina, and one basidiomycete (*Ustilago maydis*). By contrast *VPS25 *was found encoded by more than one exon in all metazoan and plant genomes, where data were available. Interestingly, introns were also found in the *VPS25 *gene of some microbial eukaryotes: apicomplexans *Theileria annulata *and *T. parva*; ciliate *Paramecium primaurelia*; ameobozoa *Dictyostelium discoideum *and *Entamoeba histolytica*; and many fungi including *Schizosaccharomyces pombe*, some Pezizomycotina (*Aspergillus fumigatus*, *A. oryzae*, *Botryotinia fuckeliana*, *Coccidioides immitis*, *Gibberalla zeae*, *Neosartorya fischeri*, *Sclerotinia sclerotiorum*, *Trichoderma reesei*, and *Uncinocarpus reesii*), some basidiomycetes (*Coprinus cinereus *and *Phanerochaete chrysosporium*), and the zygomycete *Rhizopus oryzae*.

The location and phases of intron positions were determined within the above genes (Table 2) and marked on a multiple sequence alignment [Additional File 6]. Six intron positions have been named 0–V, with intron I being a phase 2 intron in all species except for the apicomplexans *T. annulata *and *T. parva*, where intron slippage is proposed to have occurred, giving rise to a phase 0 intron (see Discussion). Intron 0 was exclusive to ciliate *P. primaurelia*. By constrast, introns I–V were found in plants and to varying extents in metazoan species. A common evolutionary origin of *VPS25 *genes was substantiated by conservation of introns I–V.

**Table 2:** Location of introns within Vps25 sequences.

Species^a^	Intron number (and phase)					
	
	0 (2)	I (2)	II (1)	III (1)	IV (0)	V (1)
**CHROMALVEOLATES**						
*Theileria annulata*	x	√^b^	x	x	x	x
*Theileria parva*	x	√^b^	x	x	x	x
*Paramecium primaurelia*	√	x	x	x	x	√
**AMOEBOZOA**						
*Entamoeba histolytica*	x	√	√	x	x	x
*Dictyostelium discoideum*	x	x	√	x	x	x
**PLANTAE**						
*Oryza sativa*	x	√	√	√	√	√
*Arabidopsis thaliana*	x	√	√	√	√	√
**OPISTHOKONTS**						
**Fungi**						
**Ascomycetes**						
*Aspergillus fumigatus*	x	x	√	x	√	√
*Aspergillus oryzae*	x	x	√	x	√	√
*Botryotinia fuckeliana*	x	x	√	x	x	√
*Coccidioides immitis*	x	x	x	x	√	x
*Cryptococcus neoformans*	x	√	√	x	√	√^c^
*Gibberella zeae*	x	x	√	x	x	√
*Neosartorya fischeri*	x	x	√	x	√	√
*Schizosaccharomyces pombe*	x	x	x	x	√	x
*Sclerotinia scleroriorum*	x	x	√	x	x	√
*Trichoderma reesei*	x	x	√	x	x	√
*Uncinocarpus reesii*	x	x	x	x	√	x
**Basidiomycetes**						
*Coprinus cinereus*	x	√	x	x	√	x
*Phanerochaete chrysosporium*	x	√	x	x	√	x
**Zygomycete**						
*Rhizopus oryzae*	x	√	√	√	√	√
**Metazoa**						
**Echinodermata**						
*Strongylocentrotus purpuratus*	x	√	√	√	√	√
**Nematoda**						
*Caenorhabditis briggsae*	x	√	√	√	x	√
*Caenorhabditis elegans*	x	√	√	√	x	√
**Arthropoda**						
*Anopheles gambiae*	x	√	x	x	x	x
*Apis mellifera*	x	√	√	x	x	√
*Drosophila melanogaster*	x	√	x	x	x	x
*Drosphila pseudoobscura*	x	√	x	x	x	x
**Chordata**						
*Fugu rubripes*	x	√	√	√	√	√
*Tetraodon nigroviridis*	x	√	√	√	√	√
*Bos taurus*	x	√	√	√	√	√
*Canis familiaris*	x	√	√	√	√	√
*Homo sapiens*	x	√	√	√	√	√
*Monodelphis domestica*	x	√	√	√	√	√
*Mus musculus*	x	√	√	√	√	√
*Rattus norvegicus*	x	√	√	√	√	√

^a^Species known to lack introns in *VPS25 *genes are listed in Additional File 1.

^b^Intron I of the apicomplexan species examined appears to have undergone 'slippage' and is now in phase 0, and is seven nucleotides downstream to all other intron I positions.

^c^See comments in Additional File 2.

### Chromosomal localization of VPS25 genes

Human *VPS25 *localizes to chromosome 17, map position 17q21.31, mouse *Vps25 *localizes to chromosome 11, map position 11D (60.0 cM), rat *Vps25 *to chromosome 10, map position 10q32.1, and *Caenorhabditis elegans *Vps25 to chromosome I (map position +14.60 cM). The known chromosomal allocations of all *VPS25 *orthologs are listed in Additional File 1. Further analysis of the chromosomal context of the spliced *Vps25 *genes revealed that the human, rat, mouse, dog and cow are found in syntenous regions [Additional Files 7, 8, 9]. These five *Vps25 *genes are found downstream to the gene encoding Ramp2 (receptor [calcitonin] activity modifying proteins 2) and upstream to the *Prkwnk4 *gene encoding the Wnk4 kinase (With no lysine [K] 4), all in head-to-tail positions on the positive strand. The partial sequence of the *VPS25 *ortholog in *Pan troglodytes *on chromosome 17 is also in a region syntenic to that of human *VPS25 *[Additional File 7].

While only part of the genomic sequence corresponding to zebrafish and chicken *Vps25 *mRNAs are currently known, these sequences localize to chromosomes 1 and 27, respectively [Additional File 1]. Our analysis of the surrounding genes [35] indicates synteny between chicken, but not zebrafish *Vps25 *regions, and mammalian *Vps25 *regions (data not shown). No synteny was found between the *Vps25 *genomic regions of *Anopheles gambiae*, *Drosophila melanogaster*, *C. elegans *[Additional File 10] or plants [Additional File 11], and the mammalian *Vps25 *regions.

Searches of the Pfam and NR databases reveal some *Vps25 *sequences that were suggestive of longer splice variants of Vps25, some involving RNA splicing of *VPS25 *with portions of the upstream or downstream genes, and others involving alternative start codons [Additional File 12]. We found no evidence for the expression of longer splice variants of human or mouse *Vps25 *[Additional File 13]. Furthermore, these variants are not evolutionarily conserved, together suggesting these are not biologically relevant.

### VPS25 pseudogenes and paralogs

In addition to the excavate species, *Trichomonas vaginalis *which has two *VPS25 *paralogs [Additional File 1], we identified six *VPS25 *pseudogenes [Additional Files 14 and 15]. Ciliate *Paramecium primaurelia *P-protein, which we identify as a Vps25 ortholog, has previously been described as having a non-processed pseudogene in a >15 kb duplicated genomic region [36]. We found no further non-processed pseudogenes.

Processed pseudogenes were found exclusively in mammalian genomes [Additional File 14], however, no pseudogenes were found in the completed *Rattus norvegicus *or *Mus musculus *genomes.

Of the mammalian species, single processed pseudogenes, lacking introns, were found in the genomes of *Echinops telfairi*, *Homo sapiens *(chromosome map position 1p12), and *Monodelphis domestica *[Additional File 14]. Two processed pseudogenes were found in the *Pan trogdolytes *genome (chromosome 1 and chromosome 2A). The nucleotide sequence of mammalian *VPS25 *pseudogenes was compared to that of the orthologous *VPS25 *sequence [Additional file 16] and this enabled us to confirm the pseudogenes were Type 1 pseudogenes, rather than paralogs, by a number of further criteria [37]. Confirmation of Type 1 pseudogenes was due to: (i) each sharing a high sequence similarity with the corresponding *VPS25 *ortholog, with BLAST E-value of less than 1e-10 (when this could be calculated), considered typical for a pseudogene; (ii) sequence alignment with the *VPS25 *ortholog not containing gaps longer than 60 bp; (iii) the alignment covers >70% of the coding sequence; and (iv) the sequence contains frame disruptions. The mammalian *VPS25 *pseudogenes are retropseudogenes, as they do not contain any introns, indicating they were inserted into the genome by the retrotransposition of the mRNA of the *VPS25 *ortholog [38]. This also means that they are released from selective pressure, which results in the characteristic mutations. For example, the single frameshift error in the *H. sapiens *pseudogene is located where intron I is found in the *VPS25 *ortholog [Additional Files 6 and 16].

In *P. troglodytes *pseudogene-1 (PS-1), frameshift errors are localized to where intron I and intron V are found in the *VPS25 *ortholog [Additional File 16]. The chromosomal region of *P. troglodytes *ps-1 is similar to the region of chromosome 1 where the *H. sapiens *pseudogene is found, as PS-1 is located adjacent to the alpha-1,2-mannosidase gene, which is next to immune costimulatory protein B7–H4 [Additional File 17]. By contrast, *P. troglodytes *PS-2, is localized to a region of chromosome 2A with no similarity to the location of the human pseudogene. While PS-2 lacks all introns, indicating retrotransposition, it contains no frameshift or stop mutations, suggesting a more recent evolution than that of PS-1.

The parabasalid *Trichomonas vaginalis *is unique as it has two *VPS25 *paralogs [Additional File 1]. Both are full-length homologs with no frame-shift mutations or in-frame stop codons [Additional File 18]. The paralogs differ by 10 amino acids, which are found at the amino-terminus of the longer *T. vaginalis*-1 protein. The protein products are 72% similar (30% identical) and have both predicted secondary structure (data not shown), and motif, conservation with Vps25 from other species [Additional File 18]. For example, the carboxy-terminal lysine and two amino-terminal PRMs are highly conserved in both paralogs. The phenylalanine immediately preceding PRM-II in yeast Vps25p is important for Vps25 dimerization and partner binding, and is conserved in *T. vaginalis *paralog-2. However, in paralog-1 this residue is a tyrosine. This change probably does not negate function, as a tyrosine substitution is typically found in plant Vps25 proteins [Additional File 18]. Likewise, non-conservative substitutions were found in a few highly conserved residues outside PRM-II, typically in paralog-1. However, each was found to have a comparable substitution(s) in Vps25 from another specie(s) [Additional File 18]. We conclude on the basis of sequence conservation that both paralogs are likely to be functional. Nonetheless, we do not know whether both genes are translated and therefore cannot rule out mutations in non-coding sequences, which would indicate one of these is a pseudogene.

### Expression of mammalian VPS25

**Figure 2:**
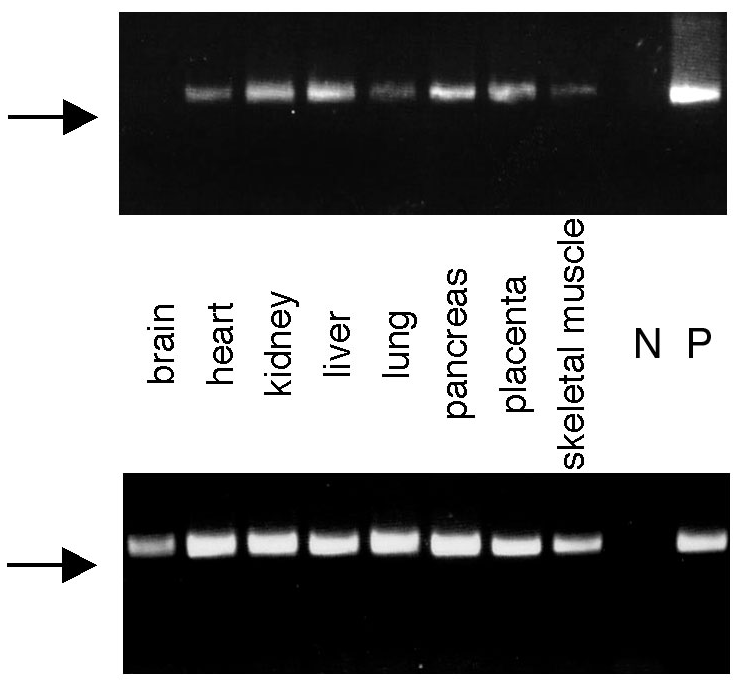
**Multiple tissue PCR analysis of the expression of human *VPS25***. A panel of normalised, first strand cDNAs, ready for quantitative PCR, was screened using primers designed to specifically amplify 547 bp portion of *VPS25 *mRNA. Expression of *VPS25 *after 30 (upper panel) or 38 cycles (lower panel) was examined. After 38 cycles saturation of most reactions had occurred, but is provided to clearly demonstrate expression in brain. A PCR product of approximately 550 bp was detected in all human tissues examined. The arrow indicates 500 bp marker. N = negative control (no DNA). P = positive control (human *VPS25 *cDNA).

To determine the levels of expression of human *VPS25 *in different cell types, we screened a human multiple tissue cDNA panel. A strongly positive PCR product was detected after 30 cycles in cDNA derived from kidney, liver, pancreas, and placenta, with weaker products resulting from heart and skeletal muscle (Figure 2). After 38 cycles, expression of *HsVPS25 *mRNA was also detected in brain and lung. No smaller splice variants were detected. These results suggest that *VPS25 *is a universally expressed gene with a fundamental cellular role.

## Discussion

Vps25p is a component of yeast endosomal sorting complex, ESCRT-II, essential for sorting ubiquitinated cargoes into endosomes [9,12,13]. When we initiated this study Pfam curated only eight Vps25 orthologs, while version 19.0 now contains 28 correctly annotated orthologs. We identified a total of 119 full-length orthologs, and a further 48 partial sequences, from a wide range of eukaryotic species. Vps25 orthologs are common to chromalveolates, excavates, amoebozoa, red and green algae, land plants, fungi, nematodes, echinoderms, platyhelminthes, arthropods, urochordata, fish, birds, amphibians, and mammals. While further curation and sequencing of gaps within *Plasmodium *genomes may result in the identification of Vps25 orthologs, the gene was strikingly absent from the complete microsporidian genome of *Encephalitozoon cuniculi*. Microsporidian genomes are strikingly compact, and this is due to a reduction in both gene size and number [38]. Homologs of genes involved in protein trafficking are the plasma membrane, endosomes, endoplasmic reticulum and Golgi apparatus have been identified in *E. cuniculi*, but these appear restricted to those involved in basic functions. A lack of a Vps25 ortholog in this obligate intracellular parasite is therefore unsurprising, and is predicted to be one of the genes lost during reductive microsporidian evolution.

The human gene product is a predicted 178 amino acid protein with a pI of 6 and the human *VPS25 *gene localized to chromosome position 17q21.31, a region syntenous to positions in chimpanzee, mouse, rat, dog, and cow. In addition to synteny between mammalian *VPS25 *gene regions, chicken *VPS25 *also appeared in a region syntenous to mammalian *VPS25*, however, that of zebrafish *Vps25 *did not. Extensive conservation of synteny has been found previously between mammals and chicken [40]. Many regions of synteny are also known to be conserved between the zebrafish and human genomes [41], however our data suggests that this does not include the *VPS25 *locus.

The Vps25 family members identified have a similar length, amino acid sequence, pI, and overall domain structure, supportive of the hypothesis these proteins are orthologous. Comparison of the amino acid alignment of the Vps25 family with the known secondary structure of yeast Vps25p, indicates that the dual WH domain structure is likely to be conserved throughout the eukaryota. A common evolutionary origin of the Vps25 proteins was further confirmed by conservation of many of the intron positions within *VPS25 *genes. Intron positions were interspersed between the secondary structure domains of the tandem WH domains. Introns I–III localized to the first WH domain; intron I after the two 3/10 helices containing the PRMs, intron II after the first alpha helix, and intron III after the third alpha helix. Intron IV and V were localized to the second WH domain, with intron IV near the end of the first helix, and intron V between helix-2 and helix-3.

Our general knowledge of the patterns of intron gain and loss is still limited [42,43], but two main theories about the evolutionary origin of introns have been proposed. The first, the Introns Early (IE) theory, proposes that introns were present in a common ancestor of prokaryotes and eukaryotes, while the Introns Late (IL) theory proposes that introns arose in the first eukaryotes. One of the earliest pieces of evidence for the IE theory was the finding that several intron positions of the triose phosphate isomerase gene were conserved between vertebrates and plants [44,45], indicating introns pre-dated the plant-animal divergence. More recently, a large-scale comparison of intron positions among animal, plant, and fungal genes by Fedorov and coworkers indicated that around 10% of all animal introns have matches in plant gene homologs and around 7% of animal introns match fungal introns [46], providing further evidence for ancestral introns pre-dating the animal-plant-fungal split and contradicting any theory based on random intron insertion. Fedorov and coworkers also found that ancestral introns (those common to animals, plants, and fungi) were not restricted to phase zero [46].

Our analyses of intron positions in *VPS25 *orthologs have identified five intron positions (introns I–V) common to animals, plants, and fungi. Parallel insertion into 'preferred' sequence sites [46], as expected by the IL theory, would be highly unlikely to explain this finding. Intron phases in *VPS25 *genes were not restricted to phase 0, as intron I was phase 2, and introns II, II, and V in phase 1. Introns I and II were also conserved in two chromalveolate (*Theileria annulata *and *T. parva*) and amoebozoan species (*Entamoeba histolytica*, and *Dicytostelium discoideum*), while intron V was conserved in the chromalveolate, *Paramecium primaurelia*. Conservation of these introns indicates that they were present in an ancient *VPS25 *gene existing before divergence of chromalveolates, amoebozoa, plants, and animals, an event occurring around 1,000,000,000 years ago [47-49]. Our data therefore provide strong support for the IE theory of intron evolution.

A list of animal and plant gene matches with the highest number of common intron positions has been published, with two genes identified with 11 common introns and a further ten genes with 6–9 introns in common [46]. Fifteen genes had five introns in common. All these genes, with high numbers of common intron positions, all had further additional introns unique to either the plant or animal gene. We can now add *VPS25 *to the list of genes with high levels of commmon plant/animal introns, and show it is the first such plant/animal gene match to exclusively have only common intron positions.

Although we found introns I–V conserved in fungi, no fungal gene had all five introns present, and many fungal *VPS25 *genes lack introns completely. Nematodes lack intron IV, and arthropods (hexapoda) lack introns III and IV. Of the hexapoda, bee (Order Hymenoptera) *VPS25 *retains intron II, while it is absent from fly (Order Diptera) *VPS25 *genes, suggesting loss of intron II after fly divergence. *VPS25 *from both amoebozoan species examined (*Entamoeba histolytica *and *Dictyostelium discoideum*) was characterized by a lack of introns II–V (suggesting loss of these three introns before divergence), while *D. discoideum *also lacked intron I, suggesting loss after divergence from *E. histolytica*. Two mechanisms have been proposed for intron loss: reverse transcription and genomic deletion [50]. We propose *VPS25 *genes lacking any of introns I–V is because of intron loss over evolutionary time. For example, loss of intron II may have occurred after the divergence of Diptera from Hymenoptera, as it is present in bee *VPS25*, but not in the two fly species (*Drosophila melanogaster *and *D. pseudoobscura*) characterized. Our findings therefore support the hypothesis that intronless and near-intronless eukaryotic genes are due to large-scale intron loss.

Intron I of *Theileria *species appears to have undergone intron slippage, when compared to the position in amoebozoan, fungal, or metazoan genes, as the intron site falls within the proposed 12 bp limit for intron displacement [51]. The intron slippage event has also led to a phase change. True intron sliding is rare [52-54], but this is not the first example of a phase change by a sliding event being accommodated in a functional gene [55]. Several mechanisms have been proposed for intron sliding [56,57]. However, we cannot rule out an alternative scenario where this altered intron position was caused by an intron loss followed by a gain.

A single convincing instance of intron gain was found in the *VPS25 *gene of *Paramecium primaurelia*. *P. primaurelia *retains ancient intron V, but compared to all other *VPS25 *genes, has a novel intron, designated intron 0, and in phase 2. This is the same phase as intron I of other *VPS25 *family genes, but is too far from the intron I site to be considered a slipped intron. At present, we lack information to determine whether this novel intron is conserved in *VPS25 *genes of other ciliates. To date, our studies of the *VPS25 *family have therefore revealed five ancient introns, evidence for intron loss, an example of intron slippage, and a single example of intron gain. Conservation of intron positions also is supportive that the proteins we have characterized are orthologous.

The *Trichomonas vaginalis *genome was the only species with evidence for Vps25 paralogs, and it had two copies of the gene. Neither gene contained mutations in the coding region affecting translation, although we cannot rule out non-coding mutations. Both genes lacked introns, but as processed pseudogenes have only been observed in metazoans and flowering plants [58], we assume the paralogous gene was formed by duplication rather than reverse transcription. *T. vaginalis *encodes a large number of Rab genes, many of these represent novel subfamilies [59]. This indicates trichomonads have a highly complex endomembrane system and provide a precedent for functional retention of additional copies of genes involved in protein trafficking in this species.

While a non-processed pseudogene has previously been reported for the *Paramecium primaurelia *P-protein [36], which we identify here as a Vps25 ortholog, processed pseudogenes of *VPS25 *have not been reported previously. We identify processed pseudogenes in four mammalian species: a single pseudogene in *Homo sapiens*, *Echinops telfairi*, *Monodelphis domestica*, and two in the *Pan troglodytes *genome, PS-1 and PS-2. Our data supports the hypothesis that the second *P. troglodytes *pseudogene has only recently evolved. First, while *P. troglodytes *ps-1 is inserted into a similar chromosomal context to the human pseudogene, PS-2 is found on a different chromosome in a region with no similarity to that of the human pseudogene. Secondly, in contrast to PS-1, PS-2 contains no frameshift or stop mutations, suggesting less elapsed evolutionary time. The lack of a corresponding PS-2 in the human genome suggests either its loss or its independent evolution after divergence of humans and chimpanzees around 5 million years ago. Sequencing of further primate genomes will help determine the origin or loss of *P. troglodytes *PS-2. The lack of a *Vps25 *pseudogene in rodent genomes may indicate that the deletion of the pseudogene after divergence from primates around 75 million years ago. There is evidence that pseudogene deletions occur more rapidly in mouse, as compared to human [60] and approximately 60% of retropseudogenes in human and mouse genes are lineage specific [37]. Ongoing mapping of the *E. telfairi *and *M. domestica *genomes may assist us in elucidating when the mammalian pseudogenes arose.

Yeast Vps25p, as well as the other ESCRT-II components, share a common tertiary structure, of tandem WH domains [12,13]. WH domains have a H1/B1/H2/H3/B2/B3 topology, where the three helices of each of the WH domains assemble beneath a small beta sheet formed by the three antiparallel beta strands. Comparison of the amino acid alignment of all Vps25 family members with the known secondary structure of yeast Vps25p reveals that all Vps25 orthologs are predicted to have a tandem WH structure, suggesting a common structure and function. Yeast Vps25 has additional beta strands between the B1 and H1 of the first WH domain. These additional strands are not conserved, indicating a more conventional WH-1 domain for most Vps25 proteins.

Our comprehensive sequence alignment of the Vps25 protein family revealed three absolutely conserved amino acids including one glutamine and a tryptophan in the amino-terminal portion. The conserved glutamine residue (glutamine-17 of yeast Vps25p) was shown experimentally to be important for interaction of Vps25p with either Vps22p or Vps36p [12,13]. By contrast, the role of tryptophan-30 remains to be determined. Yeast arginine-83, also necessary for Vps25p to bind Vps22p or Vps36p [12], and was found to be highly conserved. Also within the amino-terminal region of Vps25 are two PPXY motifs (motif I and II) [13]. These motifs are involved in dimerization of unliganded Vps25p and interact with Vps22p and Vps36p [13,20]. Motif I was absent in chromalveolate, diplomonad and kinetoplast sequences, and both motifs were absent from *Paramecium primaurelia *Vps25. The significance of these absences is not known, but we are currently examining the conservation of other ESCRT-II components in these species. Of those species with both proline-rich motifs, the general consensus can now be modified to: P-[WP]-X-[YF] for motif I and P-P-[FYL]-[FY] for motif II.

Basic residues potentially implicated in nucleic acid recognition are found at positions arginine-23 and lysine-99 in the amino-terminal half of yeast Vps25p [20]. We find these are only moderately conserved. Our analysis also reveals two further moderately conserved basic residues in the carboxy-terminal half of Vps25, arginine-126 and arginine-183, which are exposed on the surface of yeast Vps25p [20]. Any role of these residues for a nuclear role of Vps25 will need to be examined experimentally in the future. Residues in the carboxy-terminal half of yeast Vps25p, the region known to be involved in interaction with ESCRT-III component Vps20p [61], have yet to be examined by mutagenesis or crystallography. Wernimont and Weissenhorn [20] suggest that tyrosine-152, glutamate-153 (at the start of WH2 helix 2) and glutamate-170 (at the start of WH2 helix 3) may be involved in yeast Vps20p interaction, due to their accessibility. Our analysis shows that glutamate-170 is not well conserved and is only present in Vps25 from 38 of 119 species. It is surprisingly well-conserved in higher animals, but not in other fungal species, while in plants it is typically an arginine residue. Glutamate-153 is more highly conserved and is found in Vps25 of 110 of the 119 species examined, and of those 9 species without glutamate-153, 4 have conservative substitutions of aspartate residues. Those species without highly conserved glutamate-153 were: the three alveolate species (*T. annulata, T. parva, P. primaurelia*), the entamoeba *E*. *histolytica*, and one plant species (*Brassica napus*). Tyrosine-152, like glutamate-170 was not conserved in plants, where it was typically a glutamate residue. In non-plant species, however, tyrosine-152 was relatively well conserved, being found in 77 of 94 (over 80%) Vps25 sequences. These analyses suggest that plant ESCRT-II interactions, particularly, may differ from those of yeast.

We further found a highly conserved lysine residue near the carboxy-terminal end of Vps25 proteins in short beta-strand [20]. In the unliganded yeast Vps25p structure, the conserved lysine is surface exposed [20], suggesting a functional rather than structural role. It is found within a conserved G-V-K-F consensus sequence. We suggest, on the basis of: (i) the previously identified role of ubiquitination in the ESCRT cascade; (ii) the surface position of the amino acid; (iii) high conservation of the residue; and (iv) the presence of a similar motif in known ubiquitin-binding proteins, that Vps25 proteins may be ubiquitinated on lysine-200 (yeast Vps25p numbering), and we are currently examining experimentally.

*VPS25 *was found expressed in a broad number of human tissues with no indication of the use of alternative terminators or alternative splicing. Expression of mammalian *VPS25 *in a wide range of tissues suggests a generic, but important cellular function(s). Mammalian ESCRT-II was originally identified as a having a nuclear role [14,15], whereas that of yeast has an established endosomal function [9]. As we have provided evidence that Vps25 family members are orthologs, related by speciation events, family members are likely to have conserved function. Bu contrast, if multiple human homologs of yeast Vps25 existed, as occurs for other ESCRT components [24], functionality may differ for each protein. Our data therefore imply that mammalian ESCRT-II will additionally have a function in endosomal sorting, analogous to that of yeast. Indeed, two recent papers have found that, under certain circumstances at least, mammalian Vps25 can be found localizing to endosomal structures [62,63]. Conversely, our data imply that yeast Vps25p may also have a nuclear role, in addition to its well-characterised function in endocytic sorting. Supporting this hypothesis, another yeast ESCRT-II component, Vps36p, is thought to have a nuclear role in regulating mRNA synthesis and/or stability [15,22]. Interestingly, many other class E Vps protein homologs in mammals have also been found in the nucleus as well as the cytosol and endosomal membranes, including Tsg101, Chmp1 (human Vps46a) and Chmp3 (rat Vps24) [64-67]. Further work on the functions of ESCRT-II proteins should provide valuable insights into the relationship between the transcriptional regulatory and endosomal trafficking pathways.

## Conclusion

We have identified a large number of Vps25 family members but suggest that gene loss has occurred in the microsporidian genome. We show that mammalian Vps25 is orthologous to yeast Vps25p and is therefore expected to have an endosomal as well as nuclear function. We detected widespread tissue expression of human *VPS25*, indicating Vps25 performs pivotal role(s) within eukaryotic cells. Synteny was found between *VPS25 *chromosomal regions in mammals and chicken, but not mammals and fish. The *VPS25 *gene family possesses many evolutionarily conserved intron sites, supportive of an ancestral origin. Evidence of intron loss, intron slippage, and intron gain were also identified. Retropseudogenes were found in four mammalian species (human, chimpanzee, hedgehog, and opossum), but were absent from rodent genomes. A recently evolved retropseudogene was identified, which was exclusive to the chimpanzee genome. We have analyzed conserved residues within Vps25, which has led us to redefine the consensus of the amino-terminal proline-rich motifs, and to hypothesize that Vps25 is ubiquitinated. Comparison of evolutionarily conserved residues over such a wide number of protein family members provides a valuable resource for studying Vps25 function in a large number of species.

## Methods

### Detection, analysis, and chromosome mapping of Vps25 family proteins

The Vps25p amino acid sequence of the yeast *Saccharomyces cerevisiae *amino acid was used in BLAST searches, using the BLASTP [68] and PSI-BLAST [69] programs, which identified homologous sequences from the NR protein database. Searches were also made of the EST and genomic databases using the TBLASTN program. BLAST searches, including genomic searches, were carried out at the National Center for Biotechnology Information (NCBI) [35]. Alternatively, specific searches were carried out at the PEDANT web site [70], the JGI database at the University of California [71], the Sanger Gene Database at the Wellcome Trust Sanger Centre [72], the *Cyanidioschyzon merolae *genome project and Silkworm genome databases at the University of Tokyo [73,74], the *Fugu *Genome Project at the Institute of Molecular and Cellular Biology, Singapore [75], the Protist EST Program at the University of Montreal [76], and the *Porphyra yezoensis *EST database at the Kazuza DNA Research Institute [77]. The Pfam [27] database (release 19.0) was also searched using the Vps25p amino acid sequence [78]. Diagrams of genomic context were modified from data available via the NCBI mapviewer, and ideograms were obtained from the same website [35].

### Alignments and protein analysis

Nucleotide sequences were aligned using Multalin (version 5.4.1) [79] at the Network Protein Sequence Analysis [80] web site [81]. EST sequences were translated using the Translate Tool at ExPASy [82]. The ExPASy Compute pI/Mw Tool [83] was used to calculate theoretical isoelectric points and molecular weights. Protein sequence alignments were generated with ClustalX (1.8) [84]. Aligned sequences were edited manually where alignment was poor, by comparison with BLASTP- and PSI-BLAST-derived data. Boxshade version 3.21 was used to format amino acid alignments [85].

### Phylogeny

Aligned amino acid sequences [Additional File 18] were used to construct phylogenetic trees. Gaps were removed from the sequence alignment, as well as amino acids flanking longer gaps, where the alignment was uncertain [Additional Data File 5]. Distance tree estimates were then generated under the minimum evolution critereon [34,86] using MEGA 3.1 software [87]. The close-neighbour-interchange heuristic search algorithms for finding the optimal tree under the minimum evolution criteria were used, where a temporary neighbour-joining tree [88] was generated for comparison [89], using dynamic criteria [90]. The bootstrap method [91] was used as a statistical test of the inferred phylogeny, and a majority-rule consensus unrooted tree generated. Branch lengths are not presented, as each branch with less than 50% statistical significance was collapsed to provide emphasis to those reliable portions of the tree. Use of maximum likelihood or Bayesian methods did not result in further clarification of branching [92-95].

### Tissue expression

Human or mouse multiple tissue cDNA panels (Clontech) were probed using oligonucleotide primers, according to the manufacturer's instructions. PCR products were analysed before saturation by taking samples after various cycle times, to ensure that the relative abundance of target in each tissue could be directly compared. The multiple tissue panels had been normalised using the mRNA expression levels of several housekeeping genes. PCR products were run in parallel with DNA size markers (MBI Fermentas) on 2% (w/v) agarose gels. The primers used to amplify the *HsVPS25 *gene were located: (i) just after the initiation codon, and (ii) in the 3'-untranslated region. The sequences of the primers were: (i) 5'-gtttcgagtggccgtggcagtatcgcttcc-3' and (ii) 5'-ggaggtaagaagtaaagggagacaggtcc-3'. These primers would detect *H. sapiens VPS25 *and produce a product of 547 bp. The primers would not detect the predicted human *VPS25 *pseudogene.

## Authors' contributions

NEB initiated the project and RS and NEB were involved in the design phases and in carrying out gene analyses and interpreting results. RS designed, carried out and interpreted PCR experiments, contributed to writing the paper, and read and approved the final manuscript.

## Supplementary Material

Additional File 1 Additional Table 1: Taxa, accession numbers and chromosome location of Vps25 equivalogs Click here for file

Additional File 2 Additional Figure 1: Amino acid sequences of full length Vps25 homologs in FASTA format Click here for file

Additional File 3 Additional Table 2: Partial Vps25 sequences Click here for file

Additional File 4 Additional Figure 2: FASTA format of partial Vps25 sequences Click here for file

Additional File 5 Additional Figure 3: Alignment used for phylogeny Click here for file

Additional File 6 Additional Figure 4: Location of introns within aligned Vps25 sequences Click here for file

Additional File 7 Additional Figure 5: Genomic context and organization of human and chimpanzee *VPS25* Click here for file

Additional File 8 Additional Figure 6: Genomic context and organization of mouse and rat *Vps25* Click here for file

Additional File 9 Additional Figure 7: Genomic context and organization of dog and cow *Vps25* Click here for file

Additional File 10 Additional Figure 8: Genomic context and organization of *A. gambiae*, *D. melanogaster*, and *C*. *elegans Vps25* Click here for file

Additional File 11 Additional Figure 9: Genomic context and organization of plant *Vps25 *genes from *O. sativa *and *A. thaliana* Click here for file

Additional File 12 Additional Table 3: Taxa, accession numbers and chromosome location of 'hybrid' splice variants of Vps25 Click here for file

Additional File 13 Additional Figure 10: Multiple tissue PCR analysis of the expression of 'hybrid' *Vps25 *genes Click here for file

Additional File 14 Additional Table 4: *VPS25 *pseudogene details Click here for file

Additional File 15 Additional Figure 11: FASTA format of VPS25 pseudogenes Click here for file

Additional File 16 Additional Figure 12: Alignment of mammalian *VPS25 *coding sequences with pseudogene sequences Click here for file

Additional File 17 Additional Figure 13: Genomic context of primate *VPS25 *pseudogenes Click here for file

Additional File 18 Additional Figure 14: Comparison of full-length Vps25 amino acid sequences Click here for file

Additional File 19 Additional Figure 15: Phylogenetic relationship of Vps25 orthologs Click here for file
