# Supplementary material for: Genetic structure and evolution of the Vps25 family, a yeast ESCRT-II component
Source: BMC Evol Biol. 2006 Aug 4;6:59. doi: 10.1186/1471-2148-6-59 (PMC1579232; doi:10.1186/1471-2148-6-59)
Supplement: Additional File 1 — Additional Table 1: Taxa, accession numbers and chromosome location of Vps25 equivalogs [file 1471-2148-6-59-S1.pdf]

# Additional File 1

## Additional Table 1: Taxa, accession numbers and chromosome location of Vps25 equivalogs.

| Species[a] | Protein accession number or identifier | mRNA (or EST ) accession number or identifier | Genomic DNA accession number or identifier | Chr[b] | Intron(s)? | Comments |
|---|---|---|---|---|---|---|
| **CHROMALVEOLATES**[c] | | | | | | |
| **Alveolates** | | | | | | |
| Apicomplexans | | | | | | |
| *Theileria annulata* | XP_955201 | XM_950108 | CR940352 | - | Yes | Hypothetical protein TA04930. |
| *Theileria parva* | XP_763533 | XM_758440 | AAGK01000005 | 3 | Yes | Hypothetical protein TP03_0505. |
| Ciliates | | | | | | |
| *Paramecium primaurelia** | AAK69405 | AF274570 | - | - | - | Named 'P protein' by Dubrana and Amar [36]. |
| **EXCAVATES**[d] | | | | | | |
| **Euglenozoa** | | | | | | |
| Kinetoplasts | | | | | | |
| *Leishmania brazilia* | LbrM35.0330 | GeneDB_LbruceiLbrM35.0330 | Contig lbraziliensis.chr35 | 35 | No | Conserved hypothetical protein. Sanger gene database identifiers. |
| *Leishmania infantum* | LinJ36.2350 | GeneDB_LinJ36.2350 | Contig LinJ36_20050901_V2.0 | 36 | No | Conserved hypothetical protein. Sanger gene database identifiers. |
| *Leishmania major* | CAJ08982 | - | CT005272 | 36 | No | Hypothetical protein LmjF36.0310. |
| *Trypanosoma brucei* | XP_822717 | XM_817624 | NC_007283 | 10 | No | Hypothetical protein Tb10.70.2510. |
| *Trypanosoma brucei gambiense* | - | gamb1156g04.q1k_7 | - | 10 | No | Conserved hypothetical protein. Sanger gene database identifier. At the amino acid level, this is 99% identical to *T. brucei*. |
| *Trypanosoma congolese* | - | congo361b06.p1k_8 | - | 10 | No | Conserved hypothetical protein. Sanger gene database identifier. By similarity, we assume the third methionine is used as the start codon. |
| *Trypanosoma cruzi* | XP_809751 | XM_804658 | AAHK01000964 | - | No | Hypothetical protein Tc00.1047053503891.110. Note identical allele on database: XP_805678. |
| *Trypanosoma vivax* | - | tviv626f01.q1k_4 | - | 10 | No | Conserved hypothetical protein. Sanger gene database identifier. |

| | | | | | | |
|---|---|---|---|---|---|---|
| **Diplomonads** | | | | | | |
| *Giardia lamblia** | XP_768354 | XM_763261 | AACB01000109 | - | No | Hypothetical protein GLP_72_20526_21083. |
| **Parabasalia** | | | | | | |
| *Trichomonas vaginalis(1)* | 97121.m00179 | - | TIGR Assembly: 97121.<br>Clone name: 1047229024195 | - | No | TIGR database identifiers. One of two homologs of Vps25 in *T. vaginalis*, the only species identified having paralogs. |
| *Trichomonas vaginalis(2)* | 88271.m00525 | - | TIGR Assembly: 88271.<br>Clone name: 1047229024149 | - | No | TIGR database identifiers. One of two homologs of Vps25 in *T. vaginalis*, the only species identified having paralogs. |
| **AMOEBOZOA**[e] | | | | | | |
| **Entamoebidae** | | | | | | |
| *Entamoeba histolytica* | - | - | AAFB01000244 | - | Yes | Full-length homolog identified. RefSeq accession number XP_654057 (XM_648965) only documents a version lacking the first 46 amino acids. This is probably because of an error in automated genome annotation, caused by the short size of the first exon not being recognized. |
| **Eumycetozoa** | | | | | | |
| *Dictyostelium discoideum** | XP_647245 | XM_642153 | AAFI01000009 | 1 | Yes | Hypothetical protein DDB0189483. |
| **PLANTAE**[f] | | | | | | |
| **Green algae** | | | | | | |
| *Chlamydomonas reinhardtii* | - | BP092005 and AW661361 | - | - | - | Two overlapping ESTs used to determine full length sequence. |
| **Rhodophyceae** | | | | | | |
| *Cyanidioschyzon merolae* | - | CMM195C | - | - | - | EST accession number is from the University of Tokyo *C. merolae* genome project |
| **Land plants**[g] | | | | | | |
| **Bryophyta** | | | | | | |
| *Physcomitrella patens* | - | BJ585140 | - | - | - | A frameshift error is predicted in EST sequence due to an extra 'g' (in run of three) and has been fixed. |
| **Gnetophyta** | | | | | | |
| *Welwitschia mirabilis* | - | DT588756 | - | - | - | Full-length EST. |
| **Pinophyta** | | | | | | |
| *Pinus taeda* | - | DR176866 | - | - | - | Full length EST. The 5' sequence has two |

| | | | | | | |
|---|---|---|---|---|---|---|
| | | | | | | ATGs separated by just four amino acids. Either of these may be used, by similarity, as the start codon. We have used the first one in our alignment. |
| **Magnoliophyta**[h] | | | | | | |
| Liliopsida | | | | | | |
| Asparagales | | | | | | |
| *Asparagus officinalis* | - | CV289691 | - | - | - | Full length EST. |
| Cyperales | | | | | | |
| *Hordeum vulgare* | - | AL507087 and AJ436036 | - | - | - | |
| *Oryza sativa* | - | AK069050 and CF303626 | NT_079967 | 1 | Yes | Deduced from two overlapping ESTs. A single nucleotide error is found in the second codon of AK069050 compared to the genomic sequence, and has been corrected. |
| *Sorghum bicolor* | - | CN147918 | - | - | - | Full length EST. |
| *Saccharum officinarum* | | CA206773 and CA112908 | - | - | - | Full length from two overlapping ESTs. |
| *Triticum aestivum* | - | BQ483596 | - | - | - | Full length EST. |
| Eudicotyledons | | | | | | |
| Asteridae | | | | | | |
| *Antirrhinum majus* | - | AJ804426 | - | - | - | Full length EST. |
| *Coffea canephora* | - | DV692659 | - | - | - | Full length EST. |
| *Lycopersicon esculentum* | - | BI208532 | - | - | - | Full length EST. |
| Rosidae | | | | | | |
| *Arabidopsis thaliana** | NP_680720 | NM_148354 | NC_003074 | 4 | Yes | Expressed protein At4g19003. There is a transcript variant, with a longer 5' non-coding region, also on the database (NM_202841) that yields an identical protein (NP_974570): At4g19004. |
| *Brassica napus* | - | CX194664 | - | - | - | Full length EST. |
| *Citrus clementina* | - | CX291560 | - | - | - | Full length EST. |
| *Fragaria vesca* | - | DV438035 | - | - | - | Full length EST. |
| *Gossypium hirsutum* | - | DT460185 | - | - | - | Full length EST. |
| *Glycine max* | - | AW459787 | - | - | - | Full length EST. |
| *Malus* x *domestica* | - | CN494485 and CV629711 | - | - | - | Full length from two overlapping ESTs. |
| *Medicago truncatula* | - | AW691121 and BQ124793 | - | - | - | Full length from two overlapping ESTs. |

| | | | | | | |
|---|---|---|---|---|---|---|
| *Populus trichocarpa* x *Populus nigra* | - | DT526189 | - | - | - | Full length EST. |
| *Populus tremula* | - | CK097172 | - | - | - | Full length EST. |
| *Vitis vinifera* | - | CB972778 | - | - | - | Full length EST. |
| **OPISTHOKONTS** | | | | | | |
| **Fungi**[i] | | | | | | |
| **Ascomycetes** | | | | | | |
| Saccharomycotina | | | | | | |
| *Candida albicans** | XP_722011 | XM_716918 | AACQ01000012 | 5 | No | Hypothetical protein CaO19.11424. As the diploid genome of *C. albicans* has been sequenced and an identical allele (hypothetical protein CaO19.3942) is also on the database (XP_721851). |
| *Candida glabrata** | XP_445378 | XM_445378 | CR380949 | C | No | Unnamed protein product. |
| *Clavispora lusitaniae* (*Candida lusitaniae*) | - | - | AAFT01000026 | - | No | Whole genome shotgun sequence. |
| *Debaryomyces hansenii** | XP_462609 | XM_462609 | CR382139 | G | No | Unnamed protein product. |
| *Eremothecium gossypii** (*Ashbya gossypii*) | NP_986349 | NM_211411 | NC_005788 | VII | No | AGL318Wp is a syntenic homolog of *S. cerevisiae*YJR102C (*VPS25*) |
| *Kluyveromyces lactis** | XP_454194 | XM_454194 | CR382125 | E | No | Unnamed protein product from predicted mRNA. |
| *Kluyveromyces waltii* | - | - | AADM01000214 | - | No | Whole genome shotgun sequence. |
| *Saccharomyces cerevisiae** | NP_012636 | - | Z49602 | 10 | No | Expressed protein, Vps25p. Locus tag: YJR102C |
| *Saccharomyces bayanus* | - | - | AACA01000001 | - | No | Whole genome shotgun sequence. MIT_Sbay_c963_12913. Syntenic ortholog of *S. cerevisiae VPS25*. |
| *Saccharomyces castellii* | - | - | ACF01000023 | - | No | Whole genome shotgun sequence. WashU_Scas_Contig691.11. Syntenic ortholog of *S. cerevisiae VPS25*. |
| *Saccharomyces kluyveri* | - | - | AACE01000012 | - | No | Whole genome shotgun sequence. WashU_Sklu_Contig2413.10. Syntenic ortholog of *S. cerevisiae VPS25*. |
| *Saccharomyces kudriavzevii* | - | - | AACI01000270 | - | No | Whole genome shotgun sequence. WashU_Skud_Contig1794.5. Syntenic ortholog of *S. cerevisiae VPS25*. |
| *Saccharomyces mikatae* | - | - | AABZ01000024 | - | No | Whole genome shotgun sequence. MIT_Smik_c741_1257. Differs by one amino acid to Washington |

| | | | | | | |
|---|---|---|---|---|---|---|
| | | | | | | University sequence of the same gene. This sequence is more similar that in other Vps25 sequences. Syntenic ortholog of *S. cerevisiae VPS25*. |
| *Saccharomyces paradoxus* | - | - | AABY01000366 | - | No | Whole genome shotgun sequence. MIT_Spar_c307_12681. Syntenic ortholog of *S. cerevisiae VPS25*. |
| *Yarrowia lipolytica** | XP_500989 | XM_500989 | CR382128 | B | No | Hypothetical protein YALI0B16786g. |
| Schizosaccharomycotina | | | | | | |
| *Schizosaccharomyces pombe** | NP_596423 | NM_001022342 | AL023706 | II | Yes | Hypothetical protein SPBC4B4.06. |
| Pezizomycotina | | | | | | |
| *Aspergillus fumigatus** | XP_751602 | XM_746509 | AAHF01000005 | 4 | Yes | Hypothetical protein Afu4g12290. |
| *Aspergillus oryzae* | BAE54894 | - | AP007150 | - | Yes | Unnamed protein product. |
| *Botryotinia fuckeliana* | - | - | AAID01001090 | - | Yes | Whole genome shotgun sequence. |
| *Coccidioides immitis* | - | - | AAEC02000030 | - | Yes | Whole genome shotgun sequence. |
| *Gibberella zeae* (*Fusarium graminearum*) | - | - | AACM01000336 | 2 | Yes | We predict the 3' end of the database sequence, accession number XP_388577 (XM_388577) encoding hypothetical protein FG08401.1, to be incorrect by similarity to other orthologs. The 'correct' carboxy-terminal sequence was deduced from the genomic DNA sequence and matching a conserved intron site. |
| *Magnaporthe grisea** | XP_368213 | XM_368213 | AACU01001494 | VI | No | Hypothetical protein MG01031.4. |
| *Neosartorya fischeri* | - | - | AAKE02000005 | - | Yes | Whole genome shotgun sequence. |
| *Neurospora crassa** | CAD11782 | AL356172 | AABX01000719 | 5 | No | Hypothetical protein B23L21.060. |
| *Phaeosphaeria nodorum* | - | - | AAGI0100034 | - | No | Whole genome shotgun sequence. |
| *Sclerotinia sclerotiorum* | - | - | AAGT01000582 | - | Yes | Whole genome shotgun sequence. |
| *Trichoderma reesei* | - | - | AAIL01000563 | - | Yes | Whole genome shotgun sequence. |
| *Uncinocarpus reesii* | - | - | AAIW01000402 | - | Yes | Whole genome shotgun sequence. |
| **Basidiomycetes** | | | | | | |
| *Coprinus cinereus* *(Coprinopsis cinerea okayama)* | - | - | AACS01000060 | - | Yes | Whole genome shotgun sequence. |

| | | | | | | |
|---|---|---|---|---|---|---|
| *Phanerochaete chrysosporium* | - | - | AADS01000433 | - | Yes | Whole genome shotgun sequence. Start codon appears 'lacking'. We have assumed that a single base is missing/misread in the sequence, immediately after the putative ATG, as this would place the start codon in an identical place to that of closely related *C. neoformans* [Additional File 2]. |
| *Ustilago maydis** | XP_760019 | XM_754926 | AACP01000132 | 11 | No | We predict that the second methionine of hypothetical protein UM03872.1 is used to translate the Vps25 equivalog, by similarity. |
| **Zygomycete** | | | | | | |
| *Rhizopus oryzae* | - | - | AACW02000311 | - | Yes | Whole genome shotgun sequence. |
| **Chytridiomycete** | | | | | | |
| *Blastocladiella emersonii* | - | CO968698 | - | - | - | Stop codon 'lacking' where it would be expected and has been created by adding a 'g', where it appears missing, by similarity. |
| **<u>Metazoa</u>** | | | | | | |
| **Echinodermata** | | | | | | |
| *Strongylocentrotus purpuratus* | XP_787383 | XM_782290 | NW_840740 | - | Yes | Predicted protein. |
| **Nematoda** | | | | | | |
| *Caenorhabditis briggsae** | CAE67992 | - | CAAC01000065 | - | Yes | Hypothetical protein CBG13602. |
| *Caenorhabditis elegans** | NP_493230 | NM_060828 | NC_003279 | 1 | Yes | Expressed protein W02A11.2. |
| *Heterodera glycines* | - | CD748153 | - | - | - | Full length EST. |
| **Platyhelminthes**[j] | | | | | | |
| Trematodes | | | | | | |
| *Paragonimus westermani* | - | AT007339 | - | - | - | One of two full length ESTs. |
| *Schistosoma japonicum** | AAW26935 | AY815203 | - | - | - | High through-put cDNA. |
| *Schistosoma mansoni* | - | CD082512 | - | - | - | Full length EST. |
| *Schmidtea mediterranea* | - | DN292820 | - | - | - | Full length EST. |
| **Arthropoda**[k] | | | | | | |
| Chelicerata | | | | | | |
| *Amblyomma variegatum* | - | BM290704 | - | - | - | Full length EST. |
| *Boophilus microplus* | - | CV447217 | - | - | - | Full length EST. |
| Hexapoda | | | | | | |
| *Aedes aegypti* | - | DV351361 | - | - | - | One of many full length ESTs. |
| *Anopheles gambiae** | XP_317805 | XM_317805 | AAAB01008966 | 3L | Yes | Hypothetical protein ENSANGP00000004798. |
| *Apis mellifera* | XP_395839 | XM_395839 | NC_007074 | LG5 | Yes | Hypothetical protein LOC412381. |
| *Acyrthosiphon pisum* | - | CN587513 | - | - | - | Full length EST. |

| | | | | | | |
|---|---|---|---|---|---|---|
| *Bombyx mori* | - | NRPG0355 (partial) | BAAB01158331 and BAAB01098348 | - | Yes | EST identifier is from the silkworm genome database and is for a cDNA beginning at amino acid ~14 of Vps25. Genomic sequence was used to identify the full length ortholog, but the sequence is split over two unmapped contigs, so introns cannot be fully mapped. |
| *Drosophila melanogaster** | NP_610398 | NM_136554 | NT_033778 | 2R | Yes | Hypothetical protein CG14750-PA. |
| *Drosophila pseudoobscura* | EAL24827 | - | CM000071 | 3 | Yes | Hypothetical protein GA13223-PA. |
| *Lutzomyia longipalpis* | - | AM100306 | - | - | - | Full length EST. |
| **Chordata** | | | | | | |
| Urochordata | | | | | | |
| *Ciona intestinalis* | - | BW032610 | - | - | - | Full length EST. |
| *Molgula tectiformis* | - | CJ406275 | - | - | - | One of two full length ESTs. |
| Vertebrata | | | | | | |
| Chondrichthyes | | | | | | |
| *Leucoraja erinacea* | - | CV222568 | - | - | - | Full length EST. |
| Neopterygii | | | | | | |
| *Danio rerio* | AAH67612 | BC067612 | NC_007112 | 1 | Yes | Full length EST. The last exon could not be mapped to the *D. rerio* genome, but this is probably due to gaps in the current chromosome 1 sequence. The lack of the final exon, is probably the reason that only an 'incorrect' version of *D. rerio* Vps25 is currently on the RefSeq database, with a different final exon to that used in the EST, and which is not evolutionarily conserved (see XP_688456). |
| *Fugu rubripes* | - | SINFRUT 00000138690 | - | - | Yes | ENSEMBL database cDNA identifier found using the Fugu Genome Project BLAST at the Institute of Molecular and Cellular Biology, Singapore. |
| *Gasterosteus aculeatus* | - | DW649763 | - | - | - | Full length EST. |
| *Ictalurus punctatus* | - | CV990154 | - | - | - | Full length EST. |
| *Oryzias latipes* | - | AM153702 | - | - | - | Full length EST. |
| *Oncorhynchus mykiss* | - | CX716611 | - | - | - | Full length EST. |
| *Platichthys flesus* | - | DV567885 | - | - | - | Full length EST with single base-call error near 3’ end. |
| *Pimephales promelas* | - | DT086288 | - | - | - | Full length EST. |
| *Salmo salar* | - | DW571474 | - | - | - | Full length EST. |

| | | | | | | |
|---|---|---|---|---|---|---|
| *Tetraodon nigroviridis** | CAG06561 | - | CAAE01014976 | 2 | Yes | Whole genome shotgun sequence. |
| Tetrapoda | | | | | | |
| Aves | | | | | | |
| *Gallus gallus* | - | BU129391 | - | - | - | One of many full length ESTs.<br>Note: the hypothetical protein on the database is incorrect (XP_418142) probably because the 5' sequence (as identified by the ESTs) has not been mapped in the current chicken genome database release. |
| *Taeniopygia guttata* | - | DV950010 | - | - | - | EST missing ATG only (by similarity to chicken mRNA sequence) and this has been added here. |
| Amphibia | | | | | | |
| *Xenopus laevis** | AAH79766 | BC079766 | - | - | - | Expressed protein. |
| *Xenopus tropicalis* | NP_001016078 | NM_001016078 | - | - | - | Expressed protein. |
| Mammalia | | | | | | |
| *Bos taurus** | NP_001015657 | NM_001015657 | NW_929520 | 19 | Yes | Expressed protein. |
| *Canis familiaris* | - | DR104834 | NW_876332 | 9 | Yes | EST sequence. Protein database currently only includes a version with a long N-terminal extension (XP_849270) and not a Vps25 equivalog. |
| *Equus caballus* | - | DN504674 and CX592216 | - | - | - | Full length sequence obtained from two overlapping ESTs. |
| *Homo sapiens** | NP_115729 | NM_032353 | NC_000017 | 17 | Yes | Also known on the database as DERP9 (dermal papilla-derived protein 9). |
| *Macaca mulatta* | - | DV770833 | - | - | - | Full length EST. |
| *Monodelphis domestica* | | | Database location: contig_26874 34718 to 34771 (+)<br>Genomic location: ENSMODG: scaffold_18 53323906 to 53323959 (+) | - | Yes | ENSEMBL identifier found using an ENSEMBL BLAST search. |
| *Mus musculus** | NP_081052 | NM_026776 | NT_039521 | 11 | Yes | Uncharacterized protein. |
| *Oryctolagus cuniculus* | - | DN890652 | - | - | - | Full length EST. |
| *Rattus norvegicus** | P0C0A1 | CK471265 | NW_047339 | 10 | Yes | Full length EST. Protein synonym: Eap20. This gene has yet to be annotated on the |

| | | | | | | |
|---|---|---|---|---|---|---|
| | | | | | | NCBI genomic database. |
| *Sus scrofa* | - | BG608976 | - | - | - | Full length EST. |
| **RHIZARIA**[l] | | | | | | |
| | | | | | | None identified to date. |

[a]Classification of species is according to the new classification of eukaryotes, rather than using the traditional Kingdoms [96, 97].
[b]Chromosome location.
[c]Note that:
(i) No homologous ESTs were found in the haptophyte sequence databases at the Protist EST Program for *Isochrysis galbana* or *Pavlova lutheri*.
(ii) No Vps25 homologs were detected in the unfinished *Cryptosporidium hominis* or complete *Cryptosporidium parvum* genomes, or in *Toxoplasma gondii* genomic sequences deposited at TIGR.
(iii) A partial sequence was found in the stramenopile *Thalassiosira pseudonana* [Additional File 2].
(iv) No Vps25 homologs were detected in the 'completed' *Plasmodium falciparum* database, however there are still gaps in sequence on many of the chromosomes. Homolog(s) were also not found in the unfinished *Plasmodium berghei*, *Plasmodium chabaudi*, *Plasmodium vivax*, or *Plasmodium yoelii yoelii* genomes. The closest match was protein PY03607 from *Plasmodium yoelii yoelii* (accession number: XP_723788). This is a hypothetical unspliced gene, and the amino-terminal sequence does not contain proline residues conserved in other Vps25 homologs. It cannot be determined whether splicing is a possibility to provide an alternative 5' end, as the shotgun sequence does not extend much past the current putative start codon. In *Plasmodium berghe*i the sequence analogous to that of PY03607 is proposed to encode a different hypothetical protein (accession number CAH96581), while the *Plasmodium falciparum* genome annotation does not describe a homolog. The *Plasmodium chabaudi* genome (CAAJ01000332) encodes a sequence similar to PY03607, but this has not been annotated as a hypothetical protein. The possibility that PY03607 is a *VPS25* homolog cannot be investigated further at this stage. Until full sequencing of *Plasmodium* species has occurred the absence of Vps25 homologs will not be conclusive.
[d]No homologous ESTs were detected in sequences from the preaxostyle species, *Streblomastix strix*, at the Protist EST Program.
[e]A partial sequence was detected in the true amoeba, *Hartmannella vermiformis* [Additional File 2].
[f]No Vps25 homolog was detected in the complete glaucophyte genome of *Cyanophora paradoxa* or in ESTs from *Glaucocystis nostochinearum*. No homologs were detected in the chloroplastida sequences of *Acetabularia acetabulum*, *Helicosporidium* sp., *Prototheca wickerhamii*, *Scenedesmus obliquus*, at the Protist EST Program. No homologs were found on the Kazuza *Porphyra yezoensis* (red algae) EST database at the Kazusa DNA Research Institute.
[g]Partial sequences were found for the cycadophyte *Cycas rumphii*, and ginkgophyte, *Ginkgo megasporophyll* [Additional File 2].
[h]A partial sequence was detected in the magnoliid, *Liriodendron tulipifera* [Additional File 2].
[i]Strikingly, no Vps25 homolog(s) were detected in the fully sequenced microsporidian genome of *Encephalitozoon cuniculi*.
[j]A partial cestode sequence is provided in Additional File 2.
[k]Partial sequences were identified in a chelicerate species and a crustacean species [Additional File 2].
[l]No homolog was detected in the Chlorarachniophyte database (*Bigelowiella natans*) at the Protist EST Program.

*Correct full length orthologous sequence listed on current Pfam PF05871 entry
