# Supplementary material for: Genetic structure and evolution of the Vps25 family, a yeast ESCRT-II component
Source: BMC Evol Biol. 2006 Aug 4;6:59. doi: 10.1186/1471-2148-6-59 (PMC1579232; doi:10.1186/1471-2148-6-59)
Supplement: Additional File 2 — Additional Figure 1: Amino acid sequences of full length Vps25 homologs in FASTA format [file 1471-2148-6-59-S2.pdf]

Additional File 2

Additional Figure 1

Amino acid sequences of full length Vps25 homologs in FASTA format.

Standard single-letter amino acid abbreviations are used. Genus names are abbreviated to a single letter, while species names are abbreviated to the first four letters. Homologs are in order of taxonomical grouping [Additional File 1].

>TannuVps25
MDSVDFNTHVKFKNFPPLYTEQINNLTLSKQLEIWHKIINDEVITNYSLHKIGTETINFP
PFKNEEIVRNVNVSFLALILEYLAEKQYAFYLHPIQLFCKKH
NVTIWGALFLKKNHKGTTLFQIHDEYTKSLNPKDNKAETDEIDSLKKKRNLLVKSTFR
FGVFPYPLSEMTNSVLECIKSQCTNRDIETIYHIFYSKKECNKDFNKFPEENLAFILS
KLSVNNQITLSFNDSVPLDSLNNKNVGVQLL
>TparvVps25
MDSVDFNTHEKFKNFPPLYTEQINNLTLSKQLEIWHKIINDEVTANYSLHKLGTASVNFP
PFKNEEILRNVDVSFLALILGYLVEKQYAFYLHPIQFFCKKNNVSIWGALFLKKSHKGST
LYQIHQDYTKALNSKDNKVEGDEIESLKKKRNLLLKSKFNFGVFPYPLTEMANSVLECIK
SQCTTRDIETVYHIFYSKRECNKDFNKFPEENLAFILSYLCVNNKLTLSFNDSVPLDSLN
NKNVGLQLL
>PprimVps25
MWHYLNSFEYTYLLIIYIKIITFKTLEISSILHVHSLSQVYSLQDHKETRKKQITQWSEI
VHLYFQSHKILESSISEILNFPIFQDSSSGIIKRLDSSEIKEILNQMAQLGSIEWKNDQN
FSVNLVSPFELADAIYAWAKEKKLIGYTETLRGITEGSQTDQSKKFYNLPQEQILKACLI
LEETGRCQVYEFDGLYSIKFI
>LbrazVps25
MSSEHWSFFGLPPFFTEQHSPATLDRQSTLWSNLLLDHAIYHTQRTAGGDTNPLLRFYTT
NSDIFYNPAINKRLSPEGAHTMLQALVARHPNHAVVVSDGGPKDFSVLVCTTEGGLKGIE
ENLLRYILEHGEVQTVAMLSKKGTVMTFDELAGGAALGYGQSRPAYLARLSSAAVPVADV
GDLSEEQAVRTYLHALDHHPVSVMRPFKVTLFNLDGTTTQPYQGVKFGGE
>LinfaVps25
MPSEHWSFFGLPPFFTEQHSPATLDRQCTLWSNLLLDHAIYHAQRTAGGDTNALLRFYTT
QSDIFYNPAIDKRLSPEAAHTMLQSLVARHPNHAVIVSDGGPRDFSVLVCTTEDGFKGME
ETLLRYILDHGEVQTTAMLSKKGTVMTFDELASGAALGYGQTRPAYLARFSSAAVPVADV
GRLSEEQAIRTYLHAVNHRPVSAMRPFKVTLFNLDGTATQPYQGVKFGGE
>LmajoVps25
MSSEHWVFFGLPPFFTEQHSSATLDRQCTLWSNLLLDHAIYHAQRTAGGDTNALLRFYTT
QSDIFYNPAINKRLSPEGAHTMLQSLVARHPNHVVIVSDGGPRDFSVLVCTTEDGFKGME
ETLLRYILDHGEVQTTAMLSKKGTVMTFDELASGAALGYGQTRSAYLARFSSAAVPVADV
GRLSEEQAIRMYLHALNRRPVSAMRPFKVTLFNLDGTATQPYQGVKFGGE
>TbrucVps25
MSKEPGHWDFFKLPPFFTLQPSPSALERQMALWGNLVMDHAAFHAQHRKRDTCPLLRLYS
CNSGLFRNETINRRLRPEDVKKVISSLVAQWSEHCVLTSDGDGMGDVSVLVTTSKGGLKE
LEQSLLAWILERGAGTTVAYLSQKGVVMTFDELVDGQCLLYSRESEQLLPRLTQEAVPIE
DVGALSQEQAVRCFLHTLAERPVSHKGLFSVTLFNLDGSDRRPYEGVKFGGAAI
>TbrucgambVps25
MSKEPGHWDFFKLPPFFTLQPSPSALERQMALWGNLVMDHAAFHAQHRKRDTCPLLRLYS
CNSGLFRNETINRRLRPEDVKKVISSLVAQWSEHCVLTSDGDGMGDASVLVTTSKGGLKE
LEQSLLAWILERGAGTTVAYLSQKGVVMTFDELVDGQCLLYSRESEQLLPRLTQEAVPIE
DVGALSQEQAVRCFLHTLAERPVSHKGLFSVTLFNLDGSDRRPYEGVKFGGAAI
>TcongVps25
MAKEPSHWDFFKLPPFFTLQPAPAALERQLMLWGNLVMDHAASHAPNTRSDACP
FLRLYSCTSSLFRNDSINRRLSSEHAKKVLSSLAVQRPEHCVLTGNDDNEEDVSLLVSTN
KGGLKELEQSLLEWILERGVGTTVAHLSQKGVVMTFNELVDGHCLAYKCESSGLVRRLSQ
DVVPIEDVGVLSHEQAVRCFLHTLSVRPISHQGLFSVTLFNLDGSNRRPYEGVKFGGAAA
R
>TcruzVps25
MSIEPDHWSFFQLPPFFALQPGPTALARQTSLWGSAIIDHAAYHAPRTRPGVCPFLRLYK
STSDVFRNPSLNRRLPPEAAARMLESLVAQHPNHCAVVLPGNEKGENDDADFAVLLACNE
SGLKGLEQALLSWILDRGAGTTTAHLAQKGVVMTFDELAESNCLAYRRDREPFLKRLSAA
PVPVGDVGALSNEQAIRTLLHSLKSRPLSALSPIHITLFNMDGSDRRPYEGVKFGGAVAC
>TvivaVps25
MTVEPNHWNFFKLPPFFTMQPAAAALARQVTLWEDLIMDHAAHHARLTNRGVCSHLRLYS

STSDVFRNNALRRRLPPEDATKILSSLASRRLSHCISVEDANGDYSVLVACNEGGLAAIE
QSLLAWILERGAGTTAASLAQNGAVMTFDELVEGQCLVYKRENEPMICRLAQDDVPVGDV
GALTEEQAVRTLLKALATRPASIRWPYRITFFNLDGSSVEPYQGVKFGGVVC
>GlambVps25
MACKDVLESSELWYFPPFFTLQQKIEQADVCTEQLKQWDALLMKWCYEVKSREIPKDNYL
EKAIFKNTKINRKVSDELFREIIDWFKRKGRLVEINKSYYLLSAPEDTIPDSLLRLLKNN
NFTVGCIITVLELKKTMDKDASDLFALPDPVLKKALEALQKSGNLALYQEGPVFGDETGI
KILGL
>TvagiVps251
MSKKKATKKPPTFEFPDFYDYPPFWTLQTNSQTKKQQLDLWASFICAYTKFYKKTEIDMI
QALDAPLFNNQKLGRRVSQQMMTEIIDYMVQSENAKWLESTKTRARIIWRTTQQIGDMVR
QYLDNIGSLNTMMTYEELINGDETEGEGFHGLSADEFHDAMTFMESKGRCKIIPGSSLVE
YGVKFF
>TvagiVps252
MSFKFPPIYSFPPFWTIQPCMEARRMQTQTWCDLILSWCKANKKEDLKVADALKTDLFNN
KAIGRALSQQDAEFFLDQLVQRQNAQWSDDKHTKCKIIFRKPAQWANIFYQWAVKNNLQG
VVFTFYELREGDDTQSEAFHNMDQEQMLKAIECLVKEKKANLIKAADFDENGVKFL
>EhistVps25
MTFGIPEFAKFPPFYTIQLVDKTKNQQLQLWSQLILKYCE
CIKKPIMKQSEFNKLPIFHNEELH
RTLSENGIELVKEFMVNNNKIIDLNKSSKLILLYKPLREWGKELYEYGNSKGLIGQSDTF
FSIENDKESVFYQMDDELLIEGLNSIKEQGKMKLVQHEGEYGIFWLK
>DdiscVps25
MSNQVFQFPPYYHKEPFFTIQPILNTRKKQFQMWQDLILQYCRYYKIYELDINESIKSNS
VLFNNEKINRKLSREALKSIIDDIIENGFAEWVDKEKEKEKEKDKDNNNNNRVLIMWRKP
DEWASLIYKWVADCGLLNTVLTVWEIQNGDDSKKQEFHQLNTTILMKSLKVLEKQSKCQT
FSQDENVGVKFFSI
>CreinVps25
MGSFAFPYFHNYPPYFTLQPVKETRDKQVALWCSLVLQYCQHTKTFVLDVQGDSPLFVNK
VINRKLNQEARVAILDELATQGRAEWMDGGKTRCLVYWRRVDEWAGAVTEFVRTFGLSDS
VMTVDELSSGDDVRGTDLYGVHPEILTRALKLLEAQGKVRTFKGATPEELGVKFI
>CmeroVps25
MLIRTTPVQETGNHQPKTEPASFAFPPWHQYPPLYTIQPCARTRERQLYLWRRLILDYCE
HFWILTLRLYDAESPQMPLFCNRAIQRRLSRSALQCIFQELVLSGDAAWSNPEEKEELLI
FWRSPRLWADELLRVVAQYGQNKGGVFTLPELASMLGERCSLGRQQRAQALQTAIPIAFL
EHILELLAAQGKARVFSTNEGRGVKFDFS
>PpateVps25
MGEFSFPYFYNYPPYFTLQPMKDTRDKQIQLWKELILKYCKHH
KLFLIDLEEEFALFENTSIQRKLTFEAREQFLSALIADGRAEWLDKNHRKCLILWRRIED
WADSLLNFVRENGIELMTLEEIISGDETYGTELAGLDRGVLVRAVKILEQRGKAAMFKGS
STDDDGVKFSPN
>WmiraVps25
MNPKNMGEFRLPDFFN
YPPYFTLQPVRDTRQKQVQLWKELIVNYCKHHKTYIINLEEEFPLFSNPAINRKLSHEAK
EAFLSALVNEGKAEWLDKGHKRCLILWRRIEDWAAYILHFVHENGLENGVMTLEELQTGV
ESRGTDLAGIDRTVLVKALRLLEQRGKAVIFKGSATDDEGVKFSA
>PtaedVps25
MADLKLPDFFNYPPYFTLQPVRDTREKQVQVWKELILNYCKHHKIF
IISLEEEFPLFSNPAIDRKLSYEAKKVFLSALVSEGRAEWLDKGQKKCLILWRRIQDWAE
YILKFVHENGLEDGVMTLEEIRAGVESRGTELAGIDRIVLIRALKLLEQRGKAVIFKGTS
TDDEGVKFSA
>AoffiVps25
MQRLGDFRLPHFFNYPPYFTLQPVRETREKQVQLWKELILDYCRSQKIFIIG
LEEEFPLFSNPVIERSLNHEARGVFLSALVSEGRAEWMDKSHKKCLILWLRIQDWADHII
KFVKDNGLEDSVVTVEDIRSGFESRGTELAGIDRGVLMRALRLLEQKGKAAIFKGSSSDD
EGVKFSV
>HvulgVps25

```
MQRTVDFKLPHFFNYPPYFTLQPVRETREKQVQLWKEL
ILDYCRSQKMYIISLEEDFPLFSNQKIERSLSYEAKEVFLAALVSEGR
AEWIDKSHKKCLILWLRIQDWANYILDFVKENGLEVTTIEDLRSGIETHGTELAGIDRGV
LMRALRLLEQKGKAVIFKGTSADDEGVKFSA
>OsatiVps25
MQRLGDFRLPPFFNYPPYFTLQPVRETREKQVQLWKDLILDYCRSQKLYIISLEEDFPLF
SNPKIERSLSHEAKEVFLAALVYEGRAEWMDKGHKKCLILWLRIQDWANYILNFVKDNGL
EDSVMTVEEIRSGIETRGTELAGIDRGVLMRALKLLEQKGKAAIFKGTSADDEGVKFSA
>SbicoVps25
MQRLGDFRLPPFFNYPPYFTLQPVRETREKQVQLWKDLILDYCRSQKIHTIS
LEEDFPLFSNPKIERSLSHEAKEVFLAALVSEGRAEWMDKGHKKCLILWLRIQDWANFIL
NFVKDNGLEVMTIEEIRSGIDTRGTELEGIDRGVLMRALRQLEQKGKAAIFKGTSADDEG
VKFSV
>SoffiVps25
MQRLGDFRLPPFFNYPPYFTLQPVRETREKQVQLWKDLILDYCRSQKIHTISLEEDFPLF
SNPKIERSLSHEAKEVFLAALVSEGRAEWMDKGHKKCLILWLRIQDWANFILNFVKDNGL
EVMTIEEIRSGIDTRGTELEGIDRGVLMRALRQLEQKGKATIFKGTSADDEGVKFSV
>TaestVps25
MQRTVDFKLPHFFNYPPYFTLQPVRETREKQVQLWKELILDYCRSQKMYIISLEEDFPLF
SNPKIERSLSYEAKEVFLAALVSEGRAEWIDKSHKKCLILWLRIQDWANYILHFVKENGL
EVTTIEDIRSGIETHGTELAGIDRGVLMRALRLLEQKGKAVIFKGSSADDEGVKFSV
>AmajuVps25
MQTLGEFKLPHFFNYPPYFTLQPVRDTREKQIQLWKELI
LDYCRTQKIFVIGLDEDFSLFTNSVIERSLSHEASEAFLSALVLEGRAEWLDKGHRKCLL
LWHRIQDWADLILSFAKDNGLEDSVMTVEEIRSGIESRGTELHGMDRTILMRALKHLEHK
GKLAIFKGTSADDEGIKFSI
>CcaneVps25
MQKLGEFKL
PHFFNYPPYFTLQPVRDTREKQVQLWKELIIEYCRAQKVFIIGLEEDFPLFSNHAIERSL
SHEARVAFLSALVSDGRAEWTDKGHKKCLILWHRIQEWADLIVHFVKENGLEDNVTTVEE
IRSGTESRGTELYGIDRSVLMRALKLLEHKGKLVIFKGASTDDEGVKFSL
>LescuVps25
MQKLGEFKLPNFFNYPPYFTLQPVRETREKQIQLWKELIIDFCRTQKIFVIALEV
DFPLFSNAAIERSLSHEAREAFLSALVSDGRAEWMDKGHRKCLVLWHRIQDWADLIVNFV
KENGLEDSVMTVEEIRSGVESRGTELHGIDRTVLMRALKVLEHKGKLAIFKGTSADDEGV
KFSV
>AthalVps25
MQKLADFKLPQFFNYPPYFTLQPVRDTREKQIQLWKELILDYCKSQKIFLIGVEEDFPLF
SNSAIDRSLSHEARETFLSAIVGEGRAEWLDKGHRKCLILWHRIQDWADIVLQFVRDNGL
EDSVMTVEEIRSGTESLGTELQGIDRTILMRALKLLENKGKLALFKGTSADDEGVKFSV
>BnapuVps25
MQKLGDFKLPQFFNYPPYFTLQPVRD
TREKQIQLWKELILDYCKSQKVFLIGVEKNFPLFSNSAIDRTLSHEAREIFLSAIVGKGR
AEWLNKGHRKCLILWHRIQNWADIILKFVRENGLKDSVMTVEKIRSGTESLGTELEGIDR
TILMRALKLLENKGKLALFKGTSADDEGVKFSV
>CclemVps25
MQKLGDFKLPAFFNYPPYFTLQPVRDTREKQIQLWKELILDYCRTQKVFLILLEEE
FPLFSNPVIERSLNNEARETFLSALVSEGRAEWLDKGHRKCLILWHRIQDWADIILGFVK
DNGLEDSVMTVEEIRLGIESRGTELHGMDRTILMRALKLLEHKGKVAIFKGTSADDEGVK
FSV
>FvescVps25
MQKLGDYKLPQFFN
YPPYFTLQPVRDTREKQIQLWKDLILDYCRTQKIFVIALEEDFPLFSNPAIERSLSHEAR
EAFLSILVSDGRAEWLDKRHRKCLILWHRIQEWANIILNFVKEYGLEDSVMTVEEIRSGI
ESRGTELHGIDRTILMRALKLLEQKGKLAIFKGTTADDEGVKFSI
>GhirsVps25
MQKLGDFKLPHFFNYPPYFTLQPVRETREKQIQLWKELILDYCRTQKIFVIR
```

```
LEEEFPLFSNSVIERTLSHEAREAFLSALVAEGRAEWLDKGRRNCLILWHRIQEWADIIV
NFVKDNGFEDSVMTVEEIRSGIESRGTELQGIDRTILVRALKLLEHKGKLAIFKGTSTDD
EGVKFSV
>GmaxVps25
MQKLGEFKLPPFFNYPPYFTLQPVRDTREKQIQLWKDLILDFCKTQKIFIIGLEEEF
PLFTNHGIERSLTHEAREAFLSALVSEGRAEWMDKGHRKCLILWHRIQDWADILIQF
AKDNGLEDGVVTIEEIRSGTESQGTDLHGIDRTILNRALKLLEQKGKLVVFKGTSTDDE
GVKFSI
>MalusVps25
MQKLGDFKLPQFFNYPPYFTLQPVRDTREKQIQLWKDLILDYCKTHHI
FVIGLEDQEFPLFSNPAIERSLTYEAREAFLSALVAQGRAEWLDKSRRKCLVLWHRIQDW
ANIVLDFVKDNGLEDSVMTVEEIRTGIESRGTELHGIDRTVLMRALKMLEQKGKLAIFKG
TTGDDEGLKFSI
>MtrunVps25
MQKLGEFKLPHFFNYPPYFTLQPV
RDTREKQIQLWKELILDYCKTQKLFVIALEEEFPLFTNTVIERSLTNEAREAFLSALVSE
GRAEWMDKGHRKCLILWHRIQDWADILLQFAKDNGLEDGVVTIEEIRFGTESQGTELHGI
DRTILNRALKLLEQKGKLVVFKGTSTDDEGIKFSV
>PtricVps25
MQKLGDFKLPQFFNYPPYFTLQPVRDTREKQVQLWK
ELILDYCRTQKIFVIGLEEEFPLFSNHLIERSLSNEAREAFLSALVSEGRAEWLDRGHRK
CLILWHRIQDWADILLHFVRDNGFEDSVMTVEEIRTGVESRGTELHGIDRTILMRALKLL
EHKGKLAIFKGTSADDEGVKFSV
>PtremVps25
MQKLGDFKLPQFFNYPPYFTLQPVRD
TREKQVQLWKELILDYCRTQKIFVIGLEEEFPLFSNHLIERSLSNEAREAFLLALVSEGR
AEWLDRGHRKCLILWHRIQDWADILLHFVRDNGFEDSVMTVEEIRTGVESRGTELHGIDR
IILMRALKLLEHKGKLAIFKGTSADDEGVKFSV
>VviniVps25
MQKLGDLKLPNFFNYPPYF
TLQPVRDTREKQVQLWKELILDYCRTQKIFVIGLEEEFPLFSNPVIERTLSHEAKEAFLS
ALVSEGRAEWMDKGHKKCLILWHRIRDWADLILRFVRENGLEDSVMTVEEIRSGTESRGT
ELHGMDRTVLMRALKQLEHKGKLAIFKGTSADDEGVKFSL
>CalbiVps25
MSDPILQFEFPKIYSFPPFYTQQPNTTVLNQQLDSWVSIILHYCEYYRITSLSIEGIPKH
SQLEVPLSSLSSIFINKTINRQVNSDFQKLIVKHLIHNKKAEFINPKKPELGVYIYWRSL
VDWGDLLYQYVDDTGQKGTVLTIYELTKSEETTVPQDLHNLDETFLVKIIKDYLIKQGKA
QLLIDENNEIGGVKIV
>CglabVps25
MPSIYAFPPLYTRQPNSLVRKQQIDTWIDILTEWCKSHRVFELGKDGVPVRESDASDADD
GADGGTTTGNEAGRSLFKNEEINRAVPPLFIDEIWSVMATRGVALVTEGRASYYVLWRTL
DSWASLILQWFETVGKLNQVVTLYELTESDETADWEFHSMPLPLLHRCLKPLCNRNRATL
MKDEHGTPVALKVV
>ClusiVps25
MFEFPKIHSFPPFYTKQRNATILENQLEAWGALILDYCEHFRIFSILDSGTVLVQ
QQPQELPPLFENKLIERAVLDEFRKEIFAHLVSKLGRAAYVDPKKRDAGILLFWRTPAEW
AALIREHVESTGQLGTVLTVYELTKLEETDPLLRNIDYNLFVRAIDVLMKQGKAQVLKAE
DGSSQIGGVKII
>DhansVps25
MTTVFEFPKIHSFPPLYTKQPNLTILHNQLESWGEIILSYCQHY
KITSLTLGGSVLHTQLNDVTTNEIPPLFENKNINRAVNDDFKAMIFKHLIHKLHRAEY
INPKQPETGILIYWKTLIEWANILHDFVERTGQLGSVLTIYELTKLEDSGVDEDLKDL
DYNLLVRILKGVLIKQGRAQILMNEDGTQIGGVKIV
>EgossVps25
MAMASELPSIYNFPPLYTRQPNSLIRAQQLDAWLGVLKDYARGRRVWIMG
```

HDGSAREPKDEGSVFFNVQLQRRVPGPFVDEIWAHALAKGAALRHSADSF
YVLWRSLDSWSALLLQWFETCGRLNQVVTIYELVAGDESAMWEFHGMHDG
LCELALGKLVERGRATLISNDGSVVAVKVI
>KlactVps25
MTLETPQIYKFPPLYT
PQTNKLIRKQQLQTWESIILQTCAQLSKWCINKNGKIYDQSTGELLISIFENPEIQRA
CSLEFQQEIWAYMLQNETALQLKESEDSNMIAIFWESLDSWSSTILEWCETSGKLNQV
ITIYEICESDENTNCKFYGMPSSFCLLVLHRLVDRNRATLLKDQGKIVGVKIV
>KwaltVps25
MTEKLPPIYNFPPLYTLQPNVLIREQQLNTWCDLILEFAKTTAAWCMSQEGSIIKDSE
NSDMSIFRNESIQRAVPAPFIEQIWSKMVQTEKALKLNGVYLILWKSVDHWSSQILQWFE
TSGKLNQVVTLYELLEGDETLGWEFHGMHSSVCEVCIQRLCDRGRATLLKEQNKIMGLKVV
>ScereVps25p
MSALPPVYSFPPLYTRQPNSLTRRQQISTWIDIISQYCKTKKIWYMSVDG
TVINDNELDSGSTDNDDSKKISKNLFNNEDIQRSVSQVFIDEIWSQMTKE
GKCLPIDQSGRRSSNTTTTRYFILWKSLDSWASLILQWFEDSGKLNQVIT
LYELSEGDETVNWEFHRMPESLLYYCLKPLCDRNRATMLKDENDKVIAIK
VV
>SbayaVps25
MMASLPPVYSFPPLYTRQPNSLTRRQQLSTWIDIISQYCKSKKIWYMSAD
GTVIDDDRSENGKIENEYSKEVGKNLFNNEEIQRSVPQVFIDEIWSQMVK
EGKCLPIDQSGRKSNNTTTTRYFILWKNLDNWASLILQWFEDSGKLNQVI
TLYELSESDETLGWEFHGMPENLLYYSLKPLCDRNRATMLKDENGKVIAI
KVV
>ScastVps25
MQPLPPIYSFPPLYTRQPNSIIRNQQLNAWIDLILQYARENKCWTMAKTG
TSTADPKFNIFHNESIQRSVSPMFIDEIWALMIKQSKAIVNNDNNNNYFI
LWQNIDSWASLILQWFEDSNKLNQVVTIYELSQGDETINWEFHQMPEPLL
ILALKPLCKRNRATMLKDEDGAPIAIKVI
>SkluyVps25
MNKQLPPIYSFPPLYTRQPNALIRQQQLDTWCDLLVQYAKQRRAWCMGSD
GTIMVQEGSLDDSRDNEESIFLNSQIQRTVPSPFVEEIWARMLQSGKVIK
RQNGYYVLWKDLDSWSSNILQWFETTGKLNQVVTIYELLEGDESIGWEFR
GMNPELCEMCLEKLCSRGRATTLKENGKIMGVKVV
>SkudrVps25
MASLPPVYSFPPLYTRQPNSLTRRQQISTWIDIISQYCKGKKIWYMSADG
TVMNDNTSGSENTDDDDSKKARKNLFNNEDIQRSVSQVFIDEIWSRMVKE
GKCLPIDQTGKKSGNATSTRYFIMWKSLDNWASLILQWFEDSGKLNQVVT
LYELSEADETLDWEFHGMPENLMYYCLKPLCDRNRATMLKDENGKVIAIK
VV
>SmikiVps25
MASLPPIYSFPPLYTRQPNSLTRRQQISTWIDIISQYCKSKRTWYMSADG
TVINDSTLDGRNTDDNDVKKTSNNLFNNEDIQRSVPQEFIGEIWLQMAKE
GKCLPIDQSGRKSSNTTTTRYFVLWKSVDNWASLILQWFEDCGKLNQVVT
LYELSQGDETINWEFHGMPENLLYYCLKPLCDRNRATMLKDENDKVIAIK
VA
>SparaVps25
MTALPPVYSFPPLYTRQPNSLTRRQQISTWIDIISQYCKCKKIWYMSADG
AVVNDNSLDSGNTDNDDSKKVSKNLFNNEDIQRSVSQVFIDEIWSQMAKE
GKCLPIDQSGRKSNNTTTTRYFILWKSLDNWASLILQWFEDSGKLNQVIT
LYELSEGDETFSWEFHGMPENLLYYCLKPLCDRNRASMLKDENDKVIAIK
VV
>YlipoVps25
MNSEIYNFPPFFTRQPNETTWQAQLSHWKDVILTHSRETKQWRL
SNTDSIFENKKIQRRLKPDVIQLVLSDLVSNKKADWVDSKTKTEVWIWWRSAEEWATL
ILAWIDSTGQNGSIVTFYDIAGDDSPGVPEMVGMDSTMLHKVCQVLVHQGKAAVMRDE
DGNEVGLKV

>SpombVps25
MRVPSIYNFPPFFTRQLNDNTWHSQKAAWQMWILLWCRENRQTS
ITINPELLESSLLHNSTIHRTLPLSVFREIVEDMVKQNLAEWTEKRNPKDVFWVYWRS
ISEWGNMILKWLSDMGREGSICTFYELQEQYKEVDCLDEVLLHKVLELLMKKGNIELM
KGSSGKYSGFKVLKA
>AfumiVps25
MSQSQSQSQSHSTTTFQFPPTYSFPPFFTRQPNSTTRLSQLQKW
SSLIQSWCRHHRIYRLSLIEAIESPLFHNATLRKRLSLSEARAVLDWMAKPEEEGGGG
RRAEWIDGGSKSVAWIWWRRPEEWAGIVADWVEATGQKNVVLTVYELLEGEATMSQEW
HGMDADVMLKSLNILVKRGKAQVFGSEGQEGVKFF
>AoryzVps25
MSNSTPFQFPPTYSFPPFFTPQPNSTTRLSQLQKWSLLIQSWCRHHRTYRLSLIEAIESP
LFHNSTLRKRIPLSEARNILDWMAESEEKGGGGRRAEWVDGTNKTIAWVWWRRPEEWAGI
LADWVENTGQKNVVLTVYELVEGEATMSQEWHGMDVDVMMKSLNVLVKRGKAQVFGSEGQ
EGVKFF
>BfuckVps25
MSTSQTQTSVKIFKFPREH
SFPPFFTLQPTSSTVHAQLRKWSDLILSYFAFHRLFRLTVSTLLSSELFKNERINRRLDE
EGLREVLEFMRKEGRVEWIGGGGNGKVGGDICWVWWRKVDEWARVIEDWVDETGQRGSVL
TLYELVEGEGGRGA
EFHGLDTEILQKALAILVKRGKA
QVFGQEDQQGVKFF
>CimmiVps25
MPPLTSHPATPTPAEQNTFPFPAPHSFPPFFTLQPNAQTLLSQLQKWSALIQAYCRHHRL
YRLSLVDALDSPLFHNKQIRKRLSLVDARRIVDWMCGAQGGRRAEWVGGEAGGKSVAWIW
WRRPEEWAGVIADWVEETAQKNTVLTLYELTEGEATMSQEFHG
MDPDVLQKSLHVLVKRGKAQVFGNEDQQGVKFF
>GzeaeVps25
MAATTTTSAPADTFKFPREYHFPAFFTRQTNLTTLHAQHNKWADLILAYARHNRIFRLSL
SEAADSDLFVNRKLDRRLQFDDIRDVVSFMHTDGRVEYVGGGTSGDVVFLYWRKPEEWAE
LVENYVEESGQKGSVLTVYELVEGDGTKG
TDIHGMDTDVLLKALNVLVKRNKAQIFGQDDSLGVKFF
>MgrisVps25
MPPSTEPFPFPREYHFPPFFTRQTNLTTHHAQLTKWSALVLAYCRHHRIFKLPLTTAAAA
SSSTSSTTDPLQSQSQSQSSSATDELFHNKTLNRRLSPADVREVIDFMCKEGRAEYCSPG
TGQAGGDVAWIYWRSPEEWAQLIEGWVDETAQKGSVLTLYELVEGDGTLGTDIHGIDRDM
LHRALQLLVKRGRAQIFGQEDSQGVKFF
>NfiscVps25
MSQTQSHPTTTFQFPPTYSFPPFF
TPQPNSTTRLSQLQKWSSLIQSWCRHYRIYRLSLIEAIESPLFHNATLRKRLSLSEARAV
LDWMAKPEEEGGGGRRAEWIDGGSKSVAWIWWRRPEEWAGIVADW
VEATGQKNVVLTVYELLDGEATMSQ
EWHGMDADVMLKSLNV
LVKRGKAQVFGSEGQEGVKFF
>NcrasVps25
MSTTSPAPSDSSTPNNSNPKTTTASTLLPNDTFPFPREFFFPPFFTRQTNLTTHHAQLTK
WSSLLLAYCRHHRLFRLSLNPDALPFHNPRINRRLAPGDIRRVVDFLRRDGRAEYVLPLG
QTKKDGAMTTTARGGGGEAGEGGEAFIYWRTPEEWGSLIEGWVEETGQRGSVLTVYELRE
GEGTRGTEIWGMDGDVLVKALGTVVKRGKAQIFQSGEDSLGVKFF
>PnodoVps25
MATASPLPSNFSTSSTLPPTTTQPAASTGFQFPPHYSFPPFFTLQPTASTRSS
QLLSWSTLIQSYCRHHRIFTLSLIDALETPLFNNTALRRRLSLRDAKTILTWMSTPEGGN
RVEFINASKSKKAAVEEEGGRCWIFWRRPEEWSAVLEEWVDRTGQKGTVLTLYEIVEGDA
SRKEEFWGMDLELLMRSLGVSVKRGKAQIFGGEGSEGVKFF
>SscleVps25
MSTPQTITPAKTFKFPREHSFPPFFTLQPTSATLHAQLRKWSDLILSYFAFYRLFRLTIS
TLLSSELFKNERINRRLDEEALREVLEFMRKEGRVEWIGGSGSGSGKVGGDVCWVWWRKV

DEWARIIEEWVDETGQRGSVLTLYELVEGEGGRGAEFHGLDAEILQKALAILVKKGKAQV
FGQEDQQGVKFF
>TreesVps25
MATTAATPSPTPPTSTSTSTPTTTTGFAFPREYSFPPFFTRQPNIAIHHAQL
TKWSALVLSYARHHRLFRLVVSSAAESELFHNRAINRRLGPADIREVLDFMRKDGRAEFI
RASSAAAAGSDVGGFSSGAGAAGGGGAGDVVLLYWRKPEEWAALVEAYVDETAQKGSVLT
VYELTEGENTRGTEFHGMDNLVLMKALNILVKQGKAQIFGSEDSLGVKFF
>UreesVps25
MATTPSLLAAPAPTPQQPPSAADTFAFPAPHSFPPFFTLQPNTQTLLSQLQKWSA
LIQSYCRHHRLYRLSLVDALDSALFHNRTIRRRLSLADARKVLDWMCSAEGGRRAEWVGG
EAGGKSAAWIWWRRPEEWAGVIADWVEETAQKNTVLTLY
ELIEGEATISQEFHGMDPDVLQKSLHTLVKRGKAQVFGSEDQQGVKFF
>CcineVps25
MSLSTHTTPSGSSSVLDFWSSTSDLTQHAHCLGYLLPSIHSAPPFFT
QQPNPSTQGIVVEQWIKLLLSYARYRKLFILRVDDAEEKGSEWDEVLRNERIN
RRVKPAYLETIISTMVKKNQAAYEPPK
QTKAVLLYWRTPEEWAEVLHEWATSTGQLNTIMTFYEI
TDPPVESPLTGIPVQLLRKAIGILGKTGRAQTISISDGEGVRFFAAK
>PchryVps25
MAHVPSKDEVAGTEESIGYLLPSIHSAPPFFTQQPNPNTQAVVTEHWTKLILSYARHRRL
FLLRVEDTEVAGNDWDEIFRNEQIRRRLLPSHLAHIMEDMVTKNKAVYEPARQTRSVLLY
WRTPEEWAEVLHNWADSTGQLNTILTFYEIIEPPVPSQLSGIPMTLLR
KAIAVLTKTSRAQIISVADGEGVRFLAGNTSK
>UmaydVps25
MAEGGSFRYPPIHAFPPFFTLQHNPVSRAQQLS
QWSTLILDYCRHHRLFTISPLAGTSDPASGSVAADPHTSLFANQSIQRSLSAESIRVVLT
HLVDHKQAAWEEQLTGAATKSKRSNANSKAFIYWKTPVQWADSIYDWVMQTGQNKSIMTL
FELNQGDLVQAQDFYLLPTPMLRQALKHLSTQGKAQIFAGTEADDGEGVKFV
>RoryzVps25
MSNFELPSIFDFPPFFTRQVTESTWKSQAFEWESIILSYARHK
HLFRLELHNATTANSGYDIFENKKINRRLSFEALQDIIEEMVKK
AEWEGGPKGSKTEAYLYWHTPEEWANLIWNWINETGQNDQIVTYYEIAHGELAEGQ
EFYDIDHNVLDKALNVLVKRGNAQIFKGTDEDSMGVKFFQ
>BemerVps25
MTSTASLNATPFPFPQLYSFPPFFTLQPHEPSRVKQVAAWLDLIRAYCAHHKLYRL
DMDSAASLDLFKNAAIGRTVPRDMLALLFREAVKAGLAEWSTPKDTTRVLVLWKSVAEWA
AAMVEWARNAGFTDTVMTGYELRFGDYVTDADFYCMDEVLFCRVIQHLAKQKRVVMVNES
KVADELGFKF
>SpurpVps25
MGNFEWPWQYEFPPFFSLQPNLETRKKQLLAWCDLFLAFHKHHRIYTVDLKEAASSELFN
NTKLNRKLSGEGILLVLEELRQKGNIEWTDKAKTRCLVMWRTPEEWGNLIYKWAGNSGMT
NTVCTLYEIAQGEDTTNEEFHGLEDWLLKRSLKCLERGRKAELMAFDGNEGVKFF
>CbrigVps25
MATTSAFKWPWQYDFPPFFTIQKSLNTKDKQLEAWARLVIDYAQHNKIYSLDIAEATTSE
LFNNQKLNRRLSTDGVNTVLQYLEQKKLIEFTDNGRTRFHIFWRRPDVWANMIYQWAVEN
AFLNTPLTLYEITHGDDTTNESFHNLEREILMKALTCLEDQRRAQLMNIGGDNEGVKFV
>CelegVps25
MAAATTTASAFKWPWQYDFPPFFTIQKSLNTKDKQLEAWA
RLVIDYAQHNKIYSLDIAEATTSELFNNQKLNRRLSTDGV
NTVLQYLEQKKLIEFTDNGRTRFHIFWRRPDVWANMIYQW
AVENAFINTPLTLYEITHGDDTTNESFHNLEREILMKALT
CLEEQRRAQLMNIGGDNEGVKFI
>HglycVps25
MSFQWPWHFDFPPFFTIQPNLSTRDKQLKAWGRLLLDYCQANRIYTTDLD
EISKSDLFNNRRLNRHLDLSGIRAVFDFLELQKHVEWKDKDKTRCNIYWRRPEEWGQLLY
EWANSIGLLNTVVTLYELTQGEDVAKESFYGLDKDVLLKGLQHLENQGKAVLIDIDGEKG
GVKFL

>PwestVps25
MSSQKFEWPWLYNFPPFFTLQPNAETRRKQQDAWCQL
VLDYFRHKNVYNISIASIRDSSCSLFHNKSISRSANSDLISNVLDELHRRGNLEWIDKQR
INARIIWRTPEEWADILFRWARDTGHGNSVCTLYELTDGEDTESEPFHGLDNTILLEALR
CLQKRGKAELISDEGVKFLCV
>SjapoVps25
MSLASEKNIWPWQYRFPPFFTLQPNAETRRKQINAWCQLVLDYF
QSKKQFSLSVASIRDPSCPLFNNKSIQRSATPDLIDLVLTELHRRGNLEWVDKSHTNA
RIIWRTPEEWADIIAKWARSTGHGSSVCTFYELTDGDNTKHEAFHGLDFSILTDAINI
LQKRGQAEIMGDAGVKFFC
>SmansVps25
MSLTPGNDVIWPWQYNFPPFFTLQPNAETRRKQINAWCQLVLNYFQSKKQFTLSVASIR
DPSCPLFNNKSIQRSASPELVDLILTELHKRGNLEWLDRSHTNARIIWRTPEEWADLIA
RWARSTGHSNSVCTFYELTDGDDTKQEAFHGLDISVLTNAITVLQKRGQAEIMEDEGVK
FYC
>SmediVps25
MSNFEWPWQYNFPPFFTLQPNAETRKKQIDAWCQLILSYHQNFKQYTLNVKDA
YQSPLFSNKSIDRKVRSDLLTCILDELQKRGNLEWTDKSNVTCKIIWRTTVEWSDLIYKW
IKKSGNVNTICTLYELTDGEETENEPFHGLDKDILISAIRILAKNKRAELLISEDLEGVK
FFQ
>AvariVps25
MTDFEWPWQYSFPPFFTLQPTLATREKQLDAWSNLILNYYRARKEYVLDVAEALASPLFH
NKDISRKLSADALREILKSMSSRSQVAWTDKQQTRCYVFWRSPEEWGKLLYDWADATGHL
NSVCTFYELVQGDDTADTEFAGLDVDLLRLSLQALEKQGKAELITFDGSEGVKVF
>BmicrVps25
MATDFEWPWQYGFPPFFTLQPTLATREKQ
LEAWSNLILNYHRAHKAYVLDVAEALASPLFHNKDISRKLSADSLKEILKYMSSRGQVAW
TDKQQSRCYVYWRSPEEWGKLIHDWADATGHLNTVCTFYELVQGDDTADTEFAGLDVDLL
RLSLQTLEKQGKAELICFDGSDGVKFF
>AaegyVps25
MAEYQWPWEYSFPPFFTVQSHGGTKDQQLSTWKSLILDYQKHSKQAVLNINEDSVPFVNE
AISRKLSPEGRLWVMEALEKTANAAPMDKRKQQWEVYWHTLDEWSSLLHGWAVANGMTNT
VCTLYELVAGDHTVGEEFHGLDQTVLKKALKVLETKGKCELIAFDDSEGVKFY
>AgambVps25
MGAFQWPWEYSFPPFFTVQVHAKTKEQQLATWKELVLNYQKHEGQALLNIAEDAPPFVNR
ELARKLSPEARLWVMEELARTGHAATADKRKQQWEVYWHTLDEWSNILYDWAVASGTTNT
VCTLYELVAGDNTVGEEFHGLDEGVLKKALKLLEGRGKCELIAFDDNEGVKFF
>AmellVps25
MAEIEWPWQYSFPPFFTLQPHSDTRAKQLSAWKSLILEYYRITK
QAIIDVREIHSSPLFNNTAINRKLPSEAVLLLLEELAKSGNASPLDKTKQRWLIYWHT
LEEWGEIIYNWAQENGFCGSVCTLFELTQGEDTIDQEFYGLDTEILIRALKTLETNKK
AELILFDDNQGVKFF
>ApisuVps25
MGDVQWPWQYSFPPFFTIQPNAETRQKQLDAWRTLLLDYCRTQKVSVIDVREGDLLPVFN
NTTISRKLSPDAIMTVLGVLQKTGNAEPLDKTRTRWNVYWHTLDEWASIVYKWAQDNAML
NTVCTFYEIASDSGDLNGIDDGVLTKALRVLERRQQAEILTLDGGSGVKFF
>BmoriVps25
MAEISWPWQYNFPPFFT
IQPHTETRSKQLEAWEQLITDYLKATKQSTIDIREASNTPLFNNIEINRKLSQEAILT
ILEDMAKAGKAAPIDKSKNVWEVYWHSLDEWGNMIYNWACNNGFNNSVCTLFELREGD
NTADQEFHNLDMNVLVKALKSLEAKGRCELMEFDDNQGVKFF
>DmelaVps25
MAEFQWPWEYTFPPFFTLQPHEETRQQQLKVWTDLFLKYLRHTNRFTLSIGDQNSPLFHN
EALKRRLSPELVLAILGELERSGHANPLDKRRQEWQVYWFTLEEYGNMVYDWVQETGQTN
TICTLYEIASGENTSHLDFYGVDEAVLLSALRLLEEKGRCELIEMDGSHGVKFF
>DpseuVps25
MTEFQWPWEYTFPPFFTLQPHEETRQQQLKVWTDLFLKYLKHTNKFSLSINEQSLPLFHN

ESIQRRLSPELILVILEQLQRSGHATALDKRRQEWQVYWYTLEAYGNMVYDWIQETGQTN
SICTLYEIASGESTTQMDFHGVDESVLLNALRLLEEKGRCELIEIDGSHGVKFF
>LlongVps25
MGEFEWPWEYNFPPFFTIQPHDKTREHQLKVWKDLILSYQKHRKEAILTVNANSPLFCND
GIKRQLSPDARLQVMEELQKSGNAAPLGNKVQWEIYWYTLDEWADKIYTWVTNCGLNNT
VCTLYELTSGDNSTSEEFYGLDEGVLRKALGKLQEKRKCELFDDESGGVKFF
>CinteVps25
MGTTSNFKWPWQYDFPPFFTMQKNADTRFKQTEAWC
SLILDYYNHHKLFRLRVTDVLASPLFYNKAIDRRLTKPDVMEILSILHKKGNIEWEDKEK
TVCKVFWKTPAQWSDIIFKWVNQSGLNNTVCTLHEITNGPHSSDQEFYALDDQILMQAIQ
ILQASGKAELMGTEGVKFFT
>MtectVps25
MPQEFAWPWQYDFPPFFT
MQENADTHEKQLNMWCDLVIAYQKYKNQATISIQEANSSPLFNNKQLNRKLQQRDIEKVF
NALCKKGNLEWTDKNKTSCYILWKTVDQWAELIYSWVGRNGMANTVCTLYEITKNENNKN
EEFYELEESLLLRAIKVLELKNKAELIGNDGVKFF
>LerinVps25
MSFEWPWQYNFPPFYTLQPNVSTQHRQLAAWCSLVLSYLRYHKLYTIDVLEAQESPLFNN
KKIQRKFPVEAIQVVLEELRKKGNLEWIDKNKTRCLIMWRRPEEWGKLVYQWVSKNGLTN
SVLTFYELSNGDDTEGEEFHGLEEWLLLRALQTLQSERKAEIITLADSKGVKFF
>DreriVps25
MSFEWPWQYNFPPFFTLQPNVDTRQKQLAAWCSLVLSYCRHRKLYTLDVLEA
QESPVFNNKKIERKLSVEAIQVVFEELRKKGNLEWLDKNKSRCLIMWRRPEEWGKLIYQW
VSKNGMVNSVFTLYELANGDDTEKEEFHGLEDWMLLRSLQALQTDGKAEIITMDDGKGVK
FF
>FrubrVps25
MSFEWPWQYNFPPFFTLQPNVDTRQKQLAAWCSLALSYCRHHKLYTLDVLEAQESPVFNN
KKIERKLSMEAIQVVFEELRKKGNLEWLDKNKSRCLVMWRRPEEWGKLMYQWVSRNGMVN
AVFTLYELSNGDDTEGEEFHGLEEWMLLRSLQALQAEGKAEIITMDDGKGVKFF
>GaculVps25
MSFEWPWQYNFPPFFTLQPNVDTRQKQLAAWCSLALSYCRHHKLYTLDVMEAQESPVFN
NKKMERKLSIEAIQVVFEELRKKGNLEWLDKNKSRCLVMWRRPEEWGKLIHQWVSKNGMV
NSVFTLYELSNGDDTEGEEFHGLEDWMLLRSLQALQTDGKAEIIIMDDGKGVKFF
>IpuncVps25
MSFEWPWQYNFPPFFTLQPNADTRQKQLAAWCSLALSYCRHRKLY
TVDVLEAQESPMFNNKKIERKLSVEAIQVVFEELRKKGNLEWMDKNKTRCLIMWRRPEEW
GKLIYQWVSKNGMVNTVFTLYELSNGDDTESEEFHGLEEWMLLRALQALQADGKAEIITM
DDGKGVKFF
>OlatiVps25
MSFEWPWQYNFPPFFTLQPNADTRQKQLEAWCSLALSYCRHHKLYTLDVMEAQESPMFNN
KKIERKLSMEAIQIVFEELRKKGNLEWLDKNKLRCLVMWRRPEEWGKLIYQWVSKNGMVN
SVFTLYELSNGDDTQGEEFHGLEEWMLLRSLQALQTDGRAEIISMDDGKGVKFF
>OmykiVps25
MSFEWPWQYNFPPFFTLQPNVDTRQKQLAAWCSLALSYCRHHKLYTLDIMEAQESPVFNH
KNIDRKLSMEAILIVFEELRKKGNLEWLDKNKTRCLVMWRRPEEWGKLIYQWVSKNGMVN
TVFTLYELANGDDTESEEFHGLEDWMLIRSLQALQMDGKAEVISMDDGKGVKFF
>PflesVps25
MSFEWPWQYNFPPFFTLQPNVDTRQKQLAAWCSLALSYCRHHKLYTLDIMEAQETPVFNN
KKIERKLSMEAIQVVFEELRKKGNLEWLDKNKSRCLVMWRRPEEWGKLIYQWVSRNGMNN
SVFTLYELVNGDDTEGEEFHGLEDWMLQRSLQALQTDGKAEIITLDDGXGVKFF
>PpromVps25
MSFEWPWQYNFPPFFTLQPNVDTRQKQLSAWCSLALSYCRHRKLYTLDVLEAQESPMFNN
KKIERKLSVEAIQVVFEELRKKGNLEWLDKNKSRCLIMWRRPEEWGKLIYQWVSKNGMVN
SVFTLYELANGDDTEKEEFHGLEDWMLLRSLQALQADGKAEIISMDDGKGVKFF
>SsalaVps25
MSFEWPWQYNFPPFFTLQPNVDTRQKQLAAWCSLALSYCRHHKLYTLDIMEAQESPVFNH
KNIDRKLSMEAILIVFEELRKKGNLEWLDKNKTRCLVMWRRPEEWGKLIYQWVSKNGMVN

TVFTLYELANGDDTESEEFHGLEDWMLIRSLQALQMDGKAEVISMDDGKGVKFF
>TnigrVps25
MSFEWPWQYNFPPFFTLQPNVDTRQKQLAAWCSLALSYCRHHKLYTLDVLEAQESPVFN
NKKIERKLSMEAIQVVFEELRKKGNLEWLDKNKSRCLVMWRRPEEWGKLIYQWVSRNGMV
NSVFTLYELSNGDETEGEEFHGLEEWMLLRSLQALQAEGRAEIITMDDGKGVKFF
>GgallVps25
MSFSWPWQYSFPPFFTLQPNGDTRQKQLAAWCALALAYSRRQRLPAMTLREAQDSPLFAN
RRLQRKLPPEAIQVVLEELRKNGNLEWLDKNKTSFLIMWRRPEEWGKLIYQWVSKNGLTN
SVFTLYELISGDDTANEEFHGLDEAMLLRALQALQQEHKAEIITLDDGRGVKFF
>TguttVps25
MSFAWPWQYSFPPFFTLQPNGETRQKQLSAWCALALAYSQQHRLPAMTVREAQDIPLFANH
RLQRKLPLESIQVVLEELRKNGNLEWLDKNKTSFLIMWKRPEEWGKLIYQWVSKNGLTNS
VFTLYELVSGDDTENEEFHGLDEATLLRALQALQQEHKAEIITLDDGRGVKFF
>XlaevVps25
MGFEWPWQYNFPPFFTLQPNVDTRQKQLSAWSSLVLSYCRQNKL
YTMNLMEIQESPLFNNKKIQRKLSLESVQVVLEELRKKGNLEWIDKNKSRFLIMWRRP
DEWGKVIYQWVSKNGMTNSVFTLYELISGDDTEGEEFHGLDEAMLLRSLEALQQEHKA
EIITLNESRGVKFF
>XtropVps25
MGFEWPWQYNFPPFFTLQPNVDTRQKQLSAWSSLVLSYCRHNKLYTMNLMEIQESPLFNN
KKIQRKLSLESVQVVLEELKKKGNLEWIDKNKSRFLIMWRRPDEWGKVIYQWVSKNGMTN
SVFTLYELISGDDTEGEEFHGLDEAMLLRSLEALQQEHKAEIILLNDSRGVKFF
>BtaurVps25
MAMSFEWPWQYRFPPFFTLQPNVDTRQKQLAAWCSLVLTFCRLHKQSSMTVMEAQESPLF
NNVKLQRKLPVESIQVVLEELRKKGNLEWLDKNKSSFLIMWRRPEEWGKLIYQWVSKSGQ
NNSVFTLYELTNGEDTEDEEFHGLDEATLLRALQALQQEHKAEIITVSDGRGVKFF
>CfamiVps25
MAMSFEWPWQYRFPPFFTLQPNVDTRQKQLAAWCSLVLSFCRLHKQSSMTVMEAQESPLF
NNVKLQRKLPVESIQVVLEELRKKGNLEWLDKNKSSFLIMWRRPEEWGKLIYQWVSRSGQ
NNSVFTLYELTNGEDTEDEEFHGLDEATLLRALQALQQEHKAEIITVSDGRGVKFF
>EcabaVps25
MAMSFEWPWQYRFPPFFTLQPNVDTRQKQLAAWCSLVLSFCRLHKQSSMTVMEAQESPLF
NNVKLQRKLPVESIQVVLEELRKKGNLEWLDKNKSSFLIMWRRPEEWGKLIYQWVSRSGQ
NNSVFTLYELTNGEDTEDEEFHGLDEATLLRALQALQQEHKAEIITVSDGRGVKFF
>HsapiVps25
MAMSFEWPWQYRFPPFFTLQPNVDTRQKQLAAWCSLVLSFCRLHKQSSMTVMEAQESPLFN
NVKLQRKLPVESIQIVLEELRKKGNLEWLDKSKSSFLIMWRRPEEWGKLIYQWVSRSGQN
NSVFTLYELTNGEDTEDEEFHGLDEATLLRALQALQQEHKAEIITVSDGRGVKFF
>MmulaVps25
MAMSFEWPWQYRFPPFFTLQPNVDTRQKQLAAWCSLVLSFCRLHKQSSMTVMEAQESPLF
NNVKLQRKLPVESIQIVLEELRKKGNLEWLDKSKSSFLIMWRRPEEWGKLIYQWVSRSGQ
NNSVFTLYELTNGEDTEDEEFHGLDEATLLRALQALQQEHKAEIITVSDGRGVKFF
>MdomeVps25
MATSFEWPWQYRFPPFFTLQPNVDTRQKQLAAWCSLVLSFCRLHRQSSMTVMEAQESPLF
NNNKLQRKLPMESIQIVLEELRKKGNLEWLDKNKSSFLIMWRRPEEWGKLIYQW
VSKSGQNNSVFTFYELTNGDDTEDEEFHGLDEATLLRALQALQLEHKAEIITVSDGRGVKFF
>MmuscVps25
MAMSFEWPWQYRFPPFFTLQPNVDTRQKQLAAWCSLVLSFCRLHKQSSMTVMEAQESPLFN
NVKLQRKLPVESIQIVLEELRKKGNLEWLDKNKSSFLIMWRRPEEWGKLIYQWVSRSGQN
NSVFTLYELTSGEDTEDEEFHGLDEATLLRALQALQQEHKAEIITVSDGRGVKFF
>OcuniVps25
MAMSFEWPWQYRFPPFFTLQPNVDTRQKQLAAWCSLVLSFCRLHKQSSMTVMEAQESPLF
NNVKLQRKLPMESIQIVLEELRKKGNLEWLDKNKSSFLIMWRRPEEWGKLIYQWVSRSGQ
NNSVFTLYELTNGEDTEDEEFHGLDEATLLRALQALQQEHKAELITISDGRGVKFF
>RnorvVps25
MAMSFEWPWQYRFPPFFTLQPNVDTRQKQLAAWCSLVLSFCRLHKQSSMTVMEAQESPLF
NNVKLQRKLPVESIQIVLEELRKKGNLEWLDKNKSSFLIMWRRPEEWGKLIYQWVSRSGQ

NNSVFTLYELTSGEDTEEEEFHGLDEATLLRALQALQQEHKAEIITVSDGRGVKFF
>SscroVps25
MAMSFEWPWQYRFPPFFTLQPNVDTRQKQLAAWCSLVLSFCRLHKQSSMTVMEAQESPLF
NNVKLQRKLPVESIQVVLEELRKKGNLEWLDKNKSSFLIMWRRPEEWGKLIYQWVSRSGQ
NNSVFTLYELTNGEDTEDEEFHGLDEATLLRALQALQQEHKAEIITVSDGRGVKFF
