# Supplementary material for: Genetic structure and evolution of the Vps25 family, a yeast ESCRT-II component
Source: BMC Evol Biol. 2006 Aug 4;6:59. doi: 10.1186/1471-2148-6-59 (PMC1579232; doi:10.1186/1471-2148-6-59)
Supplement: Additional File 3 — Additional Table 2: Partial Vps25 sequences [file 1471-2148-6-59-S3.pdf]

Additional File 3

Additional Table 2: Partial Vps25 sequences.

Species	mRNA or EST accession number or identifier	Genomic DNA accession number or identifier	Comments
**CHROMALVEOLATES**			
**Alveolates**			
Ciliates			
Tetrahymena thermophila	-	Clone name: 8254385	TIGR clone identifier. Could not determine the carboxy-terminus due to lack of sequence with similarity to known homologs. ATA used as a start codon, by similarity.
Paramecium tetraurelia	-	-	Sequence can be found in reference [36].
**Stramenopiles**			
Thalassiosira pseudonana	-	AAFD01000630	Two nearby regions of similarity found, but full-length gene could not be determined, due to lack of sequence with similarity to known homologs.
**AMOEBOZOA**			
**Amoebidae**			
Hartmannella vermiformis	EST ID: HVE00008892		Missing the first 5-10 amino acids, due to EST starting after the ATG. Protist EST program identifier. Original EST name: pep_rw0305_260703t7_rw_v4mghvdl_0033_rw_v4mghvdl_0033g06.t7m.scf.
**Entamoebidae**			
Entamoeba invadens	-	inv082e03.q1k	Sanger gene database identifier. Genome-survey shotgun sequencing does not go back far enough to reach the first exon and sequence encoding the the amino-terminal 16 amino acids.
Entamoeba moshkovskii	-	mosh036b08.p1k	Sanger gene database identifier. Genome-survey shotgun sequence is quite short lacks sequence encoding both the amino-terminal 26, and carboxy-terminal 70, amino acids.
**PLANTAE**			
**Green algae**			
Chlamydomonas incerta	CIL00001108	-	Missing the first ~6 amino acids, as EST starts after ATG. Searching sequences at the Protist EST Program identifier. Original EST name: pep_cp0082_150304t7_cp_rwlcireg_0029_cp_rwlcireg_0029f05.t7m.scf
**Land plants**			
**Cycadophyta**			
Cycas rumphii	DR062507	-	The only EST match, and sequence does not reach the 3' end (i.e. amino-terminal sequence only).
**Ginkgophyta**			
Ginkgo biloba	DR063699	-	Only EST match. It includes the stop codon, but starts mid-way through the coding sequence. (i.e. carboxy-terminal sequence only.)
**Pinophyta**			

Picea engelmannii x *Picea sitchensis*	DR475258	-	EST sequence corresponding to the first ~20 amino acids is not available.
Picea glauca	DV979402	-	EST sequence corresponding to the first ~18 amino acids is not available.
Pseudotsuga menziesii var. *menziesii*	CN639295	-	EST sequence corresponding to the last ~50 amino acids is not available.
**Magnoliophyta**			
Magnoliids			
Liriodendron tulipifera	CK767577	-	Partial EST corresponding to start codon and 5' end of Vps25 only.
Liliopsida			
Acorales			
Acorus americanus	CO999123	-	Partial EST corresponding to start codon and 5' end only. Contains a few errors near end of sequence run, which have been corrected, by similarity.
Cyperales			
Avena sativa	CN819871	-	EST missing sequence corresponding to first ~75 amino acids missing.
Brachypodium distachyon	DV486221		EST missing sequence corresponding to last ~90 amino acids.
Zea mays	CO458683 and AI920666	-	Missing approximately first 5 amino acids, by similarity. Sequence to stop codon obtained from two overlapping ESTs.
Eudicotyledons			
Asteridae			
Helianthus annuus	CD847190	-	EST is full length, but has too many erros to be included in our full length analyses. For example, there is an ‘X’ in sequence due to 'n' base-call; A stop codon in the coding region should probably be a cysteine; a frameshift error occurs just before the end, due to a read of four 'a's where there should only be three.
Lactuca serriola	BQ986821	-	EST is missing sequence corresponding to last few amino acids only.
Leymus chinensis	CN466260	-	EST sequence only corresponds to the carboxy-terminal half of the protein.
Caryophyllidae			
Beta vulgaris	BQ587202		EST too short to reach 3' end.
**OPISTHOKONTS**			
**Fungi******			
**Ascomycetes**			
Zygosaccharomyces rouxii	-	AL392948	Genomic shotgun sequence. Missing sequence encoding the first~85 amino acids. Sequence present contains no introns.
Pichia augusta	-	AL432282	Amino-terminal sequence only, as genomic shotgun sequence then deteriorates and ends.
Pichia sorbitophila	-	AL415496	Amino-terminal sequence only, with some errors, and then genomic shotgun sequence ends.
Pezizomycotina			
Ajellomyces capsulatus	CV583131	-	EST missing 3' sequence encoding around the last 15 amino acids, and also has a single base-call error.
Aspergillus flavis	CO134982	-	EST missing sequence encoding around first 35 and last 8 amino acids.
Aspergillus nidulans	AA785542	-	EST containing 5' sequence encoding the first 25

(*Emericella nidulans*)			amino acids only.
Chaetomium globosum	-	AAFU01001212	Whole genome shotgun sequence, spliced at least once. Cannot identify a start or stop codon, where expected by similarity. Also maybe a frameshift errorr in the 5' sequence, which we have not corrected, leading to a run of glycines. An extra 'c' in a run of two has caused a frameshift error (by similarity) in the last exon, which has been corrected.
Gibberella moniliformis		AAIM01000453	Whole genome shotgun sequence, but nucleotide 1 of contig is in the middle of the Vps25 gene, so only sequence encoding carboxy-termminal half of the protein was determined. This region is spliced once, using the same intron site as for *Gibberella zeae*.
**Basidiomycete**			
Cryptococcus neoformans var. *neoformans* B-3501A	-	AAEY01000048	Hypothetical protein CNBJ0610. The last exon on the database sequence is predicted to be incorrect, as neither the intron splice donor or acceptor, nor the stop codon, are conserved in other species. However, the carboxy-terminal sequence we propose lacks the highly conserved lysine residue, so we cannot be sure this is the correct alternative at this stage.
**Metazoa**			
**Nematoda**			
Ancylostoma caninum	BQ666448	-	EST sequence missing region encoding the first ~9 amino acids.
Brugia malayi	AW067668		EST sequence predicted to be missing 3' sequence encoding the last ~30 amino acids.
Meloidogyne incognita	BE238910	-	Full length EST, with frameshift error. By similarity, this is probably due to a double 't' being read, and this has been corrected. Also has a glutamine (Q) instead of the conserved lysine (K) near the carboxy-terminus. This is unexpected, and this could be due to a single base-call error. In the closely related *M. hapla*, below, this base is would be that required for a lysine residue, but the next base is uncalled in *M. hapla*. Until this amino acid identity can be resolved, this sequence has not been included in our full-length Vps25 analyses.
Meloidogyne hapla	BM883256	-	EST missing 5' sequence encoding the first ~50 amino acids. Also one uncalled base just before stop codon (putatively coding for the conserved lysine residue).
Trichinella spiralis	BG302232	-	EST sequence good until ~3 amino acids before stop codon.
**Platyhelminthes**			
Cestode			
Echinococcus multilocularis	BU493062	-	EST missing 3' sequence encoding the last ~10 amino acids.
**Arthropoda**			
Crustacea			
Homarus americanus	CN951058	-	EST missing first few amino acids and 3' sequence encoding about the last 26 amino acids.

Penaeus monodon	AW600722	-	EST lacks 3' sequence encoding around the last 80 amino acids, and contains a single nucleotide error creating an unexpected stop codon ('corrected' to an 'X').
Hexapoda			
Anopheles albimanus	DR748047	-	EST is missing 3' sequence encoding around last 20 amino acids
Armigeres subalbatus	AY440285	-	EST is missing sequence encoding approximately first 4 and last 14 amino acids.
Locusta migratoria	CO844251	-	EST contains 5' sequence only corresponding to only around half the predicted cDNA.
Myzus persicae	DW010507	-	EST is lacking 3' sequence encoding the last 4 amino acids.
Tribolium castaneum	DT774906	-	EST is lacking 3' sequence encoding the last 40 amino acids, and also contains some base-call errors.
**Chordata**			
Vertebrata			
Tetrapoda			
Amphibia			
Ambystoma mexicanum	CN033154	-	Full length EST. Does not have the conserved lysine residue. This is probably due to a single base miscall, and there are no additional ESTs with which to compare. Therefore this sequence has not yet been included in our full-length analyses.
Ambystoma tigrinum tigrinum	CN055702	-	Sole EST. Is missing 3' sequence encoding around 13-15 amino acids from carboxy-terminus.
Mammalia			
Dasypus novemcinctus		ARMA:scaffold_7434:7881-11060 Database location: contig_146112	ENSEMBL identifier found using an ENSEMBL BLAST search. A frameshift error occurs and is predicted to be due to a missing 'g' near end of contig. This has been corrected. The sequence is also lacking the last ~60 amino acids, as the contig ends and is followed by a gap on the scaffold.
Echinops telfairi	-	Genomic location scaffold: TENREC:scaffold_267235 6270 to 6392 (-) Database location: contig_399742 713 to 835 (-)	ENSEMBL identifier found using an ENSEMBL BLAST search. There is a gap in middle of the sequence, as the sequence corresponding to two exons is missing on the scaffold. The final ~14 amino acids are also absent, and there is a frameshift mutation, predicted to be due to a missing 'c', just before contig sequence ends, which has been corrected.
Loxodonta africana		Database location: contig_587556 202 to 357 (+) Genomic location: BROADE1: scaffold_56777 4023 to 5406 (+) and Database location: contig_587557 242 to 352 (+) Genomic location:	ENSEMBL identifiers found using an ENSEMBL BLAST search. Missing first ~14 amino acids and sequence in the middle, the former due to a total lack of sequence before the first contig, and a further lack of sequence between the first and second contigs on the scaffold.

		BROADE1:scaffold_56777 7666 to 7776 (+)	
Pan troglodytes	-	NW_118498 and NW_118883	Located in a region syntenic to human *VPS25*on chromosome 17. However, the protein annotated to this region on the genome is not a Vps25 ortholog, but a hypothetical protein, with part of Vps25 fused to a Wnk4-like kinase domain (see XP_511521). This notation is probably due to gaps between contig sequences in this region of the chromosome. For example, NW_118498 is missing the splice acceptor after intron II, and first four amino acids of the next exon, due to a gap between contig sequences. Most, but not all, of the last intron, is then found in NW_118883, which is a short contig unmapped relative to others on chromosome 17.
