# Supplementary material for: Genetic structure and evolution of the Vps25 family, a yeast ESCRT-II component
Source: BMC Evol Biol. 2006 Aug 4;6:59. doi: 10.1186/1471-2148-6-59 (PMC1579232; doi:10.1186/1471-2148-6-59)
Supplement: Additional File 4 — Additional Figure 2: FASTA format of partial Vps25 sequences [file 1471-2148-6-59-S4.pdf]

Additional File 4

Additional Figure 2

FASTA format of partial Vps25 sequences.

A forward slash (/) indicates a gap in Vps25 sequence. Dots (..) at the start or end of the sequence indicate missing amino- or carboxy-terminal sequence. Standard single-letter amino acid abbreviations are used. Genus names are abbreviated to a single letter, while species names are abbreviated to the first four letters. Homologs are in order of taxonomical grouping [Additional File 3].

```
>TtherVps25

MKYQLFKKYLSLLYNFCLSQYYSVIPNKMVQSQAILTFIQ
VYFLNFQLTIFKmIQDHAETRQKQLASWADITHAYFKQKNINNSSLT
ELSSESYPFFCLQNLGIPSKNIIFKLIKILiILW
ENENYDGFQVQKYSANEIADEIYKWAIK
QAQMRVYYFIQNNLNYLYFNSIEFYDLPDNQILYALEVLQ
ENKKCI..
>Ptetr
..DHKETRKKQITQWSEI
VHLYFQSHKILESSISEILNFPIFQDSSSGIIKKLDSSEIKEILNQMAQLGSIEWKNDQN
FTVNLVSPFELADAIYAWAKEKKLIGYTETLRGITEGSQTDQSKKFYNLPQEQILKACLI
LEETGRCQVYEFDGLYSIKFI
>TpseuVps25
MKPPLPTRNNSLQHKTPNLPFFTPTPCTDPPHRQPKSLYHKITI/
PFYCSRIPSLLTFPIQTATTSSRQLPDFYHFPPFFTIQPVLSTREKQLGQWRELILKYRKLMI/
GPTNFLVFLHANTTQCNTQWFNDNTTQHTTTNSGHGEWEDPNQRTRCRILCRKPEQLASDVYE
WAVANGYIGSVCTVYELHSGEWLIRWKF..
>HvermVps25
..PAIYDLPPFYTLQPVLNTREKQLEIWAQFIIDYTKHFRIYELSVPEAAETPLFKNTKINR
RLAPDAIRSCLDYLASKNNGEWLDKDKNRIIIYWKTPEQWANIIHKKVQDNGMIDTVLTL
FELTEGEMVENEEFYKLDSRILMKALNILERRGVAKVFSGAQSADHVGVKFFK
>EmoshVps25
..QLQLWSRLVLLFCSYTKKPVFDKTNFTKLNIFFNDQLN
RLGKLSEEGIVLVKDYMLKHKQIYQPDKSDKFIVLYKPL..
>EinvVps25
..VQPIDRTREYQMELWGQVVTQFCERRSISVVTLADFLSLGIFKNEEINRKLSEDGIKLV
FDYMQRKKQIQQLGQSSKYFVAFKSIVDWANDLLDY
GTTYGLSGSSTTFFSLQNEDTSVFYKCDTTLLLAVCEVLKSKGQNQTSRENGSYG
ILWK
>CinceVps25
..PYFHNYPPYFTLQPVKETRDKQVALWCSLILQYCQQTKTFVLDVQGDSPLFVNKGINRKL
NTEARVVLLDELVAQGRAEWMDKGKTKCLVYWKRVDEWAAVVTEFVRTYGLSDSVMTVDE
LSSGDDVRGTDLYGLHAEVLTRALKLLEAQGKVRTFKGATPEELGVKFI
>CrumpVps25
MGDFKLPYFFNYPPYFTLQPVRDTREKQ
AQVWKELILAYCKHHKIYIVGLEEDFPLFSNPAIDRKLSHEAKEVFLSALISEGRAEWLD
KGHKKCLILWRRIQDWAEFILQFV..
>GbiloVps25 (C-term only)
..KELILNYCKHHKIYVVGLEEDFPLFSNPAIDRKLSHEAKEVFLSGLVSEGRAEWLDKGH
KKCLIVWRRIQDWADYILQFVRENGLEDGVMTLEEIRSGVESRGTELAGIDRIVLVRALK
LLEQRGKAVIFKGISTDDEGVKFSA
>PengeVps25
..SLQPVRDTREKQVQ
LWKELILNYCKHHKIFIVSLEEEFPLFSNPAIDRKLSYEAKKVFLSALVSEGRAEWLDKG
QKNCLILWRRIQDWAEYILKFVRENGLEDGVMTLEEIRSGVESRGTELAGIDRIVLIRAL
KLLEQRGKAVMFKGTSTDDEGVKFSA
>PglauVps25
..FTLQPVRDTREKQVQLWKELILNYCKHHKIFIVSLEEEFPLF
SNPAIDRKLSYEAKKVFLSALVSEGRAEWLDKGQKNCLILWRRIQDWAEYILKFVRENGL
EDGVMTLEEIRSGVESRGTELAGIDRIVLIRALKLLEQRGKAVMFKGTSTDDEGVKFSA
>PmenzVps25
MGDLKLPDFFNYPPYFTLQPVRDTREKQVQ
LWKELILNYCKHHKIYIVSLEEEFPLFSNPAIDRKLSYEAKKVFLSALVSEGRAEWLDKG
QKKCLILWRRIQDWAEYILKFARENGLEDGVMT..
>LtuliVps25 (very N-term only)
MQKLGDFKLPHFFNYPPYFTLQPVRDTREKQVQLWKELILDYCRSQKIF..
>AamerVps25
MQRVGDFKLPNFF
NYPPYFTLQPVRETREKQVQLWKELILDYCRTLKVFTIGLEEDFPLFSNPEIQRTLTHEA
REVFLSALVSEGRAEWINKGHKKCLILWMRIQDWADCVLRLVRDNGLEDSVMTVEAIRSG
IESPGGTELDGIDRTVLMRALKLLEQKGKVALFQ..
>AsatiVps25
..EVFLAALVSEGRAEWIDKSHKKCLILWLRIQDWANYILDFVKENGLEVTTVEDIRS
GIETHGTELAGIDRSVLMRALKLLEQKGKAAIFKGTSADDEGVKFSV
>BdistVps25
MQRPGDF
KLPPFFNYPPYFTLQPVRETREKQVQLWKDLILDYCRTQKIYIVPLEEDFPLFSNSNIER
SLSYEAKEVFLASLVNEGRAE..
>ZmaysVps25
..GGRSCGDAEAGGFQAPPFFN
```

YPPYFTLQPVRETREKQVQLWKDLILDYCRSQKIHTISLEEDFPLFSNAKIERSLSYEAK
EVFLAALVSEGRAEWMDKGHKKCLILWLRIQDWANFILNFVKDNGLEVMTIEEIRSGIDT
RGTELEGIDRGVLMRALRHLKQKGKAAIFKGPSADDEGVKFSV
>HannuVps25
MHTAGEHKLPHFFHYAAYFTLHPVRETREKQIQLWKELILNY**c**RAHKIFVIGLEEDFPLF
SNPAIERSLNHEAREAFLSALVLDGRAEWMDKSHRQCLILWHRIKDWADIILH**X**AKENGL
EDSVMTVEEIRTGIESRGTDIHGMDRGVLMRALKLLGNKGKLAIFKGTSTDDERV**KFSV**
>LserriVps25
MQTLGDHKLPHFFNYPPYFTLQPVRETREKQIQLWKELILDYCRTQKIFIIGLEEDFPLF
SNPAIERSLNHEAREAFLSAIVLDGRAEWMDKSHRQCLILWHRIKDWADLIVRFVKENGL
EDSVMTIEEIRSGIESRGTDIHGMDRTVLVRALKLL**X**TKGKLAIFKGTSTDD..
>LchinVps25
..KNHKKCLILWLRIQDWAKYILDFVKENGLEVTTVEDIRSGIETHGTELAGIDRGVLV
RALRLLEQKGKAVIFKGTSADDEGVKFSA
>BvulgVps25
MQNFGDFKLPNFFNYPPYFTLQPFRDTRDKQIQLWKELILEFCRSQKIFIIALEEDFPLF
SNPTIERSLSHEAREAFLSALVAEGRAEWMDKGH..
>ZrouxVps25
..VPQVFINEIWSTMCQEGKAIGTDKGKKESPTYYILWRTVDSWASLILQWFESAGKLQQVV
TIYELSQGDESIGWEFHGMPEPLTADCLKPLCHRHRATIVNDEYGKPVAVKVV
>PauguVps25
MSFKFPSIYDFPPFFTKQVHGQTLETQKSQWIQLILDYCRYNKIWILSLNGESGDSKLD
HVIQRT..
>PsorbVps25
MDEFQFPXNHFFPPMYTKQPNSTILQNQLDSWSDPNSIILSILQDN
QTITYWQHFILTTRRFKRGQATSVIRQYIHQ..
>AcapsVps25
MHLQRQQHLPFTFPATYSFPP
FFTLQPNTTTRLSQFQKWSALIQAWCRHHRLYRLSLIDAIDSPLFHNADIHKRFILADAR
TVVDWMVRGEGGGRRAEWVGGESGGKAVAWIW**X**RTPEEWASVLADWVEETAHKNTVLTLY
ELMEGEATVLQEFHVMDPDVLSKSLNVLVKRGK..
>AflavVps25
..TRLSQLQKWSLLIQSWCRHHRTYRLSLIEAIESPLFHNSTLRKRIPLSEARNILDWMA
ESEEKGGGGRRAEWVDGTNKTIAWVWWRRPEEWAGILADWVENTGQKNVVLTVYELVEGE
ATMSQEWHGMDVDVMMKSLNVLVKRGKAQVFGSE..
>AniduVps25
MAATIPSQSFHFPPNYSFPPFFSLQPNANTRAVQLQRWSSLIQSWCRHHRI
YKINLVEAIASPLFRNTTLKKQLGLSDARTVLDWMGK..
>CglobVps25
..PRDYSFPPFFTRQTNLTTTTPSLPNGPNLVLAYCRHHRIFKLSLSGRSGTTGTTTTDP
TTTTTITPDTNNGTRDTTALFYNRRLDRGLSHADIREVIDFLRRDGRAEYVSAPTSGTTT
LPIGIPGGAGGAGADGGGGGGGGGGGDMAWIYWRTPEEWASLVEGWVESTGQRGSVLTVY
ELVEGDGTRGSEFHGMDQELLRKALNVLVKQGKAQVFGQEDSLGVKFF
>GmoniVps25
..VVFLYWRKPEEWAELVENYVEETGQKGSVLTVYELVEGDGTKG
NIHGMDTDVLLKALNILVKRNKAQIFGQDDSLGVKFF
>Cneof
MASQPVTFPSQPRREMAPTPLRSHKSASGFEYPAMWSFPPFFT
LQPNPQTLAHQLQLWRRLVLDWSRHERVFEMNTDSTGKDVLEVFENRTIN
RRLLPPSLKTLMGEMVKNGEAAANPPKQDSQYLIYWRKPEEWGDIIYHW
VMDNGLNSSIMTFYEITDGDLSHTTEFHELPISILRKALETLVKRGRAQI
IEGKDEIGEGVRFL
>AcaniVps25
..YDFPPFFTLQQTLVTREKQLETWASLVIDYAQHNKIYTLDVAEIANSELFHNQKLNRRLS
PEGIRAVFDYLEQKKHVEWLDIAKTRCHIYWRRPDEWAALIYAWAVSNGLLNTPCTLYEI
AHGDDTVQESFYGLEKDVLVKALRSLELQRRAQLMNIGTESEGVKFLQ
>BmalaVps25
MSFKWPWQYEFPPFFTLQTTLITREKQLEAWSRLVVDYCQF
HKIYTVDLTDISNSELFVNSALNRKLSLDGVRVVFDYLEHKRHIDWLDKTKNRCHIYWRR
PEEWAILIYEWAVSNSLLNTPCTLYEITQGDDVTQESFYGLDKDVL..
>MincoVps25
MTFQWPWQYDFPPFFTLQPNLQTRDKQLKSWSRLVLDYCQFNKIYSANFEEISHSELFNN
RRHNRRLDDFGIRAVFDHLENLKHIEWCDKQKTRCNIYWRRPEEWGIQIYEWANSIGLLN
SVVTLFELTQGEDAIQESFYGWNKDILSQGLTHLEKQGKAVLIDIDGETGGVQFI
>MhaplVps25
VNFEEISNSELFNNRRLNRRLDDFGIRAVFEYLESLKHIEWCDKQRTRCNIYWRRPEEWG
LQIYEWANSIGLLNSVVTLFELTQGEDAVQESFYGLDKEILLKGLSHLEKQGKAVLIDID
GEKGGVXFI
>TspirVps25
MTFRWPWQYEFPPFFTLQPNLQTRERQLEAWASLVLNYFQANCLHCLDVVDAQQSELFY
NTKIDRKLSLEGIYAVLDRLRQSKHLEWQDKQKQRCLIFWRSPAEWATLIYDWAVRNGFT

```
NTVCTLFELMHGEDSVDEPFHKLNEDVLLEALHQLEVSGQAELILVDSNDKGV..
>EmultVps25
MPSANEEFSWPWQYDFPPFFTL
QPNEETRRKQMDAWCQLVLSYFQSKKLFSVDVSTLMTSQCELFSNAKINRKANPQLIQAI
FDELHRRGNLEWQDRSHKVASIQWRKASSWAADIEKWVRATGRGNTVCTIYEITDGDDTE
GEPFHGLDRAVITEALRYLEKHGKAEI..
>BmoriVps25
MAEISWPWQYNFPPFFT
IQPHTETRSKQLEAWEQLITDYLKATKQSTIDIREASNTPLFNNIEINRKLSQEAILT
ILEDMAKAGKAAPIDKSKNVWEVYWHSLDEWGNMIYNWACNNGFNNSVCTLFELREGD
NTADQ
EFHNLDMNVLVKALKSLEAKGRCELMEFDDNQGVKFF
>HamerVps25
..EFEWPWQYSFPPFFTLQPNADVRRTQLDSWCALVVSWGKSNSVSQIDVTEASSLPVFKNN
AISRALPVDGIKVVLDELAKRGNLEWTDKTRRRGYLLWRSPSEWGQQIYSWAQSTSRINN
VCTLYEINQGDESADQEFHGLDGDILL..
>PmonoVps25
MTDFEWPWQYNFPPFFTLQPNADVRKIQLDAWCALVLGWGKSNNVRQIDVTEASSLPVFK
NSAISRALPADAIAVVLEKLAKRGNLEWTDXSKKKGYLFWKTL..
>AalbVps25
MGAFEWPWEYSFPPFFTVQVQTRTKEIQLGTWKDLVRNYQRHERQALLNITEDTPLFVNE
AISRKLPIDGRLWVMEALEKTGHAAPVDKQKQQWEVYWHTLPEWATLVHEWAVTNGMTNT
VCTLYELVAGDTTVGEEFHGLDQGVFXXILKTLXXRXXCXLIAFXX..
>AsubaVps25
..PWEYNFPPFFTVQSHGNTKEQQLSTWKSLILDYQKHTKQVVLNINEDCAP
FANDAISRKLPPEGRLWVMEALEKSANASPIDKRKQQWEI
YWHTLDEWSTLLHSWAVANGMTNTVCTLYELVAGGHTIGEEFHGLEQAVLKKALKVLESR
GKCELI..
>BmoriVps25
MAEISWPWQYNFPPFFT
IQPHTETRSKQLEAWEQLITDYLKATKQSTIDIREASNTPLFNNIEINRKLSQEAILT
ILEDMAKAGKAAPIDKSKNVWEVYWHSLDEWGNMIYNWACNNGFNNSVCTLFELREGD
NTADQ
EFHNLDMNVLVKALKSLEAKGRCELMEFDDNQGVKFF
>LmigrVps25
MSVENPNIEFPWQFHFPPFF
TLQPHAETRAKQLAAWRSLVLEWYGATLQSKVDLREAGRSPPFANAAINRRLPEEGITAV
MEELARTGHAERLDK..
>MpersVps25
MGEVQWPWQYSFPPFFTIQPNAETRQKQLDAWRTLLLDYCRMQKICVIDVREGDLLPVFN
NTNISRKLSPEAIMTVLGVLQKTGNAEPLDKTRTRWNVYWHTLDEWASIVYKWAQDNAML
NTVYLNGIDDGVLTKALRVLERRQQAEILTLEGGSG..
>TcastVps25
MSTSASEWPWQYHFPPFFTLQPHPETRAKQV
SAWKSLILDYCQKNKLYIVDVREAHQLPLFHNTTMNRKLDPSVIVSILSELAKTKNAAAF
AGACVRWGFCWPSLGVWASLFFVPLCGLGVLACVLPLFVLSLG..
>AmexiVps25
MGFEWPWQYSFPPFFTLQPNVDTRQKQLAAWCSLVLSYCRHNKLYNMTVLEAQESPLFNN
KKIQRKFSMESIQVVLEELRRKGNLEWLDKTKSSFLIMWRRPEEWGKLIYQWVSKNGMTN
TVFTFYELTSGDDTEKEEFYGLDESMLLRSLQHLQLEHKAEVIMLNDSKGGQVF
>AtigrVps25
MGFEWPWQYSFPPFFTLQPNVDTRQKQLAAWCSLVLSYCRHNKLYNMTVLEAQESPLFNN
KKIQRKFSMESIQVVLEELRRKGNLEWLDKTKSSFLIMWRRPEEWGKLIYQWVSKNGMTN
TVFTFYELTSGDDTEKEEFYGLYESMLLRSLQHLPLEHKAEV..
>DnoveVps25
MAMSFEWPWQYRFPPFFT
LQPNVDTRQKQLAAWCSLVLSFCRLHKQSSMTVMEAQESPLFNNVKLQ
RKLPVESIQIVLEELRKK
GNLEWLDKNKSSFLIMWRRPE/EWGKLIYQWV..
>EtelfVps25
MAMGFEWPWQYRFPPFFT
LQPNVDTRQKQLAAWCSLVLSFCRLHKQSCMTVMEAQESPLFNNVKLQ//
VSKSGQNNSVFTLYELTNGEDTEDE
EFHGLDEATLLRALQALQQEHKA..
>LafricVps25
..LQPNVDTRQKQLAAWCSLVLSFCRLHKQSSMTVMEAQESPLFNNVKLQ
RKLPVESIQIVLEELRKKG
GNLEWLDKNKSSFLIMWRRPEEWGKLIYQW//
EFHGLDEATLLRALQALQQEHKAEIITVSDGRGVKFF
>PtrogVps25
MAMSFEWPWQYRFPPFFT
```

LQPNVDTRQKQLAAWCSLVLSFCRLHKQSSMTVMEAQESPLFNNVKLQRILP
LQPNVDTRQKQLAAWCSLVLSFCRLHKQSSMTVMEAQESPLFNNVKLQ/
WLDRAKSAFWIMWRGQRKWGKLIYQW
VSRSGQNNSVFTLYELTNGEDTEDE
/LQALQQEHKAEIITVSDGRGVKFF
