# Supplementary material for: Genetic structure and evolution of the Vps25 family, a yeast ESCRT-II component
Source: BMC Evol Biol. 2006 Aug 4;6:59. doi: 10.1186/1471-2148-6-59 (PMC1579232; doi:10.1186/1471-2148-6-59)
Supplement: Additional File 5 — Additional Figure 3: Alignment used for phylogeny [file 1471-2148-6-59-S5.pdf]

Additional File 5

Additional Figure 3

Alignment used for phylogeny.

PHYLIP format of edited Vps25 amino acid sequences. Nomenclature as in Additional File 2.

```
    119    158
Tannu  NTHVKFKNFP PLYTEQINNL TLSKQLEIWH KIINDEVITN YSLHKIGTET
       PPFKNEEIVR NVNVSFLALI LEYLAEPIQL FCKKFGVFPY PLSEMTNSVL
       ECIKSQCTNI ETIYHIFYSK KEDFNKFPEE NLAFILSKLS VNNQITLSFN
       DKNVGVQL
Tparv  NTHEKFKNFP PLYTEQINNL TLSKQLEIWH KIINDEVTAN YSLHKLGTAS
       PPFKNEEILR NVDVSFLALI LGYLVEPIQF FCKKFGVFPY PLTEMANSVL
       ECIKSQCTTI ETVYHIFYSK REDFNKFPEE NLAFILSYLC VNNKLTLSFN
       DKNVGLQL
Pprim  SSILHVHSLS QVYSLQDHKE TRKKQITQWS EIVHLYFQSH -KILESSISE
       PIFQDSSIIK RLDSSEIKEI LNQMAQLGSI EWKNFSVNLV SPFELADAIY
       AWAKEKKLIT ETLRGITEGS QTKFYNLPQE QILKACLI-L EETGRCQVYE
       F-LYSIKF
Lbraz  SEHWSFFGLP PFFTEQHSPA TLDRQSTLWS NLLLDHAIYH TQRTAGGDTN
       DIFYNPAINK RLSPEGAHTM LQALVAPNHA VVVSVCTTEG GLKGIEENLL
       RYILEHGEVV MTFDELAGGA ALDVGDLSEE QAVRTYLHAL DHHPVSVMRP
       FPYQGVKF
Linfa  SEHWSFFGLP PFFTEQHSPA TLDRQCTLWS NLLLDHAIYH AQRTAGGDTN
       DIFYNPAIDK RLSPEAAHTM LQSLVAPNHA VIVSVCTTED GFKGMEETLL
       RYILDHGEVV MTFDELASGA ALDVGRLSEE QAIRTYLHAV NHRPVSAMRP
       FPYQGVKF
Lmajo  SEHWVFFGLP PFFTEQHSSA TLDRQCTLWS NLLLDHAIYH AQRTAGGDTN
       DIFYNPAINK RLSPEGAHTM LQSLVAPNHV VIVSVCTTED GFKGMEETLL
       RYILDHGEVV MTFDELASGA ALDVGRLSEE QAIRMYLHAL NRRPVSAMRP
       FPYQGVKF
Tbruc  PGHWDFFKLP PFFTLQPSPS ALERQMALWG NLVMDHAAFH AQHRKR-DTC
       GLFRNETINR RLRPEDVKKV ISSLVASEHC VLTSVTTSKG GLKELEQSLL
       AWILERGAGV MTFDELVDGQ CLDVGALSQE QAVRCFLHTL AERPVSHKGL
       FPYEGVKF
Tbruc  PGHWDFFKLP PFFTLQPSPS ALERQMALWG NLVMDHAAFH AQHRKR-DTC
       GLFRNETINR RLRPEDVKKV ISSLVASEHC VLTSVTTSKG GLKELEQSLL
       AWILERGAGV MTFDELVDGQ CLDVGALSQE QAVRCFLHTL AERPVSHKGL
       FPYEGVKF
Tcong  PSHWDFFKLP PFFTLQPAPA ALERQLMLWG NLVMDHAASH APNTRS-DAC
       SLFRNDSINR RLSSEHAKKV LSSLAVPEHC VLTGVSTNKG GLKELEQSLL
       EWILERGVGV MTFNELVDGH CLDVGVLSHE QAVRCFLHTL SVRPISHQGL
       FPYEGVKF
Tcruz  PDHWSFFQLP PFFALQPGPT ALARQTSLWG SAIIDHAAYH APRTRP-GVC
       DVFRNPSLNR RLPPEAAARM LESLVAPNHC AVVLLACNES GLKGLEQALL
       SWILDRGAGV MTFDELAESN CLDVGALSNE QAIRTLLHSL KSRPLSALSP
       IPYEGVKF
Tviva  PNHWNFFKLP PFFTMQPAAA ALARQVTLWE DLIMDHAAHH ARLTNR-GVC
       DVFRNNALRR RLPPEDATKI LSSLASLSHC ISVEVACNEG GLAAIEQSLL
       AWILERGAGV MTFDELVEGQ CLDVGALTEE QAVRTLLKAL ATRPASIRWP
       YPYQGVKF
Glamb  LESSELWYFP PFFTLQQQAD VCTEQLKQWD ALLMKWCYEV -KSREIPKDN
       AIFKNTKINR KVSDELFREI IDWFKRKGRL VEINYYLLSA PEDTIPDSLL
       RLLKNNNFTI ITVLELKKTM DKDLFALPDP VLKKALEA-L QKSGNLALYQ
       EDETGIKI
Tvagi  FEFPDFYDYP PFWTLQTNSQ TKKQQLDLWA SFICAYTKFY -KKTEIDMIQ
       PLFNNQKLGR RVSQQMMTEI IDYMVQSENA KWLEARIIWR TTQQIGDMVR
       QYLDNIGSLM MTYEELINGD ETGFHGLSAD EFHDAMTF-M ESKGRCKIIP
```

```
         GVEYGVKF
Tvagi    FKFPPIYSFP PFWTIQPCME ARRMQTQTWC DLILSWCKAN -KKEDLKVAD
         DLFNNKAIGR ALSQQDAEFF LDQLVQRQNA QWSDCKIIFR KPAQWANIFY
         QWAVKNNLQV FTFYELREGD DTAFHNMDQE QMLKAIEC-L VKEKKANLIK
         ADENGVKF
Ehist    FGIPEFAKFP PFYTIQLVDK TKNQQLQLWS QLILKYCECI KKPIMKQSEF
         PIFHNEELHR TLSENGIELV KEFMVNNNKI IDLNLILLYK PLREWGKELY
         EYGNSKGLIS DTFFSIENDK ESVFYQMDDE LLIEGLNS-I KEQGKMKLVQ
         HGEYGIFW
Ddisc    FQFPPYYHKE PFFTIQPILN TRKKQFQMWQ DLILQYCRYY -KIYELDINE
         VLFNNEKINR KLSREALKSI IDDIIENGFA EWVDVLIMWR KPDEWASLIY
         KWVADCGLLV LTVWEIQNGD DSEFHQLNTT ILMKSLKV-L EKQSKCQTFS
         QENVGVKF
Crein    FAFPYFHNYP PYFTLQPVKE TRDKQVALWC SLVLQYCQHT -KTFVLDVQG
         PLFVNKVINR KLNQEARVAI LDELATQGRA EWMDCLVYWR RVDEWAGAVT
         EFVRTFGLSV MTVDELSSGD DVDLYGVHPE ILTRALKL-L EAQGKVRTFK
         GEELGVKF
Cmero    FAFPPWHQYP PLYTIQPCAR TRERQLYLWR RLILDYCEHF -WILTLRLYD
         PLFCNRAIQR RLSRSALQCI FQELVLSGDA AWSNLLIFWR SPRLWADELL
         RVVAQYGQNV FTLPELASML GELQTAIPIA FLEHILEL-L AAQGKARVFS
         -EGRGVKF
Ppate    FSFPYFYNYP PYFTLQPMKD TRDKQIQLWK ELILKYCKHH -KLFLIDLEE
         ALFENTSIQR KLTFEAREQF LSALIADGRA EWLDCLILWR RIEDWADSLL
         NFVRENGIEL MTLEEIISGD ETELAGLDRG VLVRAVKI-L EQRGKAAMFK
         GDDDGVKF
Wmira    FRLPDFFNYP PYFTLQPVRD TRQKQVQLWK ELIVNYCKHH -KTYIINLEE
         PLFSNPAINR KLSHEAKEAF LSALVNEGKA EWLDCLILWR RIEDWAAYIL
         HFVHENGLEV MTLEELQTGV ESDLAGIDRT VLVKALRL-L EQRGKAVIFK
         GDDEGVKF
Ptaed    LKLPDFFNYP PYFTLQPVRD TREKQVQVWK ELILNYCKHH -KIFIISLEE
         PLFSNPAIDR KLSYEAKKVF LSALVSEGRA EWLDCLILWR RIQDWAEYIL
         KFVHENGLEV MTLEEIRAGV ESELAGIDRI VLIRALKL-L EQRGKAVIFK
         GDDEGVKF
Aoffi    FRLPHFFNYP PYFTLQPVRE TREKQVQLWK ELILDYCRSQ -KIFIIGLEE
         PLFSNPVIER SLNHEARGVF LSALVSEGRA EWMDCLILWL RIQDWADHII
         KFVKDNGLEV VTVEDIRSGF ESELAGIDRG VLMRALRL-L EQKGKAAIFK
         GDDEGVKF
Hvulg    FKLPHFFNYP PYFTLQPVRE TREKQVQLWK ELILDYCRSQ -KMYIISLEE
         PLFSNQKIER SLSYEAKEVF LAALVSEGRA EWIDCLILWL RIQDWANYIL
         DFVKENGLEV TTIEDLRSGI ETELAGIDRG VLMRALRL-L EQKGKAVIFK
         GDDEGVKF
Osati    FRLPPFFNYP PYFTLQPVRE TREKQVQLWK DLILDYCRSQ -KLYIISLEE
         PLFSNPKIER SLSHEAKEVF LAALVYEGRA EWMDCLILWL RIQDWANYIL
         NFVKDNGLEV MTVEEIRSGI ETELAGIDRG VLMRALKL-L EQKGKAAIFK
         GDDEGVKF
Sbico    FRLPPFFNYP PYFTLQPVRE TREKQVQLWK DLILDYCRSQ -KIHTISLEE
         PLFSNPKIER SLSHEAKEVF LAALVSEGRA EWMDCLILWL RIQDWANFIL
         NFVKDNGLEV MTIEEIRSGI DTELEGIDRG VLMRALRQ-L EQKGKAAIFK
         GDDEGVKF
Soffi    FRLPPFFNYP PYFTLQPVRE TREKQVQLWK DLILDYCRSQ -KIHTISLEE
         PLFSNPKIER SLSHEAKEVF LAALVSEGRA EWMDCLILWL RIQDWANFIL
         NFVKDNGLEV MTIEEIRSGI DTELEGIDRG VLMRALRQ-L EQKGKATIFK
         GDDEGVKF
Taest    FKLPHFFNYP PYFTLQPVRE TREKQVQLWK ELILDYCRSQ -KMYIISLEE
         PLFSNPKIER SLSYEAKEVF LAALVSEGRA EWIDCLILWL RIQDWANYIL
         HFVKENGLEV TTIEDIRSGI ETELAGIDRG VLMRALRL-L EQKGKAVIFK
         GDDEGVKF
```

```
Amaju  FKLPHFFNYP PYFTLQPVRD TREKQIQLWK ELILDYCRTQ -KIFVIGLDE
       SLFTNSVIER SLSHEASEAF LSALVLEGRA EWLDCLLLWH RIQDWADLIL
       SFAKDNGLEV MTVEEIRSGI ESELHGMDRT ILMRALKH-L EHKGKLAIFK
       GDDEGIKF
Ccane  FKLPHFFNYP PYFTLQPVRD TREKQVQLWK ELIIEYCRAQ -KVFIIGLEE
       PLFSNHAIER SLSHEARVAF LSALVSDGRA EWTDCLILWH RIQEWADLIV
       HFVKENGLEV TTVEEIRSGT ESELYGIDRS VLMRALKL-L EHKGKLVIFK
       GDDEGVKF
Lescu  FKLPNFFNYP PYFTLQPVRE TREKQIQLWK ELIIDFCRTQ -KIFVIALEV
       PLFSNAAIER SLSHEAREAF LSALVSDGRA EWMDCLVLWH RIQDWADLIV
       NFVKENGLEV MTVEEIRSGV ESELHGIDRT VLMRALKV-L EHKGKLAIFK
       GDDEGVKF
Athal  FKLPQFFNYP PYFTLQPVRD TREKQIQLWK ELILDYCKSQ -KIFLIGVEE
       PLFSNSAIDR SLSHEARETF LSAIVGEGRA EWLDCLILWH RIQDWADIVL
       QFVRDNGLEV MTVEEIRSGT ESELQGIDRT ILMRALKL-L ENKGKLALFK
       GDDEGVKF
Bnapu  FKLPQFFNYP PYFTLQPVRD TREKQIQLWK ELILDYCKSQ -KVFLIGVEK
       PLFSNSAIDR TLSHEAREIF LSAIVGKGRA EWLNCLILWH RIQNWADIIL
       KFVRENGLKV MTVEKIRSGT ESELEGIDRT ILMRALKL-L ENKGKLALFK
       GDDEGVKF
Cclem  FKLPAFFNYP PYFTLQPVRD TREKQIQLWK ELILDYCRTQ -KVFLILLEE
       PLFSNPVIER SLNNEARETF LSALVSEGRA EWLDCLILWH RIQDWADIIL
       GFVKDNGLEV MTVEEIRLGI ESELHGMDRT ILMRALKL-L EHKGKVAIFK
       GDDEGVKF
Fvesc  YKLPQFFNYP PYFTLQPVRD TREKQIQLWK DLILDYCRTQ -KIFVIALEE
       PLFSNPAIER SLSHEAREAF LSILVSDGRA EWLDCLILWH RIQEWANIIL
       NFVKEYGLEV MTVEEIRSGI ESELHGIDRT ILMRALKL-L EQKGKLAIFK
       GDDEGVKF
Ghirs  FKLPHFFNYP PYFTLQPVRE TREKQIQLWK ELILDYCRTQ -KIFVIRLEE
       PLFSNSVIER TLSHEAREAF LSALVAEGRA EWLDCLILWH RIQEWADIIV
       NFVKDNGFEV MTVEEIRSGI ESELQGIDRT ILVRALKL-L EHKGKLAIFK
       GDDEGVKF
GmaxV  FKLPPFFNYP PYFTLQPVRD TREKQIQLWK DLILDFCKTQ -KIFIIGLEE
       PLFTNHGIER SLTHEAREAF LSALVSEGRA EWMDCLILWH RIQDWADILI
       QFAKDNGLEV VTIEEIRSGT ESDLHGIDRT ILNRALKL-L EQKGKLVVFK
       GDDEGVKF
Malus  FKLPQFFNYP PYFTLQPVRD TREKQIQLWK DLILDYCKTH -HIFVIGLED
       PLFSNPAIER SLTYEAREAF LSALVAQGRA EWLDCLVLWH RIQDWANIVL
       DFVKDNGLEV MTVEEIRTGI ESELHGIDRT VLMRALKM-L EQKGKLAIFK
       GDDEGLKF
Mtrun  FKLPHFFNYP PYFTLQPVRD TREKQIQLWK ELILDYCKTQ -KLFVIALEE
       PLFTNTVIER SLTNEAREAF LSALVSEGRA EWMDCLILWH RIQDWADILL
       QFAKDNGLEV VTIEEIRFGT ESELHGIDRT ILNRALKL-L EQKGKLVVFK
       GDDEGIKF
Ptric  FKLPQFFNYP PYFTLQPVRD TREKQVQLWK ELILDYCRTQ -KIFVIGLEE
       PLFSNHLIER SLSNEAREAF LSALVSEGRA EWLDCLILWH RIQDWADILL
       HFVRDNGFEV MTVEEIRTGV ESELHGIDRT ILMRALKL-L EHKGKLAIFK
       GDDEGVKF
Ptrem  FKLPQFFNYP PYFTLQPVRD TREKQVQLWK ELILDYCRTQ -KIFVIGLEE
       PLFSNHLIER SLSNEAREAF LLALVSEGRA EWLDCLILWH RIQDWADILL
       HFVRDNGFEV MTVEEIRTGV ESELHGIDRI ILMRALKL-L EHKGKLAIFK
       GDDEGVKF
Vvini  LKLPNFFNYP PYFTLQPVRD TREKQVQLWK ELILDYCRTQ -KIFVIGLEE
       PLFSNPVIER TLSHEAKEAF LSALVSEGRA EWMDCLILWH RIRDWADLIL
       RFVRENGLEV MTVEEIRSGT ESELHGMDRT VLMRALKQ-L EHKGKLAIFK
       GDDEGVKF
Calbi  FEFPKIYSFP PFYTQQPNTT VLNQQLDSWV SIILHYCEYY -RITSLSIEG
```

```
       SIFINKTINR QVNSDFQKLI VKHLIHNKKA EFINVYIYWR SLVDWGDLLY
       QYVDDTGQKV LTIYELTKSE ETDLHNLDET FLVKIIKDYL IKQGKAQLLI
       DEIGGVKI
Cglab  ---PSIYAFP PLYTRQPNSL VRKQQIDTWI DILTEWCKSH -RVFELGKDG
       SLFKNEEINR AVPPLFIDEI WSVMATRGVA LVTEYYVLWR TLDSWASLIL
       QWFETVGKLV VTLYELTESD ETEFHSMPLP LLHRCLKP-L CNRNRATLMK
       DTPVALKV
Clusi  FEFPKIHSFP PFYTKQRNAT ILENQLEAWG ALILDYCEHF -RIFSILDSG
       PLFENKLIER AVLDEFRKEI FAHLVSLGRA AYVDILLFWR TPAEWAALIR
       EHVESTGQLV LTVYELTKLE ETLLRNIDYN LFVRAIDV-L MKQGKAQVLK
       AQIGGVKI
Dhans  FEFPKIHSFP PLYTKQPNLT ILHNQLESWG EIILSYCQHY -KITSLTLGG
       PLFENKNINR AVNDDFKAMI FKHLIHLHRA EYINILIYWK TLIEWANILH
       DFVERTGQLV LTIYELTKLE DSDLKDLDYN LLVRILKGVL IKQGRAQILM
       NQIGGVKI
Egoss  SELPSIYNFP PLYTRQPNSL IRAQQLDAWL GVLKDYARGR -RVWIMGHDG
       SVFFNVQLQR RVPGPFVDEI WAHALAKGAA LRHSFYVLWR SLDSWSALLL
       QWFETCGRLV VTIYELVAGD ESEFHGMHDG LCELALGK-L VERGRATLIS
       NSVVAVKV
Klact  LETPQIYKFP PLYTPQTNKL IRKQQLQTWE SIILQTCAQL -SKWCINKNG
       SIFENPEIQR ACSLEFQQEI WAYMLQNETA LQLKIAIFWE SLDSWSSTIL
       EWCETSGKLV ITIYEICESD ENKFYGMPSS FCLLVLHR-L VDRNRATLLK
       DKIVGVKI
Kwalt  EKLPPIYNFP PLYTLQPNVL IREQQLNTWC DLILEFAKTT -AAWCMSQEG
       SIFRNESIQR AVPAPFIEQI WSKMVQTEKA LKLNYLILWK SVDHWSSQIL
       QWFETSGKLV VTLYELLEGD ETEFHGMHSS VCEVCIQR-L CDRGRATLLK
       EKIMGLKV
Scere  SALPPVYSFP PLYTRQPNSL TRRQQISTWI DIISQYCKTK -KIWYMSVDG
       NLFNNEDIQR SVSQVFIDEI WSQMTKEGKC LPIDYFILWK SLDSWASLIL
       QWFEDSGKLV ITLYELSEGD ETEFHRMPES LLYYCLKP-L CDRNRATMLK
       DKVIAIKV
Sbaya  ASLPPVYSFP PLYTRQPNSL TRRQQLSTWI DIISQYCKSK -KIWYMSADG
       NLFNNEEIQR SVPQVFIDEI WSQMVKEGKC LPIDYFILWK NLDNWASLIL
       QWFEDSGKLV ITLYELSESD ETEFHGMPEN LLYYSLKP-L CDRNRATMLK
       DKVIAIKV
Scast  QPLPPIYSFP PLYTRQPNSI IRNQQLNAWI DLILQYAREN -KCWTMAKTG
       NIFHNESIQR SVSPMFIDEI WALMIKSKAI VNNDYFILWQ NIDSWASLIL
       QWFEDSNKLV VTIYELSQGD ETEFHQMPEP LLILALKP-L CKRNRATMLK
       DAPIAIKV
Skluy  KQLPPIYSFP PLYTRQPNAL IRQQQLDTWC DLLVQYAKQR -RAWCMGSDG
       SIFLNSQIQR TVPSPFVEEI WARMLQSGKV IKRQYYVLWK DLDSWSSNIL
       QWFETTGKLV VTIYELLEGD ESEFRGMNPE LCEMCLEK-L CSRGRATTLK
       EKIMGVKV
Skudr  ASLPPVYSFP PLYTRQPNSL TRRQQISTWI DIISQYCKGK -KIWYMSADG
       NLFNNEDIQR SVSQVFIDEI WSRMVKEGKC LPIDYFIMWK SLDNWASLIL
       QWFEDSGKLV VTLYELSEAD ETEFHGMPEN LMYYCLKP-L CDRNRATMLK
       DKVIAIKV
Smiki  ASLPPIYSFP PLYTRQPNSL TRRQQISTWI DIISQYCKSK -RTWYMSADG
       NLFNNEDIQR SVPQEFIGEI WLQMAKEGKC LPIDYFVLWK SVDNWASLIL
       QWFEDCGKLV VTLYELSQGD ETEFHGMPEN LLYYCLKP-L CDRNRATMLK
       DKVIAIKV
Spara  TALPPVYSFP PLYTRQPNSL TRRQQISTWI DIISQYCKCK -KIWYMSADG
       NLFNNEDIQR SVSQVFIDEI WSQMAKEGKC LPIDYFILWK SLDNWASLIL
       QWFEDSGKLV ITLYELSEGD ETEFHGMPEN LLYYCLKP-L CDRNRASMLK
       DKVIAIKV
Ylipo  --NSEIYNFP PFFTRQPNET TWQAQLSHWK DVILTHSRET -KQWRLSN--
       SIFENKKIQR RLKPDVIQLV LSDLVSNKKA DWVDVWIWWR SAEEWATLIL
```

```
      AWIDSTGQNI VTFYDIAGDD SPEMVGMDST MLHKVCQV-L VHQGKAAVMR
      DNEVGLKV
Spomb -RVPSIYNFP PFFTRQLNDN TWHSQKAAWQ MWILLWCREN -RQTSITINP
      SLLHNSTIHR TLPLSVFREI VEDMVKQNLA EWTEFWVYWR SISEWGNMIL
      KWLSDMGREI CTFYELQEQY K-EVDCLDEV LLHKVLEL-L MKKGNIELMK
      GKYSGFKV
Afumi FQFPPTYSFP PFFTRQPNST TRLSQLQKWS SLIQSWCRHH -RIYRLSLIE
      PLFHNATLRK RLSLSEARAV LDWMAKGRRA EWIDAWIWWR RPEEWAGIVA
      DWVEATGQKV LTVYELLEGE ATEWHGMDAD VMLKSLNI-L VKRGKAQVFG
      SGQEGVKF
Aoryz FQFPPTYSFP PFFTPQPNST TRLSQLQKWS LLIQSWCRHH -RTYRLSLIE
      PLFHNSTLRK RIPLSEARNI LDWMAEGRRA EWVDAWVWWR RPEEWAGILA
      DWVENTGQKV LTVYELVEGE ATEWHGMDVD VMMKSLNV-L VKRGKAQVFG
      SGQEGVKF
Bfuck FKFPREHSFP PFFTLQPTSS TVHAQLRKWS DLILSYFAFH -RLFRLTVST
      ELFKNERINR RLDEEGLREV LEFMRKEGRV EWIGCWVWWR KVDEWARVIE
      DWVDETGQRV LTLYELVEGE GGEFHGLDTE ILQKALAI-L VKRGKAQVFG
      QDQQGVKF
Cimmi FPFPAPHSFP PFFTLQPNAQ TLLSQLQKWS ALIQAYCRHH -RLYRLSLVD
      PLFHNKQIRK RLSLVDARRI VDWMCGGRRA EWVGAWIWWR RPEEWAGVIA
      DWVEETAQKV LTLYELTEGE ATEFHGMDPD VLQKSLHV-L VKRGKAQVFG
      NDQQGVKF
Gzeae FKFPREYHFP AFFTRQTNLT TLHAQHNKWA DLILAYARHN -RIFRLSLSE
      DLFVNRKLDR RLQFDDIRDV VSFMHTDGRV EYVGVFLYWR KPEEWAELVE
      NYVEESGQKV LTVYELVEGD GTDIHGMDTD VLLKALNV-L VKRNKAQIFG
      QDSLGVKF
Mgris FPFPREYHFP PFFTRQTNLT THHAQLTKWS ALVLAYCRHH -RIFKLPLTT
      ELFHNKTLNR RLSPADVREV IDFMCKEGRA EYCSAWIYWR SPEEWAQLIE
      GWVDETAQKV LTLYELVEGD GTDIHGIDRD MLHRALQL-L VKRGRAQIFG
      QDSQGVKF
Nfisc FQFPPTYSFP PFFTPQPNST TRLSQLQKWS SLIQSWCRHY -RIYRLSLIE
      PLFHNATLRK RLSLSEARAV LDWMAKGRRA EWIDAWIWWR RPEEWAGIVA
      DWVEATGQKV LTVYELLDGE ATEWHGMDAD VMLKSLNV-L VKRGKAQVFG
      SGQEGVKF
Ncras FPFPREFFFP PFFTRQTNLT THHAQLTKWS SLLLAYCRHH -RLFRLSLN-
      LPFHNPRINR RLAPGDIRRV VDFLRRDGRA EYVLAFIYWR TPEEWGSLIE
      GWVEETGQRV LTVYELREGE GTEIWGMDGD VLVKALGT-V VKRGKAQIFQ
      SDSLGVKF
Pnodo FQFPPHYSFP PFFTLQPTAS TRSSQLLSWS TLIQSYCRHH -RIFTLSLID
      PLFNNTALRR RLSLRDAKTI LTWMSTGNRV EFINCWIFWR RPEEWSAVLE
      EWVDRTGQKV LTLYEIVEGD ASEFWGMDLE LLMRSLGV-S VKRGKAQIFG
      GGSEGVKF
Sscle FKFPREHSFP PFFTLQPTSA TLHAQLRKWS DLILSYFAFY -RLFRLTIST
      ELFKNERINR RLDEEALREV LEFMRKEGRV EWIGCWVWWR KVDEWARIIE
      EWVDETGQRV LTLYELVEGE GGEFHGLDAE ILQKALAI-L VKKGKAQVFG
      QDQQGVKF
Trees FAFPREYSFP PFFTRQPNIA IHHAQLTKWS ALVLSYARHH -RLFRLVVSS
      ELFHNRAINR RLGPADIREV LDFMRKDGRA EFIRVLLYWR KPEEWAALVE
      AYVDETAQKV LTVYELTEGE NTEFHGMDNL VLMKALNI-L VKQGKAQIFG
      SDSLGVKF
Urees FAFPAPHSFP PFFTLQPNTQ TLLSQLQKWS ALIQSYCRHH -RLYRLSLVD
      ALFHNRTIRR RLSLADARKV LDWMCSGRRA EWVGAWIWWR RPEEWAGVIA
      DWVEETAQKV LTLYELIEGE ATEFHGMDPD VLQKSLHT-L VKRGKAQVFG
      SDQQGVKF
Ccine YLLPSIHSAP PFFTQQPNPS TQGIVVEQWI KLLLSYARYR -KLFILRVDD
      EVLRNERINR RVKPAYLETI ISTMVKKNQA AYEPVLLYWR TPEEWAEVLH
      EWATSTGQLI MTFYEITDPP VEPLTGIPVQ LLRKAIGI-L GKTGRAQTIS
```

```
       I-GEGVRF
Pchry  YLLPSIHSAP PFFTQQPNPN TQAVVTEHWT KLILSYARHR -RLFLLRVED
       EIFRNEQIRR RLLPSHLAHI MEDMVTKNKA VYEPVLLYWR TPEEWAEVLH
       NWADSTGQLI LTFYEIIEPP VPQLSGIPMT LLRKAIAV-L TKTSRAQIIS
       V-GEGVRF
Umayd  FRYPPIHAFP PFFTLQHNPV SRAQQLSQWS TLILDYCRHH -RLFTISPLA
       SLFANQSIQR SLSAESIRVV LTHLVDHKQA AWEEAFIYWK TPVQWADSIY
       DWVMQTGQNI MTLFELNQGD LVDFYLLPTP MLRQALKH-L STQGKAQIFA
       GDGEGVKF
Roryz  FELPSIFDFP PFFTRQVTES TWKSQAFEWE SIILSYARHK -HLFRLELHN
       DIFENKKINR RLSFEALQDI IEEMVKKAEW EGGPAYLYWH TPEEWANLIW
       NWINETGQNI VTYYEIAHGE LAEFYDIDHN VLDKALNV-L VKRGNAQIFK
       GDSMGVKF
Bemer  FPFPQLYSFP PFFTLQPHEP SRVKQVAAWL DLIRAYCAHH -KLYRLDMDS
       DLFKNAAIGR TVPRDMLALL FREAVKAGLA EWSTVLVLWK SVAEWAAAMV
       EWARNAGFTV MTGYELRFGD YVDFYCMDEV LFCRVIQH-L AKQKRVVMVN
       EDELGFKF
Spurp  FEWPWQYEFP PFFSLQPNLE TRKKQLLAWC DLFLAFHKHH -RIYTVDLKE
       ELFNNTKLNR KLSGEGILLV LEELRQKGNI EWTDCLVMWR TPEEWGNLIY
       KWAGNSGMTV CTLYEIAQGE DTEFHGLEDW LLKRSLKC-L ERGRKAELMA
       FGNEGVKF
Cbrig  FKWPWQYDFP PFFTIQKSLN TKDKQLEAWA RLVIDYAQHN -KIYSLDIAE
       ELFNNQKLNR RLSTDGVNTV LQYLEQKKLI EFTDFHIFWR RPDVWANMIY
       QWAVENAFLP LTLYEITHGD DTSFHNLERE ILMKALTC-L EDQRRAQLMN
       IDNEGVKF
Celeg  FKWPWQYDFP PFFTIQKSLN TKDKQLEAWA RLVIDYAQHN -KIYSLDIAE
       ELFNNQKLNR RLSTDGVNTV LQYLEQKKLI EFTDFHIFWR RPDVWANMIY
       QWAVENAFIP LTLYEITHGD DTSFHNLERE ILMKALTC-L EEQRRAQLMN
       IDNEGVKF
Hglyc  FQWPWHFDFP PFFTIQPNLS TRDKQLKAWG RLLLDYCQAN -RIYTTDLDE
       DLFNNRRLNR HLDLSGIRAV FDFLELQKHV EWKDCNIYWR RPEEWGQLLY
       EWANSIGLLV VTLYELTQGE DVSFYGLDKD VLLKGLQH-L ENQGKAVLID
       IEKGGVKF
Pwest  FEWPWLYNFP PFFTLQPNAE TRRKQQDAWC QLVLDYFRHK -NVYNISIAS
       SLFHNKSISR SANSDLISNV LDELHRRGNL EWIDARIIWR TPEEWADILF
       RWARDTGHGV CTLYELTDGE DTPFHGLDNT ILLEALRC-L QKRGKAELIS
       D--EGVKF
Sjapo  NIWPWQYRFP PFFTLQPNAE TRRKQINAWC QLVLDYFQSK -KQFSLSVAS
       PLFNNKSIQR SATPDLIDLV LTELHRRGNL EWVDARIIWR TPEEWADIIA
       KWARSTGHGV CTFYELTDGD NTAFHGLDFS ILTDAINI-L QKRGQAEIMG
       D--AGVKF
Smans  VIWPWQYNFP PFFTLQPNAE TRRKQINAWC QLVLNYFQSK -KQFTLSVAS
       PLFNNKSIQR SASPELVDLI LTELHKRGNL EWLDARIIWR TPEEWADLIA
       RWARSTGHSV CTFYELTDGD DTAFHGLDIS VLTNAITV-L QKRGQAEIME
       D--EGVKF
Smedi  FEWPWQYNFP PFFTLQPNAE TRKKQIDAWC QLILSYHQNF -KQYTLNVKD
       PLFSNKSIDR KVRSDLLTCI LDELQKRGNL EWTDCKIIWR TTVEWSDLIY
       KWIKKSGNVI CTLYELTDGE ETPFHGLDKD ILISAIRI-L AKNKRAELLI
       SDLEGVKF
Avari  FEWPWQYSFP PFFTLQPTLA TREKQLDAWS NLILNYYRAR -KEYVLDVAE
       PLFHNKDISR KLSADALREI LKSMSSRSQV AWTDCYVFWR SPEEWGKLLY
       DWADATGHLV CTFYELVQGD DTEFAGLDVD LLRLSLQA-L EKQGKAELIT
       FGSEGVKV
Bmicr  FEWPWQYGFP PFFTLQPTLA TREKQLEAWS NLILNYHRAH -KAYVLDVAE
       PLFHNKDISR KLSADSLKEI LKYMSSRGQV AWTDCYVYWR SPEEWGKLIH
       DWADATGHLV CTFYELVQGD DTEFAGLDVD LLRLSLQT-L EKQGKAELIC
       FGSDGVKF
```

```
Aaegy  YQWPWEYSFP PFFTVQSHGG TKDQQLSTWK SLILDYQKHS -KQAVLNINE
       VPFVNEAISR KLSPEGRLWV MEALEKTANA APMDWEVYWH TLDEWSSLLH
       GWAVANGMTV CTLYELVAGD HTEFHGLDQT VLKKALKV-L ETKGKCELIA
       FDSEGVKF
Agamb  FQWPWEYSFP PFFTVQVHAK TKEQQLATWK ELVLNYQKHE -GQALLNIAE
       PPFVNRELAR KLSPEARLWV MEELARTGHA ATADWEVYWH TLDEWSNILY
       DWAVASGTTV CTLYELVAGD NTEFHGLDEG VLKKALKL-L EGRGKCELIA
       FDNEGVKF
Amell  IEWPWQYSFP PFFTLQPHSD TRAKQLSAWK SLILEYYRIT -KQAIIDVRE
       PLFNNTAINR KLPSEAVLLL LEELAKSGNA SPLDWLIYWH TLEEWGEIIY
       NWAQENGFCV CTLFELTQGE DTEFYGLDTE ILIRALKT-L ETNKKAELIL
       FDNQGVKF
Apisu  VQWPWQYSFP PFFTIQPNAE TRQKQLDAWR TLLLDYCRTQ -KVSVIDVRE
       PVFNNTTISR KLSPDAIMTV LGVLQKTGNA EPLDWNVYWH TLDEWASIVY
       KWAQDNAMLV CTFYEIASDS G-DLNGIDDG VLTKALRV-L ERRQQAEILT
       LGGSGVKF
Bmori  ISWPWQYNFP PFFTIQPHTE TRSKQLEAWE QLITDYLKAT -KQSTIDIRE
       PLFNNIEINR KLSQEAILTI LEDMAKAGKA APIDWEVYWH SLDEWGNMIY
       NWACNNGFNV CTLFELREGD NTEFHNLDMN VLVKALKS-L EAKGRCELME
       FDNQGVKF
Dmela  FQWPWEYTFP PFFTLQPHEE TRQQQLKVWT DLFLKYLRHT -NRFTLSIGD
       PLFHNEALKR RLSPELVLAI LGELERSGHA NPLDWQVYWF TLEEYGNMVY
       DWVQETGQTI CTLYEIASGE NTDFYGVDEA VLLSALRL-L EEKGRCELIE
       MGSHGVKF
Dpseu  FQWPWEYTFP PFFTLQPHEE TRQQQLKVWT DLFLKYLKHT -NKFSLSINE
       PLFHNESIQR RLSPELILVI LEQLQRSGHA TALDWQVYWY TLEAYGNMVY
       DWIQETGQTI CTLYEIASGE STDFHGVDES VLLNALRL-L EEKGRCELIE
       IGSHGVKF
Llong  FEWPWEYNFP PFFTIQPHDK TREHQLKVWK DLILSYQKHR -KEAILTVNA
       PLFCNDGIKR QLSPDARLQV MEELQKSGNA APLGWEIYWY TLDEWADKIY
       TWVTNCGLNV CTLYELTSGD NSEFYGLDEG VLRKALGK-L QEKRKCELFD
       -ESGGVKF
Cinte  FKWPWQYDFP PFFTMQKNAD TRFKQTEAWC SLILDYYNHH -KLFRLRVTD
       PLFYNKAIDR RLTKPDVMEI LSILHKKGNI EWEDCKVFWK TPAQWSDIIF
       KWVNQSGLNV CTLHEITNGP HSEFYALDDQ ILMQAIQI-L QASGKAELMG
       --TEGVKF
Mtect  FAWPWQYDFP PFFTMQENAD THEKQLNMWC DLVIAYQKYK -NQATISIQE
       PLFNNKQLNR KLQQRDIEKV FNALCKKGNL EWTDCYILWK TVDQWAELIY
       SWVGRNGMAV CTLYEITKNE NNEFYELEES LLLRAIKV-L ELKNKAELIG
       --NDGVKF
Lerin  FEWPWQYNFP PFYTLQPNVS TQHRQLAAWC SLVLSYLRYH -KLYTIDVLE
       PLFNNKKIQR KFPVEAIQVV LEELRKKGNL EWIDCLIMWR RPEEWGKLVY
       QWVSKNGLTV LTFYELSNGD DTEFHGLEEW LLLRALQT-L QSERKAEIIT
       LDSKGVKF
Dreri  FEWPWQYNFP PFFTLQPNVD TRQKQLAAWC SLVLSYCRHR -KLYTLDVLE
       PVFNNKKIER KLSVEAIQVV FEELRKKGNL EWLDCLIMWR RPEEWGKLIY
       QWVSKNGMVV FTLYELANGD DTEFHGLEDW MLLRSLQA-L QTDGKAEIIT
       MDGKGVKF
Frubr  FEWPWQYNFP PFFTLQPNVD TRQKQLAAWC SLALSYCRHH -KLYTLDVLE
       PVFNNKKIER KLSMEAIQVV FEELRKKGNL EWLDCLVMWR RPEEWGKLMY
       QWVSRNGMVV FTLYELSNGD DTEFHGLEEW MLLRSLQA-L QAEGKAEIIT
       MDGKGVKF
Gacul  FEWPWQYNFP PFFTLQPNVD TRQKQLAAWC SLALSYCRHH -KLYTLDVME
       PVFNNKKMER KLSIEAIQVV FEELRKKGNL EWLDCLVMWR RPEEWGKLIH
       QWVSKNGMVV FTLYELSNGD DTEFHGLEDW MLLRSLQA-L QTDGKAEIII
       MDGKGVKF
Ipunc  FEWPWQYNFP PFFTLQPNAD TRQKQLAAWC SLALSYCRHR -KLYTVDVLE
```

```
      PMFNNKKIER KLSVEAIQVV FEELRKKGNL EWMDCLIMWR RPEEWGKLIY
      QWVSKNGMVV FTLYELSNGD DTEFHGLEEW MLLRALQA-L QADGKAEIIT
      MDGKGVKF
Olati FEWPWQYNFP PFFTLQPNAD TRQKQLEAWC SLALSYCRHH -KLYTLDVME
      PMFNNKKIER KLSMEAIQIV FEELRKKGNL EWLDCLVMWR RPEEWGKLIY
      QWVSKNGMVV FTLYELSNGD DTEFHGLEEW MLLRSLQA-L QTDGRAEIIS
      MDGKGVKF
Omyki FEWPWQYNFP PFFTLQPNVD TRQKQLAAWC SLALSYCRHH -KLYTLDIME
      PVFNHKNIDR KLSMEAILIV FEELRKKGNL EWLDCLVMWR RPEEWGKLIY
      QWVSKNGMVV FTLYELANGD DTEFHGLEDW MLIRSLQA-L QMDGKAEVIS
      MDGKGVKF
Pfles FEWPWQYNFP PFFTLQPNVD TRQKQLAAWC SLALSYCRHH -KLYTLDIME
      PVFNNKKIER KLSMEAIQVV FEELRKKGNL EWLDCLVMWR RPEEWGKLIY
      QWVSRNGMNV FTLYELVNGD DTEFHGLEDW MLQRSLQA-L QTDGKAEIIT
      LDGXGVKF
Pprom FEWPWQYNFP PFFTLQPNVD TRQKQLSAWC SLALSYCRHR -KLYTLDVLE
      PMFNNKKIER KLSVEAIQVV FEELRKKGNL EWLDCLIMWR RPEEWGKLIY
      QWVSKNGMVV FTLYELANGD DTEFHGLEDW MLLRSLQA-L QADGKAEIIS
      MDGKGVKF
Ssala FEWPWQYNFP PFFTLQPNVD TRQKQLAAWC SLALSYCRHH -KLYTLDIME
      PVFNHKNIDR KLSMEAILIV FEELRKKGNL EWLDCLVMWR RPEEWGKLIY
      QWVSKNGMVV FTLYELANGD DTEFHGLEDW MLIRSLQA-L QMDGKAEVIS
      MDGKGVKF
Tnigr FEWPWQYNFP PFFTLQPNVD TRQKQLAAWC SLALSYCRHH -KLYTLDVLE
      PVFNNKKIER KLSMEAIQVV FEELRKKGNL EWLDCLVMWR RPEEWGKLIY
      QWVSRNGMVV FTLYELSNGD ETEFHGLEEW MLLRSLQA-L QAEGRAEIIT
      MDGKGVKF
Ggall FSWPWQYSFP PFFTLQPNGD TRQKQLAAWC ALALAYSRRQ -RLPAMTLRE
      PLFANRRLQR KLPPEAIQVV LEELRKNGNL EWLDFLIMWR RPEEWGKLIY
      QWVSKNGLTV FTLYELISGD DTEFHGLDEA MLLRALQA-L QQEHKAEIIT
      LDGRGVKF
Tgutt FAWPWQYSFP PFFTLQPNGE TRQKQLSAWC ALALAYSQQH -RLPAMTVRE
      PLFANHRLQR KLPLESIQVV LEELRKNGNL EWLDFLIMWK RPEEWGKLIY
      QWVSKNGLTV FTLYELVSGD DTEFHGLDEA TLLRALQA-L QQEHKAEIIT
      LDGRGVKF
Xlaev FEWPWQYNFP PFFTLQPNVD TRQKQLSAWS SLVLSYCRQN -KLYTMNLME
      PLFNNKKIQR KLSLESVQVV LEELRKKGNL EWIDFLIMWR RPDEWGKVIY
      QWVSKNGMTV FTLYELISGD DTEFHGLDEA MLLRSLEA-L QQEHKAEIIT
      LESRGVKF
Xtrop FEWPWQYNFP PFFTLQPNVD TRQKQLSAWS SLVLSYCRHN -KLYTMNLME
      PLFNNKKIQR KLSLESVQVV LEELKKKGNL EWIDFLIMWR RPDEWGKVIY
      QWVSKNGMTV FTLYELISGD DTEFHGLDEA MLLRSLEA-L QQEHKAEIIL
      LDSRGVKF
Btaur FEWPWQYRFP PFFTLQPNVD TRQKQLAAWC SLVLTFCRLH -KQSSMTVME
      PLFNNVKLQR KLPVESIQVV LEELRKKGNL EWLDFLIMWR RPEEWGKLIY
      QWVSKSGQNV FTLYELTNGE DTEFHGLDEA TLLRALQA-L QQEHKAEIIT
      VDGRGVKF
Cfami FEWPWQYRFP PFFTLQPNVD TRQKQLAAWC SLVLSFCRLH -KQSSMTVME
      PLFNNVKLQR KLPVESIQVV LEELRKKGNL EWLDFLIMWR RPEEWGKLIY
      QWVSRSGQNV FTLYELTNGE DTEFHGLDEA TLLRALQA-L QQEHKAEIIT
      VDGRGVKF
Ecaba FEWPWQYRFP PFFTLQPNVD TRQKQLAAWC SLVLSFCRLH -KQSSMTVME
      PLFNNVKLQR KLPVESIQVV LEELRKKGNL EWLDFLIMWR RPEEWGKLIY
      QWVSRSGQNV FTLYELTNGE DTEFHGLDEA TLLRALQA-L QQEHKAEIIT
      VDGRGVKF
Hsapi FEWPWQYRFP PFFTLQPNVD TRQKQLAAWC SLVLSFCRLH -KQSSMTVME
      PLFNNVKLQR KLPVESIQIV LEELRKKGNL EWLDFLIMWR RPEEWGKLIY
```

```
       QWVSRSGQNV FTLYELTNGE DTEFHGLDEA TLLRALQA-L QQEHKAEIIT
       VDGRGVKF
Mmula  FEWPWQYRFP PFFTLQPNVD TRQKQLAAWC SLVLSFCRLH -KQSSMTVME
       PLFNNVKLQR KLPVESIQIV LEELRKKGNL EWLDFLIMWR RPEEWGKLIY
       QWVSRSGQNV FTLYELTNGE DTEFHGLDEA TLLRALQA-L QQEHKAEIIT
       VDGRGVKF
Mdome  FEWPWQYRFP PFFTLQPNVD TRQKQLAAWC SLVLSFCRLH -RQSSMTVME
       PLFNNNKLQR KLPMESIQIV LEELRKKGNL EWLDFLIMWR RPEEWGKLIY
       QWVSKSGQNV FTFYELTNGD DTEFHGLDEA TLLRALQA-L QLEHKAEIIT
       VDGRGVKF
Mmusc  FEWPWQYRFP PFFTLQPNVD TRQKQLAAWC SLVLSFCRLH -KQSSMTVME
       PLFNNVKLQR KLPVESIQIV LEELRKKGNL EWLDFLIMWR RPEEWGKLIY
       QWVSRSGQNV FTLYELTSGE DTEFHGLDEA TLLRALQA-L QQEHKAEIIT
       VDGRGVKF
Ocuni  FEWPWQYRFP PFFTLQPNVD TRQKQLAAWC SLVLSFCRLH -KQSSMTVME
       PLFNNVKLQR KLPMESIQIV LEELRKKGNL EWLDFLIMWR RPEEWGKLIY
       QWVSRSGQNV FTLYELTNGE DTEFHGLDEA TLLRALQA-L QQEHKAELIT
       IDGRGVKF
Rnorv  FEWPWQYRFP PFFTLQPNVD TRQKQLAAWC SLVLSFCRLH -KQSSMTVME
       PLFNNVKLQR KLPVESIQIV LEELRKKGNL EWLDFLIMWR RPEEWGKLIY
       QWVSRSGQNV FTLYELTSGE DTEFHGLDEA TLLRALQA-L QQEHKAEIIT
       VDGRGVKF
Sscro  FEWPWQYRFP PFFTLQPNVD TRQKQLAAWC SLVLSFCRLH -KQSSMTVME
           PLFNNVKLQR KLPVESIQVV LEELRKKGNL EWLDFLIMWR RPEEWGKLIY
           QWVSRSGQNV FTLYELTNGE DTEFHGLDEA TLLRALQA-L QQEHKAEII
           TVDGRGVKF
```
