# Supplementary material for: Genetic structure and evolution of the Vps25 family, a yeast ESCRT-II component
Source: BMC Evol Biol. 2006 Aug 4;6:59. doi: 10.1186/1471-2148-6-59 (PMC1579232; doi:10.1186/1471-2148-6-59)
Supplement: Additional File 6 — Additional Figure 4: Location of introns within aligned Vps25 sequences [file 1471-2148-6-59-S6.pdf]

Additional File 6

Additional Figure 4

Location of introns within aligned Vps25 sequences.

Thirty six full length Vps25 orthologs were found to be encoded by greater than one exon (Table 2). These genes were translated and aligned using ClustalX and shaded using Boxshade, where regions of greater than 50% conservation are shaded (identical amino acids are in black boxes and similar amino acids in gray boxes). A consensus amino acid sequence is presented below the alignment. Totally conserved amino acids are in upper case letters, whilst those highly conserved are in lower case letters. Amino acid numbers are given to the left of each sequence. The asterisks are present where a given intron site is present in the corresponding gene. Where intron splice sites are conserved, the phases of intron insertion were always identical, with the exception of intron Is, which appears to have slipped by 7 nucleotides and changed phase (Table 2). Splice sites are numbered 0-V above the alignment, and correlate with the intron nomenclature from Table 2.

```
                                     0                            I Is

TannuVps25    1 MDSVDF------------- ----------------------NTHVKFKNFPPLYT EQ*INNLTLSKQLEIWHKIINDEVIT
TparvVps25    1 MDSVDF------------- ----------------------NTHEKFKNFPPLYT EQ*INNLTLSKQLEIWHKIINDEVTA
PprimVps25    1 MWHYLNSFEYTYLLIIYIK*IITFKTLEI-------------SSILHVHSLSQVYS LQ DHKETRKKQITQWSEIVHLYFQS
EhistVps25    1 MT----------------- ----------------------FGIPEFAKFPPFYT IQ LVDKTKNQQLQLWSQLILKYCEC
DdiscVps25    1 MSNQV-------------- ----------------------FQFPPYYHKEPFFT*IQ PILNTRKKQFQMWQDLILQYCRY
OsatiVps25    1 MQRLGD------------- ----------------------FRLPPFFNYPPYFT LQ PVRETREKQVQLWKDLILDYCRS
AthalVps25    1 MQKLAD------------- ----------------------FKLPQFFNYPPYFT*LQ PVRDTREKQIQLWKELILDYCKS
SpombVps25    1 M------------------ -----------------------RVPSIYNFPPFFT*RQ LNDNTWHSQKAAWQMWILLWCRE
AfumiVps25    1 MSQSQSQSQSHSTTT---- ----------------------FQFPPTYSFPPFFT RQ PNSTTRLSQLQKWSSLIQSWCRH
AoryzVps25    1 MSNSTP------------- ----------------------FQFPPTYSFPPFFT PQ PNSTTRLSQLQKWSLLIQSWCRH
BfuckVps25    1 MSTSQTQTSVKI------- ----------------------FKFPREHSFPPFFT LQ PTSSTVHAQLRKWSDLILSYFAF
CimmiVps25    1 MPPLTSHPATPTPAEQNT- ----------------------FPFPAPHSFPPFFT LQ PNAQTLLSQLQKWSALIQAYCRH
GzeaeVps25    1 MAATTTTSAPADT------ ----------------------FKFPREYHFPAFFT RQ TNLTTLHAQHNKWADLILAYARH
NfiscVps25    1 MSQTQSHPTTT-------- ----------------------FQFPPTYSFPPFFT PQ PNSTTRLSQLQKWSSLIQSWCRH
SscleVps25    1 MSTPQTITPAKT------- ----------------------FKFPREHSFPPFFT LQ PTSATLHAQLRKWSDLILSYFAF
TreesVps25    1 MATTAATPSPTPPTSTSTS TPTTTTG---------------FAFPREYSFPPFFT RQ PNIAIHHAQLTKWSALVLSYARH
UreesVps25    1 MATTPSLLAAPAPTPQQPP SAADT-----------------FAFPAPHSFPPFFT LQ PNTQTLLSQLQKWSALIQSYCRH
CcineVps25    1 MSLSTHTTPSGSSSVLDFW SSTSDLTQHAHCLG--------YLLPSIHSAPPFFT*QQ PNPSTQGIVVEQWIKLLLSYARY
PchryVps25    1 MAHVPSKDEVAGTEESIG- ----------------------YLLPSIHSAPPFFT*QQ PNPNTQAVVTEHWTKLILSYARH
RoryzVps25    1 MSN---------------- ----------------------FELPSIFDFPPFFT*RQ VTESTWKSQAFEWESIILSYARH
SpurpVps25    1 MGN---------------- ----------------------FEWPWQYEFPPFFS*LQ PNLETRKKQLLAWCDLFLAFHKH
CbrigVps25    1 MATTSA------------- ----------------------FKWPWQYDFPPFFT*IQ KSLNTKDKQLEAWARLVIDYAQH
CelegVps25    1 MAAATTTASA--------- ----------------------FKWPWQYDFPPFFT*IQ KSLNTKDKQLEAWARLVIDYAQH
AgambVps25    1 MGA---------------- ----------------------FQWPWEYSFPPFFT*VQ VHAKTKEQQLATWKELVLNYQKH
AmellVps25    1 MAE---------------- ----------------------IEWPWQYSFPPFFT*LQ PHSDTRAKQLSAWKSLILEYYRI
DmelaVps25    1 MAE---------------- ----------------------FQWPWEYTFPPFFT*LQ PHEETRQQQLKVWTDLFLKYLRH
DpseuVps25    1 MTE---------------- ----------------------FQWPWEYTFPPFFT*LQ PHEETRQQQLKVWTDLFLKYLKH
FrubrVps25    1 MS----------------- ----------------------FEWPWQYNFPPFFT*LQ PNVDTRQKQLAAWCSLALSYCRH
TnigrVps25    1 MS----------------- ----------------------FEWPWQYNFPPFFT*LQ PNVDTRQKQLAAWCSLALSYCRH
BtaurVps25    1 MAMS--------------- ----------------------FEWPWQYRFPPFFT*LQ PNVDTRQKQLAAWCSLVLTFCRL
CfamiVps25    1 MAMS--------------- ----------------------FEWPWQYRFPPFFT*LQ PNVDTRQKQLAAWCSLVLSFCRL
HsapiVps25    1 MAMS--------------- ----------------------FEWPWQYRFPPFFT*LQ PNVDTRQKQLAAWCSLVLSFCRL
MdomeVps25    1 MATS--------------- ----------------------FEWPWQYRFPPFFT*LQ PNVDTRQKQLAAWCSLVLSFCRL
MmuscVps25    1 MAMS--------------- ----------------------FEWPWQYRFPPFFT*LQ PNVDTRQKQLAAWCSLVLSFCRL
RnorvVps25    1 MAMS--------------- ----------------------FEWPWQYRFPPFFT*LQ PNVDTRQKQLAAWCSLVLSFCRL
SscroVps25    1 MAMS--------------- ----------------------FEWPWQYRFPPFFT*LQ PNVDTRQKQLAAWCSLVLSFCRL
consensus     1 M                                         f fp  y fppfft lQ pn  tr kql  W  lilsycr

                                        II         III

TannuVps25   46 NYSLHKIGTETIN------FPPFKNEE--IV RNVNVSFLALILEYLAEK QYAFYLHPIQLFCKKHNVTIWGALFLKKNHK
TparvVps25   46 NYSLHKLGTASVN------FPPFKNEE--IL RNVDVSFLALILGYLVEK QYAFYLHPIQFFCKKNNVSIWGALFLKKSHK
PprimVps25   68 H-KILESSISEILN-----FPIFQDSSSGII KRLDSSEIKEILNQMAQL --------------------GSIEWKNDQ--
EhistVps25   42 -IKKPIMKQSEFNK-----LPIFHNEE--LH*RTLSENGIELVKEFMVNN --------------------NKIIDLNKSS-
DdiscVps25   45 -YKIYELDINESIKSN---SVLFNNEK--IN*RKLSREALKSIIDDIIEN --------------------GFAEWVDKE--
OsatiVps25   46 -QKLYIISLEED-------FPLFSNPK--IE*RSLSHEAKEVFLAALVYE*--------------------GRAEWMDKG--
AthalVps25   46 -QKIFLIGVEED-------FPLFSNSA--ID*RSLSHEARETFLSAIVGE*--------------------GRAEWLDKG--
SpombVps25   40 -NRQTSITINPELLES----SLLHNST--IH RTLPLSVFREIVEDMVKQ --------------------NLAEWTEKR--
AfumiVps25   55 -HRIYRLSLIEAIES-----PLFHNAT--LR KRLSLSEARAVLDWMAKP -------------EEEGGGGRRAEWIDGG--
AoryzVps25   46 -HRTYRLSLIEAIES-----PLFHNST--LR KRIPLSEARNILDWMAES -------------EEKGGGGRRAEWVDGT--
BfuckVps25   52 -HRLFRLTVSTLLSS-----ELFKNER--IN RRLDEEGLREVLEFMRKE --------------------GRVEWIGGG--
CimmiVps25   58 -HRLYRLSLVDALDS-----PLFHNKQ--IR KRLSLVDARRIVDWMCGA -----------------QGGRRAEWVGGE--
GzeaeVps25   53 -NRIFRLSLSEAADS-----DLFVNRK--LD RRLQFDDIRDVVSFMHTD --------------------GRVEYV-----
NfiscVps25   51 -YRIYRLSLIEAIES-----PLFHNAT--LR KRLSLSEARAVLDWMAKP -------------EEEGGGGRRAEWIDGG--
SscleVps25   52 -YRLFRLTISTLLSS-----ELFKNER--IN RRLDEEALREVLEFMRKE --------------------GRVEWIGGS--
TreesVps25   66 -HRLFRLVVSSAAES-----ELFHNRA--IN RRLGPADIREVLDFMRKD --------------------GRAEFIRAS--
UreesVps25   64 -HRLYRLSLVDALDS-----ALFHNRT--IR RRLSLADARKVLDWMCSA -----------------EGGRRAEWVGGE--
CcineVps25   73 -RKLFILRVDDAEEKGSEWDEVLRNER--IN*RRVKPAYLETIISTMVKK --------------------NQAAYEPPK--
PchryVps25   58 -RRLFLLRVEDTEVAGNDWDEIFRNEQ--IR*RRLLPSHLAHIMEDMVTK --------------------NKAVYEPAR--
RoryzVps25   43 -KHLFRLELHNAT-TANSGYDIFENKK--IN RRLSFEALQDIIEEMVKK*----------------------AEWEGGP--
SpurpVps25   43 -HRIYTVDLKEAASS-----ELFNNTK--LN*RKLSGEGILLVLEELRQK*--------------------GNIEWTDKA--
CbrigVps25   46 -NKIYSLDIAEATTS-----ELFNNQK--LN*RRLSTDGVNTVLQYLEQK*--------------------KLIEFTDNG--
CelegVps25   50 -NKIYSLDIAEATTS-----ELFNNQK--LN*RRLSTDGVNTVLQYLEQK*--------------------KLIEFTDNG--
AgambVps25   43 -EGQALLNIAEDA-------PPFVNRE--LA RKLSPEARLWVMEELART --------------------GHAATADKR--
AmellVps25   43 -TKQAIIDVREIHSS-----PLFNNTA--IN*RKLPSEAVLLLLEELAKS --------------------GNASPLDKT--
DmelaVps25   43 -TNRFTLSIGDQNS------PLFHNEA--LK RRLSPELVLAILGELERS --------------------GHANPLDKR--
DpseuVps25   43 -TNKFSLSINEQSL------PLFHNES--IQ RRLSPELILVILEQLQRS --------------------GHATALDKR--
FrubrVps25   42 -HKLYTLDVLEAQES-----PVFNNKK--IE*RKLSMEAIQVVFEELRKK*--------------------GNLEWLDKN--
TnigrVps25   42 -HKLYTLDVLEAQES-----PVFNNKK--IE*RKLSMEAIQVVFEELRKK*--------------------GNLEWLDKN--
BtaurVps25   44 -HKQSSMTVMEAQES-----PLFNNVK--LQ*RKLPVESIQVVLEELRKK*--------------------GNLEWLDKN--
CfamiVps25   44 -HKQSSMTVMEAQES-----PLFNNVK--LQ*RKLPVESIQVVLEELRKK*--------------------GNLEWLDKN--
HsapiVps25   44 -HKQSSMTVMEAQES-----PLFNNVK--LQ*RKLPVESIQIVLEELRKK*--------------------GNLEWLDKS--
MdomeVps25   44 -HRQSSMTVMEAQES-----PLFNNNK--LQ*RKLPMESIQIVLEELRKK*--------------------GNLEWLDKN--
MmuscVps25   44 -HKQSSMTVMEAQES-----PLFNNVK--LQ*RKLPVESIQIVLEELRKK*--------------------GNLEWLDKN--
RnorvVps25   44 -HKQSSMTVMEAQES-----PLFNNVK--LQ*RKLPVESIQIVLEELRKK*--------------------GNLEWLDKN--
SscroVps25   44 -HKQSSMTVMEAQES-----PLFNNVK--LQ*RKLPVESIQVVLEELRKK*--------------------GNLEWLDKN--
consensus    81  hkly lsi ea  s     plf n k  I  rrl  e l  vle l k                     g aewldk
```

```
                                                                                       IV

TannuVps25   118 GTTLFQIHDEYTKSLNPKDNKAETDEIDSLKKKRNLLVKSTFRFGVFPYPLSEMTNSVLEC IKSQCTNRDIETIYHIFYS
TparvVps25   118 GSTLYQIHQDYTKALNSKDNKVEGDEIESLKKKRNLLLKSKFNFGVFPYPLTEMANSVLEC IKSQCTTRDIETVYHIFYS
PprimVps25   120 ------------------------------------------NFSVNLVSPFELADAIYAW AKEKKLIGYTETLRGITEG
EhistVps25    93 ------------------------------------------KLILLYKPLREWGKELYEY GNSKGLIGQSDTFFSIEND
DdiscVps25    97 ---------------------------KEKEKEKDKDNNNNNRVLIMWRKPDEWASLIYKW VADCGLLNTVLTVWEIQNG
OsatiVps25    94 ----------------------------------------HKKCLILWLRIQDWANYILNF*VKDNGLEDSVMTVEEIRSG
AthalVps25    94 ----------------------------------------HRKCLILWHRIQDWADIVLQF*VRDNGLEDSVMTVEEIRSG
SpombVps25    91 --------------------------------------NPKDVFWVYWRSISEWGNMILKW*LSDMGREGSICTFYELQEQ
AfumiVps25   112 ---------------------------------------SKSVAWIWWRRPEEWAGIVADW*VEATGQKNVVLTVYELLEG
AoryzVps25   103 ---------------------------------------NKTIAWVWWRRPEEWAGILADW*VENTGQKNVVLTVYELVEG
BfuckVps25   102 ----------------------------------GNGKVGGDICWVWWRKVDEWARVIEDW VDETGQRGSVLTLYELVEG
CimmiVps25   111 -------------------------------------AGGKSVAWIWWRRPEEWAGVIADW*VEETAQKNTVLTLYELTEG
GzeaeVps25   100 -----------------------------------GGGTSGDVVFLYWRKPEEWAELVENY VEESGQKGSVLTVYELVEG
NfiscVps25   108 ---------------------------------------SKSVAWIWWRRPEEWAGIVADW*VEATGQKNVVLTVYELLDG
SscleVps25   102 --------------------------------GSGSGKVGGDVCWVWWRKVDEWARIIEEW VDETGQRGSVLTLYELVEG
TreesVps25   116 ----------------SAAAAGSDVGGFSSGAGAAGGGGAGDVVLLYWRKPEEWAALVEAY VDETAQKGSVLTVYELTEG
UreesVps25   117 -------------------------------------AGGKSAAWIWWRRPEEWAGVIADW*VEETAQKNTVLTLYELIEG
CcineVps25   128 ---------------------------------------QTKAVLLYWRTPEEWAEVLHEW*ATSTGQLNTIMTFYEITDP
PchryVps25   113 ---------------------------------------QTRSVLLYWRTPEEWAEVLHNW*ADSTGQLNTILTFYEIIEP
RoryzVps25    95 -------------------------------------KGSKTEAYLYWHTPEEWANLIWNW*INETGQNDQIVTYYEIAHG
SpurpVps25    93 ----------------------------------------KTRCLVMWRTPEEWGNLIYKW*AGNSGMTNTVCTLYEIAQG
CbrigVps25    96 ----------------------------------------RTRFHIFWRRPDVWANMIYQW AVENAFLNTPLTLYEITHG
CelegVps25   100 ----------------------------------------RTRFHIFWRRPDVWANMIYQW AVENAFINTPLTLYEITHG
AgambVps25    91 ----------------------------------------KQQWEVYWHTLDEWSNILYDW AVASGTTNTVCTLYELVAG
AmellVps25    93 ----------------------------------------KQRWLIYWHTLEEWGEIIYNW AQENGFCGSVCTLFELTQG
DmelaVps25    92 ----------------------------------------RQEWQVYWFTLEEYGNMVYDW VQETGQTNTICTLYEIASG
DpseuVps25    92 ----------------------------------------RQEWQVYWYTLEAYGNMVYDW IQETGQTNSICTLYEIASG
FrubrVps25    92 ----------------------------------------KSRCLVMWRRPEEWGKLMYQW*VSRNGMVNAVFTLYELSNG
TnigrVps25    92 ----------------------------------------KSRCLVMWRRPEEWGKLIYQW*VSRNGMVNSVFTLYELSNG
BtaurVps25    94 ----------------------------------------KSSFLIMWRRPEEWGKLIYQW*VSKSGQNNSVFTLYELTNG
CfamiVps25    94 ----------------------------------------KSSFLIMWRRPEEWGKLIYQW*VSRSGQNNSVFTLYELTNG
HsapiVps25    94 ----------------------------------------KSSFLIMWRRPEEWGKLIYQW*VSRSGQNNSVFTLYELTNG
MdomeVps25    94 ----------------------------------------KSSFLIMWRRPEEWGKLIYQW*VSKSGQNNSVFTFYELTNG
MmuscVps25    94 ----------------------------------------KSSFLIMWRRPEEWGKLIYQW*VSRSGQNNSVFTLYELTSG
RnorvVps25    94 ----------------------------------------KSSFLIMWRRPEEWGKLIYQW*VSRSGQNNSVFTLYELTSG
SscroVps25    94 ----------------------------------------KSSFLIMWRRPEEWGKLIYQW*VSRSGQNNSVFTLYELTNG
consensus    161                                         ks  ivfwrrpeewa liy w v  tgq ntv Tlyel  g

                     V

TannuVps25   198 KKECNK DFNKFPEENLAFILSKLSVNNQITLSFNDSVPLDSLNNKNVGVQLL------
TparvVps25   198 KRECNK DFNKFPEENLAFILSYLCVNNKLTLSFNDSVPLDSLNNKNVGLQLL------
PprimVps25   158 SQTDQS*KKFYNLPQEQILKACLILEETGRCQVYEFDG--------LYSIKFI------
EhistVps25   131 KE---S VFYQMDDELLIEGLNSIKEQGKMKLVQHE---------GEYGIFWL-----K
DdiscVps25   150 DDSKKQ EFHQLNTTILMKSLKVLEKQSKCQTFSQD---------ENVGVKFFSI----
OsatiVps25   134 IETRGT*ELAGIDRGVLMRALKLLEQKGKAAIFKGTS-------ADDEGVKFSA-----
AthalVps25   134 TESLGT*ELQGIDRTILMRALKLLENKGKLALFKGTS-------ADDEGVKFSV-----
SpombVps25   133 YK---- EVDCLDEVLLHKVLELLMKKGNIELMKGSS-------GKYSGFKVLKA----
AfumiVps25   153 EATMSQ*EWHGMDADVMLKSLNILVKRGKAQVFGSE---------GQEGVKFF------
AoryzVps25   144 EATMSQ*EWHGMDVDVMMKSLNVLVKRGKAQVFGSE---------GQEGVKFF------
BfuckVps25   148 EGGRGA*EFHGLDTEILQKALAILVKRGKAQVFGQE---------DQQGVKFF------
CimmiVps25   154 EATMSQ EFHGMDPDVLQKSLHVLVKRGKAQVFGNE---------DQQGVKFF------
GzeaeVps25   145 DGTKGT*DIHGMDTDVLLKALNVLVKRNKAQIFGQD---------DSLGVKFF------
NfiscVps25   149 EATMSQ*EWHGMDADVMLKSLNVLVKRGKAQVFGSE---------GQEGVKFF------
SscleVps25   150 EGGRGA*EFHGLDAEILQKALAILVKKGKAQVFGQE---------DQQGVKFF------
TreesVps25   180 ENTRGT*EFHGMDNLVLMKALNILVKQGKAQIFGSE---------DSLGVKFF------
UreesVps25   160 EATISQ EFHGMDPDVLQKSLHTLVKRGKAQVFGSE---------DQQGVKFF------
CcineVps25   169 PVE--S PLTGIPVQLLRKAIGILGKTGRAQTISIS---------DGEGVRFFAA---K
PchryVps25   154 PVP--S QLSGIPMTLLRKAIAVLTKTSRAQIISVA---------DGEGVRFLAGNTSK
RoryzVps25   138 ELAEGQ*EFYDIDHNVLDKALNVLVKRGNAQIFKGTD-------EDSMGVKFFQ-----
SpurpVps25   133 EDTTNE*EFHGLEDWLLKRSLKCLERGRKAELMAF---------DGNEGVKFF------
CbrigVps25   136 DDTTNE*SFHNLEREILMKALTCLEDQRRAQLMNI--------GGDNEGVKFV------
CelegVps25   140 DDTTNE*SFHNLEREILMKALTCLEEQRRAQLMNI--------GGDNEGVKFI------
AgambVps25   131 DNTVGE EFHGLDEGVLKKALKLLEGRGKCELIAFD---------DNEGVKFF------
AmellVps25   133 EDTIDQ*EFYGLDTEILIRALKTLETNKKAELILFD---------DNQGVKFF------
DmelaVps25   132 ENTSHL DFYGVDEAVLLSALRLLEEKGRCELIEMD---------GSHGVKFF------
DpseuVps25   132 ESTTQM DFHGVDESVLLNALRLLEEKGRCELIEID---------GSHGVKFF------
FrubrVps25   132 DDTEGE*EFHGLEEWMLLRSLQALQAEGKAEIITMD---------DGKGVKFF------
TnigrVps25   132 DETEGE*EFHGLEEWMLLRSLQALQAEGRAEIITMD---------DGKGVKFF------
BtaurVps25   134 EDTEDE*EFHGLDEATLLRALQALQQEHKAEIITVS---------DGRGVKFF------
CfamiVps25   134 EDTEDE*EFHGLDEATLLRALQALQQEHKAEIITVS---------DGRGVKFF------
HsapiVps25   134 EDTEDE*EFHGLDEATLLRALQALQQEHKAEIITVS---------DGRGVKFF------
MdomeVps25   134 DDTEDE*EFHGLDEATLLRALQALQLEHKAEIITVS---------DGRGVKFF------
MmuscVps25   134 EDTEDE*EFHGLDEATLLRALQALQQEHKAEIITVS---------DGRGVKFF------
RnorvVps25   134 EDTEEE*EFHGLDEATLLRALQALQQEHKAEIITVS---------DGRGVKFF------
SscroVps25   134 EDTEDE*EFHGLDEATLLRALQALQQEHKAEIITVS---------DGRGVKFF------
consensus    241 e t    efhgld  llikal ll   gka lv  d         d  gvkff
```
