# Supplementary material for: Genetic structure and evolution of the Vps25 family, a yeast ESCRT-II component
Source: BMC Evol Biol. 2006 Aug 4;6:59. doi: 10.1186/1471-2148-6-59 (PMC1579232; doi:10.1186/1471-2148-6-59)
Supplement: Additional File 7 — Additional Figure 5: Genomic context and organization of human and chimpanzee VPS25 [file 1471-2148-6-59-S7.pdf]

Additional File 7

Additional Figure 5

Genomic context and organization of human and chimpanzee *VPS25* (*HsVPS25* and *PtVPS25*).

Gene names are above the corresponding arrows and arrow point to orientation on the genome. Drawings are approximately to scale (see scale bars) and exon diagrams are in the 5’ to 3’ direction.

(A) *HsVPS25* location on chromosome 17 map position 17q21.31.

LEFT- Ideogram of human chromosome with the area amplified marked.

BOTTOM- Genes localising to region 41225458-41349446 bp (arrowheads) on Build 34.3: *HsVPS25* (black arrow); surrounding genes (gray arrows).

Surrounding genes include *EZH1*, *BECN1*, *RAMP2* (receptor [calcitonin] activity modifying protein 2), *WNK4* (protein kinase Wnk4), *FLJ40137* (encodes a partial cyclin N-terminal domain), and *HSPC009* (encodes putative transmembrane protein*)*. TOP- The coding sequence has 6 exons (vertical lines) and covers 5.59 kb (see Table 2).

(**B**) *PtVPS25* location on chromosome 17. Currently, the map position and ideograms of chimpanzee chromosome 17 are unavailable. BOTTOM- Genes localising to region 41225458-41349446 bp (arrowheads) on Build 1.1. The gene layout available from the NCBI has been modified in light of our analyses [see Additional Files 1 and 12] and NCBI annotated gene LOC454699 has been resolved into two genes: *VPS25* and *WNK4*. *PtVPS25* (black arrow); surrounding genes (gray arrows). Surrounding genes include *EZH1*, *BECN1*, *RAMP2* (receptor [calcitonin] activity modifying protein 2), and *WNK4* (protein kinase Wnk4). TOP- The coding sequence is proposed to have 6 exons, by similarity with human *VPS25*. However, gaps between contig sequences (indicated by slashes) mean the exact sequence of a couple of introns and exons (gray) is unclear. Numbering provided for exon 6 is from an 'orphan' contig, unmapped relative to other genes on chromosome 17 [see Additional File 1].

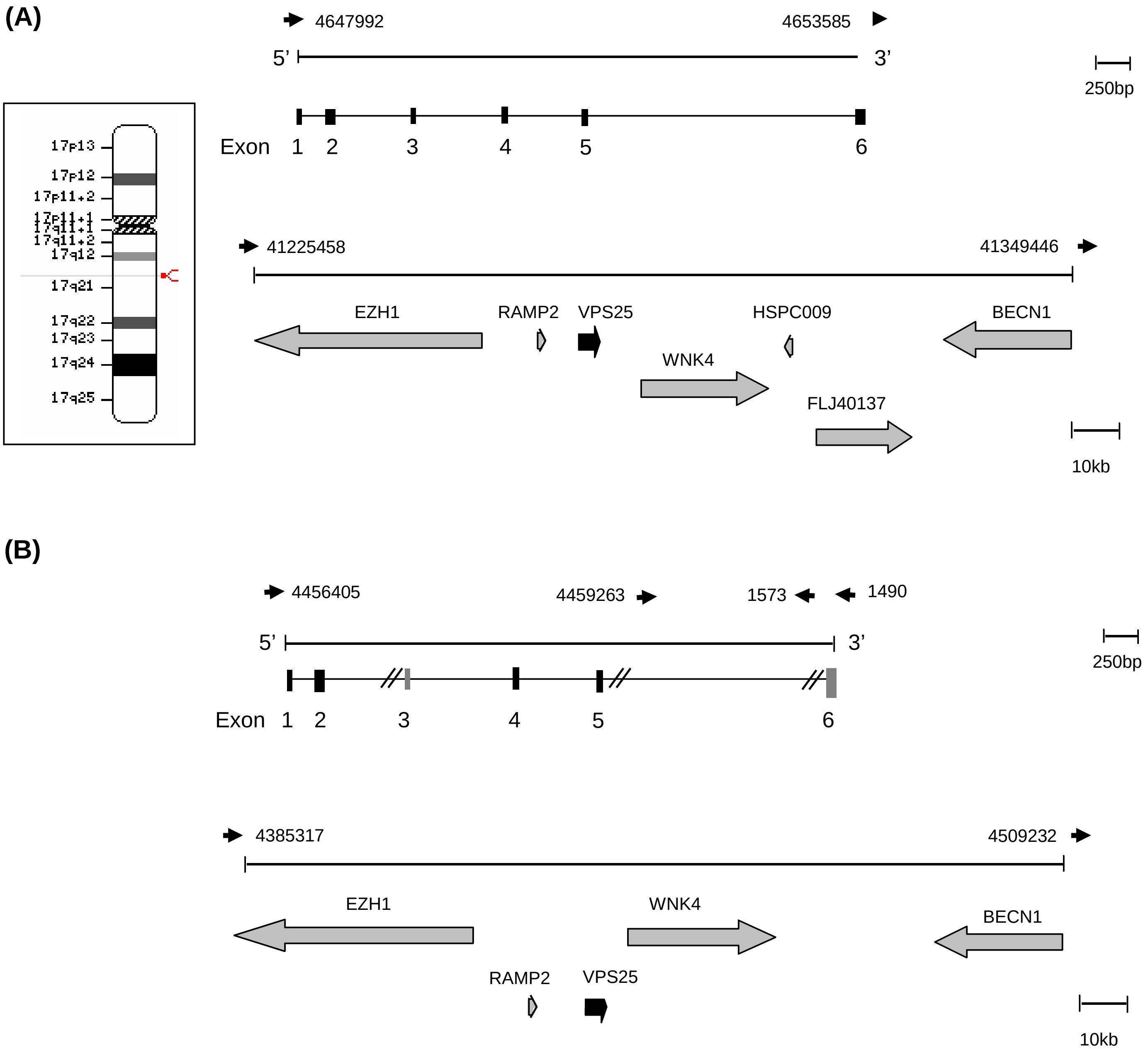

(A)
4647992
4653585
5'
3'
250bp
Exon 1 2 3 4 5 6
17p13
17p12
17p11.2
17p11.1
17q11.1
17q11.2
17q12
17q21
17q22
17q23
17q24
17q25
41225458
41349446
EZH1
RAMP2
VPS25
HSPC009
BECN1
WNK4
FLJ40137
10kb
(B)
4456405
4459263
1573
1490
5'
3'
250bp
Exon 1 2 3 4 5 6
4385317
4509232
EZH1
WNK4
BECN1
RAMP2
VPS25
10kb
