# Supplementary material for: Genetic structure and evolution of the Vps25 family, a yeast ESCRT-II component
Source: BMC Evol Biol. 2006 Aug 4;6:59. doi: 10.1186/1471-2148-6-59 (PMC1579232; doi:10.1186/1471-2148-6-59)
Supplement: Additional File 8 — Additional Figure 6: Genomic context and organization of mouse and rat Vps25 [file 1471-2148-6-59-S8.pdf]

Additional File 8

Additional Figure 6

Genomic context and organization of mouse and rat *Vps25* (*MmVps25* and *RnVps25)*.

(A) *MmVps25* location on chromosome 11, map position 11D (60.0 cM). LEFT-Ideogram of the mouse chromosome with the area amplified marked. BOTTOM- Genes localising to region of 12672776-12783927 bp (arrowheads) on Build 32.1: *MmVps25* (black arrow); surrounding genes (gray arrows). Surrounding genes include *Ezh1*, *Becn1*, *Ramp2* and *Wnk4* (orthologous to the *H. sapiens* genes), *D830013H23RIK*, *D11Ertd99e* (encodes putative transmembrane protein), and *1700051C09RIK* (encodes a partial cyclin N-terminal domain). TOP- The coding sequence has 6 exons (vertical lines) and covers 5.27 kb (see Table 2).

(**B**) *RnVps25* location on chromosome 10, map position 10q32.1. LEFT-Ideogram of the rat chromosome with the area amplified marked. BOTTOM-Genes localising to region 90213177-90332861 bp (arrowheads) on Build 3.4: *RnVps25* (black arrow); surrounding genes (gray arrows). Note: NCBI database currently does not annotate *Vps25* to this area, despite its presence there, due to sequence annotation errors which remain to be corrected [see Additional Files 1 and 12]. Surrounding genes include *Ezh1*, *Becn1*, *Ramp2*, *Wnk4, Ezh1* and *RGD1564337*, the latter being similar to *D11Ertd99e* of mouse. TOP-The coding sequence has 6 exons (see Table 2) and covers 4.93 kb. TOP- We predict that the coding sequence has 6 exons (vertical lines) and covers 4.93 kb, differing from the NCBI entry. Numbering from chromosome 10 genomic contig NW_047339 is presented.

(A)

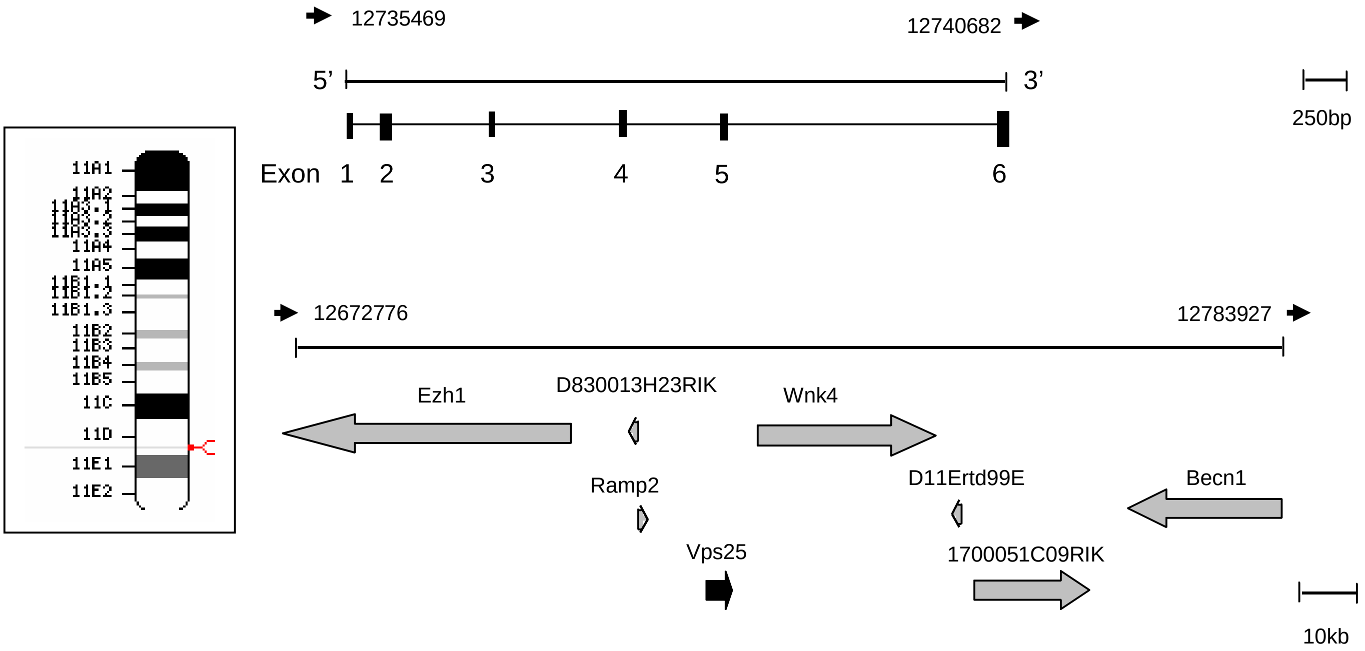

12735469
12740682
5'
3'
250bp
Exon 1 2 3 4 5 6
11A1
11A2
11A4
11A5
11B1.3
11B2
11B3
11B4
11B5
11C
11D
11E1
11E2
12672776
12783927
Ezh1
D830013H23RIK
Wnk4
Ramp2
D11Ertd99E
Becn1
Vps25
1700051C09RIK
10kb

(B)

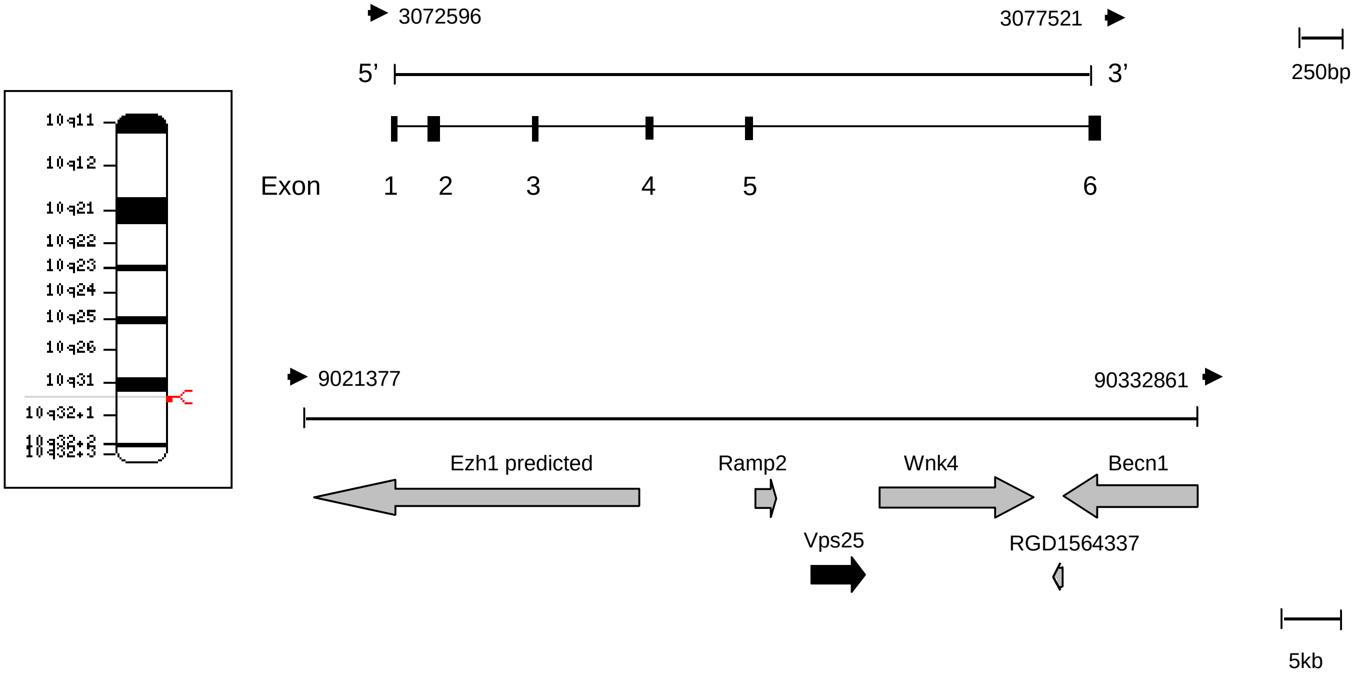

3072596
3077521
5'
3'
250bp
Exon 1 2 3 4 5 6
10q11
10q12
10q21
10q22
10q23
10q24
10q25
10q26
10q31
10q32.1
9021377
90332861
Ezh1 predicted
Ramp2
Wnk4
Becn1
Vps25
RGD1564337
5kb
