# Supplementary material for: Genetic structure and evolution of the Vps25 family, a yeast ESCRT-II component
Source: BMC Evol Biol. 2006 Aug 4;6:59. doi: 10.1186/1471-2148-6-59 (PMC1579232; doi:10.1186/1471-2148-6-59)
Supplement: Additional File 9 — Additional Figure 7: Genomic context and organization of dog and cow Vps25 [file 1471-2148-6-59-S9.pdf]

Additional File 9

Additional Figure 7

Genomic context and organization of dog and cow Vps25 (*CfVps25* and *BtVps25*).

(A) *CfVps25* location on chromosome 9. BOTTOM- Genes localising to region of 23540951-23608432 bp (arrowheads) on Build 2.1: *CfVps25* is indicated by a black arrow and surrounding genes as gray arrows. Note: NCBI database currently does not annotate a Vps25 equivalog to this area, but annotates a homolog with a long amino-terminal extension [see Additional Files 1 and 12]. Here we present a corrected diagram. Surrounding genes on the same strand as *CfVps25* include *LOC490959* (similar to *Wnk4),* and *LOC480515* (similar to *Ramp2*). On the opposite strand are *LOC480514* (similar to *CG7319-PC* isoform C) and *LOC480516* (similar to *Ezh1*). TOP- We predict, by similarity, that the coding sequence has 6 exons (vertical lines) and covers 4.6 kb, which differs from the NCBI entry.

(B) *BtVps25* location on chromosome 19. BOTTOM- Genes localising to region of 23540951-23608432 bp (arrowheads) on Build 2.1: *BtVps25* is indicated by a black arrow and surrounding genes as gray arrows. Surrounding genes on the same strand as *BtVps25* include *LOC615567* (similar to *Wnk4),* and *LOC504230* (similar to *Ramp2*). On the opposite strand are *MGC127306* (similar to *CG7319-PC* isoform C) and *LOC533087* (similar to *Ezh1*). TOP- The coding sequence has 6 exons (vertical lines) and covers 4.2 kb. Numbering is from genomic sequence NW_929520.

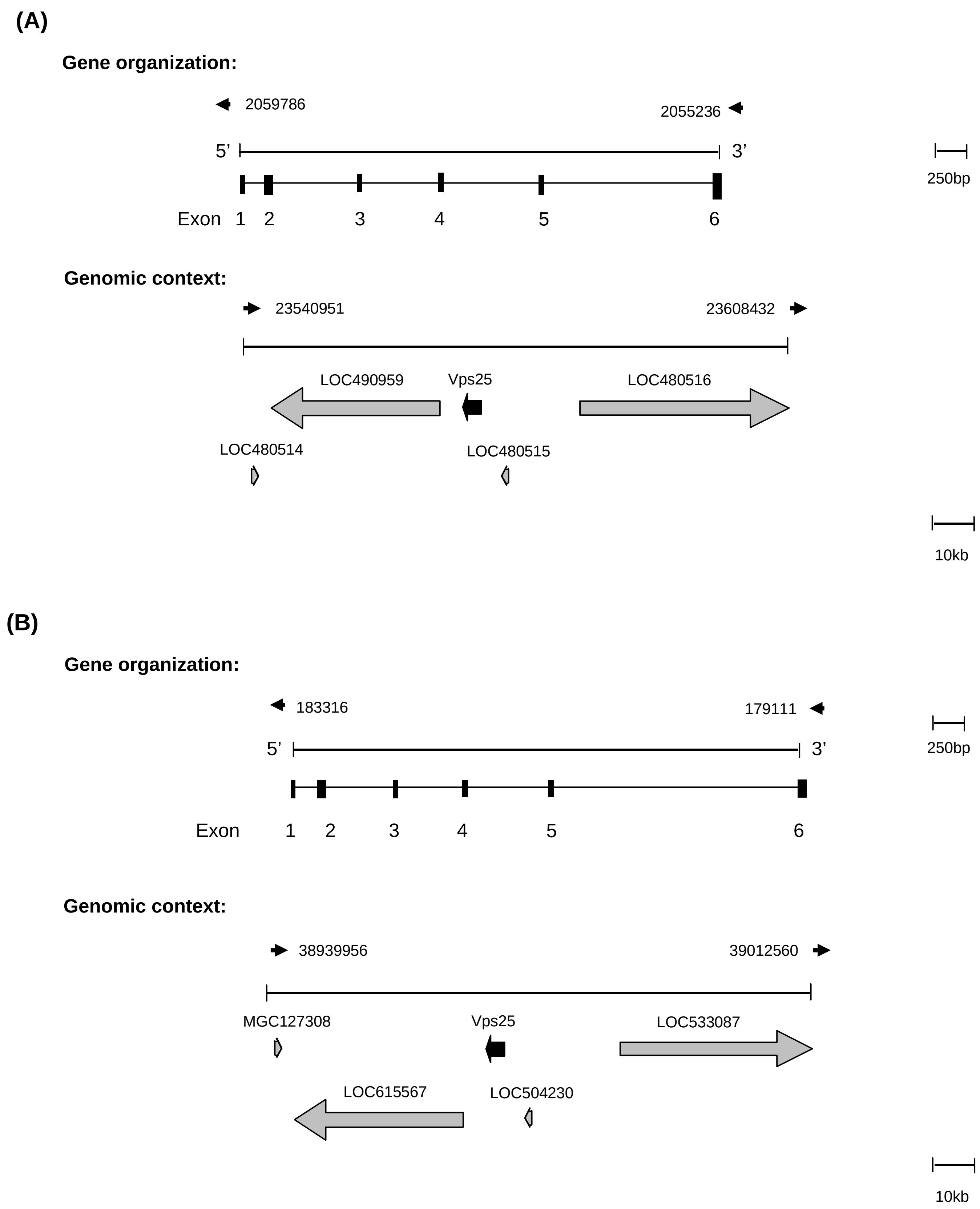
(A)
Gene organization:
2059786
2055236
5'
3'
250bp
Exon 1 2 3 4 5 6
Genomic context:
23540951
23608432
LOC490959
Vps25
LOC480516
LOC480514
LOC480515
10kb
(B)
Gene organization:
183316
179111
5'
3'
250bp
Exon 1 2 3 4 5 6
Genomic context:
38939956
39012560
MGC127308
Vps25
LOC533087
LOC615567
LOC504230
10kb
