# Supplementary material for: Genetic structure and evolution of the Vps25 family, a yeast ESCRT-II component
Source: BMC Evol Biol. 2006 Aug 4;6:59. doi: 10.1186/1471-2148-6-59 (PMC1579232; doi:10.1186/1471-2148-6-59)
Supplement: Additional File 10 — Additional Figure 8: Genomic context and organization of A. gambiae, D. melanogaster, and C. elegans Vps25 [file 1471-2148-6-59-S10.pdf]

Additional File 10

Additional Figure 8

Genomic context and organization of *Anopheles gambiae*, *D. melanogaster*, and *C. elegans Vps25* (*AgVps25*, *DmVps25*, and *CeVps25*).

The sequences are shown in the 5’ to 3’ direction. Drawings are to scale (see scale bars). (**A)** *CeVps25* location on chromosome I, map position position +14.60 cM (interpolated genetic position). TOP- The genomic context surrounding chromosome *CeVps25* localising to region of bp 12746151-12753349 (arrowheads) on genome release WS97. *Vps25* is shown as a black arrow and the surrounding genes as gray arrows, with the arrow pointing to the orientation on the genome. Gene names are above the corresponding arrow. Other genes in this location include *UBA-2* (ubiquitin-activating enzyme-related protein 2) and *IN331* (a RING finger protein) on the plus strand. *IN322* (a translational suppressor-like protein) and *IN324* (an unknown protein) are coded on the minus DNA strand, along with *Vps25*. BOTTOM- The coding sequence has 5 exons (filled blocks) and covers 3.17 kb.

(B) *AgVps25* location on chromosome 3. TOP- The genomic context surrounding *AgVps25* (ESANGGP00000004798) on chromosome 3. Genes localising to region of bp 24571066-24656916 (arrowheads) are shown. *Vps25* is highlighted as a black arrow with the arrow pointing to the orientation on the genome. The surrounding genes are gray arrows, and the names indicated above arrow. Genes on the minus strand with *Vps25* include *ESANGG00000014912* (solute carrier family) and *ESANGG00000004803*

(similar to CD2 antigen binding protein). On the plus strand are *ESANGG0000001190* (gene product has an amino acid transporter/permease domain) and *ESANGG00000015001* (a putative potassium-chloride cotransporter). BOTTOM-The coding sequence has 2 exons (filled blocks) and covers 0.6 kb.

(C) *DmVps25* location on chromosome 2R. TOP- The genomic context surrounding *DmVps25* (CG14750) on chromosome 2R. Genes localising to region of bp 3676855-3690933 (arrowheads) on Build 3.2 are shown. *Vps25* is highlighted as a black arrow with the arrow pointing to the orientation on the genome. The surrounding genes are gray arrows, and their names indicated above arrow. Genes on the plus strand with *Vps25* include *Cirl* (G-protein coupled receptor family) and *CG1474*9 (C-terminus similar to RNA export mediator proteins). On the minus strand is *CG8734* a putative galactosyltransferase. BOTTOM- The coding sequence has 2 exons (filled blocks) and covers 0.6 kb.

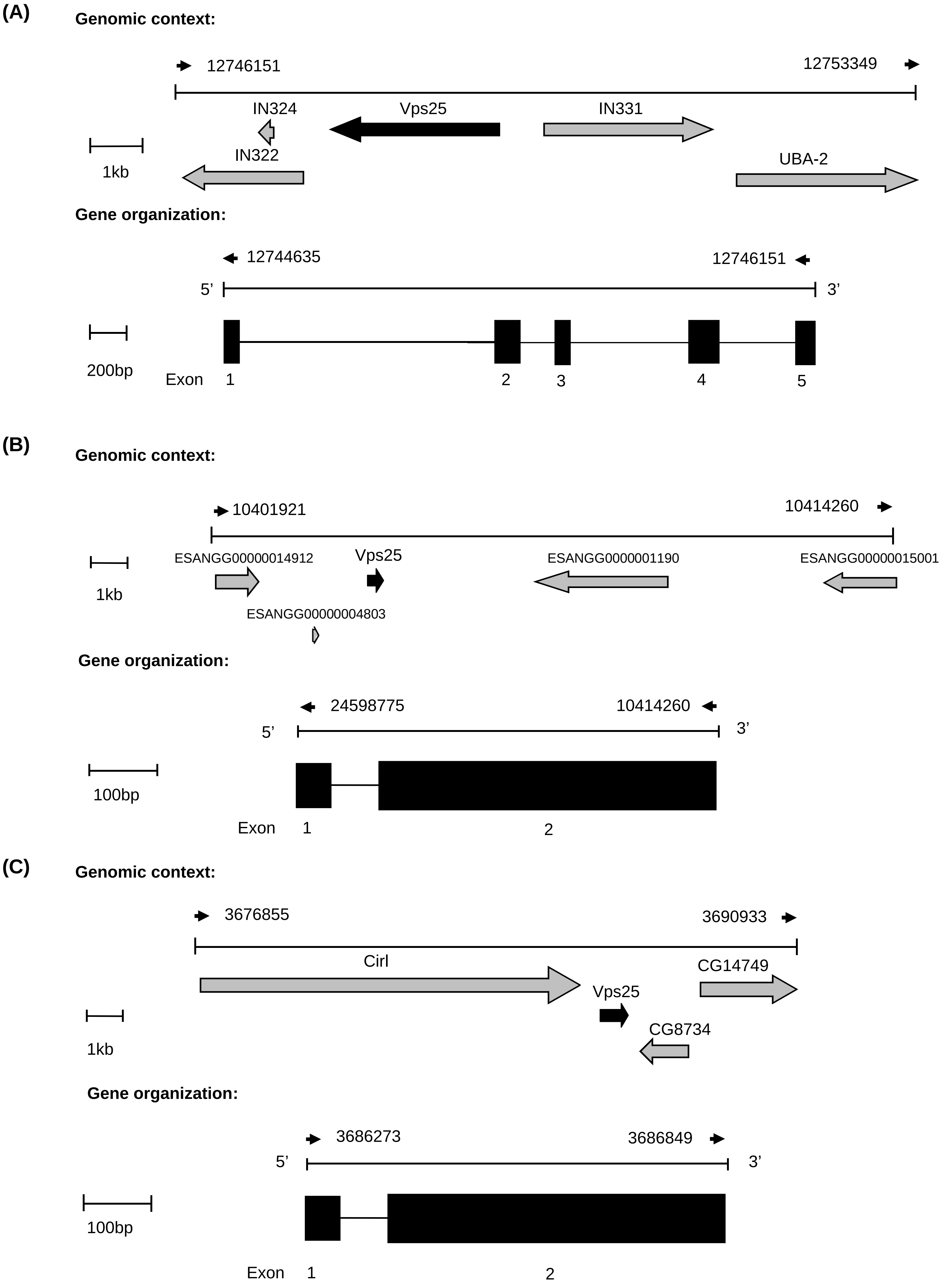
(A)
Genomic context:
12746151
12753349
IN324
Vps25
IN331
1kb
IN322
UBA-2
Gene organization:
12744635
12746151
5’
3’
200bp
Exon 1
2
3
4
5
(B)
Genomic context:
10401921
10414260
ESANGG00000014912
Vps25
ESANGG0000001190
ESANGG00000015001
1kb
ESANGG00000004803
Gene organization:
24598775
10414260
5’
3’
100bp
Exon 1
2
(C)
Genomic context:
3676855
3690933
Cirl
CG14749
Vps25
1kb
CG8734
Gene organization:
3686273
3686849
5’
3’
100bp
Exon 1
2
