# Supplementary material for: Genetic structure and evolution of the Vps25 family, a yeast ESCRT-II component
Source: BMC Evol Biol. 2006 Aug 4;6:59. doi: 10.1186/1471-2148-6-59 (PMC1579232; doi:10.1186/1471-2148-6-59)
Supplement: Additional File 11 — Additional Figure 9: Genomic context and organization of plant Vps25 genes from O. sativa and A. thaliana [file 1471-2148-6-59-S11.pdf]

Additional File 11

Additional Figure 9

Genomic context and organization of plant *Vps25* genes from *Oryza sativa* and *Arabidopsis thaliana* (*OsVps25* and *AtVps25)*.

(**A)** Location of *OsVps25* on chromosome 1. TOP- The genomic context of *OsVps25*. Genes localising to region of bp 108295-132019 (arrowheads) are shown. *Vps25* is highlighted as a black arrow. The surrounding genes are indicated by gray arrows, and arrows point to the orientation on the genome. Gene names are above the corresponding arrow. Note: NCBI database currently does not annotate *Vps25* or *UbC5a* as independent genes in this location [see comments in Additional Files 1 and 4]. The other genes we depict on the minus strand with *Vps25* are *UbC5a*, encoding a ubiquitin-conjugating enzyme, and a gene encoding hypothetical protein P0694A04.24. On the plus strand are genes encoding hypothetical proteins P0694A04.25, P0694A04.27, and P0694A04.28 (the latter being a putative acid phosphatase). BOTTOM- Gene organization of *Vps25*. Exons are represented by filled blocks and introns by horizontal lines. Numbering is from PAC clone P0694A04. The sequence is shown in the 5' to 3' direction. The coding sequence of *OsVps25* has 6 exons and covers 2.9 kb.

(B) *AtVps25* location on chromosome 4. TOP- The genomic context surrounding *Vps25* (also known as At4g19003) on chromosome 4. Genes localising to region of bp 10401921-10414260 (arrowheads), using numbering from clone CHR4v01212004, are shown. *Vps25* is highlighted as a black arrow with the surrounding genes as gray arrows, and the arrow pointing to

the orientation on the genome. Other genes on the minus strand with *Vps25* include *Rpn9* (26*S* proteasome regulatory subunit), *At4g18990* (a putative endo-xyloglucan transferase), and *At4g19010* (a 4-coumaroyl-CoA synthase). On the plus strand is *At4g19000* (encoding an IWS1 C-terminal domain-containing protein).

BOTTOM- The coding sequence has 6 exons (filled blocks) and covers 1.01 kb. The sequence is shown in the 5' to 3' direction. Drawings are approximately to scale (see scale bars).

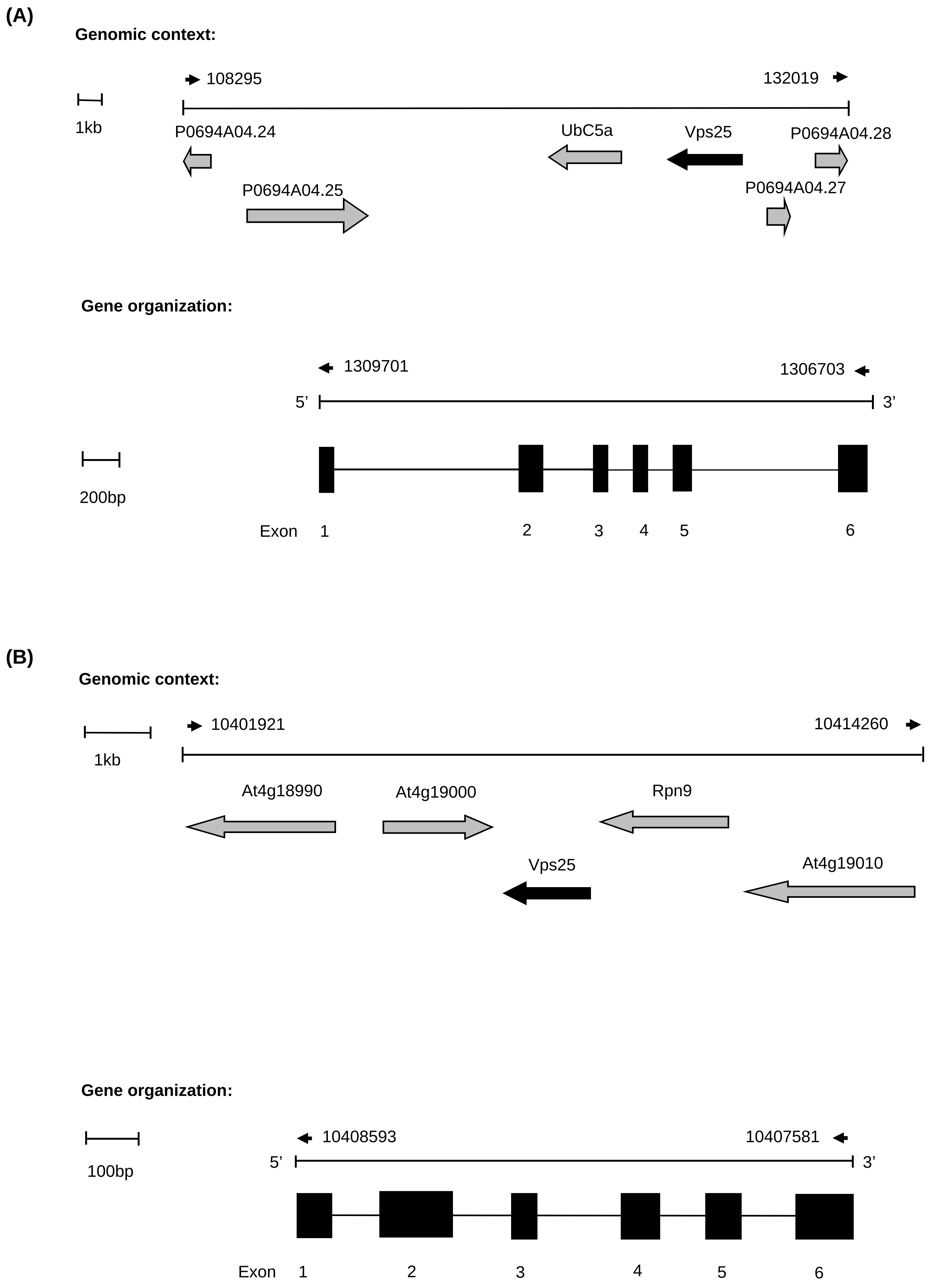
(A)
Genomic context:
108295
132019
1kb
P0694A04.24
UbC5a
Vps25
P0694A04.28
P0694A04.25
P0694A04.27
Gene organization:
1309701
1306703
5’
3’
200bp
Exon 1 2 3 4 5 6
(B)
Genomic context:
10401921
10414260
1kb
At4g18990
At4g19000
Rpn9
Vps25
At4g19010
Gene organization:
10408593
10407581
100bp
5’
3’
Exon 1 2 3 4 5 6
