# Supplementary material for: Genetic structure and evolution of the Vps25 family, a yeast ESCRT-II component
Source: BMC Evol Biol. 2006 Aug 4;6:59. doi: 10.1186/1471-2148-6-59 (PMC1579232; doi:10.1186/1471-2148-6-59)
Supplement: Additional File 12 — Additional Table 3: Taxa, accession numbers and chromosome location of 'hybrid' splice variants of Vps25 [file 1471-2148-6-59-S12.pdf]

Additional File 12

Additional Table 3: Taxa, accession numbers and chromosome location of 'hybrid' splice variants of Vps25.

Species	Protein accession number	mRNA or EST accession number or identifier	Genomic DNA accession number or identifier	Chr	Spliced	Comments
**EXCAVATES**						
**Euglenozoa**						
Kinetoplasts						
Trypanosoma cruzi	XP_805678	XM_800585	AAHK01001895	-	No	Tc00.1047053510287.20 has an extra 53 amino acids at the amino-terminus compared to the Vps25 equivalog, due to the use of an upstream in-frame start codon. The upstream methionine is not conserved in the closely related genomic sequence of *T. brucei*, *T .b. gambiense*, or *T. vivax*.
Trypanosoma congolese	-	congo361b06.p1k_8	-	10	No	There are two methionines upstream and in-frame from that we havepredicted to be used as the start codon for the Vps25 equivalog. Use of these alternative methionines would result in an extra 55 or 66 amino acids being added to the amino-terminus. The upstream methionines are not conserved in the closely related genomic sequence of *T. brucei*, *T .b. gambiense*, or *T. vivax*.
**PLANTAE**						
**Land plants**						
**Magnoliophyta**						
Liliopsida						
Cyperales						
Oryza sativa	NP_917340	NM_192451	NT_079967	1	Yes	Hypothetical protein P0694A04.26. The prototype for the Pfam PF05871 (DUF852) domain. The translation product has a portion of Vps25 fused to a portion of the ubiquitin-conjugating enzyme, UbC5a. This is not conserved in other plant species, and may be due to an error in gene annotation.

<u>**OPISTHOKONTS**</u>						
<u>**Fungi**</u>						
**Ascomycetes**						
Saccharomycotina						
Kluyveromyces lactis	XP_454194	XM_454194	CR382125	E	No	An upstream, in-frame, methionine could add an extra 28 amino acids, to that predicted to be used to translate the Vps25 equivalog. This methionine is not conserved in the genome of closely related *K. waltii*.
**Basidiomycetes**						
Ustilago maydis	XP_760019	XM_754926	AACP01000132	11	No	Hypothetical protein UM03872.1 is predicted to use an in-frame, upstream, methionine which predicts 27 amino-terminal amino acids compared to the Vps25 equivalog.
<u>**Metazoa**</u>						
**Chordata**						
Vertebrata						
Neopterygii						
Danio rerio	XP_688456	XM_683364	NC_007112	1	Yes	Predicted to use an upstream start codon, which adds 8 amino acids to that we predict is used to translate the Vps25 equivalog. This sequence is also truncated at the 3' end compared to the Vps25 equivalog.
Tetrapoda						
Mammalia						
Canis familiaris	XP_849270	XM_844177	NW_876332	9	Yes (on NCBI link)	This is the only canine Vps25 homolog on the protein database at present, but it is not a Vps25 equivalog, as it has a long amino-terminal extension, due to a predicted upstream exon. We suggest this is an annotation error due to the presence of erroneous rat and chimpanzee sequences on the database (see comments below).
Mus musculus	-	BF577527	AL590969	-	Yes	EST begins with Ramp2 coding sequence followed by Vps25 coding sequence, enters the last exon, but the EST ends before the Vps25 stop codon. In order to translate a Ramp2-Vps25 chimera, a -1 frameshift event would need to take place. It has been documented (e.g. on the Protist EST website) that small numbers of chimeric sequences can be generated due to artifactual chimeric clones generated during cDNA library construction. We suggest this is the derivation of this EST

						sequence, as it is not conserved in other species, nor is the sequence in the region where the frameshift event would need to occur.
Pan troglodytes	XP_511521	XM_511521	NW_118498	17	Yes (see NCBI link)	This protein is annotated to the region on the chromosome 17 syntenic to that containing human *VPS25*. A *VPS25* ortholog, has not been annotated to this region, but rather this hypothetical protein, which has part of Vps25 fused to a Wnk4-like kinase domain. This notation is probably due to gaps between the contig sequences in this region of the chromosome and is unlikely to be correct (see below).
Rattus norvegicus	AAP86260	AY321328	NW_047339	10	Yes	High throughput cDNA. This is a longer version of Vps25 with portions of Wnk4 kinase sequence spliced to the amino-terminus of Vps25. The RefSeq database, however, has corrected a similar entry (corrected on January 2006; originally deposited on May 2003) to annotate a true Wnk4 kinase (see NM_175579), with no similarity to Vps25. Nonetheless, the genomic database has yet to be updated.
