# Supplementary material for: Genetic structure and evolution of the Vps25 family, a yeast ESCRT-II component
Source: BMC Evol Biol. 2006 Aug 4;6:59. doi: 10.1186/1471-2148-6-59 (PMC1579232; doi:10.1186/1471-2148-6-59)
Supplement: Additional File 13 — Additional Figure 10: Multiple tissue PCR analysis of the expression of 'hybrid' Vps25 genes [file 1471-2148-6-59-S13.pdf]

Additional File 13

Additional Figure 10

Multiple tissue PCR analysis of the expression of 'hybrid' *Vps25* genes.

A panel of normalised, first strand mouse cDNAs, ready for quantitative PCR, was screened using primers designed to amplify potential mRNAs encoding longer splice variants which may have a Vps25 domain (VTF) [see Additional Files 1 and 12]. These included the Ramp2 sequence with Vps25 sequence spliced to the 3' end (MmRamp2-VTF), and the Wnk4 gene with Vps25 sequence spliced to the 5' end (MmWnk4Long).

(A) Expression of *MmRamp2* (left-hand side) and *MmRamp2-VTF* (right-hand side). Primers were designed to yield products of 511 bp (*MmRamp2*) or 436 bp (*MmRamp2-VTF*). *MmRamp2* PCR products are shown after 34 cycles, as at 38 cycles saturation was observed (data not shown). Expression of *MmRamp2* was detected in all tissues examined, but was highest in lung. No *MmRamp2-VTF* PCR products could be detected after 38 cycles, nor could product be detected after a further 38 cycles using fresh PCR reagents (data not shown). **(B)** Expression of *MmWnk4* (left-hand side) and *MmWnk4L* (right-hand side). Expression was highest in kidney, as expected [98]. No *MmWNK4L* product was seen after 38 cycles, or a further 38 cycles (data not shown). Primers were designed to yield 1470 bp (*MmWnk4*) or 1293 bp (*MmWnk4L*) products. The arrow indicates 500 bp marker and arrowhead the 1000 bp marker. N=negative control (no DNA). P=positive control (*HsVPS25* cDNA). H=housekeeping gene positive control glyceraldehyde-3-phosphate dehydrogenase (*G3PDH*) with an expected product size of 983bp was detected using the following primer pair: 5’-tgaaggtcggagtcaacggatttggt-3’ and 5’-catgtgggccatgaggtccaccac-3’, yielding a product of 983 bp. Primers designed to detect *MmWnk4* were: 5’-ctagcacctcgaaatacggagactgg-3’ and 5’-ggctaccatctcctgagccacctc-3’. The PCR product size expected was 1470 bp. These mouse *Wnk4* primers would also detect *Wnk4L*, if it exists in the same tissues (although our data shows it does not), with a product of the same size. For mouse *Wnk4L*, the primer pairs were

5’-ccttgtcggttccgggttttctgg-3’ and 5’-ccgtagaatcgtgcgactccagcgc-3’ to amplify a 1293 bp mRNA fragment. These primers would not detect *MmWnk4* or *MmVps25*. Primers designed to detect *MmRAMP2* were: 5’-cgcccagcggccgacagcgttgtgcctcc-3’ and 5’-ggcctgggcatcgctgtctttactcc-3’ and should produce a 511 bp product. These mouse and primers would also detect *Ramp2-VTF*, if it exists. Primers specific for *MmRamp2-VTF* were: 5’-cgggtggatctcggctgggtgtgacc-3’ and 5’-gcaccaggcggccagctgcttctgc-3’. The PCR product size expected is 436 bp and these primers would not detect either *MmRamp2* or *MmVps25*. We did not detect the expression of mouse or human VTF domain proteins Ramp2-VTF or Wnk4L experimentally in a wide range of normal tissues. The MmRAMP-VTF sequence (on the database) was obtained from a colon cDNA library (a tissue we did not test), and RnWnk4L was found in a screen of cDNAs from regenerating liver [99]. Together, these data suggest that these gene products are highly regulated and/or cell-type specific and will not have any housekeeping function. This indicates that, unlike the ubiquitously expressed Vps25 family, which is expected to have a basic cellular role, the longer VTF proteins could have specific roles. Alternatively, the long splice variants may be artifacts of cDNA library creation, and may not be biologically relevant. Further investigations are needed to characterise the expression and function of VTF-domain mRNAs, as currently their role remains unclear.

Figure 10

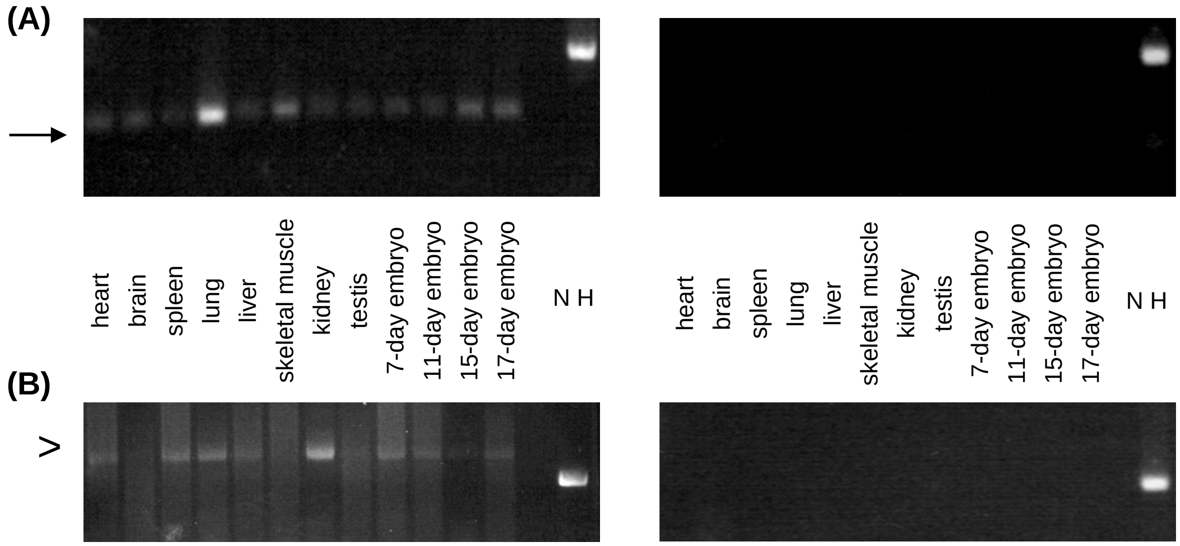
(A)
heart
brain
spleen
lung
liver
skeletal muscle
kidney
testis
7-day embryo
11-day embryo
15-day embryo
17-day embryo
N H
heart
brain
spleen
lung
liver
skeletal muscle
kidney
testis
7-day embryo
11-day embryo
15-day embryo
17-day embryo
N H
(B)
