# Supplementary material for: Genetic structure and evolution of the Vps25 family, a yeast ESCRT-II component
Source: BMC Evol Biol. 2006 Aug 4;6:59. doi: 10.1186/1471-2148-6-59 (PMC1579232; doi:10.1186/1471-2148-6-59)
Supplement: Additional File 14 — Additional Table 4: VPS25 pseudogene details [file 1471-2148-6-59-S14.pdf]

Additional File 14

Additional Table 4: Identified *VPS25* pseudogenes.

Species	Genomic accession number	Chr	Spliced	Comments
**<u>CHROMALVEOLATES</u>**				
Ciliate				
Paramecium primaurelia	AF274569	-	Yes	A nonprocessed pseudogene found in a duplicated genomic region [36].
**<u>OPISTHOKONTS</u>**				
**<u>Metazoa</u>**				
**Chordata**				
Vertebrata				
Tetrapoda				
Mammalia				
Echinops telfairi	Database location: contig_646582, 1768 to 1977 (-). Genomic location: TENREC:scaffold _318986 4229 to 4438 (-)	-	No	ENSEMBL BLAST. Full length processed pseudogene, with a stop codon after amino acid number 8, and frame-shift mutations further on.
Homo sapiens	NT_019273	1	No	Processed pseudogene contains mutations, including a single frameshift mutation where first intron of *VPS25* is found.
Monodelphis domestica	Database location: contig_12669, 14791 to 14925 (+). Genomic location: ENSMODG: scaffold_6, 14332234 to 14332368 (+)	-	No	ENSEMBL BLAST. Processed pseudogene has many mutations, including frameshift, internal stop codons, and lacks a stop codon at end of putative coding region.
Pan troglodytes (PS-1)	NW_101744	1	No	The first *P. troglodytes* pseudogene has a frameshift error where the first intron would've originally been, and another around where the last intron would've been. The sequence corresponding to last exon is the least-well conserved.
Pan troglodytes (PS-2)	NW_103538	2A	No	The second *P. troglodytes* pseudogene is an intronless full-length homolog 99% identical to *H. sapiens VPS25* at the nucleotide level, with no frameshift (or other) mutations. The full length sequence of the comparative *P. troglodytes VPS25* equivalog is not known.
