# Supplementary material for: Genetic structure and evolution of the Vps25 family, a yeast ESCRT-II component
Source: BMC Evol Biol. 2006 Aug 4;6:59. doi: 10.1186/1471-2148-6-59 (PMC1579232; doi:10.1186/1471-2148-6-59)
Supplement: Additional File 15 — Additional Figure 11: FASTA format of VPS25 pseudogenes [file 1471-2148-6-59-S15.pdf]

Additional File 15

Additional Figure 11

FASTA format of *VPS25* pseudogenes.

Nucleotide sequences are provided using standard single-letter abbreviations for bases.

```
>PprimVPS25PS
ATGTAGCATTTACTTGAATTCATTTGAATACACTTACTTATTAATAATCTATATTAAAAT
GTAGGATGCTTAAGACAAATTAAAATTACATTCAAAACTCTTAAAATATCCTCCATTTTA
CACATACATTCATTAACTCAAGTATATAGCTTATAAGATCATAAAGAAACAAGAAAGAAA
CAAATAACTTAATGGGGTGAGATTGTCCATCTCTACTTTCAATCACATAAAGTTTTGGAA
TCCTTAAATTAGAGAAATCCTTAATTTTCCCATTTTTTAAGATTCATCTTCTGGGACCAT
TAAGAGATTAGATTCTCCAGAAATCAAAGAAATACCCAACCAAACGGCACAATCAGGATC
CATAGAATGGAAAAATGATTAAAATTTCTCTGTGAATTTGGTTAGTCCTTTCGAATTAGC
AGATGCAATCTTTGCTTGGGCTAAGGAGAAAAAATTGATTGGTTACACAGAGACATTATG
AGGCATTACTGAAGGAAGTCAGACTGATTAAACTAAAAGTATTATTGTACATATATATAT
AGATTTTATAATTTACCTTAAGAATAAATTCTCAAAGCATGCTTAATTTTAGAAGAAACT
GGGAGGTTTTAAGTATATGATTTGATGGTTTATATAGTATCAAATTTATATGA
>EtelfVPS25PS
ATGGCGATGGGTTTCGAGTGGCCGTAGCAGGAGAGCTTCCCACCCTCCTTTACGTTACAG
CCGAACGTGGACACCCGGCAGAAGCAGCTGGCCGCCTGGTGCTCCCTGGTCCTGTCCTTC
TGCCGCCTGCACAAACAGTCCTGCATGACGGTGATGGAAGCGCAGGAGAGCCCGCTCTTC
CAACAACGTCAAGCTGCAGCGGAAGCTCCCTGTGGAATCCATTCAGATTGTATTAGAGGA
ACTGAGGGAGAAAGGGAACCTTGAGTGGTTGGATAAGAGAAGGTCTAGCTTCCTAATCAT
GTGGCGGAGGCCGCAAGAATGGGGGAAACTCATCCATCAGTGGGTTTCAAAAAGTGGCCA
AAACAACTCCGTGTTCACCCTGTATGAACTGACCAATGGGGAAGACACAGAGGACGAGGA
GTTCCACAGGCTGGATGAGGCGACCCTGCTGCGTGCCCTGCAGGCCCTCCAGCAGGAGCA
CAAGGCCGAGATCATCGCCACCGGCAGCAGCGACGCGAGGCGTCAAATTCTTCTAG
>MdomeVPS25PS
CTGGCAATGAGTTTCTAATGGCCATTGTGGCACCACTTCTTTCCCCCTTT
TTCACATTGCAGCCAAACATGGGTACCACCTGGCACTCTCTGGTCTTCTCTTTCTGCCAT
CTGTGTAGACTATCCAGAATGACAGTAGTAGAGACACAACAATGCCCACTTTTTCTCACC
AATAAAAAAGAAACTCCCCCAAGAAATCTAACTAGGTAGTGATGGAGGAACTGCTTCTTC
ATCATGTGAGAAAAGACCTGAAGAGTGGGGGAAGTTCATCTACCAATGGGGACCCAGCCC
CCTTCATCAGGCACATGAAAAGCTGTGGTCTGGATTTTGCTGTGGTTTTCAGAGAAGAGA
CAGAATAACTGGGGATTTCCATCCCTTATGAACTGACCAATGGAGGTGACACACAGGACA
AGGACTTCTATGGCAGAGATTAGGCAAGGCTTCCTGGAACTCTGCAGGCTCTATAGATGG
AGCCCAGGGCTAAGATCATTCCAGTGAGCCACAGCAAAGGGATCAAAGTTCTTTTTGCA
>PtVPS25PS-1
ATGGTGATGAATTTCAAGTGGTTGTGGCAGTATCGCTTTCCCGCCCTTTACATT
ACAGCTGAACGTGGCCACTTGGCAGAAGCAGCTGGCCACCTGGTGTTCGTTGGTTCTGTC
CATCTGCTGCCTGCACAGACAGTCCAGCATGATGGTTATGGATGCTCAGGAGATCCTGCT
CTTCAGCAACATCAAGCTGTGGAAGCTTCCTGTGGGATCAATCCAGGTTGTATTAGAGGA
ACTGAGGAAGAATGGGAACTTACAGTGGCTGGATAAGAGCAAGTCTAGTTTCCTAATCAT
GTGGCGGAGGCCAGAAGAATGGGGAAAACTCATCTATCAGTGGGTCTCCAGGAGTGGCCA
GAACAACTCCGTACTCAGCCTGTATGAGCTGACCAATGGGGAAGACATAGAGAATGAGGT
GTTCCACGGACTAAAGGAGGCCTTCTGTGGGCTCTGCAGGCCCTTCAGTAGGAACATAAG
GCTGAGATCATCACCATCTCACTCGGAGACCAGTGATGGCTGAGGTGTTGGCTGAGGTGT
>PtVPS25PS-2
ATGGCGATGAGTTTCGAGTGGCCGTGGCAGTATCGCTTCCCACCCTTCTTTA
CGTTACAACCGAATGTGGACACTCGGCAGAAGCAGCTGGCCGCCTGGTGCTCGCTGGTCCTGTCCTTCTG
CCGCCTGCACAAACAGTCCAGCATGACGGTGATGGAAGCTCAGGAGAGCCCGCTCTTCAACAACGTCAAG
CTACAGCGAAAGCTTCCTGTGGAGTCGATCCAGATTGTATTAGAGGAACTGAGGAAGAAAGGGAACCTCG
AGTGGTTGGATAAGAGCAAGTCCAGCTTCCTGATCATGTGGCGGAGGCCAGAAGAATGGGGGAAACTCAT
CTATCAGTGGGTTTCCAGGAGTGGCCAGAACAACTCCATCTTTACCCTGTATGAACTGACTAATGGGGAA
GACACAGAGGATGAGGAGTTCCATGGGCTGGATGAAGCCACTCTACTGCGGGCTCTGCAGGCCCTACAGC
AGGAGCACAAGGCCGAGATCATCACCGTCAGCGATGGCCGAGGTGTCAAGTTCTTCTAG
>HsapiVPS25PS
ATGGTGACGAATTTCAAGTGGTTGTGGCAGTATCGCTTTCCCGCCCTTTACATTACAGCTGAACGTGGCC
ACTTGGCAGAAGCAGCTGGCCACCTGGTGTTTGTTGGTTCTGTCCATCTGCTGCCTGCACAGACAGTCAA
GCATGATGGTTATGGATGCTCAGGAGATCCTGCTCTTCAGCAACATCAAGCTGTGGAAGCTTCCTGTGGG
ATCAATCCAGGTTGTATTAGAGGAACTGAGGAAGAATGGGAACTTACAGTGGCTGGATAAGAGCAAGTCT
AGTTTCCTAATCATGTGGCGGAGGCCAGAAGAATGGGGAAAACTCATCTATCAGTGGGTCTCCAGGAGTG
GCCAGAACAACTCCGTACTTAGCCTGTATGAGCTGACCAATGGGGAAGACATAGAGAATGAGGTGTTCCA
CGGACTAAAGGAGGCCTTCTGTGGGCTCTGCAGGCCCTTCAGTAGGAACATAAGGCTGAGATCATCACCA
TCTCACTCGGAGACCAGTGATGGCTGAGGTGTCAGGTCCATCTGT
```
