# Supplementary material for: Genetic structure and evolution of the Vps25 family, a yeast ESCRT-II component
Source: BMC Evol Biol. 2006 Aug 4;6:59. doi: 10.1186/1471-2148-6-59 (PMC1579232; doi:10.1186/1471-2148-6-59)
Supplement: Additional File 16 — Additional Figure 12: Alignment of mammalian VPS25 coding sequences with pseudogene sequences [file 1471-2148-6-59-S16.pdf]

Additional File 16

Additional Figure 12

Alignment of mammalian *VPS25* coding sequences with processed pseudogene sequences.

The complete coding sequence of *H. sapiens VPS25*, the almost complete sequence of *Pan troglodytes VPS25*, and the coding sequence of *Monodelphis domestica Vps25* were compared to their pseudogenes: *HsVPS25PS*, *PtVPS25PS-1*, *PtVPS25PS-2*, and *MdVps25PS*. Pseudogenes for which there is currently no accurate parental DNA sequence (i.e. *M. domestica, E. telfairi*) were not included. As the parental *P. troglodytes* sequence was incomplete, the coding sequence of *Macaca mulatta VPS25* was included for comparison. Sequences were aligned using Multalin and shaded using Boxshade, where identical nucleotides are in black boxes. 'N's indicate a lack available sequence, and dashes represent gaps introduced to provided optimal alignment.

```
Hsapiens          1 ATGGCGATGAGTTTCGAGTGGCCGTGGCAGTATCGCTTCC--CACCCTTCTTTACGTTACAACCGAATGTGGACACTCGG
Ptroglodytes      1 ATGGCGATGAGTTTCGAGTGGCCGTGGCAGTATCGCTTCC--CACCCTTCTTTACGTTACAACCGAATGTGGACACTCGG
Mmulatta          1 ATGGCGATGAGTTTCGAGTGGCCGTGGCAGTATCGCTTCC--CACCCTTCTTTACGTTACAACCGAATGTGGACACTCGG
HsVPS25PS         1 ATGGTGACGAATTTCAAGTGGTTGTGGCAGTATCGCTTTC--CCGCC--CTTTACATTACAGCTGAACGTGGCCACTTGG
PtVPS25PS_1       1 ATGGTGATGAATTTCAAGTGGTTGTGGCAGTATCGCTTTC--CCGCC--CTTTACATTACAGCTGAACGTGGCCACTTGG
PtrogVPS25PS_2    1 ATGGCGATGAGTTTCGAGTGGCCGTGGCAGTATCGCTTCC--CACCCTTCTTTACGTTACAACCGAATGTGGACACTCGG
Mdomestica        1 ATGGCGACGAGTTTCGAGTGGCCGTGGCAATACCGATTCC--CTCCTTTCTTCACGTTACAGCCGAACGTGGACACCCGG
MdVps25PS         1 ------ATGAGTTTCTAATGGCCATTGTGGCACCACTTCTTTCCCCCTTTTTCACATTGCAGCCAAACATGGGTAC----

Hsapiens         79 CAGAAGCAGCTGGCCGCCTGGTGCTCGCTGGTCCTGTCCTTCTGCCGCCTGCACAAACAGTCCAGCATGACGGTGATGGA
Ptroglodytes     79 CAGAAGCAGCTGGCCGCCTGGTGCTCGCTGGTCCTGTCCTTCTGCCGCCTGCACAAACAGTCCAGCATGACGGTGATGGA
Mmulatta         79 CAGAAGCAGCTGGCCGCCTGGTGCTCGCTGGTCCTGTCCTTCTGCCGCCTGCACAAACAGTCCAGCATGACGGTGATGGA
HsVPS25PS        77 CAGAAGCAGCTGGCCACCTGGTGTTTGTTGGTTCTGTCCATCTGCTGCCTGCACAGACAGTCAAGCATGATGGTTATGGA
PtVPS25PS_1      77 CAGAAGCAGCTGGCCACCTGGTGTTCGTTGGTTCTGTCCATCTGCTGCCTGCACAGACAGTCCAGCATGATGGTTATGGA
PtVPS25PS_2      79 CAGAAGCAGCTGGCCGCCTGGTGCTCGCTGGTCCTGTCCTTCTGCCGCCTGCACAAACAGTCCAGCATGACGGTGATGGA
Mdomestica       79 CAAAAGCAGCTGGCGGCCTGGTGTTCTCTGGTCCTGTCCTTCTGCCGCCTGCACCGACAGTCCAGCATGACGGTGATGGA
MdVps25PS        71 --------------CACCTGGCACTCTCTGGTCTTCTCTTTCTGCCATCTGTGTAGACTATCCAGAATGACAGTAGTAGA

Hsapiens        159 AGCTCAGGAGAGCCCGCTCTTCAACAACGTCAAGCTACAGCGAAAGCTTCCTGTGGAGTCGATCCAGATTGTATTAGAGG
Ptroglodytes    159 AGCTCAGGAGAGCCCGCTCTTCAACAACGTCAAGCTACAGCGAAAGCTTCCTGTGGAGTCGATCCAGATTGTATTAGAGG
Mmulatta        159 AGCTCAGGAGAGCCCGCTCTTCAACAACGTCAAGCTACAGCGAAAGCTTCCTGTGGAGTCGATCCAGATTGTATTAGAGG
HsVPS25PS       157 TGCTCAGGAGATCCTGCTCTTCAGCAACATCAAGCT---GTGGAAGCTTCCTGTGGGATCAATCCAGGTTGTATTAGAGG
PtVPS25PS_1     157 TGCTCAGGAGATCCTGCTCTTCAGCAACATCAAGCT---GTGGAAGCTTCCTGTGGGATCAATCCAGGTTGTATTAGAGG
PtVPS25PS_2     159 AGCTCAGGAGAGCCCGCTCTTCAACAACGTCAAGCTACAGCGAAAGCTTCCTGTGGAGTCGATCCAGATTGTATTAGAGG
Mdomestica      159 GGCACAAGAAAGCCCGCTCTTCAACAACAATAAGCTGCAGAGGAAGCTTCCTATGGAATCTATCCAGATTGTGTTGGAGG
MdVps25PS       137 GACACAACAATGCCCACTTTTTCTCACCAATAAAAA----GAAACTCCCCCAAGAAATCTAACTAGGTAGTGATGGAGG

Hsapiens        239 AACTGAGGAAGAAAGGGAACCTCGAGTGGTTGGATAA--GAGCAAGTCCAGCTTCCTG-----ATCATGTGGCGGAGGCC
Ptroglodytes    239 AACTGAGGAAGAAAGNNNNNNNNNNNTGGTTGGATA---GAGCAAAGTCAGCTTTCTGG----ATCATGTGGCGAGGGCA
Mmulatta        239 AACTGAGGAAGAAAGGGAACCTCGAGTGGTTGGATAA--GAGCAAGTCTAGCTTCCTG-----ATCATGTGGCGGAGGCC
HsVPS25PS       234 AACTGAGGAAGAATGGGAACTTACAGTGGCTGGATAA--GAGCAAGTCTAGTTTCCTA-----ATCATGTGGCGGAGGCC
PtVPS25PS_1     234 AACTGAGGAAGAATGGGAACTTACAGTGGCTGGATAA--GAGCAAGTCTAGTTTCCTA-----ATCATGTGGCGGAGGCC
PtVPS25PS_2     239 AACTGAGGAAGAAAGGGAACCTCGAGTGGTTGGATAA--GAGCAAGTCCAGCTTCCTG-----ATCATGTGGCGGAGGCC
Mdomestica      239 AACTAAGGAAGAAAGGGAACCTGGAGTGGTTGGATAA--GAACAAGTCCAGCTTCTTG-----ATCATGTGGCGGAGGCC
MdVps25PS       213 AACTGCTTCTTCATCATGTGAGAAAAGACCTGAAGAGTGGGGGAAGTTCATCTACCAATGGGGACCCAGCCCCCTTCATC

Hsapiens        312 AGAAGAATGGGGGAAACTCATCTAT------CAGTGGGTTTCCAGGAGTGGCCAGAACAACTCCGTCTTT--ACCCTGTA
Ptroglodytes    312 GAGAAAATGGGGGAAACTCATCTAT------CAGTGGGTTTCCAGGAGTGGCCAGAACAACTCCGTCTTT--ACCCTGTA
Mmulatta        312 AGAAGAATGGGGGAAACTCATCTAT------CAGTGGGTTTCCAGGAGTGGCCAGAACAACTCCGTCTTT--ACCCTGTA
HsVPS25PS       307 AGAAGAATGGGGAAAACTCATCTAT------CAGTGGGTCTCCAGGAGTGGCCAGAACAACTCCGTACTT--AGCCTGTA
PtVPS25PS_1     307 AGAAGAATGGGGAAAACTCATCTAT------CAGTGGGTCTCCAGGAGTGGCCAGAACAACTCCGTACTC--AGCCTGTA
PtVPS25PS_2     312 AGAAGAATGGGGGAAACTCATCTAT------CAGTGGGTTTCCAGGAGTGGCCAGAACAACTCCATCTTT--ACCCTGTA
Mdomestica      312 AGAAGAATGGGGGAAACTCATCTAC------CAGTGGGTTTCAAAGAGTGGCCAGAACAACTCTGTATTT--ACCTTTTA
MdVps25PS       293 AGGCACATGAAAAGCTGTGGTCTGGATTTTGCTGTGGTTTTCAGAGAAGAGACAGAATAACTGGGGATTTCCATCCCTTA

Hsapiens        384 TGAACTGACTAATGGGGAAGACACAGAGGATGAGGAGTTCCACGGGCTGGATGAAGCCACTCTACTGCGGGCTCTGCAGG
Ptroglodytes    384 TGAACTGACTAATGGGGAAGACACAGAGGATGAGGNNNNNNNNNNNNNNNNNNNNNNNNNNNNNNNNNNNNNNCTGCAGG
Mmulatta        384 TGAACTAACTAACGGGGAAGACACAGAGGATGAGGAGTTCCATGGGCTGGATGAGGCCACTCTACTGCGGGCTCTGCAGG
HsVPS25PS       379 TGAGCTGACCAATGGGGAAGACATAGAGAATGAGGTGTTCCACGGACTAAAGGAGGCC----TTCTGTGGGCTCTGCAGG
PtVPS25PS_1     379 TGAGCTGACCAATGGGGAAGACATAGAGAATGAGGTGTTCCACGGACTAAAGGAGGCC----TTCTGTGGGCTCTGCAGG
PtVPS25PS_2     384 TGAACTGACTAATGGGGAAGACACAGAGGATGAGGAGTTCCATGGGCTGGATGAAGCCACTCTACTGCGGGCTCTGCAGG
Mdomestica      384 TGAACTAACCAATGGAGATGATACCGAGGATGAGGAATTCCATGGGCTGGATGAAGCCACACTTCTTCGAGCTCTGCAGG
MdVps25PS       373 TGAACTGACCAATGGAGGTGACACACAGGACAAGGACTTCTATGGCAGAGATTAGGCAAGGCTTCCTGGAACTCTGCAGG

Hsapiens        464 CCCTACAGCAGGAGCACAAGGCCGAGATCATCACTGTC-------------AGCGATGGCCGAGGCGTCA-AGTTCTTCT
Ptroglodytes    464 CCCTACAGCAGGAGCACAAGGCCGAGATCATCACCGTC-------------AGCGATGGCCGAGGTGTCA-AGTTCTTCT
Mmulatta        464 CACTACAGCAGGAACACAAGGCCGAGATCATCACCGTC-------------AGCGATGGCCGAGGCGTCA-AGTTCTTCT
HsVPS25PS       455 CCCTTCAGTAGGAACATAAGGCTGAGATCATCACCATCTCACTCGGAGACCAGTGATGGCTGAGGTGTCA-GGTCCATCT
PtVPS25PS_1     455 CCCTTCAGTAGGAACATAAGGCTGAGATCATCACCATCTCACTCGGAGACCAGTGATGGCTGAGGTGTT--GGCTGAGGT
PtVPS25PS_2     464 CCCTACAGCAGGAGCACAAGGCCGAGATCATCACCGTC-------------AGCGATGGCCGAGGTGTCA-AGTTCTTCT
Mdomestica      464 CCCTACAGCTGGAACACAAGGCTGAGATCATCACTGTC-------------AGCGATGGCCGAGGGGTCA-AATTTTTTT
MdVps25PS       453 CTCTATAGATGGAGCCCAGGGCTAAGATCATTCCAGTG-------------AGCCACAGCAAAGGGATCAAAGTTCTTTT

Hsapiens        530 AG--
Ptroglodytes    530 AG--
Mmulatta        530 AG--
HsVPS25PS       534 GT--
PtVPS25PS_1     533 GT--
PtVPS25PS_2     530 AG--
Mdomestica      530 AA--
MdVps25PS       520 TGCA
```
