# Supplementary material for: Genetic structure and evolution of the Vps25 family, a yeast ESCRT-II component
Source: BMC Evol Biol. 2006 Aug 4;6:59. doi: 10.1186/1471-2148-6-59 (PMC1579232; doi:10.1186/1471-2148-6-59)
Supplement: Additional File 17 — Additional Figure 13: Genomic context of primate VPS25 pseudogenes [file 1471-2148-6-59-S17.pdf]

Additional File 17

Additional Figure 13

Genomic context of primate *VPS25* pseudogenes.

(A) The *Homo sapiens* pseudogene *HsVPS25PS* localizes to chromosome 1 map position 1p12. Genes localising to region 117398257-117830187 bp (arrowheads) on Build 35.1 are shown. *HsVPS25PS* is shown as a black arrow (*LOC44189)* and the surrounding genes as gray arrows, with the arrow pointing to the orientation on the genome. The surrounding genes on the plus strand include the *MAN1A2* and *FAM46C* genes, encoding alpha1,2-mannosidase and family with sequence similarity 46, member C, respectively. On the opposite strand are the B7-H4 gene, encoding the immune costimulatory protein VTCN1, and *LOC401959*, which is a voltage-dependent anion channel 2 pseudogene.

(B) The *Pan troglodytes VPS25PS*-1 is located on chromosome 1, region 4064976-4083747 bp (arrowheads), Build 1.1. *PtVPS25PS-1* is shown as a black arrow (*LOC469434*) and the surrounding genes as gray arrows, with the arrow pointing to the orientation on the genome. The surrounding genes include *MAN1A2* genes, encoding alpha1,2-mannosidase also the positive strand. On the opposite strand is *LOC469433* encoding an ortholog of human immune costimulatory protein B7-H4, and a pseudogene (*LOC469435*) similar to the voltage-dependent anion channel-2.

(C) The second *P. troglodytes* pseudogene, *PtVPS25PS*-2, is located on chromosome 2A in region 739052-739842 bp (arrowheads). *PtVPS25PS-2* is

shown as a black arrow and the surrounding genes as gray arrows, with the arrow pointing to the orientation on the genome. *PtVPS25PS-2* overlaps *LOC459431*, as annotated on the NCBI database. This has been proposed (on the database) to encode a spliced mRNA and not a *VPS25* pseudogene. However, the proposed protein (XP_530480) is not conserved in any other eukaryotic species, and has not been included in our diagram. All genes surrounding *PtVPS25PS*-2 are on the opposite strand, and include *LOC459429*, which encodes a protein with a partial interalpha trypsin inhibitor (ITI) domain; *LOC459430*, which encodes a protein with an ITI and ATP-synthase domains; *LOC470447*, similar to cyclic-nucleotide-gated cation channel alpha 3 (CNG3); and *LOC470448*, similar to inositol polyphosphate-4-phosphate (type 1, isoform b). Gene names are above the corresponding arrows. Drawings are approximately to scale (see scale bar).

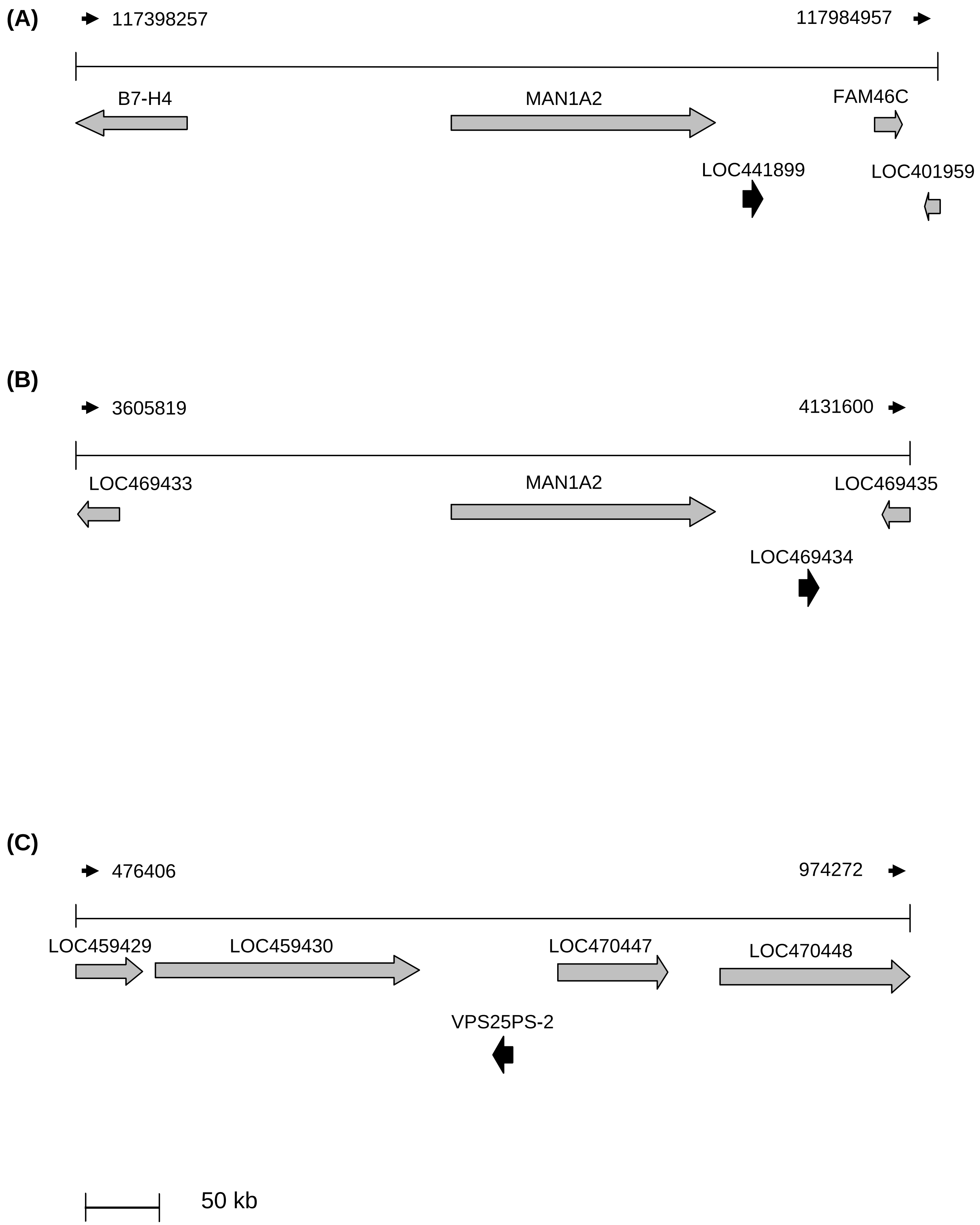
(A)
117398257
117984957
B7-H4
MAN1A2
FAM46C
LOC441899
LOC401959
(B)
3605819
4131600
LOC469433
MAN1A2
LOC469435
LOC469434
(C)
476406
974272
LOC459429
LOC459430
LOC470447
LOC470448
VPS25PS-2
50 kb
