# Supplementary material for: Genetic structure and evolution of the Vps25 family, a yeast ESCRT-II component
Source: BMC Evol Biol. 2006 Aug 4;6:59. doi: 10.1186/1471-2148-6-59 (PMC1579232; doi:10.1186/1471-2148-6-59)

Additional File 18

Additional Figure 14 - Comparison of full-length Vps25 amino acid sequences

Multiple alignments were generated using ClustalX. A consensus amino acid sequence is found below the alignment, where uppercase letters represent the four totally conserved amino acids. Shading was done using Boxshade, where regions of greater than 50% conservation are shaded (identical amino acids are in black boxes and similar amino acids in gray boxes). An asterisk indicates a highly conserved lysine close to the carboxy-terminus. Protein accession numbers are found in Additional File 1. Gaps required for optimal alignment are indicated by dashes. Standard single letter amino acid abbreviations are used. The secondary structure elements of yeast Vps25p are provided beneath the alignment. Prior to the first winged helix (WH domain) are two 3/10 helices (η1 and η2), while WH-1 consists of the requisite pattern of alpha (α) helices and beta (β) strands, with a further uncharacteristic beta strand (Uβ) shaded in gray found in the unliganded structure [20]. WH-2 is more typical of a WH domain and follows more closely the helix (H1)- beta (B1)-H2-H2-B2-B3 format. Disordered regions in the yeast Vps25p structure are indicated by a dashed line. Introns I-III in the human *VPS25* gene are found after η2, α2, and α3 of WH1, while intron IV interrupts H1, and intron V is found prior to H3, in WH-2.

```
Theileria annulata             MDSVDF----------------------------------------------------------------NTHVKFKNFPPLYTEQI---NNLTLSKQLE
Theileria parva                MDSVDF----------------------------------------------------------------NTHEKFKNFPPLYTEQI---NNLTLSKQLE
Paramecium primaurelia         MWHYLNSFEYTYLLIIYIKIITFKTLEI------------------------------------------SSILHVHSLSQVYSLQD---HKETRKKQIT
Leishmania brazilia            MS--------------------------------------------------------------------SEHWSFFGLPPFFTEQH---SPATLDRQST
Leishmania infantum            MP--------------------------------------------------------------------SEHWSFFGLPPFFTEQH---SPATLDRQCT
Leishmania major               MS--------------------------------------------------------------------SEHWVFFGLPPFFTEQH---SSATLDRQCT
Trypanosoma brucei             MSKE------------------------------------------------------------------PGHWDFFKLPPFFTLQP---SPSALERQMA
Trypanosoma b.gambiense        MSKE------------------------------------------------------------------PGHWDFFKLPPFFTLQP---SPSALERQMA
Trypanosoma congolese          MPISVPLLLLAMLWLLPPQLLHNFLSFFLLLVHFIYRAPNFPWTSEAASTRGGRGVTQSRSLALAPMAKEPSHWDFFKLPPFFTLQP---APAALERQLM
Trypanosoma cruzi              MSIE------------------------------------------------------------------PDHWSFFQLPPFFALQP---GPTALARQTS
Trypanosoma vivax              MTVE------------------------------------------------------------------PNHWNFFKLPPFFTMQP---AAAALARQVT
Giardia lamblia                MACKDV----------------------------------------------------------------LESSELWYFPPFFTLQQKIEQADVCTEQLK
Trichomonas vaginalis 1        MSKKKATKKPPT----------------------------------------------------------FEFPDFYDYPPFWTLQT---NSQTKKQQLD
Trichomonas vaginalis 2        MS--------------------------------------------------------------------FKFPPIYSFPPFWTIQP---CMEARRMQTQ
Entamoeba histolytica          MT--------------------------------------------------------------------FGIPEFAKFPPFYTIQL---VDKTKNQQLQ
Dictyostelium discoideum       MSNQV-----------------------------------------------------------------FQFPPYYHKEPFFTIQP---ILNTRKKQFQ
Chlamydomonas reinhardtii      MGS-------------------------------------------------------------------FAFPYFHNYPPYFTLQP---VKETRDKQVA
Cyanidioschyzon merolae        MLIRTTPVQETGNHQPKTEPAS------------------------------------------------FAFPPWHQYPPLYTIQP---CARTRERQLY
Physcomitrella patens          MGE-------------------------------------------------------------------FSFPYFYNYPPYFTLQP---MKDTRDKQIQ
Welwitschia mirabilis          MNPKNMGE--------------------------------------------------------------FRLPDFFNYPPYFTLQP---VRDTRQKQVQ
Pinus taeda                    MAD-------------------------------------------------------------------LKLPDFFNYPPYFTLQP---VRDTREKQVQ
Asparagus officinalis          MQRLGD----------------------------------------------------------------FRLPHFFNYPPYFTLQP---VRETREKQVQ
Hordeium vulgare               MQRTVD----------------------------------------------------------------FKLPHFFNYPPYFTLQP---VRETREKQVQ
Oryza sativa                   MQRLGD----------------------------------------------------------------FRLPPFFNYPPYFTLQP---VRETREKQVQ
Sorghum bicolor                MQRLGD----------------------------------------------------------------FRLPPFFNYPPYFTLQP---VRETREKQVQ
Saccharum officinarum          MQRLGD----------------------------------------------------------------FRLPPFFNYPPYFTLQP---VRETREKQVQ
Triticum aestivum              MQRTVD----------------------------------------------------------------FKLPHFFNYPPYFTLQP---VRETREKQVQ
Antirrhinum majus              MQTLGE----------------------------------------------------------------FKLPHFFNYPPYFTLQP---VRDTREKQIQ
Coffea canephora               MQKLGE----------------------------------------------------------------FKLPHFFNYPPYFTLQP---VRDTREKQVQ
Lycopersicon esculentum        MQKLGE----------------------------------------------------------------FKLPNFFNYPPYFTLQP---VRETREKQIQ
Arabidopsis thaliana           MQKLAD----------------------------------------------------------------FKLPQFFNYPPYFTLQP---VRDTREKQIQ
Brassica napus                 MQKLGD----------------------------------------------------------------FKLPQFFNYPPYFTLQP---VRDTREKQIQ
Citrus clementina              MQKLGD----------------------------------------------------------------FKLPAFFNYPPYFTLQP---VRDTREKQIQ
Fragaria vesca                 MQKLGD----------------------------------------------------------------YKLPQFFNYPPYFTLQP---VRDTREKQIQ
Gossypium hirsutum             MQKLGD----------------------------------------------------------------FKLPHFFNYPPYFTLQP---VRETREKQIQ
Glycine max                    MQKLGE----------------------------------------------------------------FKLPPFFNYPPYFTLQP---VRDTREKQIQ
Malus x domestica              MQKLGD----------------------------------------------------------------FKLPQFFNYPPYFTLQP---VRDTREKQIQ
Medicago truncatula            MQKLGE----------------------------------------------------------------FKLPHFFNYPPYFTLQP---VRDTREKQIQ
Populus trichocarpa            MQKLGD----------------------------------------------------------------FKLPQFFNYPPYFTLQP---VRDTREKQVQ
Populus tremula                MQKLGD----------------------------------------------------------------FKLPQFFNYPPYFTLQP---VRDTREKQVQ
Vitis vinifera                 MQKLGD----------------------------------------------------------------LKLPNFFNYPPYFTLQP---VRDTREKQVQ
Candida albicans               MSDPILQ---------------------------------------------------------------FEFPKIYSFPPFYTQQP---NTTVLNQQLD
Candida glabrata               M------------------------------------------------------------------------PSIYAFPPLYTRQP---NSLVRKQQID
Clavispora lusitaniae          M---------------------------------------------------------------------FEFPKIHSFPPFYTKQR---NATILENQLE
Debaryomyces hansenii          MTTV------------------------------------------------------------------FEFPKIHSFPPLYTKQP---NLTILHNQLE
Eremothecium gossypii          MAMA------------------------------------------------------------------SELPSIYNFPPLYTRQP---NSLIRAQQLD
Kluveromyces lactis            MRKGDPALTLSSSIRIHHSETETFINIGMT----------------------------------------LETPQIYKFPPLYTPQT---NKLIRKQQLQ
Kluveromyces waltii            MT--------------------------------------------------------------------EKLPPIYNFPPLYTLQP---NVLIREQQLN
Saccharomyces cerevisiae       M---------------------------------------------------------------------SALPPVYSFPPLYTRQP---NSLTRRQQIS
Saccharomyces bayanus          MM--------------------------------------------------------------------ASLPPVYSFPPLYTRQP---NSLTRRQQLS
Saccharomyces castellii        M---------------------------------------------------------------------QPLPPIYSFPPLYTRQP---NSIIRNQQLN
Saccharomyces kluyveri         MN--------------------------------------------------------------------KQLPPIYSFPPLYTRQP---NALIRQQQLD
Saccharomyces kudriavzevii     M---------------------------------------------------------------------ASLPPVYSFPPLYTRQP---NSLTRRQQIS
Saccharomyces mikatae          M---------------------------------------------------------------------ASLPPIYSFPPLYTRQP---NSLTRRQQIS
Saccharomyces paradoxus        M---------------------------------------------------------------------TALPPVYSFPPLYTRQP---NSLTRRQQIS
Yarrowia lipolytica            M-----------------------------------------------------------------------NSEIYNFPPFFTRQP---NETTWQAQLS
Schizosaccharomyces pombe      M----------------------------------------------------------------------RVPSIYNFPPFFTRQL---NDNTWHSQKA
Aspergillus fumigatus          MSQSQSQSQSHSTTT-------------------------------------------------------FQFPPTYSFPPFFTRQP---NSTTRLSQLQ
Aspergillus oryzae             MSNSTP----------------------------------------------------------------FQFPPTYSFPPFFTPQP---NSTTRLSQLQ
Botryotinia fuckeliana         MSTSQTQTSVKI----------------------------------------------------------FKFPREHSFPPFFTLQP---TSSTVHAQLR
Coccidioides immitis           MPPLTSHPATPTPAEQNT----------------------------------------------------FPFPAPHSFPPFFTLQP---NAQTLLSQLQ
Gibberella zeae                MAATTTTSAPADT---------------------------------------------------------FKFPREYHFPAFFTRQT---NLTTLHAQHN
Magnoporthe grisea             MPPSTEP---------------------------------------------------------------FPFPREYHFPPFFTRQT---NLTTHHAQLT
Neosartorya fischeri           MSQTQSHPTTT-----------------------------------------------------------FQFPPTYSFPPFFTPQP---NSTTRLSQLQ
Neurpospora crassa             MSTTSPAPSDSSTPNNSNPKTTASTLLPNDT---------------------------------------FPFPREFFFPPFFTRQT---NLTTHHAQLT
Phaesosphaeria nodorum         MATASPLPSNFSTSSTLPPTTTQPAASTG-----------------------------------------FQFPPHYSFPPFFTLQP---TASTRSSQLL
Sclerotinia sclerotiorum       MTPQTITPAKT-----------------------------------------------------------FKFPREHSFPPFFTLQP---TSATLHAQLR
Trichoderma reesei             MATTAATPSPTPPTSTSTSTPTTTTG--------------------------------------------FAFPREYSFPPFFTRQP---NIAIHHAQLT
Uncinocarpus reesii            MATTPSLLAAPAPTPQQPPSAADT----------------------------------------------FAFPAPHSFPPFFTLQP---NTQTLLSQLQ
Coprinus cinereus              MSLSTHTTPSGSSSVLDFWSSTSDLTQHAHCLG-------------------------------------YLLPSIHSAPPFFTQQP---NPSTQGIVVE
Phanerochaete chrysosporium    MAHVPSKDEVAGTEESIG----------------------------------------------------YLLPSIHSAPPFFTQQP---NPNTQAVVTE
Ustilago maydis                MAATASVTPSVKGSSPAAGASPIASAGMAEGGS-------------------------------------FRYPPIHAFPPFFTLQH---NPVSRAQQLS
Rhizopus oryzae                MSN-------------------------------------------------------------------FELPSIFDFPPFFTRQV---TESTWKSQAF
Blastocladiella emersonii      MTSTASLNATP-----------------------------------------------------------FPFPQLYSFPPFFTLQP---HEPSRVKQVA
Strongylocentrotus purpuratus  MGN-------------------------------------------------------------------FEWPWQYEFPPFFSLQP---NLETRKKQLL
Caenorhabditis briggsae        MATTSA----------------------------------------------------------------FKWPWQYDFPPFFTIQK---SLNTKDKQLE
Caenorhabditis elegans         MAAATTTASA------------------------------------------------------------FKWPWQYDFPPFFTIQK---SLNTKDKQLE
Heterodera glycines            MS--------------------------------------------------------------------FQWPWHFDFPPFFTIQP---NLSTRDKQLK
Paragonimus westermani         MSSQK-----------------------------------------------------------------FEWPWLYNFPPFFTLQP---NAETRRKQQD
Schistosoma japonicum          MSLASEK---------------------------------------------------------------NIWPWQYRFPPFFTLQP---NAETRRKQIN
Schistosoma mansoni            MSLTPGND--------------------------------------------------------------VIWPWQYNFPPFFTLQP---NAETRRKQIN
Schistosoma mediterranea       MSN-------------------------------------------------------------------FEWPWQYNFPPFFTLQP---NAETRKKQID
Amblyomma variegatum           MTD-------------------------------------------------------------------FEWPWQYSFPPFFTLQP---TLATREKQLD
Boophilus microplus            MATD------------------------------------------------------------------FEWPWQYGFPPFFTLQP---TLATREKQLE
Aedes aegypti                  MAE-------------------------------------------------------------------YQWPWEYSFPPFFTVQS---HGGTKDQQLS
Anopheles gambiae              MGA-------------------------------------------------------------------FQWPWEYSFPPFFTVQV---HAKTKEQQLA
Apis mellifera                 MAE-------------------------------------------------------------------IEWPWQYSFPPFFTLQP---HSDTRAKQLS
Acyrthosiphon pisum            MGD-------------------------------------------------------------------VQWPWQYSFPPFFTIQP---NAETRQKQLD
Bombyx mori                    MAE-------------------------------------------------------------------ISWPWQYNFPPFFTIQP---HTETRSKQLE
Drosphila melanogaster         MAE-------------------------------------------------------------------FQWPWEYTFPPFFTLQP---HEETRQQQLK
Drosphila pseudoobscura        MTE-------------------------------------------------------------------FQWPWEYTFPPFFTLQP---HEETRQQQLK
Lutzomyia longipalpis          MGE-------------------------------------------------------------------FEWPWEYNFPPFFTIQP---HDKTREHQLK
Ciona intesinalis              MGTTSN----------------------------------------------------------------FKWPWQYDFPPFFTMQK---NADTRFKQTE
Molgula tectiformis            MPQE------------------------------------------------------------------FAWPWQYDFPPFFTMQE---NADTHEKQLN
Leucoraga erinacea             MS--------------------------------------------------------------------FEWPWQYNFPPFYTLQP---NVSTQHRQLA
Danio rerio                    MS--------------------------------------------------------------------FEWPWQYNFPPFFTLQP---NVDTRQKQLA
Fugu rubripes                  MS--------------------------------------------------------------------FEWPWQYNFPPFFTLQP---NVDTRQKQLA
Gasterosteus aculeatus         MS--------------------------------------------------------------------FEWPWQYNFPPFFTLQP---NVDTRQKQLA
Ictalurus punctatus            MS--------------------------------------------------------------------FEWPWQYNFPPFFTLQP---NADTRQKQLA
Oryzias latipes                MS--------------------------------------------------------------------FEWPWQYNFPPFFTLQP---NADTRQKQLE
Oncorhynchus mykiss            MS--------------------------------------------------------------------FEWPWQYNFPPFFTLQP---NVDTRQKQLA
Platichthys flesus             MS--------------------------------------------------------------------FEWPWQYNFPPFFTLQP---NVDTRQKQLA
Pimephales promelas            MS--------------------------------------------------------------------FEWPWQYNFPPFFTLQP---NVDTRQKQLS
Salmo salar                    MS--------------------------------------------------------------------FEWPWQYNFPPFFTLQP---NVDTRQKQLA
Tetraodon nigroviridis         MS--------------------------------------------------------------------FEWPWQYNFPPFFTLQP---NVDTRQKQLA
Gallus gallus                  MS--------------------------------------------------------------------FSWPWQYSFPPFFTLQP---NGDTRQKQLA
Taeniopygia guttata            MS--------------------------------------------------------------------FAWPWQYSFPPFFTLQP---NGETRQKQLS
Xenopus laevis                 MG--------------------------------------------------------------------FEWPWQYNFPPFFTLQP---NVDTRQKQLS
Xenopus tropicalis             MG--------------------------------------------------------------------FEWPWQYNFPPFFTLQP---NVDTRQKQLS
Bos taurus                     MAMS------------------------------------------------------------------FEWPWQYRFPPFFTLQP---NVDTRQKQLA
Canis familiaris               MAMS------------------------------------------------------------------FEWPWQYRFPPFFTLQP---NVDTRQKQLA
Equus caballus                 MAMS------------------------------------------------------------------FEWPWQYRFPPFFTLQP---NVDTRQKQLA
Homo sapiens                   MAMS------------------------------------------------------------------FEWPWQYRFPPFFTLQP---NVDTRQKQLA
Macaca mulatta                 MAMS------------------------------------------------------------------FEWPWQYRFPPFFTLQP---NVDTRQKQLA
Monodelphis domestica          MATS------------------------------------------------------------------FEWPWQYRFPPFFTLQP---NVDTRQKQLA
Mus musculus                   MAMS------------------------------------------------------------------FEWPWQYRFPPFFTLQP---NVDTRQKQLA
Oryctolagus cuniculus          MAMS------------------------------------------------------------------FEWPWQYRFPPFFTLQP---NVDTRQKQLA
Rattus norvegicus              MAMS------------------------------------------------------------------FEWPWQYRFPPFFTLQP---NVDTRQKQLA
Sus scrofa                     MAMS------------------------------------------------------------------FEWPWQYRFPPFFTLQP---NVDTRQKQLA
consensus                      M                                                                     f fp  y fppfftlQp   n etr kql
```

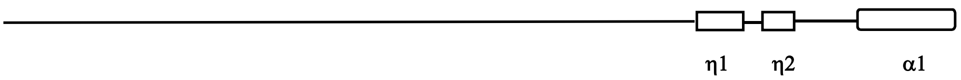

```
Theileria annulata             IWHKIINDEVITNYSLHKIGTETIN------------------------FPPFKNEE--IVRNVNVSFLALILEYLAEKQYAFYLHPIQLFCKKHNVTI
Theileria parva                IWHKIINDEVTANYSLHKLGTASVN------------------------FPPFKNEE--ILRNVDVSFLALILGYLVEKQYAFYLHPIQFFCKKNNVSI
Paramecium primaurelia         QWSEIVHLYFQSH-KILESSISEILN-----------------------FPIFQDSSSGIIKRLDSSEIKEILNQMAQL--------GSIEWKNDQ---
Leishmania brazilia            LWSNLLLDHAIYHTQRTAGGDTNPLLRFYT-------------------TNSDIFYNPA--INKRLSPEGAHTMLQALVAR------HPNHAVVVSDGG--
Leishmania infantum            LWSNLLLDHAIYHAQRTAGGDTNALLRFYT-------------------TQSDIFYNPA--IDKRLSPEAAHTMLQSLVAR------HPNHAVIVSDGG--
Leishmania major               LWSNLLLDHAIYHAQRTAGGDTNALLRFYT-------------------TQSDIFYNPA--INKRLSPEGAHTMLQSLVAR------HPNHVVIVSDGG--
Trypanosoma brucei             LWGNLVMDHAAFHAQHRKR-DTCPLLRLYS-------------------CNSGLFRNET--INRRLRPEDVKKVISSLVAQ------WSEHCVLTSDGD--
Trypanosoma b.gambiense        LWGNLVMDHAAFHAQHRKR-DTCPLLRLYS-------------------CNSGLFRNET--INRRLRPEDVKKVISSLVAQ------WSEHCVLTSDGD--
Trypanosoma congolese          LWGNLVMDHAASHAPNTRS-DACPFLRLYS-------------------CTSSLFRNDS--INRRLSSEHAKKVLSSLAVQ------RPEHCVLTGNDD--
Trypanosoma cruzi              LWGSAIIDHAAYHAPRTRP-GVCPFLRLYK-------------------STSDVFRNPS--INRRLPPEAAARMLESLVAQ------HPNHCAVVLPGNE-
Trypanosoma vivax              LWEDLIMDHAAHHARLTNR-GVCSHLRLYS-------------------STSDVFRNNA--IRRRLPPEDATKILSSLASR------RLSHCISVEDAN--
Giardia lamblia                QWDALLMKWCYEV-KSREIPKDNYLEK----------------------AIFKNTK--ILRKVSDELFREIIDWFKRK--------GRLVEINKS---
Trichomonas vaginalis 1        LWASFICAYTKFY-KKTEIDMIQALDA----------------------PLFNNQK--LGRRVSQMMMTEIIDYMVQS--------ENAKWLE-----
Trichomonas vaginalis 2        TWCDLILSWCKAN-KKEDLKVADALKT----------------------DLFNNKA--IGRALSQQDAEFFLDQLVQR--------QNAQWSD-----
Entamoeba histolytica          LWSQLILKYCECIKKPIMKQSEFNK------------------------LPIFHNEE--LHRTLSENGIELVKEFMVNN--------NKIIDLNKS---
Dictyostelium discoideum       MWQDLILQYCRYY-KIYEIDINESIKSN---------------------SVLFNNEK--INRKLSREALKSIIDDIIEN--------GFAEWVDKEKE-
Chlamydomonas reinhardtii      LWCSLVLQYCQHT-KTFVIDVQGD-------------------------SPLFVNKV--INRKLNQEARVAILDELATQ--------GRAEWMDGGKT-
Cyanidioschyzon merolae        LWRRLILDYCEHF-WILTLRLYDAESP----------------------QMPLFCNRA--IQRRLSRSALQCIFQELVLS--------GDAAWSMPEEK-
Physcomitrella patens          LWKELILKYCKHH-KLFLIDLEEE-------------------------FALFENTS--IQRKLTFEAREQFLSALIAD--------GRAEWLDKNHR-
Welwitschia mirabilis          LWKELIVNYCKHH-KTYIINLEEE-------------------------FPLFENPA--INRKLSHEAKEAFLSALVNE--------GKAEWLDKGHK-
Pinus taeda                    VWKELILNYCKHH-KIFIISLEEE-------------------------FPLFSNPA--INDRKLSYEAKKVFLSALVSE--------GRAEWLDKGQK-
Asparagus officinalis          LWKELILDYCRSQ-KIFIIGLEEE-------------------------FPLFSNPV--IERSLNHEARGVFLSALVSE--------GRAEWMDKSHK-
Hordeium vulgare               LWKELILDYCRSQ-KMYIISLEED-------------------------FPLFSNQK--IERSLSYEAKEVFLAALVSE--------GRAEWIDKSHK-
Oryza sativa                   LWKDLILDYCRSQ-KLYIISLEED-------------------------FPLFSNPK--IERSLSHEAKEVFLAALVYE--------GRAEWMDKGHK-
Sorghum bicolor                LWKDLILDYCRSQ-KIHTISLEED-------------------------FPLFSNPK--IERSLSHEAKEVFLAALVSE--------GRAEWMDKGHK-
Saccharum officinarum          LWKDLILDYCRSQ-KIHTISLEED-------------------------FPLFSNPK--IERSLSHEAKEVFLAALVSE--------GRAEWMDKGHK-
Triticum aestivum              LWKELILDYCRSQ-KMYIISLEED-------------------------FPLFSNPK--IERSLSYEAKEVFLAALVSE--------GRAEWIDKSHK-
Antirrhinum majus              LWKELILDYCRTQ-KIFVIGLDED-------------------------FSLFTNSV--IERSLSHEASEAFLSALVLE--------GRAEWLDKGHR-
Coffea canephora               LWKELIIEYCRAQ-KVFIIGLEED-------------------------FPLFSNHA--IERSLSHEARVAFLSALVSD--------GRAEWTDKGHK-
Lycopersicon esculentum        LWKELIIDFCRTQ-KIFVIALEVD-------------------------FPLFSNAA--IERSLSHEAREAFLSALVSD--------GRAEWLDKGHK-
Arabidopsis thaliana           LWKELILDYCKSQ-KIFLIGVEED-------------------------FPLFSNSA--IERSLSHEARETFLSAIVGE--------GRAEWLDKGHR-
Brassica napus                 LWKELILDYCKSQ-KVFLIGVEKN-------------------------FPLFSNSA--IDRTLSHEAREIFLSAIVGK--------GRAEWLNKGHR-
Citrus clementina              LWKELILDYCRTQ-KVFLILLEEE-------------------------FPLFSNPV--IERSLNNEARETFLSALVSE--------GRAEWLDKGHR-
Fragaria vesca                 LWKDLILDYCRTQ-KIFVIALEED-------------------------FPLFSNPA--IERSLSHEAREAFLSILVSD--------GRAEWLDKRHR-
Gossypium hirsutum             LWKELILDYCRTQ-KIFVIRLEEE-------------------------FPLFSNSV--IERTLSHEAREAFLSALVAE--------GRAEWLDKGHR-
Glycine max                    LWKDLILDFCKTQ-KIFIIGLEEE-------------------------FPLFTNHG--IERSLTHEAREAFLSALVSE--------GRAEWMDKGHR-
Malus x domestica              LWKDLILDYCKTH-HIFVIGLEDQE------------------------FPLFSNPA--IERSLTYEAREAFLSALVAQ--------GRAEWLDKSRR-
Medicago truncatula            LWKELILDYCKTQ-KLFVIALEEE-------------------------FPLFTNTV--IERSLTNEAREAFLSALVSE--------GRAEWMDKGHR-
Populus trichocarpa            LWKELILDYCRTQ-KIFVIGLEEE-------------------------FPLFSNHL--IERSLSNEAREAFLSALVSE--------GRAEWLDRGHR-
Populus tremula                LWKELILDYCRTQ-KIFVIGLEEE-------------------------FPLFSNHL--IERSLSNEAREAFLLALVSE--------GRAEWLDRGHR-
Vitis vinifera                 LWKELILDYCRTQ-KIFVIGLEEE-------------------------FPLFSNPV--IERTLSHEAKEAFLSALVSE--------GRAEWMDKGHK-
Candida albicans               SWVSIILHYCEYY-RITSISIEGIPKHSQLEV-----------------PLSSLSSIFINKT--INRQVNSDFQKLIVKHLIHN--------KAEFINPKKP-
Candida glabrata               TWIDIILTEWCKSH-RVFELGKDGVPVRESDASDADDGADGGTT-TGNEAGRSLFKNEE--INRAVPPLFIDEIWSVMATR--------GVALVTEGR---
Clavispora lusitaniae          AWGALILDYCEHF-RIFSILDSGEVLVQQQ---------------PQELPPLFENKL--IERAVLDEFRKEIFAHLVSK-------LGRAAYVDPKKR-
Debaryomyces hansenii          SWGEIILSYCQHY-KITSLTLGGSVLHTQLND-------------VTTNEIPPLFENKN--INRAVNDEFKAMIFKHLIHK-------LHRAEYINPKQP-
Eremothecium gossypii          AWLGVLKDYARGR-RVWIMGHDGSARE------------------PKDEGSVFFNVQ--LQRRVPGPFVDEIWAHALAK--------GAALRHS-----
Kluveromyces lactis            TWESIILQTCAQL-SKWCINKNGKIYDQ-----------------STGELLISIFENPE--IQRACSLEFQQEIWAYMLQN--------ETALQLKESED-
Kluveromyces waltii            TWCDLILEFAKTT-AAWCMSQEGSIIK------------------DSENSDMSIFRNES--IQRAVPAPFIEQIWSKMVQT--------EKALKLN-----
Saccharomyces cerevisiae       TWIDIISQYCKTK-KIWYMSVDGTVINDNELDSGSTDND-----DSKKISKNLFNNED--IQRSVSQVFIDEIWSQMTKE--------GKCLPID-----
Saccharomyces bayanus          TWIDIISQYCKSK-KIWYMSADGTVIDDDRSENGKIENE-----YSKEVGKNLFNNEE--IQRSVPQVFIDEIWSQMVKE--------GKCLPID-----
Saccharomyces castellii        AWIDLILQYAREN-KCWIMAKTGTSTADPKF--------------------NIFHNES--IQRSVSPMFIDEIWALMIKSQ-------KAIVNND-----
Saccharomyces kluyveri         TWCDLLVQYAKQR-RAWCMGSDGTIMVQEGSLD-----------DSRDNEESIFLNSQ--IQRTVPSPFVEEIWARMLQS--------GKVIKRQ-----
Saccharomyces kudriavzevii     TWIDIISQYCKGK-KIWYMSADGTVMNDNTSGSENTDDD-----DSKKARKNLFNNED--IQRSVSQVFIDEIWSRMVKE--------GKCLPID-----
Saccharomyces mikatae          TWIDIISQYCKSK-RTWYMSADGTVINDSTLDGRNTDDN-----DVKKTSNNLFNNED--IQRSVPQEFIGEIWLQMAKE--------GKCLPID-----
Saccharomyces paradoxus        TWIDIISQYCKCK-KIWYMSADGAVVNDNSLDSGNTDND-----DSKKVSKNLFNNED--IQRSVSQVFIDEIWSQMAKE--------GKCLPID-----
Yarrowia lipolytica            HWKDVILTHSRET-KQMRLSN------------------------------TDSIFENKK--IQRRLKPDVIQLVLSDLVSN--------KKADWVDSKT--
Schizosaccharomyces pombe      AWQMWILWCREN-KQTSITINPEL--------------------LESSLLHNST--IHRTLPLSVFREIIVEDMVKQ--------NLAEWTEKRNP-
Aspergillus fumigatus          KWSSLIQSWCRHH-RIYRLSLIEA---------------------IESPLFHNAT--IRKRLSLSEARAVLDWMAKP-EEEGGGGRAEWIDGGS--
Aspergillus oryzae             KWSLLIQSWCRHH-RTYRLSLIEA---------------------IESPLFHNST--IRKRIPLSEARNILDWMAES-EEKGGGGRAEWIDGGS--
Botryotinia fuckeliana         KWSDLILSYFAFH-RLFRLTVSTL---------------------LSSELFKNER--INRRLDEEGLREVLEFMRKE--------GRVEWIGGGGN-
Coccidioides immitis           KWSALIQAYCRHH-RLYRLSLVDA---------------------LDSPLFHNKQ--IRKRLSLVDARRIVDWMCGA-----QGGRRAEWVGGEAG-
Gibberella zeae                KWADLILAYARHN-RLYRISLSEA---------------------ADSDLFVNRK--LDRRLQFDDIRDVVSFMHTD--------GRVEYVGGGTS-
Magnoporthe grisea             KWSALVLAYCRHH-RIFKLPLTTAAAASSSTSSTTDPLQSQSQSQSSSATDELFHNKT--INRRLSPADVREVIDFMCKE--------GRAEYCSPGTG-
Neosartorya fischeri           KWSSLIQSWCRHY-RIYRLSLIEA---------------------IESPLFHNAT--IRKRLSLSEARAVLDWMAKP-EEEGGGGRAEWIDGGS--
Neurpospora crassa             KWSSLLLAYCRHH-RLFRLSLN-----------------------PDALPFHNPR--INRRLAPGDIRRVVDFLRRD--------GRAEYVLPLGQ-
Phaesosphaeria nodorum         SWSTLIQSYCRHH-RIFTLSLIDA---------------------LETPLFNNTA--LRRRLSLRDAKTILTWMSTP-----EGGNRVEFINASKS-
Sclerotinia sclerotiorum       KWSDLILSYFAFY-RLFRLTISTL---------------------LSSELFKNER--INRRLDEEALREVLEFMRKE--------GRVEWIGGSGS-
Trichoderma reesei             KWSALVLSYARHH-RLFRLVVSSA---------------------AESELFHNRA--IDRRLGPADIREVLDFMRKD--------GRAEFIRASSA-
Uncinocarpus reesii            KWSALIQSYCRHH-RLYRLSLVDA---------------------LDSALFHNRT--IRRRLSLADARKVLDWMCSA-----EGGRRAEWVGGEAG-
Coprinus cinereus              QWIKLLLSYARYR-KLFILRVDDAE--------------------EKGSEWDEVLRNER--INRRVKPAYLETIISTMVKK--------NQAAYEPPKQ--
Phanerochaete chrysosporium    HWTKLILSYARHR-RLFLLRVEDTE--------------------VAGNDWDEIFRNEQ--IRRRLLPSHLAHIMEDMVTK--------NKAVYEPARQ--
Ustilago maydis                QWSTLILDYCRHH-RLFTISPLAGTSDPASGSVAAD------------PHTSLFANQS--IQRSLSAESIRVVLTHLVDH--------KQAAWEQLTG-
Rhizopus oryzae                EWESIILSYARHK-HLFRLELHNAT--------------------TANSGYDIFENKK--INRRLSFEALQDIIEEMVKK--------AEWEGGPKGS--
Blastocladiella emersonii      AWTDLIRAYCAHH-KLYRLDVDSAASL------------------------DLFKNAA--IGRTVPRDMLALLFREAVKA--------GLAEWSTPKD--
Strongylocentrotus purpuratus  AWCDLFLAFHKHH-RIYTVDLKEAASS------------------------ELFNNTK--INRKLSGEGILLVLEELRQK--------GNIEWTDKA---
Caenorhabditis briggsae        AWARLVIDYAQHN-KIYSLDIAEATTS------------------------ELFNNQK--LNRRLSTDGVNTVLQYLEQK--------KLIEFTDNG---
Caenorhabditis elegans         AWARLVIDYAQHN-KIYSLDIAEATTS------------------------ELFNNQK--LNRRLSTDGVNTVLQYLEQK--------KLIEFTDNG---
Heterodera glycines            AWGRLLLDYCQAN-RIYTTDLDEISKS------------------------DLFNNRR--LNRHLDLSGIRAVFDFLELQ--------KHVEWKDKD---
Paragonimus westermani         AWCQLVLDYFRHK-NVYNISIASIRDSS-----------------------CSLFHNKS--ISRSANSDLISNVLDELHRR--------GNLEWIDKQ---
Schistosoma japonicum          AWCQLVLDYFQSK-KQFSLSVASIRDPS-----------------------CPLFNNKS--IQRSATPDLIDLVLTELHRR--------GNLEWVDKS---
Schistosoma mansoni            AWCQLVLNYFQSK-KQFTLSVASIRDPS-----------------------CPLFNNKS--IQRSASPELVDLILTELHKR--------GNLEWLDRS---
Schistosoma mediterranea       AWCQLILSYHQNF-KQYTLNVKDAYQS------------------------PLFNNKS--IDRKVRSDLLTCILDELQKR--------GNLEWTDKS---
Amblyomma variegatum           AWSNLILNYYRAR-KEYVLDVAEALAS------------------------PLFHNKD--ISRKLSADALREILKSMSSR--------SQVAWTDKQ---
Boophilus microplus            AWSNLILNYHRAH-KAYVLDVAEALAS------------------------PLIHNKD--ISRKLSADSLKEILKYMSSR--------GQVAWTDKQ---
Aedes aegypti                  TWKSLILDYQKHS-KQAVINLNEDS--------------------------VPFVNEA--ISRKLSPEGRLWVMEALEKT--------ANAAPMDKR---
Anopheles gambiae              TWKELVLNYQKHE-GQALLNIAEDA--------------------------PPFVNRE--LARKLSPEARLWVMEELART--------GHAATADKR---
Apis mellifera                 AWKSLILEYYRIT-KQAIIDVREIHSS------------------------PLFNNTA--INRKLPSEARLVLLEELAKS--------GNASPLDKT---
Acyrthosiphon pisum            AWRTLLLDYCRTQ-KVSVIDVREGDLL------------------------PVFNNTT--ISRKLSPDAIMTVLGVLQKT--------GNAEPLDKT---
Bombyx mori                    AWEQLITDYLKAT-KQSTIDIREASNT------------------------PLFNNIE--INRKLSQEAILTILEDMAKA--------GKAAPIDKS---
Drosphila melanogaster         VWTDLFLKYLRHT-NRFTLSIGDQNS-------------------------PLFHNEA--LKRRLSPELVLAILGELERS--------GHANPLDKR---
Drosphila pseudoobscura        VWTDLFLKYLKHT-NKFSLSINEQSL-------------------------PLFHNES--IQRRLSPELILVILEQLQRS--------GHATALDKR---
Lutzomyia longipalpis          VWKDLILSYQKHR-KEAIITVNANS--------------------------PLFCNDG--IKRQLSPDARLQVMEELQKS--------GNAAPLGN----
Ciona intesinalis              AWCSLILDYYNHH-KLFRLRVTDVLAS------------------------PLFNYKA--IDRRLTKPDVMEILSILHKK--------GNIEWEDKE---
Molgula tectiformis            MWCDLVIAYQKYK-NQATISIQEANSS------------------------PLFNNKQ--LNRKLQQRDIEKVFNALCKK--------GNLEWTDKN---
Leucoraga erinacea             AWCSLVLSYLRYH-KLYTIDVLEAQES------------------------PLFNNKK--IQRKFPVEAIQVVLEELRKK--------GNLEWIDKN---
Danio rerio                    AWCSLVLSYCRHR-KLYTLDVLEAQES------------------------PVFNNKK--IERKLSVEAIQVVFEELRKK--------GNLEWLDKN---
Fugu rubripes                  AWCSLALSYCRHH-KLYTLDVLEAQES------------------------PVFNNKK--IERKLSMEAIQVVFEELRKK--------GNLEWLDKN---
Gasterosteus aculeatus         AWCSLALSYCRHH-KLYTLDMMEAQES------------------------PVFNNKK--MERKLSIEAIQVVFEELRKK--------GNLEWLDKN---
Ictalurus punctatus            AWCSLALSYCRHR-KLYTVDVLEAQES------------------------PMFNNKK--IERKLSVEAIQVVFEELRKK--------GNLEWMDKN---
Oryzias latipes                AWCSLALSYCRHH-KLYTLDMMEAQES------------------------PMFNNKK--IERKLSMEAIQIVFEELRKK--------GNLEWLDKN---
Oncorhynchus mykiss            AWCSLALSYCRHH-KLYTLDIMEAQES------------------------PVFNHKN--IDRKLSMEAILIVFEELRKK--------GNLEWLDKN---
Platichthys flesus             AWCSLALSYCRHH-KLYTLDIMEAQET------------------------PVFNNKK--IERKLSMEAIQVVFEELRKK--------GNLEWLDKN---
Pimephales promelas            AWCSLALSYCRHR-KLYTLDVLEAQES------------------------PMFNNKK--IERKLSVEAIQVVFEELRKK--------GNLEWLDKN---
Salmo salar                    AWCSLALSYCRHH-KLYTLDIMEAQES------------------------PVFNHKN--IDRKLSMEAILIVFEELRKK--------GNLEWLDRS---
Tetraodon nigroviridis         AWCSLALSYCRHH-KLYTLDVLEAQES------------------------PVFNNKK--IERKLSMEAIQVVFEELRKK--------GNLEWLDKN---
Gallus gallus                  AWCALALAYSRRQ-RLPAMTLREAQDS------------------------PLFANRR--IQRKLPPEAIQVVLEELRKN--------GNLEWLDKN---
Taeniopygia guttata            AWCALALAYSQQH-RLPAWTVREAQDI------------------------PLFANHR--IQRKLPLESIQVVLEELRKN--------GNLEWTDKN---
Xenopus laevis                 AWSSLVLSYCRQN-KLYTMNLMEIQES------------------------PLFNNKK--IQRKLSLESVQVVLEELRKK--------GNLEWIDKN---
Xenopus tropicalis             AWSSLVLSYCRHN-KLYTMNLMEIQES------------------------PLFNNKK--IQRKLSLESVQVVLEELRKK--------GNLEWIDKN---
Bos taurus                     AWCSLVLTFCRLH-KQSSMTVMEAQES------------------------PLFNNVK--IQRKLPVESIQVVLEELRKK--------GNLEWLDKN---
Canis familiaris               AWCSLVLSFCRLH-KQSSMTVMEAQES------------------------PLFNNVK--LQRKLPVESIQVVLEELRKK--------GNLEWLDKN---
Equus caballus                 AWCSLVLSFCRLH-KQSSMTVMEAQES------------------------PLFNNVK--IQRKLPVESIQVVLEELRKK--------GNLEWLDKN---
Homo sapiens                   AWCSLVLSFCRLH-KQSSMTVMEAQES------------------------PLFNNVK--LQRKLPVESIQIVLEELRKK--------GNLEWLDKS---
Macaca mulatta                 AWCSLVLSFCRLH-KQSSMTVMEAQES------------------------PLFNNVK--LQRKLPVESIQIVLEELRKK--------GNLEWLDKS---
Monodelphis domestica          AWCSLVLSFCRLH-RQSSMTVMEAQES------------------------PLFNNNK--IQRKLPMESIQIVLEELRKK--------GNLEWLDKS---
Mus musculus                   AWCSLVLSFCRLH-KQSSMTVMEAQES------------------------PLFNNVK--LQRKLPVESIQIVLEELRKK--------GNLEWLDKN---
Oryctolagus cuniculus          AWCSLVLSFCRLH-KQSSMTVMEAQES------------------------PLFNNVK--LQRKLPMESIQIVLEELRKK--------GNLEWLDKN---
Rattus norvegicus              AWCSLVLSFCRLH-KQSSMTVMEAQES------------------------PLFNNVK--LQRKLPVESIQIVLEELRKK--------GNLEWLDKN---
Sus scrofa                     AWCSLVLSFCRLH-KQSSMTVMEAQES------------------------PLFNNVK--IQRKLPVESIQVVLEELRKK--------GNLEWLDKN---
consensus                      W  lil ycr h kly i i e                             plf n    i rrls e l  il  l          gh ewvdk
```

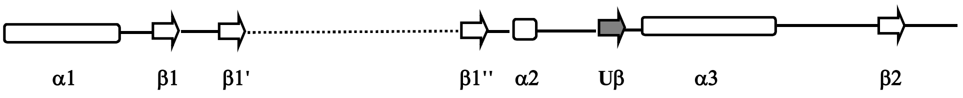

```
Theileria annulata              WGALFLKKNHKGTTLFQIHDEYTKSLNPKDNKAETDEIDSLKKKRNLLVKSTFRFGVFPYPLSEMTNSVLECIKSQCTN---------RDIETIYHIFYS
Theileria parva                 WGALFLKKSHKGSTLYQIHQDYTKALNSKDNKVEGDEIESLKKKRNLLLKSKFNFGVFPYPLTEMANSVLECIKSQCTT---------RDIETVYHIFYS
Paramecium primaurelia          -----------------------------------------------------NFSVNLVSPFELADAIYAWAKEKKLI---------GYTETLRGITEG
Leishmania brazilia             -----------------------------------------------PKDFSVLVCTTEGGLKGIEENLLRYILEHGEVQTVAMLSKKGTVMTFDELAGG
Leishmania infantum             -----------------------------------------------PRDFSVLVCTTEDGFKGMEETLLRYILDHGEVQTTAMLSKKGTVMTFDELASG
Leishmania major                -----------------------------------------------PRDFSVLVCTTEDGFKGMEETLLRYILDHGEVQTTAMLSKKGTVMTFDELASG
Trypanosoma brucei              ----------------------------------------------GMGDVSVLVTTSKGGLKELEQSLLAWILERGAGTTVAYLSQKGVVMTFDELVDG
Trypanosoma b.gambiense         ----------------------------------------------GMGDASVLVTTSKGGLKELEQSLLAWILERGAGTTVAYLSQKGVVMTFDELVDG
Trypanosoma congolese           ----------------------------------------------NEEDVSLLVSTNKGGLKELEQSLLEWILERGVGTTVAHLSQKGVVMTFNELVDG
Trypanosoma cruzi               ------------------------------------------KGENDDADFAVLLACNESGLKGLEQALLSWILDRGAGTTTAHLAQKGVVMTFDELAES
Trypanosoma vivax               ------------------------------------------------GDYSVLVACNEGGLAAIEQSLLAWILERGAGTTAASLAQNGAVMTFDELVEG
Giardia lamblia                 ------------------------------------------------------YYLLSAPEDTIPDSLLRLLKNNNFT--------VGCIITVLELKKT
Trichomonas vaginalis 1         -------------------------------------------------STKTRARIIWRTTQQIGDMVRQYLDNIGSL---------NTMMTYEELING
Trichomonas vaginalis 2         -------------------------------------------------DKHTKCKIIFRKPAQWANIFYQWAVKNNLQ---------GVVFTFYELREG
Entamoeba histolytica           ----------------------------------------------------SKLILLYKPLREWGKELYEYGNSKGLI---------GQSDTFFSIEND
Dictyostelium discoideum        ----------------------------------------KEKEKDKDNNNNNRVLIMWRKPDEWASLIYKWVADCGLL---------NTVLTVWEIQNG
Chlamydomonas reinhardtii       -----------------------------------------------------RCLVYWRRVDEWAGAVTEFVRTFGLS---------DSVMTVDELSSG
Cyanidioschyzon merolae         ----------------------------------------------------EELLIFWRSPRLWADELLRVVAQYGQN--------KGGVFTLPELASM
Physcomitrella patens           -----------------------------------------------------KCLILWRRIEDWADSLLNFVRENGIE-----------LMTLEEIISG
Welwitschia mirabilis           -----------------------------------------------------RCLILWRRIEDWAAYILHFVHENGLE---------NGVMTLEELQTG
Pinus taeda                     -----------------------------------------------------KCLILWRRIQDWAEYILKFVHENGLE---------DGVMTLEEIRAG
Asparagus officinalis           -----------------------------------------------------KCLILWLRIQDWADHIIKFVKDNGLE---------DSVVTVEDIRSG
Hordeium vulgare                -----------------------------------------------------KCLILWLRIQDWANYILDFVKENGLE-----------VTTIEDLRSG
Oryza sativa                    -----------------------------------------------------KCLILWLRIQDWANYILNFVKDNGLE---------DSVMTVEEIRSG
Sorghum bicolor                 -----------------------------------------------------KCLILWLRIQDWANFILNFVKDNGLE-----------VMTIEEIRSG
Saccharum officinarum           -----------------------------------------------------KCLILWLRIQDWANFILNFVKDNGLE-----------VMTIEEIRSG
Triticum aestivum               -----------------------------------------------------KCLILWLRIQDWANYILHFVKENGLE-----------VTTIEDIRSG
Antirrhinum majus               -----------------------------------------------------KCLLLWHRIQDWADLILSFAKDNGLE---------DSVMTVEEIRSG
Coffea canephora                -----------------------------------------------------KCLILWHRIQEWADLIVHFVKENGLE---------DNVTTVEEIRSG
Lycopersicon esculentum         -----------------------------------------------------KCLVLWHRIQDWADLIVNFVKENGLE---------DSVMTVEEIRSG
Arabidopsis thaliana            -----------------------------------------------------KCLILWHRIQDWADIVLQFVRDNGLE---------DSVMTVEEIRSG
Brassica napus                  -----------------------------------------------------KCLILWHRIQNWADIILKFVRENGLK---------DSVMTVEKIRSG
Citrus clementina               -----------------------------------------------------KCLILWHRIQDWADIILGFVKDNGLE---------DSVMTVEEIRLG
Fragaria vesca                  -----------------------------------------------------KCLILWHRIQEWANIILNFVKEYGLE---------DSVMTVEEIRSG
Gossypium hirsutum              -----------------------------------------------------NCLILWHRIQEWADIIVNFVKDNGFE---------DSVMTVEEIRSG
Glycine max                     -----------------------------------------------------KCLILWHRIQDWADILIQFAKDNGLE---------DGVVTIEEIRSG
Malus x domestica               -----------------------------------------------------KCLVLWHRIQDWANIVLDFVKDNGLE---------DSVMTVEEIRTG
Medicago truncatula             -----------------------------------------------------KCLILWHRIQDWADILLQFAKDNGLE---------DGVVTIEEIRFG
Populus trichocarpa             -----------------------------------------------------KCLILWHRIQDWADILLHFVRDNGFE---------DSVMTVEEIRTG
Populus tremula                 -----------------------------------------------------KCLILWHRIQDWADILLHFVRDNGFE---------DSVMTVEEIRTG
Vitis vinifera                  -----------------------------------------------------KCLILWHRIRDWADLILRFVRENGLE---------DSVMTVEEIRSG
Candida albicans                ---------------------------------------------------ELGVYIYWRSLVDWGDLLYQYVDDTGQK---------GTVLTIYELTKS
Candida glabrata                ----------------------------------------------------ASYYVLWRTLDSWASLILQWFETVGKL---------NQVVTLYELTES
Clavispora lusitaniae           ---------------------------------------------------DAGILLFWRTPAEWAALIREHVESTGQL---------GTVLTVYELTKL
Debaryomyces hansenii           ---------------------------------------------------ETGILIYWKTLIEWANILHDFVERTGQL---------GSVLTIYELTKL
Eremothecium gossypii           ---------------------------------------------------ADSFYVLWRSLDSWSALLLQWFETCGRL---------NQVVTIYELVAG
Kluveromyces lactis             ---------------------------------------------------SNMIAIFWESLDSWSSTILEWCETSGKL---------NQVITIYEICES
Kluveromyces waltii             ----------------------------------------------------GVYLILWKSVDHWSSQILQWFETSGKL---------NQVVTLYELLEG
Saccharomyces cerevisiae        -----------------------------------------QSGRRSSNTTTTRYFILWKSLDSWASLILQWFEDSGKL---------NQVITLYELSEG
Saccharomyces bayanus           -----------------------------------------QSGRKSNNTTTTRYFILWKNLDNWASLILQWFEDSGKL---------NQVITLYELSES
Saccharomyces castellii         --------------------------------------------------NNNNYFILWQNIDSWASLILQWFEDSNKL---------NQVVTIYELSQG
Saccharomyces kluyveri          ----------------------------------------------------NGYYVLWKDLDSWSSNILQWFETTGKL---------NQVVTIYELLEG
Saccharomyces kudriavzevii      -----------------------------------------QTGKKSGNATSTRYFIMWKSLDNWASLILQWFEDSGKL---------NQVVTLYELSEA
Saccharomyces mikatae           -----------------------------------------QSGRKSSNTTTTRYFVLWKSVDNWASLILQWFEDCGKL---------NQVVTLYELSQG
Saccharomyces paradoxus         -----------------------------------------QSGRKSNNTTTTRYFILWKSLDNWASLILQWFEDSGKL---------NQVITLYELSEG
Yarrowia lipolytica             ---------------------------------------------------KTEVWIWWRSAEEWATLILAWIDSTGQN---------GSIVTFYDIAGD
Schizosaccharomyces pombe       ---------------------------------------------------KDVFWVYWRSISEWGNMILKWLSDMGRE---------GSICTFYELQEQ
Aspergillus fumigatus           ---------------------------------------------------KSVAWIWWRRPEEWAGIVADWVEATGQK---------NVVLTVYELLEG
Aspergillus oryzae              ---------------------------------------------------KTIAWVWWRRPEEWAGILADWVENTGQK---------NVVLTVYELVEG
Botryotinia fuckeliana          -----------------------------------------------GKVGGDICWVWWRKVDEWARVIEDWVDETGQR---------GSVLTLYELVEG
Coccidioides immitis            --------------------------------------------------GKSVAWIWWRRPEEWAGVIADWVEETAQK---------NTVLTLYELTEG
Gibberella zeae                 ---------------------------------------------------GDVVFLYWRKPEEWAELVENYVEESGQK---------GSVLTVYELVEG
Magnoporthe grisea              ------------------------------------------------QAGGDVAWIYWRSPEEWAQLIEGWVDETAQK---------GSVLTLYELVEG
Neosartorya fischeri            ---------------------------------------------------KSVAWIWWRRPEEWAGIVADWVEATGQK---------NVVLTVYELLDG
Neurpospora crassa              -------------------------------TKKDGAMTTTARGGGGEAGEGGEAFIYWRTPEEWGSLIEGWVEETGQR---------GSVLTVYELREG
Phaesosphaeria nodorum          -------------------------------------------KKAAVEEEGGRCWIFWRRPEEWSAVLEEWVDRTGQK---------GTVLTLYEIVEG
Sclerotinia sclerotiorum        ---------------------------------------------GSGKVGGDVCWVWWRKVDEWARIIEEWVDETGQR---------GSVLTLYELVEG
Trichoderma reesei              ------------------------------AAAGSDVGGFSSGAGAAGGGGAGVVLLYWRKPEEWAALVEAYVDETAQK---------GSVLTVYELTEG
Uncinocarpus reesii             --------------------------------------------------GKSAAWIWWRRPEEWAGVIADWVEETAQK---------NTVLTLYELIEG
Coprinus cinereus               ---------------------------------------------------TKAVLLYWRTPEEWAEVLHEWATSTGQL---------NTIMTFYEITDP
Phanerochaete chrysosporium     ---------------------------------------------------TRSVLLYWRTPEEWAEVLHNWADSTGQL---------NTILTFYEIIEP
Ustilago maydis                 -----------------------------------------AATKSKRSNANSKAFIYWKTPVQWADSIYDWVMQTGQN---------KSIMTLFELNQG
Rhizopus oryzae                 ---------------------------------------------------KTEAYLYWHTPEEWANLIWNWINETGQN---------DQIVTYYEIAHG
Blastocladiella emersonii       ---------------------------------------------------TTRVLVLWKSVAEWAAAMVEWARNAGFT---------DTVMTGYELRFG
Strongylocentrotus purpuratus   ---------------------------------------------------KTRCLVMWRTPEEWGNLIYKWAGNSGMT---------NTVCTLYEIAQG
Caenorhabditis briggsae         ---------------------------------------------------RTRFHIFWRRPDVWANMIYQWAVENAFL---------NTPLTLYEITHG
Caenorhabditis elegans          ---------------------------------------------------RTRFHIFWRRPDVWANMIYQWAVENAFI---------NTPLTLYEITHG
Heterodera glycines             ---------------------------------------------------KTRCNIYWRRPEEWGQLLYEWANSIGLL---------NTVVTLYELTQG
Paragonimus westermani          ---------------------------------------------------RINARIIWRTPEEWADILFRWARDTGHG---------NSVCTLYELTDG
Schistosoma japonicum           ---------------------------------------------------HTNARIIWRTPEEWADIIAKWARSTGHG---------SSVCTFYELTDG
Schistosoma mansoni             ---------------------------------------------------HTNARIIWRTPEEWADLIARWARSTGHS---------NSVCTFYELTDG
Schistosoma mediterranea        ---------------------------------------------------NVTCKIIWRTTVEWSDLIYKWIKKSGNV---------NTICTLYELTDG
Amblyomma variegatum            ---------------------------------------------------QTRCYVFWRSPEEWGKLLYDWADATGHL---------NSVCTFYELVQG
Boophilus microplus             ---------------------------------------------------QSRCYVYWRSPEEWGKLIHDWADATGHL---------NTVCTFYELVQG
Aedes aegypti                   ---------------------------------------------------KQQWEVYWHTLDEWSSLLHGWAVANGMT---------NTVCTLYELVAG
Anopheles gambiae               ---------------------------------------------------KQQWEVYWHTLDEWSNILYDWAVASGTT---------NTVCTLYELVAG
Apis mellifera                  ---------------------------------------------------KQRWLIYWHTLEEWGEIIYNWAQENGFC---------GSVCTLFELTQG
Acyrthosiphon pisum             ---------------------------------------------------RTRWNVYWHTLDEWASIVYKWAQDNAML---------NTVCTFYEIASD
Bombyx mori                     ---------------------------------------------------KNVWEVYWHSLDEWGNMIYNWACNNGFN---------NSVCTLFELREG
Drosphila melanogaster          ---------------------------------------------------RQEWQVYWFTLEEYGNMVYDWVQETGQT---------NTICTLYEIASG
Drosphila pseudoobscura         ---------------------------------------------------RQEWQVYWYTLEAYGNMVYDWIQETGQT---------NSICTLYEIASG
Lutzomyia longipalpis           ---------------------------------------------------KVQWEIYWYTLDEWADKIYTWVTNCGLN---------NTVCTLYELTSG
Ciona intesinalis               ---------------------------------------------------KTVCKVFWKTPAQWSDIIFKWVNQSGLN---------NTVCTLHEITNG
Molgula tectiformis             ---------------------------------------------------KTSCYILWKTVDQWAELIYSWVGRNGMA---------NTVCTLYEITKN
Leucoraga erinacea              ---------------------------------------------------KTRCLIMWRRPEEWGKLVYQWVSKNGLT---------NSVLTFYELSNG
Danio rerio                     ---------------------------------------------------KSRCLIMWRRPEEWGKLIYQWVSKNGMV---------NSVFTLYELANG
Fugu rubripes                   ---------------------------------------------------KSRCLVMWRRPEEWGKLMYQWVSRNGMV---------NAVFTLYELSNG
Gasterosteus aculeatus          ---------------------------------------------------KSRCLVMWRRPEEWGKLIHQWVSKNGMV---------NSVFTLYELSNG
Ictalurus punctatus             ---------------------------------------------------KTRCLIMWRRPEEWGKLIYQWVSKNGMV---------NTVFTLYELSNG
Oryzias latipes                 ---------------------------------------------------KLRCLVMWRRPEEWGKLIYQWVSKNGMV---------NSVFTLYELSNG
Oncorhynchus mykiss             ---------------------------------------------------KTRCLVMWRRPEEWGKLIYQWVSKNGMV---------NTVFTLYELANG
Platichthys flesus              ---------------------------------------------------KSRCLVMWRRPEEWGKLIYQWVSRNGMN---------NSVFTLYELVNG
Pimephales promelas             ---------------------------------------------------KSRCLIMWRRPEEWGKLIYQWVSKNGMV---------NSVFTLYELANG
Salmo salar                     ---------------------------------------------------KTRCLVMWRRPEEWGKLIYQWVSKNGMV---------NTVFTLYELANG
Tetraodon nigroviridis          ---------------------------------------------------KSRCLVMWRRPEEWGKLIYQWVSRNGMV---------NSVFTLYELSNG
Gallus gallus                   ---------------------------------------------------KTSFLIMWRRPEEWGKLIYQWVSKNGLT---------NSVFTLYELISG
Taeniopygia guttata             ---------------------------------------------------KTSFLIMWKRPEEWGKLIYQWVSKNGLT---------NSVFTLYELVSG
Xenopus laevis                  ---------------------------------------------------KSRFLIMWRRPDEWGKVIYQWVSKNGMT---------NSVFTLYELISG
Xenopus tropicalis              ---------------------------------------------------KSRFLIMWRRPDEWGKVIYQWVSKNGMT---------NSVFTLYELISG
Bos taurus                      ---------------------------------------------------KSSFLIMWRRPEEWGKLIYQWVSKSGQN---------NSVFTLYELTNG
Canis familiaris                ---------------------------------------------------KSSFLIMWRRPEEWGKLIYQWVSRSGQN---------NSVFTLYELTNG
Equus caballus                  ---------------------------------------------------KSSFLIMWRRPEEWGKLIYQWVSRSGQN---------NSVFTLYELTNG
Homo sapiens                    ---------------------------------------------------KSSFLIMWRRPEEWGKLIYQWVSRSGQN---------NSVFTLYELTNG
Macaca mulatta                  ---------------------------------------------------KSSFLIMWRRPEEWGKLIYQWVSRSGQN---------NSVFTLYELTNG
Monodelphis domestica           ---------------------------------------------------KSSFLIMWRRPEEWGKLIYQWVSKSGQN---------NSVFTFYELTNG
Mus musculus                    ---------------------------------------------------KSSFLIMWRRPEEWGKLIYQWVSRSGQN---------NSVFTLYELTSG
Oryctolagus cuniculus           ---------------------------------------------------KSSFLIMWRRPEEWGKLIYQWVSRSGQN---------NSVFTLYELTNG
Rattus norvegicus               ---------------------------------------------------KSSFLIMWRRPEEWGKLIYQWVSRSGQN---------NSVFTLYELTSG
Sus scrofa                      ---------------------------------------------------KSSFLIMWRRPEEWGKLIYQWVSRSGQN---------NSVFTLYELTNG
consensus                                                                              lilwrk dewa mi  wv   g          ntvmTiyel  g
```

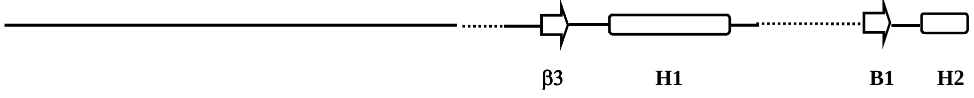

```
                                                                                               *
Theileria annulata               KKE------------------CNKDFNKFPEENLAFILSKLSVNNQITLSFNDSV---PLD-SLNNKNVGVQLL------
Theileria parva                  KRE------------------CNKDFNKFPEENLAFILSYLCVNNKLTLSFNDSV---PLD-SLNNKNVGLQLL------
Paramecium primaurelia           SQTD-----------------QSKKFYNLPQEQILKACLI-LEETGRCQVYEFDG------------LYSIKFI------
Leishmania brazilia              AALGYGQSRPAYLARLSSAAVPVADVGDLSEEQAVRTYLHALDHHPVSVMRPFKVTLFNLDGTTTQPYQGVKFGGE----
Leishmania infantum              AALGYGQTRPAYLARFSSAAVPVADVGRLSEEQAIRTYLHAVNHRPVSAMRPFKVTLFNLDGTATQPYQGVKFGGE----
Leishmania major                 AALGYGQTRSAYLARFSSAAVPVADVGRLSEEQAIRMYLHALNRRPVSAMRPFKVTLFNLDGTATQPYQGVKFGGE----
Trypanosoma brucei               QCLLYSRESEQLLPRLTQEAVPIEDVGALSQEQAVRCFLHTLAERPVSHKGLFSVTLFNLDGSDRRPYEGVKFGGAAI--
Trypanosoma b.gambiense          QCLLYSRESEQLLPRLTQEAVPIEDVGALSQEQAVRCFLHTLAERPVSHKGLFSVTLFNLDGSDRRPYEGVKFGGAAI--
Trypanosoma congolese            HCLAYKCESSGLVRRLSQDVVPIEDVGVLSHEQAVRCFLHTLSVRPISHQGLFSVTLFNLDGSNRRPYEGVKFGGAAAR-
Trypanosoma cruzi                NCLAYRRDREPFLKRLSAAPVPVGDVGALSNEQAIRTLLHSLKSRPLSALSPIHITLFNMDGSDRRPYEGVKFGGAVAC-
Trypanosoma vivax                QCLVYKRENEPMICRLAQDDVPVGDVGALTEEQAVRTLLKALATRPASIRWPYRITFFNLDGSSVEPYQGVKFGGVVC--
Giardia lamblia                  MDK------------------DASDLFALPDPVLKKALEA-LQKSGNLALYQE--------GPVFGDETGIKILGL----
Trichomonas vaginalis 1          DET------------------EGEGFHGLSADEFHDAMTF-MESKGRCKIIPGSS----------LVEYGVKFF------
Trichomonas vaginalis 2          DDT------------------QSEAFHNMDQEQMLKAIEC-LVKEKKANLIKAAD----------FDENGVKFL------
Entamoeba histolytica            KES---------------------VFYQMDDELLIEGLNS-IKEQGKMKLVQH------------EGEYGIFWL-----K
Dictyostelium discoideium        DDS------------------KKQEFHQLNTTILMKSLKV-LEKQSKCQTFSQ------------DENVGVKFFSI----
Chlamydomonas reinhardtii        DDV------------------RGTDLYGVHPEILTRALKL-LEAQGKVRTFKGA----------TPEELGVKFI------
Cyanidioschyzon merolae          LGERCSLGRQQR---------AQALQTAIPIAFLEHILEL-LAAQGKARVFS------------TNEGRGVKFDFS----
Physcomitrella patens            DET------------------YGTELAGLDRGVLVRAVKI-LEQRGKAAMFKGS----------STDDDGVKFSPN----
Welwitschia mirabilis            VES------------------RGTDLAGIDRTVLVKALRL-LEQRGKAVIFKGS----------ATDDEGVKFSA-----
Pinus taeda                      VES------------------RGTELAGIDRIVLIRALKL-LEQRGKAVIFKGT----------STDDEGVKFSA-----
Asparagus officinalis            FES------------------RGTELAGIDRGVLMRALRL-LEQKGKAAIFKGS----------SSDDEGVKFSV-----
Hordeium vulgare                 IET------------------HGTELAGIDRGVLMRALRL-LEQKGKAVIFKGT----------SADDEGVKFSA-----
Oryza sativa                     IET------------------RGTELAGIDRGVLMRALKL-LEQKGKAAIFKGT----------SADDEGVKFSA-----
Sorghum bicolor                  IDT------------------RGTELEGIDRGVLMRALRQ-LEQKGKAAIFKGT----------SADDEGVKFSV-----
Saccharum officinarum            IDT------------------RGTELEGIDRGVLMRALRQ-LEQKGKATIFKGT----------SADDEGVKFSV-----
Triticum aestivum                IET------------------HGTELAGIDRGVLMRALRL-LEQKGKAVIFKGS----------SADDEGVKFSV-----
Antirrhinum majus                IES------------------RGTELHGMDRTILMRALKH-LEHKGKLAIFKGT----------SADDEGIKFSI-----
Coffea canephora                 TES------------------RGTELYGIDRSVLMRALKL-LEHKGKLVIFKGA----------STDDEGVKFSL-----
Lycopersicon esculentum          VES------------------RGTELHGIDRTVLMRALKV-LEHKGKLAIFKGT----------SADDEGVKFSV-----
Arabidopsis thaliana             TES------------------LGTELQGIDRTILMRALKL-LENKGKLALFKGT----------SADDEGVKFSV-----
Brassica napus                   TES------------------LGTELEGIDRTILMRALKL-LENKGKLALFKGT----------SADDEGVKFSV-----
Citrus clementina                IES------------------RGTELHGMDRTILMRALKL-LEHKGKVAIFKGT----------SADDEGVKFSV-----
Fragaria vesca                   IES------------------RGTELHGIDRTILMRALKL-LEQKGKLAIFKGT----------TADDEGVKFSI-----
Gossypium hirsutum               IES------------------RGTELQGIDRTILVRALKL-LEHKGKLAIFKGT----------STDDEGVKFSV-----
Glycine max                      TES------------------QGTDLHGIDRTILNRALKL-LEQKGKLVVFKGT----------STDDEGVKFSI-----
Malus x domestica                IES------------------RGTELHGIDRTVLMRALKM-LEQKGKLAIFKGT----------TGDDEGLKFSI-----
Medicago truncatula              TES------------------QGTELHGIDRTILNRALKL-LEQKGKLVVFKGT----------STDDEGIKFSV-----
Populus trichocarpa              VES------------------RGTELHGIDRTILMRALKL-LEHKGKLAIFKGT----------SADDEGVKFSV-----
Populus tremula                  VES------------------RGTELHGIDRIILMRALKL-LEHKGKLAIFKGT----------SADDEGVKFSV-----
Vitis vinifera                   TES------------------RGTELHGMDRTVLMRALKQ-LEHKGKLAIFKGT----------SADDEGVKFSL-----
Candida albicans                 EETT-----------------VPQDLHNLDETFLVKIIKDYLIKQGKAQLLIDE----------NNEIGGVKIV------
Candida glabrata                 DET------------------ADWEFHSMPLPLLHRCLKP-LCNRNRATLMKDE----------HGTPVALKVV------
Clavispora lusitaniae            EET-------------------DPLLRNIDYNLFVRAIDV-LMKQGKAQVLKAE--------DGSSQIGGVKII------
Debaryomyces hansenii            EDSG-----------------VDEDLKDLDYNLLVRILKGVLIKQGRAQILMNE---------DGTQIGGVKIV------
Eremothecium gossypii            DES------------------AMWEFHGMHDGLCELALGK-LVERGRATLISND-----------GSVVAVKVI------
Kluveromyces lactis              DEN------------------TNCKFYGMPSSFCLLVLHR-LVDRNRATLLKD-----------QGKIVGVKIV------
Kluveromyces waltii              DET------------------LGWEFHGMHSSVCEVCIQR-LCDRGRATLLKE-----------QNKIMGLKVV------
Saccharomyces cerevisiae         DET------------------VNWEFHRMPESLLYYCLKP-LCDRNRATMLKDE----------NDKVIAIKVV------
Saccharomyces bayanus            DET------------------LGWEFHGMPENLLYYSLKP-LCDRNRATMLKDE----------NGKVIAIKVV------
Saccharomyces castellii          DET------------------INWEFHQMPEPLLILALKP-LCKRNRATMLKDE----------DGAPIAIKVI------
Saccharomyces kluyveri           DES------------------IGWEFRGMNPELCEMCLEK-LCSRGRATTLKE-----------NGKIMGVKVV------
Saccharomyces kudriavzevii       DET------------------LDWEFHGMPENLMYYCLKP-LCDRNRATMLKDE----------NGKVIAIKVV------
Saccharomyces mikatae            DET------------------INWEFHGMPENLLYYCLKP-LCDRNRATMLKDE----------NDKVIAIKVA------
Saccharomyces paradoxus          DET------------------FSWEFHGMPENLLYYCLKP-LCDRNRASMLKDE----------NDKVIAIKVV------
Yarrowia lipolytica              DSP------------------GVPEMVGMDSTMLHKVCQV-LVHQGKAAVMRDE----------DGNEVGLKV-------
Schizosaccharomyces pombe        YK----------------------EVDCLDEVLLHKVLEL-LMKKGNIELMKGS----------SGKYSGFKVLKA----
Aspergillus fumigatus            EAT------------------MSQEWHGMDADVMLKSLNI-LVKRGKAQVFGSE------------GQEGVKFF------
Aspergillus oryzae               EAT------------------MSQEWHGMDVDVMMKSLNV-LVKRGKAQVFGSE------------GQEGVKFF------
Botryotinia fuckeliana           EGG------------------RGAEFHGLDTEILQKALAI-LVKRGKAQVFGQE------------DQQGVKFF------
Coccidioides immitis             EAT------------------MSQEFHGMDPDVLQKSLHV-LVKRGKAQVFGNE------------DQQGVKFF------
Gibberella zeae                  DGT------------------KGTDIHGMDTDVLLKALNV-LVKRNKAQIFGQD------------DSLGVKFF------
Magnoporthe grisea               DGT------------------LGTDIHGIDRDMLHRALQL-LVKRGRAQIFGQE------------DSQGVKFF------
Neosartorya fischeri             EAT------------------MSQEWHGMDADVMLKSLNV-LVKRGKAQVFGSE------------GQEGVKFF------
Neurpospora crassa               EGT------------------RGTEIWGMDGDVLVKALGT-VVKRGKAQIFQSGE-----------DSLGVKFF------
Phaesosphaeria nodorum           DAS------------------RKEEFWGMDLELLMRSLGV-SVKRGKAQIFGGE------------GSEGVKFF------
Sclerotinia sclerotiorum         EGG------------------RGAEFHGLDAEILQKALAI-LVKKGKAQVFGQE------------DQQGVKFF------
Trichoderma reesei               ENT------------------RGTEFHGMDNLVLMKALNI-LVKQGKAQIFGSE------------DSLGVKFF------
Uncinocarpus reesii              EAT------------------ISQEFHGMDPDVLQKSLHT-LVKRGKAQVFGSE------------DQQGVKFF------
Coprinus cinereus                PVE--------------------SPLTGIPVQLLRKAIGI-LGKTGRAQTISIS------------DGEGVRFFAA---K
Phanerochaete chrysosporium      PVP--------------------SQLSGIPMTLLRKAIAV-LTKTSRAQIISVA------------DGEGVRFLAGNTSK
Ustilago maydis                  DLV------------------QAQDFYLLPTPMLRQALKH-LSTQGKAQIFAGTE---------ADDGEGVKFV------
Rhizopus oryzae                  ELA------------------EGQEFYDIDHNVLDKALNV-LVKRGNAQIFKGTD----------EDSMGVKFFQ-----
Blastocladiella emersonii        DYV------------------TDADFYCMDEVLFCRVIQH-LAKQKRVVMVNESK---------VADELGFKF-------
Strongylocentrotus purpuratus    EDT------------------TNEEFHGLEDWLLKRSLKC-LERGRKAELMAF------------DGNEGVKFF------
Caenorhabditis briggsae          DDT------------------TNESFHNLEREILMKALTC-LEDQRRAQLMNI-----------GGDNEGVKFV------
Caenorhabditis elegans           DDT------------------TNESFHNLEREILMKALTC-LEEQRRAQLMNI-----------GGDNEGVKFI------
Heterodera glycines              EDV------------------AKESFYGLDKDVLLKGLQH-LENQGKAVLIDI-----------DGEKGGVKFL------
Paragonimus westermani           EDT------------------ESEPFHGLDNTILLEALRC-LQKRGKAELISD---------------EGVKFLCV----
Schistosoma japonicum            DNT------------------KHEAFHGLDFSILTDAINI-LQKRGQAEIMGD---------------AGVKFFC-----
Schistosoma mansoni              DDT------------------KQEAFHGLDISVLTNAITV-LQKRGQAEIMED---------------EGVKFYC-----
Schistosoma mediterranea         EET------------------ENEPFHGLDKDILISAIRI-LAKNKRAELLIS------------EDLEGVKFFQ-----
Amblyomma variegatum             DDT------------------ADTEFAGLDVDLLRLSLQA-LEKQGKAELITF------------DGSEGVKVF------
Boophilus microplus              DDT------------------ADTEFAGLDVDLLRLSLQT-LEKQGKAELICF------------DGSDGVKFF------
Aedes aegypti                    DHT------------------VGEEFHGLDQTVLKKALKV-LETKGKCELIAF------------DDSEGVKFY------
Anopheles gambiae                DNT------------------VGEEFHGLDEGVLKKALKL-LEGRGKCELIAF------------DDNEGVKFF------
Apis mellifera                   EDT------------------IDQEFYGLDTEILIRALKT-LETNKKAELILF------------DDNQGVKFF------
Acyrthosiphon pisum              SG----------------------DLNGIDDGVLTKALRV-LERRQQAEILTL------------DGGSGVKFF------
Bombyx mori                      DNT------------------ADQEFHNLDMNVLVKALKS-LEAKGRCELMEF------------DDNQGVKFF------
Drosphila melanogaster           ENT------------------SHLDFYGVDEAVLLSALRL-LEEKGRCELIEM------------DGSHGVKFF------
Drosphila pseudoobscura          EST------------------TQMDFHGVDESVLLNALRL-LEEKGRCELIEI------------DGSHGVKFF------
Lutzomyia longipalpis            DNS------------------TSEEFYGLDEGVLRKALGK-LQEKRKCELFD-------------DESGGVKFF------
Ciona intesinalis                PHS------------------SDQEFYALDDQILMQAIQI-LQASGKAELMG---------------TEGVKFFT-----
Molgula tectiformis              ENN------------------KNEEFYELEESLLLRAIKV-LELKNKAELIG---------------NDGVKFF------
Leucoraga erinacea               DDT------------------EGEEFHGLEEWLLLRALQT-LQSERKAEIITL------------ADSKGVKFF------
Danio rerio                      DDT------------------EKEEFHGLEDWMLLRSLQA-LQTDGKAEIITM------------DDGKGVKFF------
Fugu rubripes                    DDT------------------EGEEFHGLEEWMLLRSLQA-LQAEGKAEIITM------------DDGKGVKFF------
Gasterosteus aculeatus           DDT------------------EGEEFHGLEDWMLLRSLQA-LQTDGKAEIIIM------------DDGKGVKFF------
Ictalurus punctatus              DDT------------------ESEEFHGLEEWMLLRALQA-LQADGKAEIITM------------DDGKGVKFF------
Oryzias latipes                  DDT------------------QGEEFHGLEEWMLLRSLQA-LQTDGRAEIISM------------DDGKGVKFF------
Oncorhynchus mykiss              DDT------------------ESEEFHGLEDWMLIRSLQA-LQMDGKAEVISM------------DDGKGVKFF------
Platichthys flesus               DDT------------------EGEEFHGLEDWMLQRSLQA-LQTDGKAEIITL------------DDGXGVKFF------
Pimephales promelas              DDT------------------EKEEFHGLEDWMLLRSLQA-LQADGKAEIISM------------DDGKGVKFF------
Salmo salar                      DDT------------------ESEEFHGLEDWMLIRSLQA-LQMDGKAEVISM------------DDGKGVKFF------
Tetraodon nigroviridis           DET------------------EGEEFHGLEEWMLLRSLQA-LQAEGRAEIITM------------DDGKGVKFF------
Gallus gallus                    DDT------------------ANEEFHGLDEAMLLRALQA-LQQEHKAEIITL------------DDGRGVKFF------
Taeniopygia guttata              DDT------------------ENEEFHGLDEATLLRALQA-LQQEHKAEIITL------------DDGRGVKFF------
Xenopus laevis                   DDT------------------EGEEFHGLDEAMLLRSLEA-LQQEHKAEIITL------------NESRGVKFF------
Xenopus tropicalis               DDT------------------EGEEFHGLDEAMLLRSLEA-LQQEHKAEIILL------------NDSRGVKFF------
Bos taurus                       EDT------------------EDEEFHGLDEATLLRALQA-LQQEHKAEIITV------------SDGRGVKFF------
Canis familiaris                 EDT------------------EDEEFHGLDEATLLRALQA-LQQEHKAEIITV------------SDGRGVKFF------
Equus caballus                   EDT------------------EDEEFHGLDEATLLRALQA-LQQEHKAEIITV------------SDGRGVKFF------
Homo sapiens                     EDT------------------EDEEFHGLDEATLLRALQA-LQQEHKAEIITV------------SDGRGVKFF------
Macaca mulatta                   EDT------------------EDEEFHGLDEATLLRALQA-LQQEHKAEIITV------------SDGRGVKFF------
Monodelphis domestica            DDT------------------EDEEFHGLDEATLLRALQA-LQLEHKAEIITV------------SDGRGVKFF------
Mus musculus                     EDT------------------EDEEFHGLDEATLLRALQA-LQQEHKAEIITV------------SDGRGVKFF------
Oryctolagus cuniculus            EDT------------------EDEEFHGLDEATLLRALQA-LQQEHKAELITI------------SDGRGVKFF------
Rattus norvegicus                EDT------------------EEEEFHGLDEATLLRALQA-LQQEHKAEIITV------------SDGRGVKFF------
Sus scrofa                       EDT------------------EDEEFHGLDEATLLRALQA-LQQEHKAEIITV------------SDGRGVKFF------
consensus                        ddt                     efhgld  vllral   l  hgka vi               d  gvkf
```

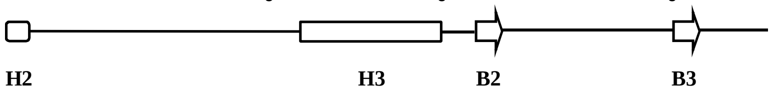

Supplement: Additional File 18 — Additional Figure 14: Comparison of full-length Vps25 amino acid sequences [file 1471-2148-6-59-S18.pdf]
