# Supplementary material for: Genetic structure and evolution of the Vps25 family, a yeast ESCRT-II component
Source: BMC Evol Biol. 2006 Aug 4;6:59. doi: 10.1186/1471-2148-6-59 (PMC1579232; doi:10.1186/1471-2148-6-59)
Supplement: Additional File 19 — Additional Figure 15: Phylogenetic relationship of Vps25 orthologs [file 1471-2148-6-59-S19.pdf]

Additional File 19

Additional Figure 15 - Phylogenetic relationship of Vps25 orthologs

Aligned amino acid sequences [Additional File 18] were used as the basis of phylogenetic analysis. Gaps were removed from the sequence alignment, as well as amino acids flanking longer gaps, where the alignment was uncertain [Additional Data File 5]. Distance tree estimates were then generated under the minimum evolution critereon [34] using MEGA 3.1 software [87]. The bootstrap method [91] was used as a statistical test of the inferred phylogeny, and the percentage of 1000 bootstrap replications supporting each node is indicated. A majority-rule consensus unrooted tree was generated. Each branch with less than 50% statistical support was collapsed to provide emphasis to the reliable portions of the tree.

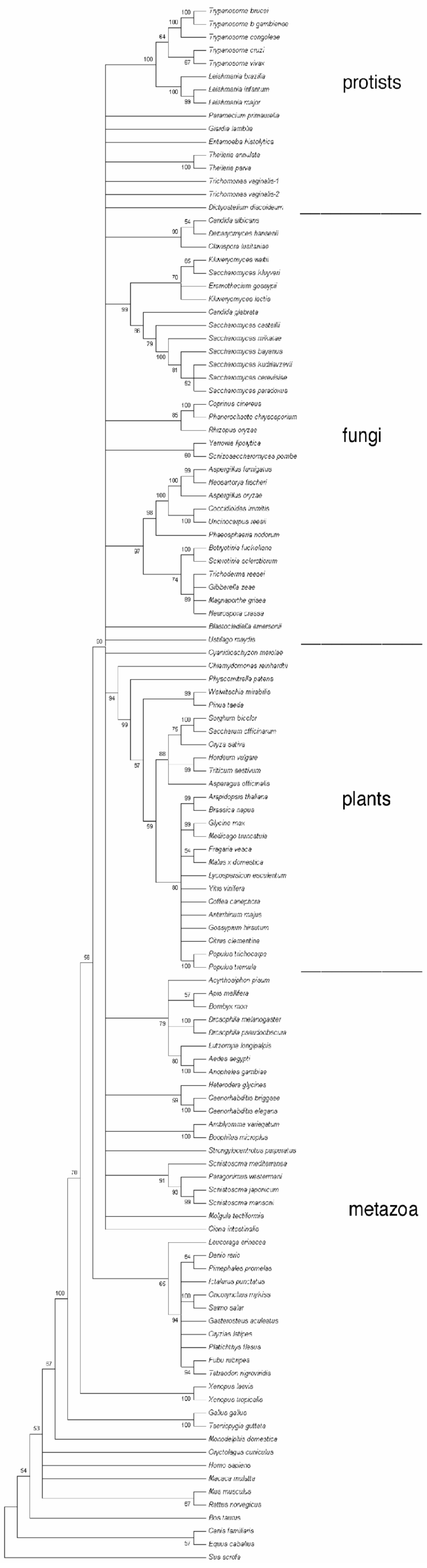
protists
fungi
plants
metazoa
Trypanosome brucei
Trypanosome b gambiense
Trypanosome congolese
Trypanosome cruzi
Trypanosome vivax
Leishmania brazilia
Leishmania infantum
Leishmania major
Paramecium primaurelia
Giardia lamblia
Entamoeba histolytica
Theileria annulata
Theileria parva
Trichomonas vaginalis-1
Trichomonas vaginalis-2
Dictyostelium discoideum
Candida albicans
Debaryomyces hansenii
Clavispora lusitaniae
Kluveryomyces waltii
Saccharomyces kluyveri
Eremothecium gossypii
Kluveryomyces lactis
Candida glabrata
Saccharomyces castellii
Saccharomyces mikatae
Saccharomyces bayanus
Saccharomyces kudriavzevii
Saccharomyces cerevisise
Saccharomyces paradoxus
Coprinus cinereus
Phanerochaete chrysosporium
Rhizopus oryzae
Yarrowia lipolytica
Schizosaccharomyces pombe
Aspergillus fumigatus
Neosartorya fischeri
Aspergillus oryzae
Coccidioides immitis
Uncinocarpus reesii
Phaeosphaeria nodorum
Botryotinia fuckeliana
Sclerotinia sclerotiorum
Trichoderma reesei
Gibberella zeae
Magnaporthe grisea
Neurospora crassa
Blastocladiella emersonii
Ustilago maydis
Cyanidioschyzon merolae
Chlamydomonas reinhardtii
Physcomitrella patens
Welwitschia mirabilis
Pinus taeda
Sorghum bicolor
Saccharum officinarum
Oryza sativa
Hordeum vulgare
Triticum aestivum
Asparagus officinalis
Arapidopsis thaliana
Brassica napus
Glycine max
Medicago truncatula
Fragaria vesca
Malus x domestica
Lycopersicon esculentum
Vitis vinifera
Coffea canephora
Antirrhinum majus
Gossypium hirsutum
Citrus clementine
Populus trichocarpa
Populus tremula
Acyrthosiphon pisum
Apis mellifera
Bombyx mori
Drosophila melanogaster
Drosophila pseudoobscura
Lutzomyia longipalpis
Aedes aegypti
Anopheles gambiae
Heterodera glycines
Caenorhabditis briggsae
Caenorhabditis elegans
Amblyomma variegatum
Boophilus microplus
Strongylocentrotus purpuratus
Schistosoma mediterranea
Paragonimus westermani
Schistosoma japonicum
Schistosoma mansoni
Molgula tectiformis
Ciona intestinalis
Leucoraga erinacea
Danio rerio
Pimephales promelas
Ictalurus punctatus
Oncorynchus mykiss
Salmo salar
Gasterosteus aculeatus
Oryzias latipes
Platichthys flesus
Fubu rubripes
Tetraodon nigroviridis
Xenopus laevis
Xenopus tropicalis
Gallus gallus
Taeniopygia guttata
Monodelphis domestica
Oryctolagus cuniculus
Homo sapiens
Macaca mulatta
Mus musculus
Rattus norvegicus
Bos taurus
Canis familiaris
Equus caballus
Sus scrofa
